# Palladium-catalyzed enantioselective alkenylation of alkenylbenzene derivatives†
†Electronic supplementary information (ESI) available. See DOI: 10.1039/c9sc02380a


**DOI:** 10.1039/c9sc02380a

**Published:** 2019-06-17

**Authors:** Zhi-Min Chen, Jianbo Liu, Jing-Yao Guo, Maximillan Loch, Ryan J. DeLuca, Matthew S. Sigman

**Affiliations:** a Department of Chemistry , University of Utah , 315 South 1400 East , Salt Lake City , Utah 84112 , USA . Email: sigman@chem.utah.edu

## Abstract

A regioselective and enantioselective palladium-catalyzed relay Heck alkenylation of alkenylbenzene derivatives to construct remote stereocenters is disclosed. Various β-substituted styrenes were readily obtained in moderate yields with good to excellent levels of enantioselectivity. This strategy provides rapid access to enantioenriched δ, ε, ζ, and η-alkenyl aryl compounds from simple starting materials. Mechanistic studies suggest that termination of the relay reaction is controlled by affinity of the arene for the Pd complex during migration.

## Abstract 2

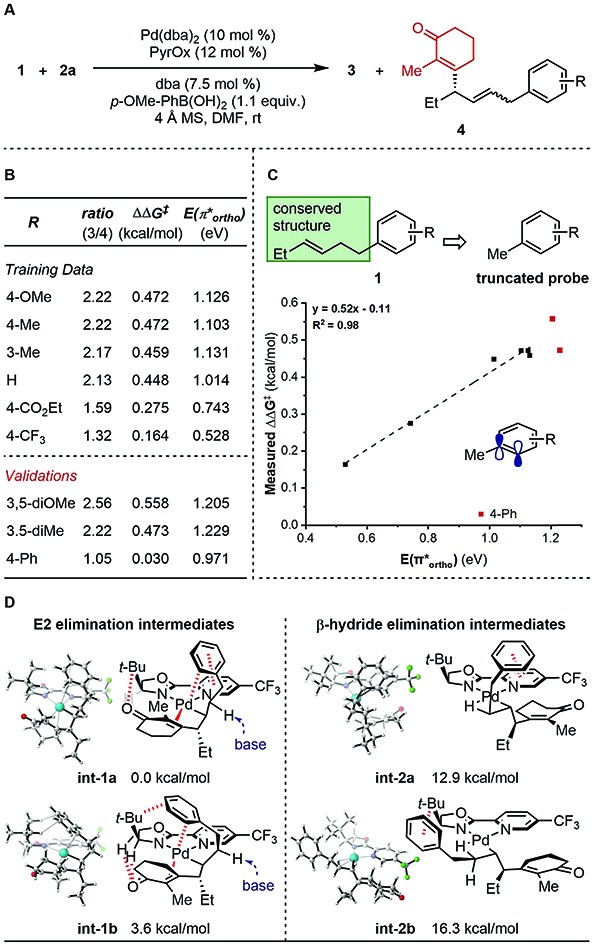
A regioselective and enantioselective palladium-catalyzed relay Heck alkenylation of alkenylbenzene derivatives to construct remote stereocenters is disclosed.

## Introduction

The migration of a metal through an alkyl chain *via* iterative β-hydride elimination/migratory insertion processes, often termed chain walking (or running) offers the opportunity to effectively functionalize multiple sites on a substrate.^1^ Initiation of this process is often accomplished through a metal-catalysed migratory insertion event of an alkene with applications in areas ranging from olefin polymerization^2^ to asymmetric catalysis.^3^ After the initial migratory insertion and subsequent chain walking, termination events are often coupled with either a functionalization of a metal-alkyl intermediate or re-establishing the unsaturation.^4^

**Scheme 1:**
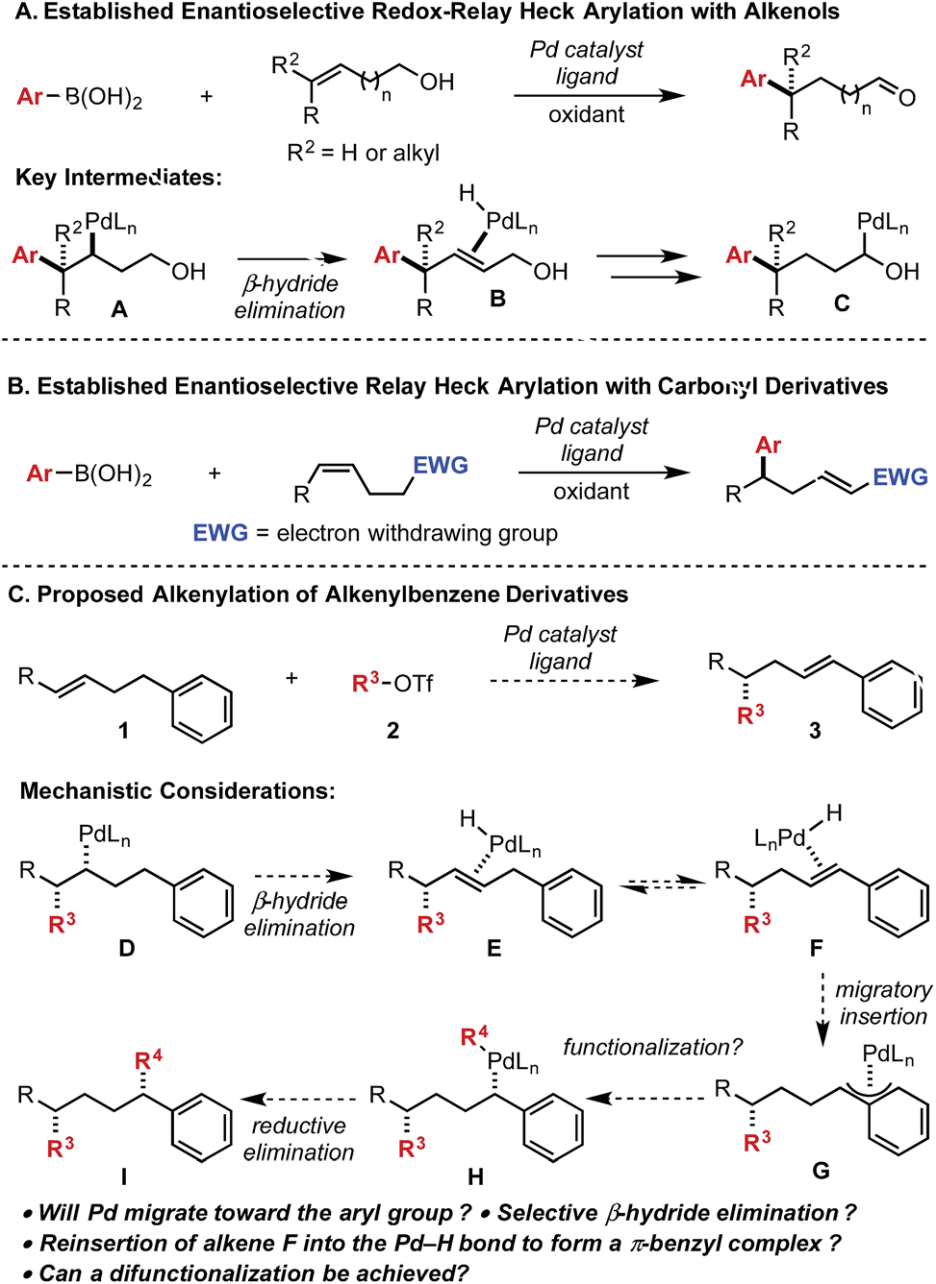
Previous work with carbonyl derivatives and proposed work with alkenylbenzene derivatives.

In this context, our lab has developed various enantioselective Heck-type reactions of di- and tri-substituted alkenes wherein the reaction is terminated by formation of a carbonyl derivative (Scheme 1A).^3^,^5^ The formation of the aldehyde facilitates this in two key regards: (1) the penultimate proposed intermediate (**C**) is thermodynamically stabilized according to computational studies and (2) the resulting aldehyde does not participate in migratory insertion events thus making the process irreversible.^6^ Coupled together, expanding the scope of chain walking events within a Heck platform will likely require similar tactics.

In this regard, our lab disclosed an enantioselective relay Heck arylation of alkenyl carbonyl derivatives, which delivered α,β-unsaturated compounds containing remote stereocenters (Scheme 1B).^7^ The α,β-unsaturated carbonyls are poor ligands compared to the initial electron rich alkene and do not participate in further Heck reactions. Combined, these results provide a basis for exploring other termination possibilities to expand the scope of these enantioselective processes. As an example, we initially envisaged an aryl group could be employed to deliver a styrenyl product (**3**) or, with the addition of a transmetallating reagent, a remotely difunctionalized product (**I**) (Scheme 1C). From a mechanistic perspective, the Pd-alkyl intermediate (**D**) arising from alkene **1** and alkenyl triflate **2** could undergo β-hydride elimination away from the newly formed stereocenter to deliver intermediate **E**. We postulated that alkene **E** would be in equilibrium with alkene **F**, which would ultimately provide the thermodynamically more stable styrene product (**3**) after dissociation of the Pd-hydride. However, styrene **F** could reinsert into the Pd-hydride bond to produce the stabilized π-benzyl intermediate (**G**) that could be intercepted with a transmetallating reagent to afford intermediate **H** followed by reductive elimination to deliver product **I** (in which two new stereocenters were formed). Herein, we demonstrate that the styrene product is favoured and can be produced in high enantioselectivity. Of perhaps more interest, we provide mechanistic evidence to why the reaction terminates in this fashion.

## Results and discussion

To initiate our investigation, Pd_2_(dba)_3_ and a pyridine oxazoline (PyrOx) ligand were combined with alkenylbenzene **1a** and alkenyl triflate **2a** under previously reported relay Heck conditions for substrates of this type (Table 1).^8^ Under these conditions, styrenyl product **3a** was isolated in 42% yield and 96 : 4 er (entry 1). The mass balance was a mixture of the undesired migratory insertion product (insertion at the alkene carbon proximal to the aryl group) and other alkene isomers **4**. Switching the precatalyst to Pd(dba)_2_ led to a slight increase in yield (entry 2) which was further improved to 55% with the addition of 7.5 mol% dba (entry 3).^9^ Additionally, during the course of our optimization, the effect of phenylboronic acid addition was evaluated (to explore the possibility of a remote difunctionalization reaction), which resulted in an increase in the isolated yield of styrenyl product **3a** (entry 4). Lastly, the addition of electron-rich *para*-methoxyphenylboronic acid improved the yield to 69% (entry 5). It should be noted that no remote difunctionalization product was observed with either arylboronic acid additive (entries 4 and 5); however, the yield of the styrenyl product is increased.

**Table 1:** Reaction optimization^*a*^

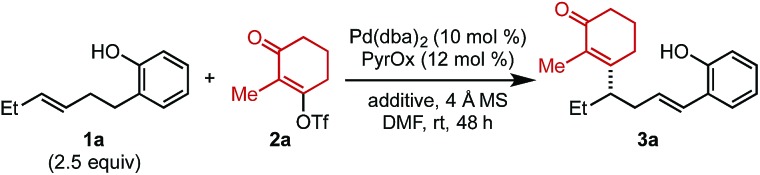			
Entry	Additive (mol%)	% Yield	Er
1^*b*^	—	42	96 : 4
2	—	48	95.5 : 4.5
3	dba (7.5)	55	95.5 : 4.5
4	dba (7.5) + **5a** (110)	60	95.5 : 4.5
5	dba (7.5) + **5b** (110)	69	95.5 : 4.5
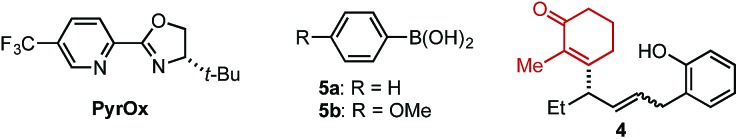			

^*a*^Each entry represents the isolated yield on 0.2 mmol scale. Er values were determined by SFC.

^*b*^Pd_2_(dba)_3_ (5 mol%) was used.

After optimized conditions were identified (entry 5), the scope of the alkenylbenzene substrate was explored (Table 2). In general, the desired styrenyl products were obtained with moderate yields and excellent enantiomeric ratios. Again, alkenyl triflate **2a** was completely converted and the mass balance was mainly composed of alkene isomers **4** (a putative explanation is discussed below). A phenyl group on the substrate (**1b**) gave the desired styrenyl product in 64% and 96.5 : 3.5 er. A variety of electron-rich aryl groups were well-tolerated including *p*-methyl (**3c**), *p*-methoxy (**3d**), *o*-methoxy (**3e**), *p*-hydoxy (**3f**), and *p*-dimethylamino (**3g**).

**Table 2:** Evaluation of alkenylbenzene substrates^*a*^

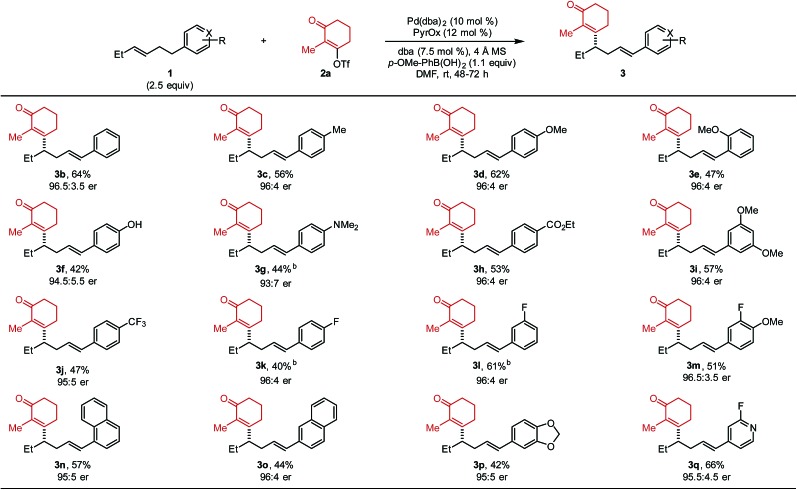

^*a*^Each entry represents the isolated yield on 0.2 mmol scale. The er values were determined by SFC.

^*b*^No *p*-methoxyphenylbronoic acid was added.

Additionally, electron-withdrawing substituents such as an ethyl ester (**3h**), two methoxy groups at the *meta* positions (**3i**), and a trifluoromethyl group (**3j**) were all competent. A fluorine atom was readily incorporated on the aromatic ring (**3k–3m**). Substrates containing naphthyl groups (**1n** and **1o**) produced the desired products **3n** and **3o** in 57% and 44% yield, respectively. A benzodioxole group (**1p**) was successfully incorporated delivering styrenyl product **3p** in 42% yield and 95 : 5 er. Lastly, a fluoropyridyl group (**1q**) delivered the corresponding product (**3q**) in 66% yield. Trisubstituted alkene substrates were also evaluated under the reaction conditions but no desired product was observed. It should be noted that the absolute configuration of **3d** was determined to be (*S*) and thus the rest of the products were assigned as (*S*) by analogy.^10^

Next, the scope of alkenyl triflates was investigated (Table 3). For example, 5,5-dimethylcyclohexenone triflate (**2r**) and 2-bromo-substituted triflate **2s** gave the corresponding products (**3r** and **3s**) in 41% and 30% yield, respectively. An enol triflate containing a methyl-substituted cyclopentenone (**2t**) delivered product **3t** in 45% yield and 97.5 : 2.5 er. Reaction with β-keto ester derived triflates **1u** and **1v** yielded the desired products (**3u**, **3v**) in 52% and 60% yield, respectively. A β-keto ester derived triflate containing a Boc-protected amine (**1w**) produced **3w** in 61% yield and 98 : 2 er. Additionally, an enol triflate containing a phthalimide (**1x**) furnished the desired styrenyl product in 51% yield and 99 : 1 er. The isolated yields of products **3u**, **3v**, and **3w** were determined after hydrogenation, as a mixture of alkene isomers were produced and complicated purification.

**Table 3:** Evaluation of alkenyl triflate scope^*a*^

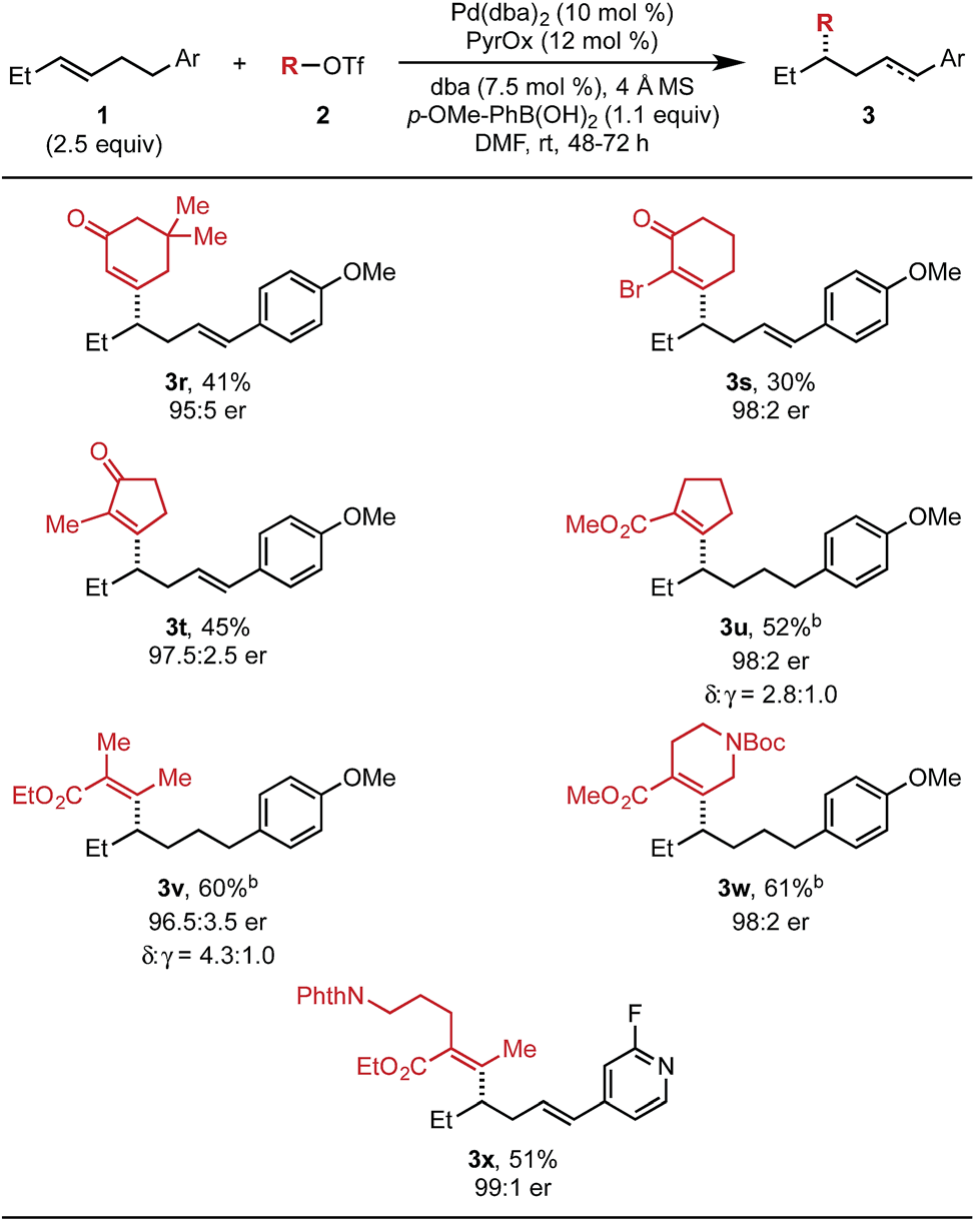

^*a*^Each entry represents the isolated yield on 0.2 mmol scale. Er values were determined by SFC.

^*b*^The isolated yield was determined after hydrogenation using Pd/C (2.5 mol%), H_2_ (balloon), in MeOH.

The effect of chain length on yield and selectivity was determined by comparing three additional substrates containing additional methylene units between the alkene and the aryl moiety (**1y–1aa**) (Table 4). When bishomoallylic aryl compound **1y** was subjected to the standard reaction conditions followed by hydrogenation, product **3y** was obtained in 63% (2.6 : 1.0 ratio of insertion products). Trishomoallylic aryl compound **1z** delivered product **3z** in similar yield and insertion selectivity, albeit with a slightly diminished er. Lastly, when the substrate contained an additional methylene unit (**1aa**), product **3aa** was observed in 57% yield and reduced site selectivity (2.0 : 1.0 mixture of migratory insertion products). On the basis of our previous work,5*b* using (*Z*)-alkene substrates, the opposite enantiomer would be produced compared to reaction of the (*E*)-alkene. Thus, the absolute configuration of **3z** and **3aa** were assigned as (*R*).

**Table 4:** Evaluation of chain length^*a*^

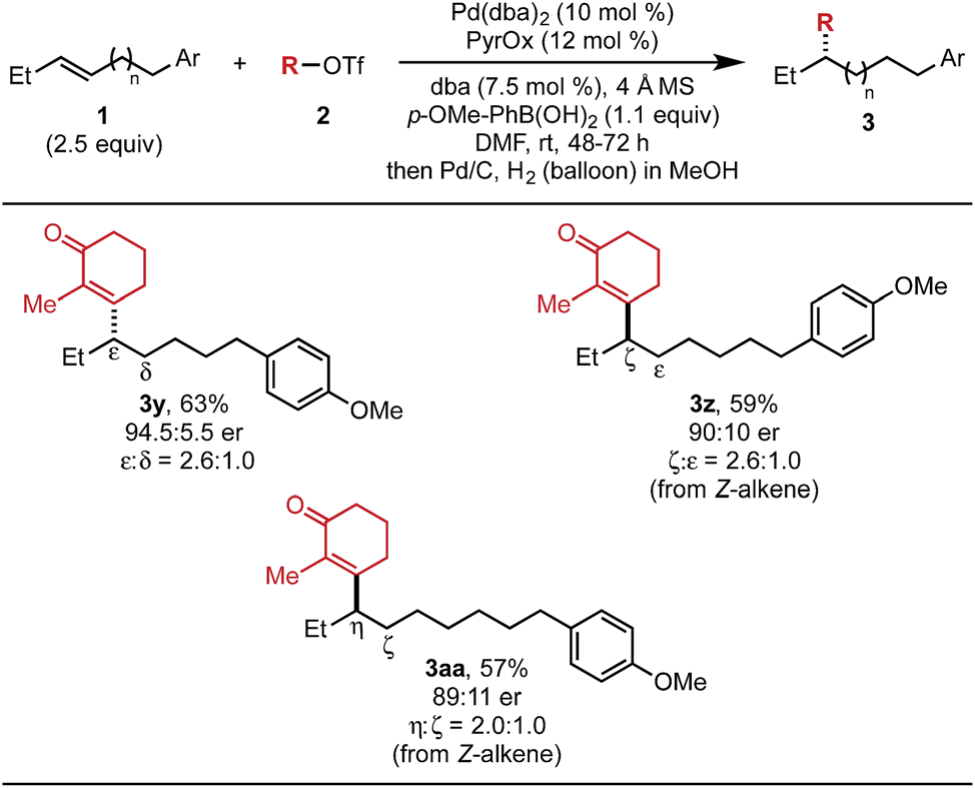

^*a*^Each entry represents the isolated yield on 0.2 mmol scale. Er values were determined by SFC.

^*b*^The isolated yield was determined after hydrogenation using Pd/C (2.5 mol%), H_2_ (balloon), in MeOH.

As alluded to above, interesting features of the reaction include the observation of alkene isomers using several of the differentiated aryl substrates as well as the inability to capture Pd-alkyl intermediates through a subsequent cross-coupling. Therefore, we set out to interrogate the reaction mechanism with a focus on why the formation of the styrene product generally terminates the chain walking event(s). To initiate this investigation, deuterated alkenylbenzene **1y–D_2_** was subjected to the optimized reaction conditions with alkenyl triflate **2a** (Table 5). The goal of this study was to determine the dynamics of the chain walking process. In the event, mono-deuterated products **3y(H–D)** and **3y(D–H)** were observed in 23% and 12% yield, respectively. In addition, di-deuterated product **3y(D–D)** was also observed in 12% yield. This mixture suggests the palladium catalyst undergoes reversible β-hydride elimination/migratory insertion at the benzylic position of the substrate, which results in the scrambling of the deuterium atoms.

**Table 5:** Deuterium labelling study^*a*^

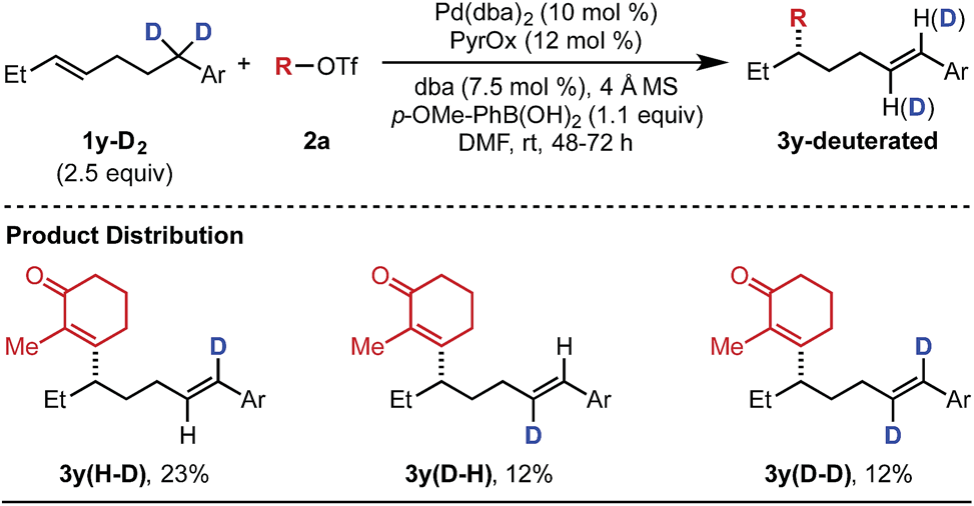

^*a*^Each entry represents the isolated yield on 0.2 mmol scale.

**Fig. 1:**
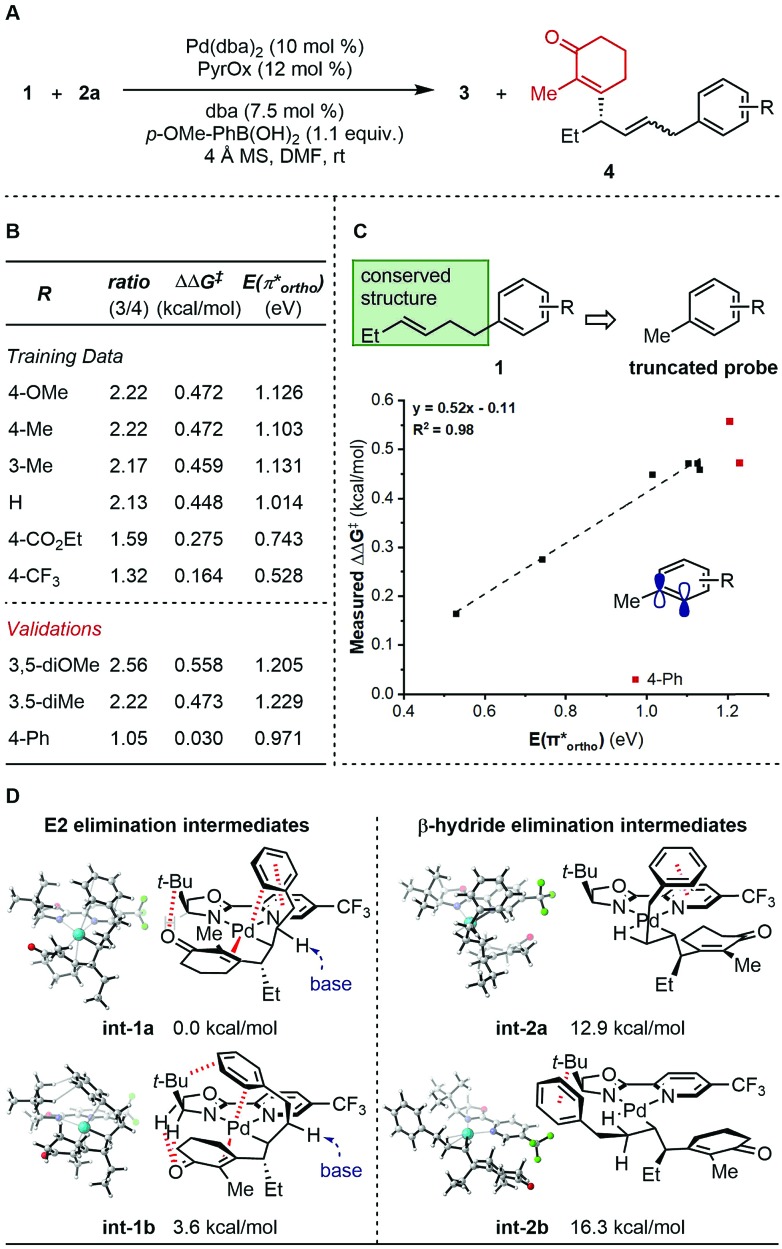
Analysis of side product formation. (A) Reaction scheme. (B) Experimental data. (C) Quantitative analysis. (D) Proposed original of side products.

To further probe the mechanism, we investigated the effect of various features of the terminating aryl group on the selective formation of styrenes. Specifically, a ratio of alkene products **3** and **4** was observed as a function of the aryl group (Fig. 1A). To probe this relationship, systematic changes of the terminal aryl on this ratio were evaluated while conserving the remainder of the reaction features (Fig. 1B), and the results were compared to a number of DFT-derived physical organic parameters. These were calculated using a simple aryl model system depicted in Fig. 1C. A correlation was identified between the observed product selectivity (represented as ΔΔ*G*^‡^) and the energy of an empty π* orbital as calculated by natural bond orbital (NBO) analysis (Fig. 1C). To validate this correlation, two extrapolations were tested that provide modestly improved selectivity. However, a substrate containing a *para*-Ph substituent performed in an unpredictable manner.

The discovery that relative π* orbital energy is correlative with the selectivity of alkene isomers formed, suggested that Pd may coordinate with the arene while chain walking. As the arene becomes less donating, the back-bonding interaction of the π-system with Pd, presumably favourable for the formation of the side product **4**, is enhanced.^11^ To further inspect this hypothesis, computational optimization of intermediate structures was performed. The cationic organopalladium intermediate stabilized by interactions with both the alkenyl and the terminal aryl group is calculated to be >10 kcal mol^–1^ lower in energy than the conformer leading up to β-hydride elimination, thus significantly raising the energy barrier of the chain walking process (Fig. 1D). Additionally, computational results support that, through the proposed palladium–Ar bonding interaction, the γ-palladium intermediates are stabilized in conformations prone to E2 elimination, with the a H–C–C–Pd dihedral angle of **int-1a** (*φ***_int-1a_**) being 160.8°, and *φ***_int-1b_** being 178.5° (see ESI† for further details), which through solvent mediated deprotonation will lead to minor products **4**. A low-energy aryl π* orbital, which allows for strong back-bonding, can lead to both enhanced stabilizing effect, and an early transition state of the proposed E2 elimination process. The unexpected low selectivity observed for the *para*-Ph-C_6_H_4_ substrate can be explained by the additional non-covalent interactions of the *para*-position phenyl group with the ligand backbone, which further stabilizes the organopalladium intermediate for elimination.^12^,5d

## Conclusions

In summary, we have successfully developed an intermolecular enantioselective alkenylative relay Heck reaction of alkenylbenzene derivatives to synthesize enantiomerically enriched β-substituted styrenes in good to high enantioselectivity. Preliminary mechanistic studies suggest reversible β-hydride elimination/migratory insertion to arrive at the thermodynamic styrenyl product. This information will provide the foundation for the future development of new termination strategies using a metal-chain walking reaction process.

## Conflicts of interest

There are no conflicts to declare.

## Supplementary Material

Supplementary information
