# Supplementary material for: Palladium-catalyzed enantioselective alkenylation of alkenylbenzene derivatives
Source: Chem Sci. 2019 Jun 17;10(30):7246–50. doi: 10.1039/c9sc02380a (PMC6685350; doi:10.1039/c9sc02380a)
Supplement: Supplementary file 1 [file SC-010-C9SC02380A-s001.pdf]

Electronic Supplementary Material (ESI) for Chemical Science.
This journal is © The Royal Society of Chemistry 2019

Palladium-catalyzed enantioselective alkenylation of alkenylbenzene derivatives

Zhi-Min Chen, [‡§a] Jianbo Liu, [‡a] Jing-Yao Guo, Maximillan Loch, Ryan DeLuca, Matthew S. Sigman *

Department of Chemistry, University of Utah, 315 South 1400 East, Salt Lake City, UT 84112, USA

Table of Contents

General Considerations S2
Substrate Preparation S3
Enantioselective Relay Heck Reaction S19
Determination of Absolute Configuration S37
Computational Analysis of Side Product Formation S41
Computational Methods S42
References S42
Table of Descriptors S44
Cartesians S45
SAPT Energies S47
SFC Traces S49
^{1}H and ^{13}C NMR Spectra S76

General Considerations:

All reactions were performed using oven-dried or flame-dried glassware equipped with a magnetic stir bar under an atmosphere of nitrogen unless otherwise noted. All reagents were purchased from commercial suppliers and used without further purification. Dichloromethane, tetrahydrofuran (THF), and acetonitrile were passed through a column of activated alumina immediately prior to use. Methanol was distilled from calcium hydride. The ligand was synthesized according to the literature procedure.[1] ^{1}H NMR spectra were obtained in $CDCl_3$ at 300 MHz, 400 MHz or 500 MHz. Chemical shifts are reported in ppm and referenced to the $CHCl_3$ singlet at 7.26 ppm. ^{13}C NMR spectra were obtained in $CDCl_3$ at 75 MHz, 100 MHz or 125 MHz and referenced to the center peak of the $CDCl_3$ triplet at 77.16 ppm. ^{19}F NMR spectra were obtained at 282 MHz ($CFCl_3$ as the outside standard and low field is positive). The abbreviations s, d, t, quint, sext, sept, dd, ddd, dt, and m stand for the resonance multiplicities singlet, doublet, triplet, quintet, sextet, septet, doublet of doublets, doublet of doublet of doublets, doublet of triplets, and multiplet, respectively. Thin-layer chromatography was performed with EMD silica gel 60 F_{254} plates eluting with solvents indicated, visualized by a 254 nm UV lamp and stained with phosphomolybdic acid (PMA). Flash chromatography was performed using EM reagent silica 60 (230-400 mesh). GC separations were performed with an HP6890 GC with a flame ionization detector equipped with a DB-5 column using a 50:1 split. IR spectra were recorded using a Thermo Nicolet FT-IR. HRMS data were obtained on a Waters LCP Premier XE instrument by ESI/TOF. LRGC-MS data were obtained on an Agilent Technologies 5975c VL MSD instrument. SFC (supercritical fluid chromatography) analysis was performed at 40 °C, using a Thar instrument fitted with AD-H and OD columns.

Synthesis of Triflates and Substrates

Synthesis of Triflates: 2a-2h

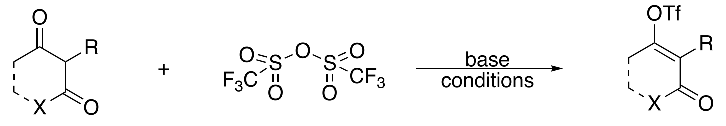

2a: 2-methyl-3-oxocyclohex-1-en-1-yl trifluoromethanesulfonate

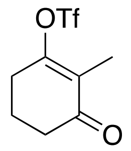

This triflate was prepared according to the literature procedure.[2] Analytical data matches with the literature.

2b: 5,5-dimethyl-3-oxocyclohex-1-en-1-yl trifluoromethanesulfonate

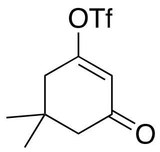

This triflate was prepared according to the literature procedure.[2] Analytical data matches with the literature.

2c: 2-bromo-3-oxocyclohex-1-en-1-yl trifluoromethanesulfonate

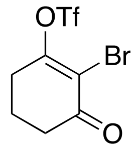

This triflate was prepared according to the literature procedure.[3] Analytical data matches with the literature.

2d: 2-methyl-3-oxocyclopent-1-en-1-yl trifluoromethanesulfonate

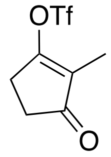

This triflate was prepared according to the literature procedure.[4] Analytical data matches with the literature.

2e: methyl 2-(((trifluoromethyl)sulfonyl)oxy)cyclopent-1-ene-1-carboxylate

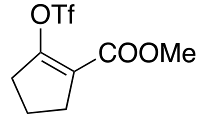

This triflate was prepared according to the literature procedure.[5] Analytical data matches with the literature.

2f: ethyl (Z)-2-methyl-3-(((trifluoromethyl)sulfonyl)oxy)but-2-enoate

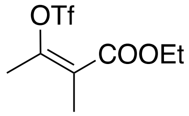

This triflate was prepared according to the literature procedure.[6] Analytical data matches with the literature.

2g: 1-(*tert*-butyl) 4-methyl 5-(((trifluoromethyl)sulfonyl)oxy)-3,6-dihydropyridine-1,4(2*H*)-dicarboxylate

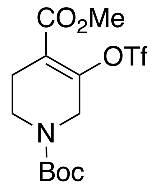

This triflate was prepared according to the literature procedure.[7] Analytical data matches with the literature.

2h: ethyl (*Z*)-5-(1,3-dioxoisoindolin-2-yl)-2-(1-(((trifluoromethyl)sulfonyl)oxy)ethylidene)-pentanoate

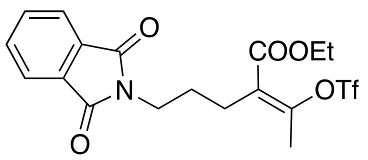

This triflate was prepared according to the literature procedure.[8] Analytical data matches with the literature.

Synthesis of Alkene Substrates: 1a-1dd

(Prepared according to a modified literature procedure)[9]

General Procedure A:

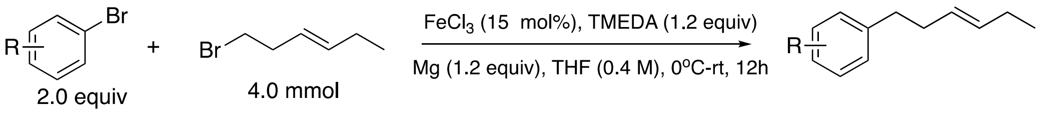

A 50 mL flask was placed in a water bath (rt), charged with magnesium turnings (116 mg, 4.8 mmol), fitted with a rubber septum, and purged with argon. A solution of $FeCl_3$ (97.2 mg, 0.6 mmol, 15 mol%) in dry THF (10 mL) was added via syringe followed by TMEDA (724 µL, 4.8 mmol). The mixture was stirred at rt for 20 min. Then, the reaction was cooled to 0 °C, and arylbromide (8.0 mmol) and alkylbromide (4.0 mmol) were added. After 12 h at rt, the reaction was quenched with saturated aqueous NH_4Cl (10 mL) and aqueous HCl (10 %, 4 mL) and extracted with ethyl acetate (3 × 20 mL). The combined organic phases were dried (Na_2SO_4), concentrated in vacuo, and subjected to flash chromatography (SiO_2/cyclohexane/ethyl acetate).

General Procedure B:

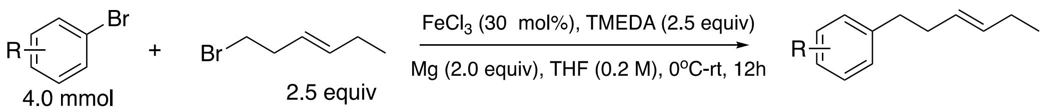

A 50 mL flask was placed in a water bath (rt), charged with magnesium turnings (192 mg, 8.0 mmol), fitted with a rubber septum, and purged with argon. A solution of $FeCl_3$ (194 mg, 1.2 mmol, 30 mol%) in dry THF (20 mL) was added via syringe followed by TMEDA (1.5 mL, 10.0

mmol). The mixture was stirred at rt for 20 min. Then, the reaction was cooled to 0 °C, and arylbromide (4 mmol) and alkylbromide (10.0 mmol) were added. After 12 h at rt, the reaction was quenched with saturated aqueous NH_4Cl (10 mL) and aqueous HCl (10 %, 4 mL) and extracted with ethyl acetate (3 × 20 mL). The combined organic phases were dried (Na_2SO_4), concentrated in vacuo, and subjected to flash chromatography (SiO_2/hexane/CH_2Cl_2).

(*E*)-1-bromohex-3-ene (**1ba**)

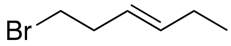

To a solution of *trans*-3-hexane-1-ol (9.0 g, 90.0 mmol) in CH_2Cl_2 (90 mL) was added PPh_3 (triphenylphosphine, 24.6 g, 93.6 mmol). NBS (*N*-bromo-succinimide, 16.8 g, 93.6 mmol) was added in batches to the colorless mixture over 40 minutes. The yellow solution was stirred for 24 h at rt. After this time, the solution was passed through a short silica plug to remove any solid and the filtrate was concentrated to afford a yellow oil. The oil was purified by chromatography on silica gel (gradient elution: hexanes), and concentrated in vacuo carefully to afford product **1ba** as a colorless oil (10.4 g, 71% yield): $\mathbf{R_f}$ = 0.70 (hexanes). NMR data match the one reported in the literature.[10] ^{1}H NMR (500 MHz, $CDCl_3$) δ 5.59 (dt, *J* = 15.3, 6.3 Hz, 1 H), 5.39 (dt, *J* = 15.3, 6.7 Hz, 1 H), 3.37 (t, *J* = 7.2 Hz, 2 H), 2.55 (q, *J* = 6.8 Hz, 2 H), 2.03 (quintet, *J* = 6.7 Hz, 2 H), 0.99 (t, *J* = 7.4 Hz, 3 H); ^{13}C NMR (125 MHz, $CDCl_3$) δ 135.4, 125.4, 36.0, 32.9, 25.5, 13.6.

(*E*)-1-(hex-3-en-1-yl)-2-methoxybenzene (**1e**):

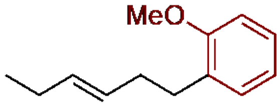

<u>The general procedure A</u> was followed using 1-bromo-2-methoxybenzene (**1se**, 14.96 g, 80 mmol) and (*E*)-1-bromohex-3-ene (**1ba**, 6.480 g, 40 mmol) in 40 mmol scale. Purification of this material by chromatography on silica gel (gradient elution: 2~20% CH_2Cl_2/hexanes) afforded product **1e** as a colorless oil (3.75 g, 49% yield): $\mathbf{R_f}$ = 0.50 (25% CH_2Cl_2/hexanes); **^{1}H NMR** (500 MHz, $CDCl_3$) δ 7.25-7.12 (m, 2H), 6.98-6.82 (m, 2H), 5.59 – 5.42 (m, 2H), 3.84 (s, 3H), 2.70 (t, *J* = 7.5 Hz, 2H), 2.35 – 2.24 (m, 2H), 2.08 – 1.96 (m, 2H), 0.99 (t, *J* = 7.5 Hz, 3H); **^{13}C NMR** (125 MHz, $CDCl_3$) δ 157.4, 132.2, 130.6, 129.8, 128.9, 126.9, 120.2, 110.2, 55.2, 32.7, 30.5, 25.6, 13.9; **IR** (neat) 2960, 2835, 1601, 1493, 1463, 1240, 1179, 1051, 1032, 965, 748

cm^{-1}; **HRMS** (ESI-TOF) *m/z* calcd for $C_{13}H_{19}O$ $(M+H)^+$: 191.1436, found 191.1439.

(*E*)-4-(hex-3-en-1-yl)phenol(**1a**)[11]

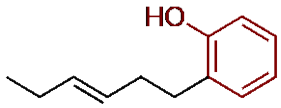

Under a nitrogen atmosphere, a 60% dispersion of NaH (0. 93 g, 23.3 mmol) in oil was washed with dry hexanes (20 mL) and dry ether (20 mL), and then the NaH was suspended in dry DMF (20 mL). A solution of ethanethiol (2.0 mL, 27 mmol) in DMF (20 mL) was added and when gas evolution had ceased, **1e** (1.92 g, 10 mmol) in DMF (10 mL) was added. The mixture was heated at reflux for 2 h, cooled in an ice bath, and carefully neutralized with aqueous 10% HCl. The neutralized solution was extracted with ether (5 X 100 mL) and the ethereal extracts were washed with water (150 mL) and brine (3 X 100 mL). The resulting pale yellow solution was dried and evaporated to leave an orange oil, which was purified by chromatography on silica gel (gradient elution: 10~35% CH_2Cl_2/hexanes) afforded product **1a** as a colorless oil (1.40 g, 78% yield): **R*f*** = 0.40 (50% CH_2Cl_2/hexanes); **^{1}H NMR** (300 MHz, $CDCl_3$) δ 7.20-7.00 (m, 2H), 6.88 (t, *J* = 6.0 Hz, 1H), 6.78 (d, *J* = 9.0 Hz, 1H), 5.60 – 5.40 (m, 2H), 4.63 (br, 1H), 2.68 (t, *J* = 7.5 Hz, 2H), 2.38 – 2.23 (m, 2H), 2.09 – 1.94 (m, 2H), 0.97 (t, *J* = 7.5 Hz, 3H); **^{13}C NMR** (75 MHz, $CDCl_3$) δ 153.4, 133.1, 130.3, 128.4, 128.0, 127.1, 120.8, 115.3, 32.7, 30.4, 25.6, 13.8; **IR** (neat) 3436, 2961, 2930, 1613, 1591, 1454, 1232, 1094, 965, 748 cm^{-1}; **HRMS** (ESI-TOF) *m/z* calcd for $C_{12}H_{17}O$ $(M+H)^+$: 177.1279, found 177.1285.

(*E*)-hex-3-en-1-ylbenzene (**1b**):

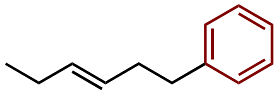

The general procedure A was followed using bromobenzene (**1sb**, 1256 mg, 8.0 mmol) and (*E*)-1-bromohex-3-ene (**1ba**, 648 mg, 4.0 mmol). Purification of this material by chromatography on silica gel (gradient elution: hexanes) afforded product **1b** as a colorless oil (332 mg, 52% yield): **R*f*** = 0.50 (hexanes); **^{1}H NMR** (500 MHz, $CDCl_3$) δ 7.35– 7.16 (m, 5H), 5.56 – 5.42 (m, 2H), 2.70 (t, *J* = 7.5 Hz, 2H), 2.38 – 2.29 (m, 2H), 2.08 – 1.96 (m, 2H), 0.99 (t, *J* = 7.5 Hz, 3H); **^{13}C NMR** (125 MHz, $CDCl_3$) δ 142.2, 132.6, 128.4, 128.3, 128.2, 125.7, 36.2, 34.4, 25.6, 13.9; **IR**

(neat) 2958, 2923, 1454, 965, 746, 698 cm^{-1}; **HRMS** (ESI-TOF) *m/z* calcd for $C_{12}H_{17}$ $(M+H)^+$: 161.1330, found 161.1331.

(*E*)-1-(hex-3-en-1-yl)-4-methylbenzene (**1c**):

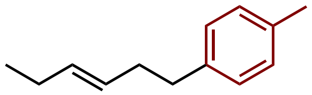

The general procedure A was followed using 1-bromo-4-methylbenzene (**1sc**, 1368 mg, 8.0 mmol) and (*E*)-1-bromohex-3-ene (**1ba**, 648 mg, 4.0 mmol). Purification of this material by chromatography on silica gel (gradient elution: hexanes) afforded product **1c** as a colorless oil (418 mg, 60% yield): R_f = 0.50 (hexanes); **^{1}H NMR** (500 MHz, $CDCl_3$) δ 7.24 – 7.05 (m, 4H), 5.56 – 5.35 (m, 2H), 2.65 (t, *J* = 7.5 Hz, 2H), 2.35 – 2.22 (m, 5H), 2.08 – 1.96 (m, 2H), 0.98 (t, *J* = 7.5 Hz, 3H); **^{13}C NMR** (125 MHz, $CDCl_3$) δ 139.2, 135.0, 132.5, 128.9, 128.5, 128.3, 35.7, 34.6, 25.6, 21.0, 13.9; **IR** (neat) 2961, 2922, 1515, 1453, 965, 804 cm^{-1}; **HRMS** (ESI-TOF) *m/z* calcd for $C_{13}H_{19}$ $(M+H)^+$: 175.1487, found 175.1501.

(*E*)-1-(hex-3-en-1-yl)-4-methoxybenzene (**1d**):

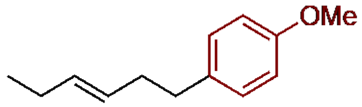

The general procedure A was followed using 1-bromo-4-methoxybenzene (**1sd**, 1496 mg, 8.0 mmol) and (*E*)-1-bromohex-3-ene (**1ba**, 648 mg, 4.0 mmol). Purification of this material by chromatography on silica gel (gradient elution: 2~20% CH_2Cl_2/hexanes) afforded product **1d** as a colorless oil (395 mg, 52% yield): R_f = 0.40 (25% CH_2Cl_2/hexanes); **^{1}H NMR** (500 MHz, $CDCl_3$) δ 7.12 (d, *J* = 5.0 Hz, 2H), 6.84 (d, *J* = 5.0 Hz, 2H), 5.59 – 5.35 (m, 2H), 3.80 (s, 3H), 2.63 (t, *J* = 10.0 Hz, 2H), 2.35 – 2.22 (m, 2H), 2.08 – 1.96 (m, 2H), 0.98 (t, *J* = 7.5 Hz, 3H); **^{13}C NMR** (125 MHz, $CDCl_3$) δ 157.7, 134.3, 132.6, 129.3, 128.4, 113.6, 55.2, 35.2, 34.7, 25.6, 13.9; **IR** (neat) 2962, 2930, 1601, 1509, 1454, 1221, 1157, 965, 823 cm^{-1}; **HRMS** (ESI-TOF) *m/z* calcd for $C_{13}H_{19}O$ $(M+H)^+$: 191.1436, found 191.1437.

(*E*)-4-(hex-3-en-1-yl)phenol(**1f**)[11]

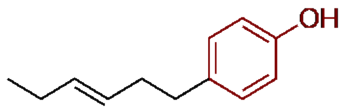

Under a nitrogen atmosphere, a 60% dispersion of NaH (0.933 g, 23.3 mmol) in oil was washed with dry hexanes (10 mL) and dry ether (10 mL), and then the NaH was suspended in dry DMF (16mL). A solution of ethanethiol (1.95 mL, 27 mmol) in DMF (23 mL) was added and when gas evolution had ceased, **1d** (1.92 g, 10.0 mmol) in DMF (13 mL) was added. The mixture was heated at reflux for 2 h, cooled in an ice bath, and carefully neutralized with aqueous 10% HCl. The neutralized solution was extracted with ether (5 X 50 mL) and the ethereal extracts were washed with water (150 mL) and brine (3 X 50 mL). The resulting pale yellow solution was dried and evaporated to leave an orange oil, which was purified by chromatography on silica gel (gradient elution: 10~35% CH_2Cl_2/hexanes) afforded product **1f** as a colorless oil (1.43 g, 80% yield): **R*f*** = 0.30 (50% CH_2Cl_2/hexanes); **^{1}H NMR** (500 MHz, $CDCl_3$) δ 7.05 (d, *J* = 5.0 Hz, 2H), 6.75 (d, *J* = 10.0 Hz, 2H), 5.52 – 5.32 (m, 2H), 4.65 (br, 1H), 2.60 (t, *J* = 7.5 Hz, 2H), 2.30 – 2.20 (m, 2H), 2.03 – 1.91 (m, 2H), 0.96 (t, *J* = 7.5 Hz, 3H); **^{13}C NMR** (125 MHz, $CDCl_3$) δ 153.4, 134.5, 132.6, 129.5, 128.4, 115.0, 35.2, 34.6, 25.6, 13.9; **IR** (neat) 3369, 2961, 2929, 1701, 1613, 1513, 1441, 1359, 1221, 1171, 966, 827 cm^{-1}; **HRMS** (ESI-TOF) *m/z* calcd for $C_{12}H_{17}O$ $(M+H)^+$: 177.1279, found 177.1282.

(*E*)-4-(hex-3-en-1-yl)-*N,N*-dimethylaniline (**1g**):

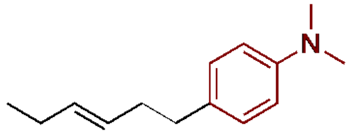

<u>The general procedure A</u> was followed using 4-bromo-*N,N*-dimethylaniline (**1sg**, 1592 mg, 8.0 mmol) and (*E*)-1-bromohex-3-ene (**1ba**, 648 mg, 4.0 mmol). Purification of this material by chromatography on silica gel (gradient elution: 2~20% CH_2Cl_2/hexanes) afforded product **1g** as a colorless oil (503 mg, 62% yield): **R*f*** = 0.30 (25% CH_2Cl_2/hexanes); **^{1}H NMR** (500 MHz, $CDCl_3$) δ 7.11 (d, *J* = 10.0 Hz, 1H), 6.74 (d, *J* = 10.0 Hz, 1H), 5.60 – 5.42 (m, 2H), 2.95 (s, 6H), 2.63 (t, *J* = 7.5 Hz, 2H), 2.35 – 2.24 (m, 2H), 2.12 – 1.98 (m, 2H), 1.02 (t, *J* = 7.5 Hz, 3H); **^{13}C NMR** (125 MHz, $CDCl_3$) δ 149.0, 132.3, 130.5, 129.0, 128.8, 113.0, 40.9, 35.2, 34.8, 25.6, 13.9; **IR** (neat) 2960, 2917, 2846, 1615, 1519, 1443, 1341, 1223, 1162, 965, 947, 803 cm^{-1}; **HRMS** (ESI-TOF) *m/z* calcd for $C_{14}H_{22}N$ $(M+H)^+$: 204.1752, found 204.1757.

ethyl (*E*)-4-(hex-3-en-1-yl)benzoate (**1h**)

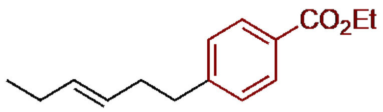

A 50 mL flask was placed in a water bath (rt), charged with magnesium turnings (144 mg, 6.0 mmol), fitted with a rubber septum, and purged with argon. A solution of $FeCl_3$ (146 mg, 0.9 mmol, 30 mol %) in dry THF (15 mL) was added via syringe followed by TMEDA (280 mg, 2.4 mmol). The mixture was stirred at rt for 20 min. Then, the reaction was cooled to 0 °C, and ethyl 4-bromobenzoate (**1sh**, 687 mg, 3 mmol) and (*E*)-1-bromohex-3-ene (**1ba**, 1215 mg, 7.5 mmol) were added. After 12 h at rt, the reaction was quenched with saturated aqueous NH_4Cl (10 mL) and aqueous HCl (10 %, 4 mL) and extracted with ethyl acetate (3 × 20 mL). The combined organic phases were dried (Na_2SO_4), concentrated in vacuo, and subjected to flash chromatography (SiO_2/hexane/CH_2Cl_2) to afford product **1h** as a colorless oil (341 mg, 49% yield): $\mathbf{R}_f$ = 0.35 (50% CH_2Cl_2/hexanes); **^{1}H NMR** (300 MHz, $CDCl_3$) δ 7.96 (d, *J* = 9.0 Hz, 2H), 7.23 (d, *J* = 6.0 Hz, 2H), 5.55 – 5.32 (m, 2H), 4.36 (q, *J* = 8.0 Hz, 2H), 2.71 (t, *J* = 7.5 Hz, 2H), 2.38 – 2.24 (m, 2H), 2.08 – 1.90 (m, 2H), 1.38 (t, *J* = 7.5 Hz, 3H), 0.94 (t, *J* = 7.5 Hz, 3H); **^{13}C NMR** (75 MHz, $CDCl_3$) δ 166.7, 147.5, 133.1, 129.5, 128.4, 128.0, 127.7, 60.7, 36.1, 34.0, 25.5, 14.3, 13.8; **IR** (neat) 2962, 2932, 1714, 1270, 1176, 1100, 1022, 967, 764, 711 cm^{-1}; **HRMS** (ESI-TOF) *m/z* calcd for $C_{15}H_{21}O_2$ $(M+H)^+$: 233.1542, found 233.1547.

(*E*)-1-(hex-3-en-1-yl)-3,5-dimethoxybenzene (**1i**)

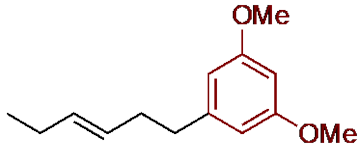

<u>The general procedure B</u> was followed using 1-bromo-3,5-dimethoxybenzene (**1si**, 864 mg, 4.0 mmol) and (*E*)-1-bromohex-3-ene (**1ba**, 1620 mg, 10.0 mmol). Purification of this material by chromatography on silica gel (gradient elution: CH_2Cl_2/hexanes) afforded product **1i** as a colorless oil (616 mg, 70% yield): $\mathbf{R}_f$ = 0.25 (25% CH_2Cl_2/hexanes); **^{1}H NMR** (500 MHz, $CDCl_3$) δ 6.35 (s, 2H), 6.30 (s, 1H), 5.55 – 5.36 (m, 2H), 3.78 (s, 6H), 2.61 (t, *J* = 10.0 Hz, 2H), 2.35 – 2.22 (m, 2H), 2.08 – 1.92 (m, 2H), 0.96 (t, *J* = 7.5 Hz, 3H); **^{13}C NMR** (125 MHz, $CDCl_3$) δ 160.7, 144.6, 132.7, 128.3, 106.5, 97.8, 55.2, 36.5, 34.2, 25.6, 13.9; **IR** (neat) 2962, 2932, 1714, 1270, 1176, 1100, 1022, 967, 764, 711 cm^{-1}; **HRMS** (ESI-TOF) *m/z* calcd for $C_{14}H_{21}O_2$ $(M+H)^+$: 221.1542, found 221.1549.

(*E*)-1-(hex-3-en-1-yl)-4-(trifluoromethyl)benzene (**1j**)

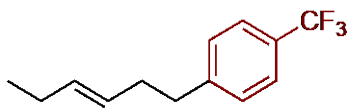

The general procedure B was followed using 1-bromo-4-(trifluoromethyl)benzene (**1sj**, 900 mg, 4.0 mmol) and (*E*)-1-bromohex-3-ene (**1ba**, 1620 mg, 10.0 mmol). Purification of this material by chromatography on silica gel (gradient elution: hexanes) afforded product **1j** as a colorless oil (483 mg, 53% yield): $\mathbf{R}_f$ = 0.50 (hexanes); **^{1}H NMR** (500 MHz, $CDCl_3$) δ 7.53 (d, *J* = 10.0 Hz, 2H), 7.28 (d, *J* = 5.0 Hz, 1H), 5.55 – 5.36 (m, 2H), 2.73 (t, *J* = 10.0 Hz, 2H), 2.38 – 2.26 (m, 2H), 2.08 – 1.92 (m, 2H), 0.96 (t, *J* = 7.5 Hz, 3H); **^{13}C NMR** (125 MHz, $CDCl_3$) δ 146.3, 133.3, 128.8, 128.2 (q, *J* = 32.5 Hz), 127.6, 125.1, 124.4 (q, *J* = 270.00 Hz), 36.0, 34.0, 25.6, 13.8; **^{19}F NMR** (282 MHz, $CDCl_3$) δ 62.7 (s, 3F); **IR** (neat) 2964, 2933, 1619, 1322, 1162, 1119, 1066, 1019, 966, 844, 827 cm^{-1}; **HRMS** (ESI-TOF) *m/z* calcd for $C_{13}H_{15}F_3Ag$ $(M+Ag)^+$: 335.0177, found 335.0219.

(*E*)-1-fluoro-4-(hex-3-en-1-yl)benzene (**1k**)

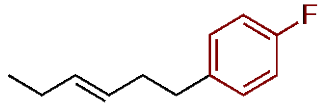

The general procedure B was followed using 1-bromo-4-fluorobenzene (**1sk**, 696 mg, 4.0 mmol) and (*E*)-1-bromohex-3-ene (**1ba**, 1620 mg, 10.0 mmol). Purification of this material by chromatography on silica gel (gradient elution: hexanes/CH_2Cl_2) afforded product **1k** as a colorless oil (292 mg, 41% yield): $\mathbf{R}_f$ = 0.50 (hexanes); **^{1}H NMR** (500 MHz, $CDCl_3$) δ 7.16 – 7.09 (m, 2H), 7.00 – 6.93 (m, 2H), 5.53 – 5.37 (m, 2H), 2.65 (t, *J* = 7.5 Hz, 2H), 2.35 – 2.22 (m, 2H), 2.08 – 1.95 (m, 2H), 0.97 (t, *J* = 7.5 Hz, 3H); **^{13}C NMR** (125 MHz, $CDCl_3$) δ 161.2 (d, *J* = 241.25 Hz), 137.7 (d, *J* = 3.75 Hz), 132.9, 129.7 (d, *J* = 7.50 Hz), 128.0, 114.9 (d, *J* = 21.25 Hz), 35.3, 34.5, 25.6, 13.9; **^{19}F NMR** (282 MHz, $CDCl_3$) δ 118.4 (m, 1F); **IR** (neat) 2962, 2931, 1601, 1509, 1455, 1221, 1157, 966, 850, 823 cm^{-1}; **HRMS** (ESI-TOF) *m/z* calcd for $C_{12}H_{16}F$ $(M+H)^+$: 179.1236, found 179.1222.

(*E*)-1-fluoro-3-(hex-3-en-1-yl)benzene (**1l**)

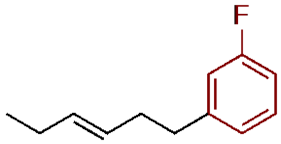

<u>The general procedure B</u> was followed using 1-bromo-3-fluorobenzene (**1sl**, 696 mg, 4.0 mmol) and (*E*)-1-bromohex-3-ene (**1ba**, 1620 mg, 10.0 mmol). Purification of this material by chromatography on silica gel (gradient elution: hexanes) afforded product **1l** as a colorless oil (356 mg, 50% yield): $\mathbf{R}_f$ = 0.50 (hexanes); **^{1}H NMR** (300 MHz, $CDCl_3$) δ 7.26 – 7.16 (m, 1H), 6.98 – 6.82 (m, 3H), 5.55 – 5.34 (m, 2H), 2.66 (t, *J* = 7.5 Hz, 2H), 2.35 – 2.22 (m, 2H), 2.08 – 1.92 (m, 2H), 0.95 (t, *J* = 7.5 Hz, 3H); **^{13}C NMR** (125 MHz, $CDCl_3$) δ 162.9 (d, *J* = 243.75 Hz), 144.8 (d, *J* = 6.25 Hz), 133.0, 129.5 (d, *J* = 8.75 Hz), 127.8, 124.1 (d, *J* = 2.50 Hz), 115.3 (d, *J* = 21.25 Hz), 112.5 (d, *J* = 21.25 Hz), 35.9 (d, *J* = 1.25 Hz), 34.1, 25.5, 13.9; **^{19}F NMR** (282 MHz, $CDCl_3$) δ 114.6 (m, 1F); **IR** (neat) 2962, 2931, 1615, 1589, 1487, 1255, 1140, 966, 864, 779, 690 cm^{-1}; **HRMS** (ESI-TOF) *m/z* calcd for $C_{12}H_{16}F$ $(M+H)^+$: 179.1236, found 179.1241.

(*E*)-2-fluoro-4-(hex-3-en-1-yl)-1-methoxybenzene (**1m**)

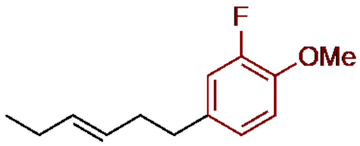

A 50 mL flask was placed in a water bath (rt), charged with magnesium turnings (192 mg, 8.0 mmol), fitted with a rubber septum, and purged with argon. A solution of $FeCl_3$ (194 mg, 1.2 mmol, 30 mol%) in dry THF (20 mL) was added via syringe followed by TMEDA (1.5 mL, 10.0 mmol). The mixture was stirred at rt for 20 min. Then, the reaction was cooled to 0 °C, and 4-bromo-2-fluoro-1-methoxybenzene (**1sm**, 816 mg, 4.0 mmol) and (*E*)-1-bromohex-3-ene (**1ba**, 648 mg, 4.0 mmol) were added. After 12 h at rt, the reaction was quenched with saturated aqueous NH_4Cl (10 mL) and aqueous HCl (10 %, 4 mL) and extracted with ethyl acetate (3 × 20 mL). The combined organic phases were dried (Na_2SO_4), concentrated in vacuo, and subjected to flash chromatography (SiO_2/hexane/CH_2Cl_2) to afford product **1m** as a colorless oil (333 mg, 40% yield): $\mathbf{R}_f$ = 0.50 (25% CH_2Cl_2/hexanes); **^{1}H NMR** (500 MHz, $CDCl_3$) δ 6.95 – 6.85 (m, 3H), 5.55 – 5.34 (m, 2H), 3.87 (s, 3H), 2.60 (t, *J* = 7.5 Hz, 2H), 2.35 – 2.22 (m, 2H), 2.05 – 1.96 (m, 2H), 0.97 (t, *J* = 7.5 Hz, 3H); **^{13}C NMR** (125 MHz, $CDCl_3$) δ 152.2 (d, *J* = 245.00 Hz), 145.6 (d, *J* = 11.25 Hz), 135.4 (d, *J* = 5.00 Hz), 132.9, 127.9, 123.8 (d, *J* = 3.75 Hz), 116.1 (d, *J* = 18.75 Hz), 113.4, 56.4, 35.1, 34.3, 25.5, 13.8; **^{19}F NMR** (282 MHz, $CDCl_3$) δ 136.4 (m, 1F); **IR** (neat)

2961, 2932, 1585, 1515, 1442, 1272, 1222, 1125, 1030, 966, 868, 804, 760 cm^{-1}; **HRMS** (ESI-TOF) *m/z* calcd for $C_{13}H_{18}OF$ $(M+H)^+$: 209.1342, found 209.1344.

(*E*)-1-(hex-3-en-1-yl)naphthalene (**1n**):

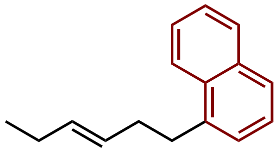

The general procedure B was followed using 1-bromonaphthalene (**1sn**, 828 mg, 4.0 mmol) and (*E*)-1-bromohex-3-ene (**1ba**, 1620 mg, 10.0 mmol). Purification of this material by chromatography on silica gel (gradient elution: hexanes) afforded product **1n** as a colorless oil (442 mg, 53% yield): **R*f*** = 0.50 (hexanes); **^{1}H NMR** (500 MHz, $CDCl_3$) δ 8.06 (d, *J* = 5.0 Hz, 1H), 7.86 (d, *J* = 5.0 Hz, 1H), 7.72 (d, *J* = 5.0 Hz, 1H), 7.60 – 7.28 (m, 4H), 5.64 – 5.42 (m, 2H), 3.14 (t, *J* = 7.5 Hz, 2H), 2.52 – 2.38 (m, 2H), 2.08 – 1.96 (m, 2H), 0.99 (t, *J* = 7.5 Hz, 3H); **^{13}C NMR** (125 MHz, $CDCl_3$) δ 138.3, 133.9, 132.7, 131.9, 128.7, 128.5, 126.5, 125.9, 125.6, 125.5, 125.4, 123.8, 33.7, 33.3, 25.6, 13.9; **IR** (neat) 2960, 2931, 1596, 1510, 1459, 964, 792, 773, 731 cm^{-1}; **HRMS** (ESI-TOF) *m/z* calcd for $C_{16}H_{19}$ $(M+H)^+$: 211.1487, found 211.1486.

(*E*)-2-(hex-3-en-1-yl)naphthalene (**1o**):

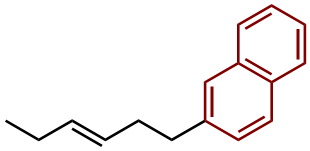

The general procedure B was followed using 2-bromonaphthalene (**1so**, 828 mg, 4.0 mmol) and (*E*)-1-bromohex-3-ene (**1ba**, 1620 mg, 10.0 mmol). Purification of this material by chromatography on silica gel (gradient elution: hexanes) afforded product **1o** as a colorless oil (435 mg, 52% yield): **R*f*** = 0.50 (hexanes); **^{1}H NMR** (500 MHz, $CDCl_3$) δ 7.85 – 7.72 (m, 3H), 7.62 (s, 1H), 7.47 – 7.37 (m, 2H), 7.34 (d, *J* = 5.0 Hz, 1H), 5.55 – 5.40 (m, 2H), 2.84 (t, *J* = 7.5 Hz, 2H), 2.46 – 2.34 (m, 2H), 2.08 – 1.92 (m, 2H), 0.96 (t, *J* = 7.5 Hz, 3H); **^{13}C NMR** (125 MHz, $CDCl_3$) δ 139.7, 133.6, 132.8, 132.0, 128.3, 127.7, 127.6, 127.5, 127.4, 126.4, 125.8, 125.0, 36.3, 34.3, 25.6, 13.9; **IR** (neat) 2960, 2930, 1600, 1508, 1453, 964, 851, 813, 742 cm^{-1}; **HRMS**

(ESI-TOF) *m/z* calcd for $C_{16}H_{19}$ $(M+H)^+$: 211.1487, found 211.1488.

(*E*)-5-(hex-3-en-1-yl)benzo[*d*][1,3]dioxole (**1p**):

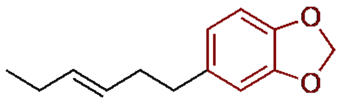

The general procedure A was followed using 5-bromobenzo[*d*][1,3]dioxole (**1sp**, 1600 mg, 8.0 mmol) and (*E*)-1-bromohex-3-ene (**1ba**, 648 mg, 4.0 mmol). Purification of this material by chromatography on silica gel (gradient elution: 2~20% CH_2Cl_2/hexanes) afforded product **1p** as a colorless oil (490 mg, 60% yield): $\mathbf{R_f}$ = 0.20 (25% CH_2Cl_2/hexanes); **^{1}H NMR** (300 MHz, $CDCl_3$) δ 6.92-6.52 (m, 3H), 5.91 (s, 2H), 5.52 – 5.32 (m, 2H), 2.59 (t, *J* = 6.0 Hz, 2H), 2.32 – 2.20 (m, 2H), 2.06 – 1.94 (m, 2H), 0.96 (t, *J* = 6.0 Hz, 3H); **^{13}C NMR** (125 MHz, $CDCl_3$) δ 147.4, 145.5, 136.1, 132.7, 128.2, 121.1, 108.9, 108.0, 100.7, 35.9, 34.7, 25.6, 13.9; **IR** (neat) 2961, 2929, 1502, 1487, 1440, 1242, 1186, 1039, 966, 938, 806, 634 cm^{-1}; **HRMS** (ESI-TOF) *m/z* calcd for $C_{13}H_{17}O_2$ $(M+H)^+$: 205.1229, found 205.1228.

(*E*)-2-fluoro-4-(hex-3-en-1-yl)pyridine (**1q**)

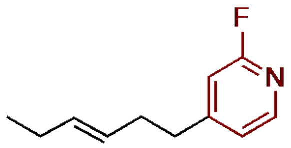

The general procedure B was followed using 4-bromo-2-fluoropyridine (**1sq**, 700 mg, 4.0 mmol) and (*E*)-1-bromohex-3-ene (**1ba**, 1620 mg, 10.0 mmol). Purification of this material by chromatography on silica gel (gradient elution: hexanes/) afforded product **1q** as a colorless oil (308 mg, 43% yield): $\mathbf{R_f}$ = 0.25 (50% CH_2Cl_2/hexanes); **^{1}H NMR** (300 MHz, $CDCl_3$) δ 8.08 (d, *J* = 3.0 Hz, 1H), 7.02-6.90 (m, 1H), 6.73 (s, 1H), 5.52 – 5.30 (m, 2H), 2.70 (t, *J* = 7.5 Hz, 2H), 2.37 – 2.24 (m, 2H), 2.04 – 1.90 (m, 2H), 0.94 (t, *J* = 7.5 Hz, 3H); **^{13}C NMR** (75 MHz, $CDCl_3$) δ 164.0 (d, *J* = 236.25 Hz), 157.0 (d, *J* = 8.25 Hz), 147.1 (d, *J* = 15.75 Hz), 133.8, 126.7, 121.7 (d, *J* = 3.75 Hz), 109.1 (d, *J* = 36.00 Hz), 35.1, 32.8, 25.4, 13.7; **^{19}F NMR** (282 MHz, $CDCl_3$) δ 69.8 (s, 1F); **IR** (neat) 2962, 2933, 1611, 1566, 1409, 1277, 1147, 967, 837, 652, 562 cm^{-1}; **HRMS** (ESI-TOF) *m/z* calcd for $C_{11}H_{15}NF$ $(M+H)^+$: 180.1189, found 180.1193.

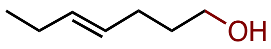

(*E*)-hept-4-en-1-ol

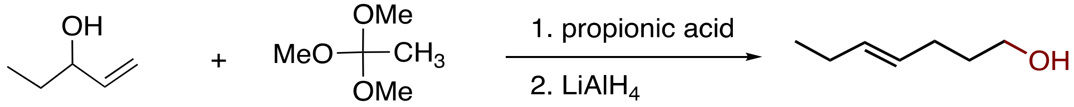

1-Penten-3-ol (2.5 g, 29 mmol, 1.0 equiv), propionic acid (0.1 mL, 1.45 mmol, 0.05 equiv), and trimethyl orthoacetate (10.5 g, 87 mmol, 3.0 equiv) were added to a high-pressure sealed tube. The reaction vessel was then sealed and heated at 120 °C for 12 h. Upon completion, the reaction contents were cooled to 25 °C, the cap was removed, and the reaction contents were reheated to 120 °C for 2 h, open to the atmosphere, to distill off the methanol byproduct. The resultant yellow oil was then dissolved in Et_2O (20 mL) and cannulated dropwise into a suspension of lithium aluminium hydride (1.1 g, 29 mmol, 1.0 equiv) in Et_2O (100 mL) at 0 °C. The resultant slurry was stirred at 0 °C for 60 min and then quenched by careful dropwise addition of saturated aqueous ammonium chloride (20 mL). The resultant biphasic mixture was stirred vigorously for 16 h at 25 °C. The layers were then allowed to separate and the aqueous layer was extracted with additional diethyl ether (2 × 200 mL). The combined organic layers were washed with brine (200 mL), dried over sodium sulphate, and concentrated. The resultant crude oil was purified by flash column chromatography (silica gel, 0-20% ethyl acetate in hexane) to afford (*E*)-hept-4-en-1-ol (2.1g, 64%) as a moderately volatile colorless oil. ^{1}H NMR (500 MHz, $CDCl_3$) δ 5.49 (m, 1 H), 5.41 (m, 1 H), 3.65 (t, *J* = 6.4 Hz, 2 H), 2.12–1.96 (m, 4 H), 1.64 (quintet, *J* = 6.8 Hz, 2 H), 1.40 (br s, 1 H), 0.97 (t, *J* = 7.2 Hz, 3 H); ^{13}C NMR (125 MHz, $CDCl_3$) δ 132.9, 128.6, 62.8, 32.6, 29.0, 25.7, 14.0. Analytical data matches with the literature.[12]

(*E*)-1-(hept-4-en-1-yl)-4-methoxybenzene (**1y**)

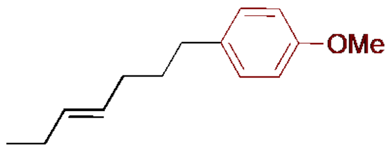

To a solution of (*E*)-hept-4-en-1-ol (1.026 g, 9.0 mmol) in CH_2Cl_2 (9 mL) was added PPh_3 (triphenylphosphine, 2.46 g, 9.36 mmol). NBS (*N*-bromo-succinimide, 1.68 g, 9.36 mmol) was added in batches to the colorless mixture over 15 minutes. The yellow solution was stirred for 24 h at rt. After this time, the solution was passed through a short silica plug to remove any solid

and the filtrate was concentrated to afford a yellow oil. The oil was purified by chromatography on silica gel (gradient elution: hexanes), and concentrated in vacuo carefully to afford product a colorless oil **1bb** (1.188 g, 75% yield): **R*f*** = 0.65 (hexanes). Using **1bb** (704 mg, 4.0 mmol) and 1-bromo-4-methoxybenzene (**1sd**, 1496 mg, 8.0 mmol) by **the general procedure A**. Purification of this material by chromatography on silica gel (gradient elution: 2~20% CH_2Cl_2/hexanes) afforded product **1y** as a colorless oil (449 mg, 55% yield): **R*f*** = 0.50 (25% CH_2Cl_2/hexanes); **^{1}H NMR** (300 MHz, $CDCl_3$) δ 7.13 (d, *J* = 6.0 Hz, 2H), 6.86 (d, *J* = 9.0 Hz, 2H), 5.59 – 5.33 (m, 2H), 3.81 (s, 3H), 2.58 (t, *J* = 9.0 Hz, 2H), 2.10 – 1.90 (m, 4H), 1.80 – 1.53 (m, 2H), 1.01 (t, *J* = 7.5 Hz, 3H); **^{13}C NMR** (75 MHz, $CDCl_3$) δ 157.6, 134.7, 132.4, 129.3, 128.8, 113.6, 55.2, 34.4, 32.0, 31.6, 25.6, 14.0; **IR** (neat) 2960, 2929, 2834, 1612, 1511, 1461, 1242, 1175, 1038, 965, 818, 805 cm^{-1}; **HRMS** (ESI-TOF) *m/z* calcd for $C_{14}H_{21}O$ $(M+H)^+$: 205.1592,found205.1591.

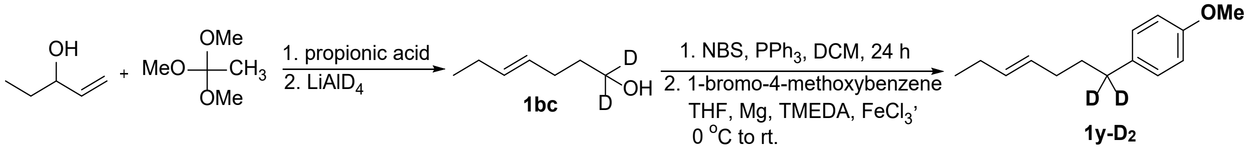

(*E*)-1-(hept-4-en-1-yl-1,1-*d*2)-4-methoxybenzene (**1y-D2**)

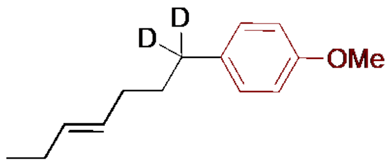

Using the same procedure of synthesis (*E*)-hept-4-en-1-ol to afford a moderately volatile colorless oil **1bc** (lithium aluminium deuteride instead of lithium aluminium hydride). To a solution of **1bc** (1.026 g, 9.0 mmol) in CH_2Cl_2 (9 mL) was added PPh_3 (triphenylphosphine, 2.46 g, 9.36 mmol). NBS (*N*-bromo-succinimide, 1.68 g, 9.36 mmol) was added in batches to the colorless mixture over 15 minutes. The yellow solution was stirred for 24 h at rt. After this time, the solution was passed through a short silica plug to remove any solid and the filtrate was concentrated to afford a yellow oil. The oil was purified by chromatography on silica gel (gradient elution: hexanes), and concentrated in vacuo carefully to afford product a colorless oil **1bd** (1.188 g, 75% yield): **R*f*** = 0.65 (hexanes).

Using this colorless oil (**1bd**, 704 mg, 4.0 mmol) and 1-bromo-4-methoxybenzene (**1sd**, 1496 mg, 8.0 mmol) by **the general procedure A**. Purification of this material by chromatography on

silica gel (gradient elution: 2~20% CH_2Cl_2/hexanes) afforded product **1y-D₂** as a colorless oil (449 mg, 55% yield): $\mathbf{R}_f$ = 0.50 (25% CH_2Cl_2/hexanes); **^{1}H NMR** (500 MHz, $CDCl_3$) δ 7.10 (d, *J* = 10.0 Hz, 2H), 6.83 (d, *J* = 10.0 Hz, 2H), 5.59 – 5.33 (m, 2H), 3.79 (s, 3H), 2.10 – 1.90 (m, 4H), 1.75 – 1.53 (m, 2H), 0.98 (t, *J* = 7.5 Hz, 3H); **HRMS** (ESI-TOF) *m/z* calcd for $C_{14}H_{19}D_2O$ $(M+H)^+$: 207.1718, found 207.1719.

(*Z*)-1-methoxy-4-(oct-5-en-1-yl)benzene (**1z**)

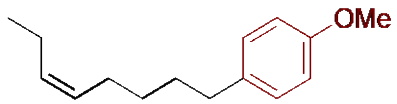

To a solution of (*Z*)-oct-5-en-1-ol (1.152 g, 9.0 mmol) in CH_2Cl_2 (9 mL) was added PPh_3 (triphenylphosphine, 2.46 g, 9.36 mmol). NBS (*N*-bromo-succinimide, 1.68 g, 9.36 mmol) was added in batches to the colorless mixture over 15 minutes. The yellow solution was stirred for 24 h at rt. After this time, the solution was passed through a short silica plug to remove any solid and the filtrate was concentrated to afford a yellow oil. The oil was purified by chromatography on silica gel (gradient elution: hexanes), and concentrated in vacuo carefully to afford product **1be** as a colorless oil (1.231 g, 72% yield): $\mathbf{R}_f$ = 0.65 (hexanes). **The general procedure A** was followed using 1-bromo-4-methoxybenzene (**1sz**, 1496 mg, 8.0 mmol) and **1be** (760 mg, 4.0 mmol). Purification of this material by chromatography on silica gel (gradient elution: 2~20% CH_2Cl_2/hexanes) afforded product **1z** as a colorless oil (523 mg, 60% yield): $\mathbf{R}_f$ = 0.50 (25% CH_2Cl_2/hexanes); **^{1}H NMR** (500 MHz, $CDCl_3$) δ 7.10 (d, *J* = 10.0 Hz, 2H), 6.83 (d, *J* = 10.0 Hz, 2H), 5.45 – 5.25 (m, 2H), 3.79 (s, 3H), 2.56 (t, *J* = 7.5 Hz, 2H), 2.10 – 1.99 (m, 4H), 1.68 – 1.50 (m, 2H), 1.47 – 1.33 (m, 2H), 0.96 (t, *J* = 7.5 Hz, 3H); **^{13}C NMR** (125 MHz, $CDCl_3$) δ 157.6, 134.8, 131.7, 129.2, 129.0, 113.6, 55.2, 34.9, 31.3, 29.3, 26.9, 20.5, 14.4; **IR** (neat) 2929, 2854, 1611, 1511, 1462, 1299, 1243, 1175, 1036, 821, 698 cm^{-1}; **HRMS** (ESI-TOF) *m/z* calcd for $C_{15}H_{23}O$ $(M+H)^+$: 219.1749, found 219.1753.

(*Z*)-1-methoxy-4-(non-6-en-1-yl)benzene (**1aa**)

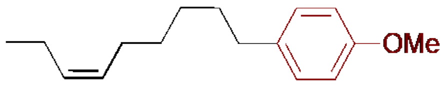

To a solution of (*Z*)-non-6-en-1-ol (1.278 g, 9.0 mmol) in CH_2Cl_2 (9 mL) was added PPh_3 (triphenylphosphine, 2.46 g, 9.36 mmol). NBS (*N*-bromo-succinimide, 1.68 g, 9.36 mmol) was

added in batches to the colorless mixture over 15 minutes. The yellow solution was stirred for 24 h at rt. After this time, the solution was passed through a short silica plug to remove any solid and the filtrate was concentrated to afford a yellow oil. The oil was purified by chromatography on silica gel (gradient elution: hexanes), and concentrated in vacuo carefully to afford product **1bf** as a colorless oil (1.377 g, 75% yield): **R*f*** = 0.65 (hexanes). **The general procedure A** was followed using 1-bromo-4-methoxybenzene (**1saa**, 1496 mg, 8.0 mmol) and **1bf** (816 mg, 4.0 mmol). Purification of this material by chromatography on silica gel (gradient elution: 2~20% CH_2Cl_2/hexanes) afforded product **1aa** as a colorless oil (575 mg, 62% yield): **R*f*** = 0.50 (25% CH_2Cl_2/hexanes); **^{1}H NMR** (300 MHz, $CDCl_3$) δ 7.13 (d, *J* = 9.0 Hz, 2H), 6.86 (d, *J* = 9.0 Hz, 2H), 5.45 – 5.28 (m, 2H), 3.81 (s, 3H), 2.58 (t, *J* = 7.5 Hz, 2H), 2.15 – 1.95 (m, 4H), 1.72 – 1.50 (m, 2H), 1.49 – 1.25 (m, 4H), 1.00 (t, *J* = 7.5 Hz, 3H); **^{13}C NMR** (75 MHz, $CDCl_3$) δ 157.5, 134.9, 131.6, 129.2, 129.1, 113.6, 55.2, 35.0, 31.7, 29.6, 28.9, 27.0, 20.5, 14.4; **IR** (neat) 2928, 2854, 1612, 1511, 1462, 1243, 1175, 1037, 826, cm^{-1}; **HRMS** (ESI-TOF) *m/z* calcd for $C_{16}H_{25}O$ $(M+H)^+$: 233.1905, found 233.1912.

(*E*)-1-(hex-3-en-1-yl)-3-methylbenzene (**1bb**)

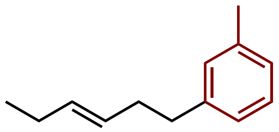

The general procedure A was followed using 1-bromo-3-methylbenzene (**1sbb**, 1368 mg, 8.0 mmol) and (*E*)-1-bromohex-3-ene (**1ba**, 648 mg, 4.0 mmol). Purification of this material by chromatography on silica gel (gradient elution: hexanes) afforded product **1bb** as a colorless oil (418 mg, 60% yield): **R*f*** = 0.60 (hexanes); **^{1}H NMR** (300 MHz, $CDCl_3$) δ 7.24 – 7.15 (m, 1H), 7.10 –6.95 (m, 3H), 5.56 – 5.35 (m, 2H), 2.69 (t, *J* = 7.5 Hz, 2H), 2.45 – 2.25 (m, 5H), 2.15 – 1.95 (m, 2H), 1.02 (t, *J* = 7.5 Hz, 3H); **^{13}C NMR** (75 MHz, $CDCl_3$) δ 142.1, 137.7, 132.5, 129.3, 128.4, 128.1, 126.4, 125.4, 36.1, 34.5, 25.6, 21.4, 13.9; **IR** (neat) 2928, 2869, 1661, 1614, 1454, 1348, 1300, 1248, 1104, 965,774, 694 cm^{-1}; **HRMS** (ESI-TOF) *m/z* calcd for $C_{13}H_{19}$ $(M+H)^+$: 175.1487, found 175.1495.

(*E*)-1-(hex-3-en-1-yl)-3,5-dimethylbenzene (**1cc**)

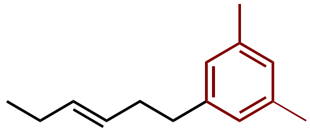

The general procedure A was followed using 1-bromo-3,5-dimethylbenzene (**1scc**, 1368 mg, 8.0 mmol) and (*E*)-1-bromohex-3-ene (**1ba**, 648 mg, 4.0 mmol). Purification of this material by chromatography on silica gel (gradient elution: hexanes) afforded product **1cc** as a colorless oil (452 mg, 60% yield): $\mathbf{R}_f$ = 0.60 (hexanes); **^{1}H NMR** (500 MHz, $CDCl_3$) δ 6.95 – 6.75 (m, 3H), 5.56 – 5.35 (m, 2H), 2.62 (t, *J* = 7.5 Hz, 2H), 2.45 – 2.25 (m, 8H), 2.15 – 1.95 (m, 2H), 1.01 (t, *J* = 7.5 Hz, 3H); **^{13}C NMR** (125 MHz, $CDCl_3$) δ 142.2, 137.6, 132.4, 128.6, 127.3, 126.3, 36.0, 34.6, 25.6, 21.3, 13.9; **IR** (neat) 2929, 2866, 1662, 1604, 1455, 1300, 1033, 966, 848, 693 cm^{-1}; **HRMS** (ESI-TOF) *m/z* calcd for $C_{14}H_{21}$ $(M+H)^+$: 189.1643, found 189.1649.

(*E*)-4-(hex-3-en-1-yl)-1,1'-biphenyl (**1dd**)

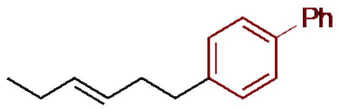

The general procedure A was followed using 4-bromo-1,1'-biphenyl (**1sdd**, 1856 mg, 8.0 mmol) and (*E*)-1-bromohex-3-ene (**1ba**, 648 mg, 4.0 mmol). Purification of this material by chromatography on silica gel (gradient elution: hexanes) afforded product **1bb** as a colorless oil (472 mg, 50% yield): $\mathbf{R}_f$ = 0.50 (hexanes); **^{1}H NMR** (500 MHz, $CDCl_3$) δ 7.61 (d, *J* = 10.0 Hz, 2H), 7.54 (d, *J* = 10.0 Hz, 2H), 7.45 (t, *J* = 10.0 Hz, 2H), 7.35 (t, *J* = 7.5 Hz, 1H), 7.28 (d, *J* = 10.0 Hz, 2H), 5.75 – 5.35 (m, 2H), 2.74 (t, *J* = 7.5 Hz, 2H), 2.45 – 2.25 (m, 2H), 2.15 – 1.95 (m, 2H), 1.00 (t, *J* = 7.5 Hz, 3H); **^{13}C NMR** (75 MHz, $CDCl_3$) δ 141.3, 141.1, 138.6, 132.7, 128.9, 128.7, 128.3, 127.0, 126.9, 35.8, 34.4, 25.6, 13.9; **IR** (neat) 3027, 2930, 2859, 1662, 1486, 1456, 1302, 763, 698 cm^{-1}; **HRMS** (ESI-TOF) *m/z* calcd for $C_{18}H_{21}$ $(M+H)^+$: 237.1643, found 237.1628.

Enantioselective Relay Heck Reaction (General Procedure C):

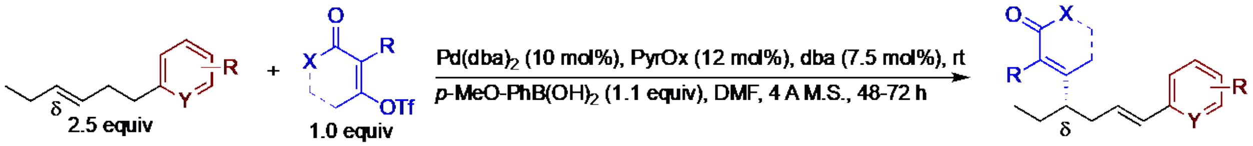

To a 10 mL Schlenk flask was added Pd(dba)$_2$ (11.5 mg, 0.02 mmol, 0.10 equiv), PyrOx (6.5 mg, 0.024 mmol, 0.12 equiv), 4 Å MS (100 mg, 500 mg/1.0 mmol **2**), and DMF (1.0 mL). A three-way adapter fitted with a balloon of N_2 was then added to the Schlenk flask and evacuated via house vacuum and refilled with N_2 three times while stirring. The resulting mixture was stirred under an N_2 atmosphere for 20 min. A solution of alkene **1** (0.50 mmol, 2.5 equiv), dba (3.5 mg, 0.015 mmol, 0.075 equiv), (4-methoxyphenyl)boronic acid (34 mg, 0.22 mmol, 1.1 equiv) and triflate reagent **2** (0.20 mmol, 1.0 equiv) in DMF (1.0 mL) was then added in a single portion. The resulting mixture was stirred for another 48 h to 72 h at ambient temperature (23 °C). The reaction progress was monitored by TLC, and upon complete consumption of triflate reagent **2** the reaction was extracted with Et_2O (3X20 mL) and washed with $NaHCO_3$ (20 mL). The combined solvent was dried over Na_2SO_4, and the filtrate was concentrated to afford a yellow oil. The oil was purified by chromatography on silica gel (first using 1:2 hexanes:CH_2Cl_2 to remove dba ~ yellow on the gel, then gradient elution: 5□10% Acetone/hexanes), and concentrated in vacuo carefully to afford product **3** as a colorless oil.

Product Purification/Characterization Data

(*S,E*)-3-(6-(2-hydroxyphenyl)hex-5-en-3-yl)-2-methylcyclohex-2-en-1-one (**3a**):

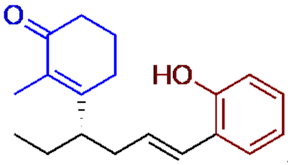

3a

The general procedure C was followed using (*E*)-4-(hex-3-en-1-yl)phenol (**1a**, 88 mg, 0.50 mmol) and 2-methyl-3-oxocyclohex-1-en-1-yl trifluoromethanesulfonate (**2a**, 52 mg, 0.20 mmol) to afford product **3a** as a colorless oil (39.2 mg, 69% yield): $\mathbf{R_f}$ = 0.25 (25% Acetone/hexanes); **^{1}H NMR** (500 MHz, $CDCl_3$) δ 7.22 (d, *J* = 10.0 Hz, 1H), 7.08 (t, *J* = 7.5 Hz, 1H), 6.86 (t, *J* = 7.5 Hz, 1H), 6.75 (d, *J* = 10.0 Hz, 1H), 6.57 (d, *J* = 20.0 Hz, 1H), 6.18-5.90 (m, 1H), 5.02 (br, 1H), 2.90 (br, 1H), 2.50-2.10 (m, 6H), 2.00-1.85 (m, 2H), 1.82 (s, 3H), 1.70-1.30 (m, 2H), 0.86 (t, *J* =

7.5 Hz, 3H); **^{13}C NMR** (75 MHz, $CDCl_3$) δ 199.9, 161.0, 152.6, 132.4, 129.7, 128.2, 127.2, 125.9, 124.6, 120.8, 115.7, 44.9, 38.1, 37.1, 25.9, 24.9, 22.5, 12.1, 10.9; **IR** (neat) 3310, 2957, 2929, 2870, 1639, 1602, 1454, 1365, 1300, 1244, 1191, 1093, 1039, 977, 751 cm^{-1}; **HRMS** (ESI-TOF) *m/z* calcd for $C_{19}H_{25}O_2$ $(M+H)^+$: 285.1855, found 285.1859; $[\alpha]_D^{20}$ = -6° (c = 0.2, $CHCl_3$).

(*S,E*)-2-methyl-3-(6-phenylhex-5-en-3-yl)cyclohex-2-en-1-one (**3b**):

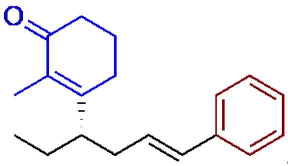

3b

<u>**The general procedure C**</u> was followed using (*E*)-4-(hex-3-en-1-yl)phenol (**1b**, 80 mg, 0.50 mmol) and 2-methyl-3-oxocyclohex-1-en-1-yl trifluoromethanesulfonate (**2a**, 52 mg, 0.20 mmol) to afford product **3a** as a colorless oil (34.3 mg, 64% yield): **R*f*** = 0.35 (25% Acetone/hexanes); **^{1}H NMR** (300 MHz, $CDCl_3$) δ 7.30-7.10 (m, 5H), 6.37 (d, *J* = 18.0 Hz, 1H), 6.05 (m, 1H), 3.00-2.75 (m, 1H), 2.50-2.10 (m, 6H), 2.00-1.85 (m, 2H), 1.81 (s, 3H), 1.70-1.30 (m, 2H), 0.85 (t, *J* = 7.5 Hz, 3H); **^{13}C NMR** (125 MHz, $CDCl_3$) δ 199.4, 160.4, 137.4, 132.4, 131.4, 128.5, 128.0, 127.1, 126.0, 44.9, 38.2, 36.8, 25.8, 25.0, 22.6, 12.1, 10.8; **IR** (neat) 2928, 2869, 1661, 1616, 1454, 1379, 1301, 1190, 1104, 964, 743, 693 cm^{-1}; **HRMS** (ESI-TOF) *m/z* calcd for $C_{19}H_{25}O$ $(M+H)^+$: 269.1905, found 269.1909; $[\alpha]_D^{20}$ = -8° (c = 0.1, $CHCl_3$).

(*S,E*)-2-methyl-3-(6-(p-tolyl)hex-5-en-3-yl)cyclohex-2-en-1-one (**3c**):

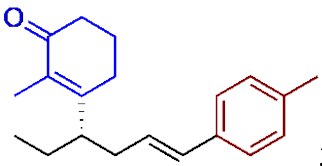

3c

<u>**The general procedure C**</u> was followed using (*E*)-1-(hex-3-en-1-yl)-4-methylbenzene (**1c**, 87 mg, 0.50 mmol) and 2-methyl-3-oxocyclohex-1-en-1-yl trifluoromethanesulfonate (**2a**, 52 mg, 0.20 mmol) to afford product **3c** as a colorless oil (31.6 mg, 56% yield): **R*f*** = 0.35 (25% Acetone/hexanes); **^{1}H NMR** (500 MHz, $CDCl_3$) δ 7.17 (d, *J* = 10.0 Hz, 2H), 7.09 (d, *J* = 10.0 Hz, 2H), 6.34 (d, *J* = 15.0 Hz, 1H), 6.10-5.90 (m, 1H), 3.00-2.80 (m, 1H), 2.50-2.20 (m, 9H), 2.00-

1.85 (m, 2H), 1.81 (s, 3H), 1.70-1.30 (m, 2H), 0.85 (t, J = 7.5 Hz, 3H); ^{13}**C NMR** (125 MHz, $CDCl_3$) δ 199.4, 160.4, 136.9, 134.6, 132.3, 131.2, 129.2, 126.9, 125.9, 44.9, 38.2, 36.8, 25.8, 25.0, 22.6, 21.1, 12.1, 10.8; **IR** (neat) 2929, 2869, 1660, 1616, 1512, 1454, 1378, 1300, 1189, 1105, 1039, 965, 812, 794 cm^{-1}; **HRMS** (ESI-TOF) *m/z* calcd for $C_{20}H_{27}O$ $(M+H)^+$: 283.2062, found 283.2063; $[\alpha]_D^{20}$ = +2.1° (c = 0.4, $CHCl_3$).

(*S,E*)-3-(6-(4-methoxyphenyl)hex-5-en-3-yl)-2-methylcyclohex-2-en-1-one (**3d**):

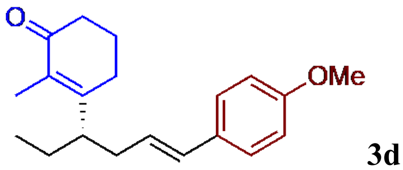

3d

The general procedure C was followed using (*E*)-1-(hex-3-en-1-yl)-4-methoxylbenzene (**1d**, 95 mg, 0.50 mmol) and 2-methyl-3-oxocyclohex-1-en-1-yl trifluoromethanesulfonate (**2a**, 52 mg, 0.20 mmol) to afford product **3d** as a colorless oil (37.0 mg, 62% yield): $\mathbf{R}_f$ = 0.30 (25% Acetone/hexanes); ^{1}**H NMR** (500 MHz, $CDCl_3$) δ 7.21 (d, J = 10.0 Hz, 2H), 6.82 (d, J = 10.0 Hz, 2H), 6.32 (d, J = 15.0 Hz, 1H), 5.90 (dt, J = 15.0 Hz, J = 7.5 Hz,1H), 3.79 (s, 3H), 2.95-2.80 (m, 1H), 2.50-2.20 (m, 6H), 2.00-1.85 (m, 2H), 1.81 (s, 3H), 1.70-1.30 (m, 2H), 0.85 (t, J = 7.5 Hz, 3H); ^{13}**C NMR** (125 MHz, $CDCl_3$) δ 199.5, 160.6, 158.9, 132.3, 130.7, 130.2, 127.0, 125.8, 114.0, 55.3, 45.0, 38.2, 36.8, 25.8, 24.9, 22.6, 12.1, 10.8; **IR** (neat) 2930, 2870, 1659, 1607, 1504, 1456, 1364, 1299, 1174, 1105, 1033, 965, 840, 821 cm^{-1}; **HRMS** (ESI-TOF) *m/z* calcd for $C_{20}H_{27}O_2$ $(M+H)^+$: 299.2011, found 299.2002; $[\alpha]_D^{20}$ = +5.5° (c = 0.4, $CHCl_3$).

(*S,E*)-3-(6-(2-methoxyphenyl)hex-5-en-3-yl)-2-methylcyclohex-2-en-1-one (**3e**):

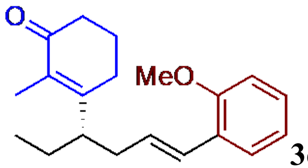

3e

The general procedure C was followed using (*E*)-1-(hex-3-en-1-yl)-2-methoxybenzene (**1e**, 95 mg, 0.50 mmol) and 2-methyl-3-oxocyclohex-1-en-1-yl trifluoromethanesulfonate (**2a**, 52 mg, 0.20 mmol) to afford product **3e** as a colorless oil (28.0 mg, 47% yield): $\mathbf{R}_f$ = 0.30 (25%

Acetone/hexanes); **^{1}H NMR** (500 MHz, $CDCl_3$) δ 7.30-7.26 (m, 1H), 7.20-7.08 (m, 1H), 6.89 (t, *J* = 7.5 Hz, 1H), 6.84 (d, *J* = 5.0 Hz, 1H), 6.68 (d, *J* = 15.0 Hz, 1H), 6.03 (dt, *J* = 15.0 Hz, *J* = 7.5 Hz, 1H), 3.81 (s, 3H), 3.00-2.80 (m, 1H), 2.50-2.10 (m, 6H), 2.00-1.85 (m, 2H), 1.82 (s, 3H), 1.70-1.30 (m, 2H), 0.86 (t, *J* = 7.5 Hz, 3H); **^{13}C NMR** (125 MHz, $CDCl_3$) δ 199.5, 160.6, 156.4, 132.4, 128.6, 128.1, 126.6, 126.5, 126.1, 120.7, 110.8, 55.4, 44.9, 38.2, 37.1, 25.9, 25.0, 22.6, 12.1, 10.8; **IR** (neat) 2930, 2870, 1660, 1616, 1488, 1326, 1242, 1177, 1103, 1029, 971, 750 cm^{-1}; **HRMS** (ESI-TOF) *m/z* calcd for $C_{20}H_{27}O_2$ $(M+H)^+$: 299.2011, found 299.2007; $[\alpha]_D^{20}$ = -10.5° (c = 0.5, $CHCl_3$).

(*S,E*)-3-(6-(4-hydroxyphenyl)hex-5-en-3-yl)-2-methylcyclohex-2-en-1-one (**3f**):

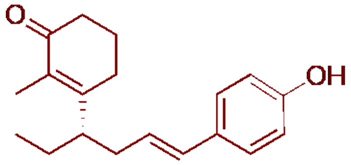

3f

<u>The general procedure C</u> was followed using (*E*)-4-(hex-3-en-1-yl)phenol (**1f**, 88 mg, 0.50 mmol) and 2-methyl-3-oxocyclohex-1-en-1-yl trifluoromethanesulfonate (**2a**, 52 mg, 0.20 mmol) to afford product **3f** as a colorless oil (23.8 mg, 42% yield): $\mathbf{R}_f$ = 0.15 (25% Acetone/hexanes); **^{1}H NMR** (500 MHz, $CDCl_3$) δ 7.15 (d, *J* = 10.0 Hz, 2H), 6.77 (d, *J* = 10.0 Hz, 2H), 6.30 (d, *J* = 15.0 Hz, 1H), 5.95-5.75 (m, 1H), 5.69 (br, 1H), 2.95-2.75 (m, 1H), 2.50-2.20 (m, 6H), 2.00-1.85 (m, 2H), 1.82 (s, 3H), 1.70-1.30 (m, 2H), 0.85 (t, *J* = 5.0 Hz, 3H); **^{13}C NMR** (125 MHz, $CDCl_3$) δ 200.0, 161.3, 155.2, 132.3, 130.8, 130.1, 127.2, 125.6, 115.5, 45.0, 38.1, 36.7, 25.8, 25.0, 22.6, 12.1, 10.8; **IR** (neat) 2927, 2869, 1660, 1608, 1510, 1456, 1326, 1299, 1246, 1174, 1035, 965, 840, 822 cm^{-1}; **HRMS** (ESI-TOF) *m/z* calcd for $C_{19}H_{25}O_2$ $(M+H)^+$: 285.1855, found 285.1859; $[\alpha]_D^{20}$ = +2° (c = 0.2, $CHCl_3$).

(*S,E*)-3-(6-(4-(dimethylamino)phenyl)hex-5-en-3-yl)-2-methylcyclohex-2-en-1-one (**3g**):

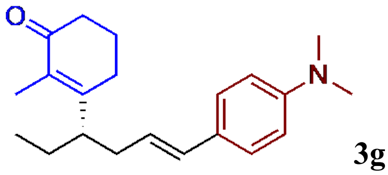

3g

The general procedure C was followed using (*E*)-4-(hex-3-en-1-yl)-*N,N*-dimethylaniline (**1g**, 102 mg, 0.50 mmol) and 2-methyl-3-oxocyclohex-1-en-1-yl trifluoromethanesulfonate (**2a**, 52 mg, 0.20 mmol) without adding (4-methoxyphenyl)boronic acid to afford product **3g** as a colorless oil (28.0 mg, 44% yield): **R*f*** = 0.30 (25% Acetone/hexanes); **^{1}H NMR** (500 MHz, $CDCl_3$) δ 7.17 (d, *J* = 10.0 Hz, 2H), 6.65 (d, *J* = 5.0 Hz, 2H), 6.28 (d, *J* = 15.0 Hz, 1H), 5.84 (dt, *J* = 15.0 Hz, *J* = 7.5 Hz, 1H), 3.00-2.80 (m, 7H), 2.45-2.15 (m, 6H), 1.95-1.85 (m, 2H), 1.81 (s, 3H), 1.70-1.30 (m, 2H), 0.85 (t, *J* = 7.5 Hz, 3H); **^{13}C NMR** (125 MHz, $CDCl_3$) δ 199.5, 160.8, 149.9, 132.2, 131.1, 126.8, 126.1, 123.7, 112.6, 45.2, 40.6, 38.2, 36.8, 25.7, 25.0, 22.6, 12.1, 10.8; **IR** (neat) 2931, 2870, 1661, 1610, 1521, 1445, 1350, 1326, 1187, 1166, 963, 947, 815 cm^{-1}; **HRMS** (ESI-TOF) *m/z* calcd for $C_{21}H_{30}NO$ $(M+H)^+$: 312.2327, found 312.2329; $[\alpha]_D^{20}$ = +13.5° (c = 0.4, $CHCl_3$).

(*S,E*)-2-methyl-3-(6-(p-tolyl)hex-5-en-3-yl)cyclohex-2-en-1-one (**3h**):

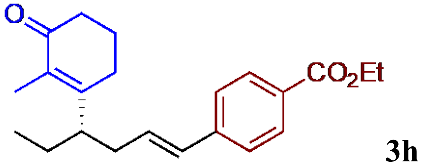

3h

The general procedure C was followed using ethyl (*E*)-4-(hex-3-en-1-yl)benzoate (**1h**, 116 mg, 0.50 mmol) and 2-methyl-3-oxocyclohex-1-en-1-yl trifluoromethanesulfonate (**2a**, 52 mg, 0.20 mmol) to afford product **3h** as a colorless oil (36.0 mg, 53% yield): **R*f*** = 0.25 (25% Acetone/hexanes); **^{1}H NMR** (500 MHz, $CDCl_3$) δ 7.95 (d, *J* = 10.0 Hz, 2H), 7.32 (d, *J* = 5.0 Hz, 2H), 6.40 (d, *J* = 15.0 Hz, 1H), 6.25-6.10 (m, 1H), 4.36 (q, *J* = 5.0 Hz, 2H), 2.95-2.80 (m, 1H), 2.50-2.20 (m, 6H), 2.00-1.85 (m, 2H), 1.81 (s, 3H), 1.70-1.42 (m, 2H), 1.38 (t, *J* = 7.5 Hz, 3H), 0.86 (t, *J* = 7.5 Hz, 3H); **^{13}C NMR** (125 MHz, $CDCl_3$) δ 199.3, 166.4, 159.9, 141.7, 132.4, 130.8, 130.7, 129.9, 129.0, 125.8, 60.8, 44.7, 38.1, 36.8, 25.8, 25.0, 22.6, 14.3, 12.1, 10.8; **IR** (neat) 2932, 2870, 1712, 1660, 1606, 1270, 1176, 1100, 1019, 969, 855, 758, 698 cm^{-1}; **HRMS** (ESI-TOF) *m/z* calcd for $C_{22}H_{29}O_3$ $(M+H)^+$: 341.2117, found 341.2119; $[\alpha]_D^{20}$ = +3.5° (c = 0.5, $CHCl_3$).

(S,E)-3-(6-(3,5-dimethoxyphenyl)hex-5-en-3-yl)-2-methylcyclohex-2-en-1-one (**3i**):

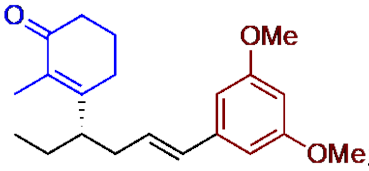

3i

The general procedure C was followed using (E)-1-(hex-3-en-1-yl)-3,5-dimethoxybenzene (**1i**, 87 mg, 0.50 mmol) and 2-methyl-3-oxocyclohex-1-en-1-yl trifluoromethanesulfonate (**2a**, 52 mg, 0.20 mmol) to afford product **3i** as a colorless oil (37.4 mg, 57% yield): $\mathbf{R}_f$ = 0.15 (25% Acetone/hexanes); **^{1}H NMR** (500 MHz, $CDCl_3$) δ 6.43 (s, 2H), 6.33 (s, 1H), 6.30 (d, J = 15.0 Hz, 1H), 6.10-5.95 (m, 1H), 3.78 (s, 6H), 2.95-2.75 (m, 1H), 2.50-2.20 (m, 6H), 2.00-1.85 (m, 2H), 1.81 (s, 3H), 1.70-1.30 (m, 2H), 0.85 (t, J = 5.0 Hz, 3H); **^{13}C NMR** (125 MHz, $CDCl_3$) δ 199.3, 160.9, 160.2, 139.4, 132.3, 131.4, 128.6, 104.2, 99.3, 55.3, 44.8, 38.1, 36.7, 25.7, 25.0, 22.6, 12.1, 10.8; **IR** (neat) 2934, 1710, 1660, 1591, 1456, 1425, 1356, 1219, 1205, 1152, 1064, 966, 826, 757 cm^{-1}; **HRMS** (ESI-TOF) m/z calcd for $C_{21}H_{29}O_3$ $(M+H)^+$: 329.2117, found 329.2121; $[\alpha]_D^{20}$ = +5.8° (c = 0.5, $CHCl_3$).

(S,E)-2-methyl-3-(6-(p-tolyl)hex-5-en-3-yl)cyclohex-2-en-1-one (**3j**):

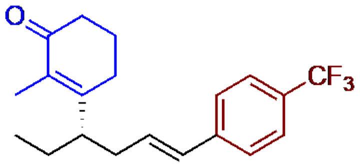

3j

The general procedure C was followed using (E)-1-(hex-3-en-1-yl)-4-(trifluoromethyl)benzene (**1j**, 114 mg, 0.50 mmol) and 2-methyl-3-oxocyclohex-1-en-1-yl trifluoromethanesulfonate (**2a**, 52 mg, 0.20 mmol) to afford product **3j** as a colorless oil (31.6 mg, 47% yield): $\mathbf{R}_f$ = 0.35 (25% Acetone/hexanes); **^{1}H NMR** (500 MHz, $CDCl_3$) δ 7.52 (d, J = 5.0 Hz, 2H), 7.36 (d, J = 10.0 Hz, 2H), 6.40 (d, J = 20.0 Hz, 1H), 6.25-6.05 (m, 1H), 2.95-2.80 (m, 1H), 2.50-2.20 (m, 6H), 2.00-1.85 (m, 2H), 1.81 (s, 3H), 1.70-1.35 (m, 2H), 0.86 (t, J = 7.5 Hz, 3H); **^{13}C NMR** (125 MHz, $CDCl_3$) δ 199.3, 159.9, 140.8, 132.5, 130.9, 130.2, 129.0 (q, J = 31.25 Hz), 126.1, 125.5 (q, J = 3.75 Hz), 124.2 (q, J = 271.25 Hz), 44.7, 38.1, 36.8, 25.8, 24.9, 22.6, 12.1, 10.8; **^{19}F NMR** (282 MHz, $CDCl_3$) δ 62.9 (s, 3F); **IR** (neat) 2931, 1643, 1613, 1515, 1453, 1323, 1265, 1222, 1162, 1066, 967, 825 cm^{-1}; **HRMS** (ESI-TOF) m/z calcd for $C_{20}H_{24}OF_3$ $(M+H)^+$: 337.1779, found

337.1784; $[\alpha]_D^{20}$ = -17.0° (c = 1.0, $CHCl_3$).

(*S,E*)-3-(6-(4-fluorophenyl)hex-5-en-3-yl)-2-methylcyclohex-2-en-1-one (**3k**):

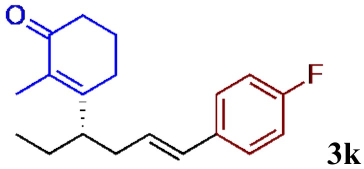

3k

<u>The general procedure C</u> was followed using (*E*)-1-fluoro-4-(hex-3-en-1-yl)benzene (**1k**, 89 mg, 0.50 mmol) and 2-methyl-3-oxocyclohex-1-en-1-yl trifluoromethanesulfonate (**2a**, 52 mg, 0.20 mmol) without adding (4-methoxyphenyl)boronic acid to afford product **3c** as a colorless oil (22.9 mg, 40% yield): **R*f*** = 0.35 (25% Acetone/hexanes); **^{1}H NMR** (500 MHz, $CDCl_3$) δ 7.22 (dd, *J* = 10.0 Hz, 5.0 Hz, 2H), 6.96 (t, *J* = 7.5 Hz, 2H), 6.33 (d, *J* = 20.0 Hz, 1H), 6.05-5.85 (m, 1H), 2.95-2.75 (m, 1H), 2.50-2.20 (m, 6H), 2.00-1.85 (m, 2H), 1.81 (s, 3H), 1.70-1.30 (m, 2H), 0.85 (t, *J* = 7.5 Hz, 3H); **^{13}C NMR** (125 MHz, $CDCl_3$) δ 199.4, 162.1 (d, *J* = 245.0 Hz), 160.2, 133.5 (d, *J* = 2.5 Hz), 132.4, 130.2, 127.8 (d, *J* = 2.5 Hz), 127.4 (d, *J* = 7.5 Hz), 115.4 (d, *J* = 22.5 Hz), 44.9, 38.2, 36.7, 25.8, 25.0, 22.6, 12.1, 10.8; **^{19}F NMR** (282 MHz, $CDCl_3$) δ 115.6 (m, 1F); **IR** (neat) 2930, 2970, 1660, 1616, 1507, 1455, 1344, 1300, 1157, 1094, 965, 812, 844, 821, 767 cm^{-1}; **HRMS** (ESI-TOF) *m/z* calcd for $C_{19}H_{24}OF$ $(M+H)^+$: 287.1811, found 287.1810; $[\alpha]_D^{20}$ = -4° (c = 0.2, $CHCl_3$).

(*S,E*)-3-(6-(3-fluorophenyl)hex-5-en-3-yl)-2-methylcyclohex-2-en-1-one (**3l**):

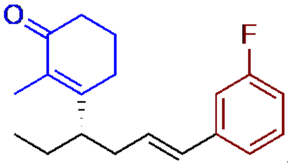

3l

<u>The general procedure C</u> was followed using (*E*)-1-fluoro-3-(hex-3-en-1-yl)benzene (**1l**, 89 mg, 0.50 mmol) and 2-methyl-3-oxocyclohex-1-en-1-yl trifluoromethanesulfonate (**2a**, 52 mg, 0.20 mmol) without adding (4-methoxyphenyl)boronic acid to afford product **3l** as a colorless oil (34.9 mg, 61% yield): **R*f*** = 0.35 (25% Acetone/hexanes); **^{1}H NMR** (500 MHz, $CDCl_3$) δ 7.30-7.15 (m, 1H), 7.10-6.85 (m, 3H), 6.33 (d, *J* = 20.0 Hz, 1H), 6.15-5.85 (m, 1H), 2.95-2.75 (m, 1H),

2.50-2.05 (m, 6H), 2.00-1.82 (m, 2H), 1.81 (s, 3H), 1.70-1.30 (m, 2H), 0.85 (t, *J* = 7.5 Hz, 3H); **^{13}C NMR** (125 MHz, $CDCl_3$) δ 199.4, 163.1 (d, *J* = 243.8 Hz), 160.0, 139.7 (d, *J* = 7.5 Hz), 132.4, 130.4(d, *J* = 2.5 Hz), 130.0 (d, *J* = 8.8 Hz), 129.5, 121.8 (d, *J* = 3.8 Hz), 113.9 (d, *J* = 21.2 Hz), 112.4 (d, *J* = 21.2 Hz), 44.8, 38.1, 36.7, 25.8, 24.9, 22.6, 12.1, 10.8; **^{19}F NMR** (282 MHz, $CDCl_3$) δ 114.0 (m, 1F); **IR** (neat) 2933, 2971, 1660, 1611, 1582, 1445, 1344, 1301, 1142, 965, 874, 778, 684 cm^{-1}; **HRMS** (ESI-TOF) *m/z* calcd for $C_{19}H_{24}OF$ $(M+H)^+$: 287.1811, found 287.1817; $[\alpha]_D^{20}$ = -0.8° (c = 0.4, $CHCl_3$).

(*S,E*)-3-(6-(3-fluoro-4-methoxyphenyl)hex-5-en-3-yl)-2-methylcyclohex-2-en-1-one (**3m**):

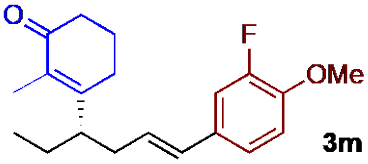

3m

<u>The general procedure C</u> was followed using (*E*)-2-fluoro-4-(hex-3-en-1-yl)-1-methoxybenzene (**1c**, 104 mg, 0.50 mmol) and 2-methyl-3-oxocyclohex-1-en-1-yl trifluoromethanesulfonate (**2a**, 52 mg, 0.20 mmol) to afford product **3m** as a colorless oil (32.2 mg, 51% yield): **R*f*** = 0.30 (25% Acetone/hexanes); **^{1}H NMR** (500 MHz, $CDCl_3$) δ 7.15-6.75 (m, 3H), 6.26 (d, *J* = 20.0 Hz, 1H), 6.00-5.80 (m, 1H), 3.87 (s, 3H), 2.95-2.75 (m, 2H), 2.50-2.15 (m, 6H), 2.00-1.85 (m, 2H), 1.81 (s, 3H), 1.70-1.30 (m, 2H), 0.85 (t, *J* = 7.5 Hz, 3H); **^{13}C NMR** (125 MHz, $CDCl_3$) δ 199.4, 160.2, 152.5 (d, *J* = 243.8 Hz), 146.9, 132.4, 131.1, 129.9 (d, *J* = 1.2 Hz), 127.3, 122.0 (d, *J* = 2.5 Hz), 113.5 (d, *J* = 2.5 Hz), 113.2 (d, *J* = 18.8 Hz), 56.4, 44.9, 38.1, 36.6, 25.8, 25.0, 22.6, 12.1, 10.8; **^{19}F NMR** (282 MHz, $CDCl_3$) δ 135.9 (m, 1F); **IR** (neat) 2933, 2871, 1658 1616, 1514, 1455, 1326, 1300, 1119, 963, 874, 811, 760 cm^{-1}; **HRMS** (ESI-TOF) *m/z* calcd for $C_{20}H_{26}O_2F$ $(M+H)^+$: 317.1917, found 317.1919; $[\alpha]_D^{20}$ = +4.7° (c = 0.5, $CHCl_3$).

(*S,E*)-2-methyl-3-(6-(naphthalen-1-yl)hex-5-en-3-yl)cyclohex-2-en-1-one (**3n**):

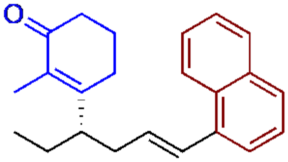

3n

<u>The general procedure C</u> was followed using (*E*)-1-(hex-3-en-1-yl)naphthalene (**1n**, 105 mg, 0.50 mmol) and 2-methyl-3-oxocyclohex-1-en-1-yl trifluoromethanesulfonate (**2a**, 52 mg, 0.20

mmol) to afford product **3n** as a colorless oil (36.2 mg, 57% yield): $\mathbf{R}_f$ = 0.35 (25% Acetone/hexanes); **^{1}H NMR** (500 MHz, $CDCl_3$) δ 8.03 (t, *J* = 5.0 Hz, 1H), 7.84 (d, *J* = 5.0 Hz, 1H), 7.74 (d, *J* = 5.0 Hz, 1H), 7.55-7.35 (m, 4H), 7.10 (d, *J* = 15.0 Hz, 1H), 6.15-6.00 (m, 1H), 3.05-2.85 (m, 1H), 2.65-2.20 (m, 6H), 2.00-1.88 (m, 2H), 1.87 (s, 3H), 1.75-1.45 (m, 2H), 0.90 (t, *J* = 7.5 Hz, 3H); **^{13}C NMR** (125 MHz, $CDCl_3$) δ 199.4, 160.3, 135.3, 133.6, 132.5, 131.4, 131.0, 128.7, 128.5, 127.5, 125.9, 125.7, 125.6, 123.74, 123.69, 44.8, 38.2, 37.1, 26.0, 25.0, 22.6, 12.1, 10.9; **IR** (neat) 2931, 2869, 1658, 1615, 1454, 1378, 1301, 1104, 966, 775, 753 cm^{-1}; **HRMS** (ESI-TOF) *m/z* calcd for $C_{23}H_{27}O$ $(M+H)^+$: 319.2062, found 319.2065; $[\alpha]_D^{20}$ = -24.9° (c = 0.4, $CHCl_3$).

(*S,E*)-2-methyl-3-(6-(naphthalen-2-yl)hex-5-en-3-yl)cyclohex-2-en-1-one (**3o**):

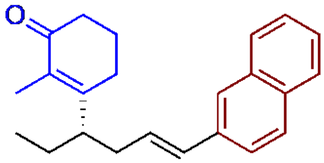

3o

<u>**The general procedure C**</u> was followed using (*E*)-2-(hex-3-en-1-yl)naphthalene (**1o**, 105 mg, 0.50 mmol) and 2-methyl-3-oxocyclohex-1-en-1-yl trifluoromethanesulfonate (**2a**, 52 mg, 0.20 mmol) to afford product **3n** as a colorless oil (28.0 mg, 44% yield): $\mathbf{R}_f$ = 0.35 (25% Acetone/hexanes); **^{1}H NMR** (500 MHz, $CDCl_3$) δ 7.80-7.70 (m, 3H), 7.63 (s, 1H), 7.55-7.35 (m, 3H), 6.54 (d, *J* = 15.0 Hz, 1H), 6.25-6.10 (m, 1H), 3.00-2.80 (m, 1H), 2.50-2.20 (m, 6H), 2.00-1.86 (m, 2H), 1.85 (s, 3H), 1.75-1.45 (m, 2H), 0.88 (t, *J* = 7.5 Hz, 3H); **^{13}C NMR** (125 MHz, $CDCl_3$) δ 199.4, 160.3, 134.8, 133.6, 132.8, 132.4, 131.5, 128.4, 128.2, 127.8, 127.6, 126.2, 125.6, 125.5, 123.4, 44.9, 38.2, 36.9, 25.8, 25.0, 22.6, 12.1, 10.9; **IR** (neat) 2933, 2870, 1661, 1616, 1454, 1379, 1326, 1301, 963, 816, 745 cm^{-1}; **HRMS** (ESI-TOF) *m/z* calcd for $C_{23}H_{27}O$ $(M+H)^+$: 319.2062, found 319.2060; $[\alpha]_D^{20}$ = +2.5° (c = 0.5, $CHCl_3$).

(*S,E*) -3-(6-(benzo[*d*][1,3]dioxol-5-yl)hex-5-en-3-yl)-2-methylcyclohex-2-en-1-one (**3p**):

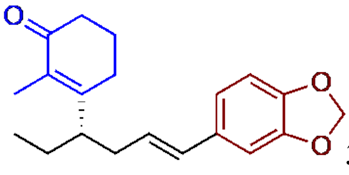
3p

The general procedure C was followed using (*E*)-5-(hex-3-en-1-yl)benzo[*d*][1,3]dioxole (**1p**, 102 mg, 0.50 mmol) and 2-methyl-3-oxocyclohex-1-en-1-yl trifluoromethanesulfonate (**2a**, 52 mg, 0.20 mmol) to afford product **3p** as a colorless oil (26.2 mg, 42% yield): **R*f*** = 0.20 (25% Acetone/hexanes); **^{1}H NMR** (500 MHz, $CDCl_3$) δ 6.81 (s, 1H), 6.75-6.65 (m, 2H), 6.28 (d, *J* = 15.0 Hz, 1H), 6.00-5.80 (m, 3H), 2.90-2.80 (m, 1H), 2.45-2.15 (m, 6H), 2.00-1.82 (m, 2H), 1.81 (s, 3H), 1.70-1.30 (m, 2H), 0.85 (t, *J* = 7.5 Hz, 3H); **^{13}C NMR** (125 MHz, $CDCl_3$) δ 199.4, 160.4, 148.0, 146.8, 132.3, 131.9, 130.9, 126.2, 120.3, 108.2, 105.4, 101.0, 44.9, 38.2, 36.7, 25.8, 25.0, 22.6, 12.1, 10.8; **IR** (neat) 2931, 2871, 1658, 1615, 1502, 1488, 1444, 1358, 1302, 1246, 1190, 1036, 963, 856, 812, 794 cm^{-1}; **HRMS** (ESI-TOF) *m/z* calcd for $C_{20}H_{25}O_3$ $(M+H)^+$: 313.1804, found 313.1805; $[\alpha]_D^{20}$ = +10° (c = 0.2, $CHCl_3$).

(*S,E*)-3-(6-(2-fluoropyridin-4-yl)hex-5-en-3-yl)-2-methylcyclohex-2-en-1-one (**3q**):

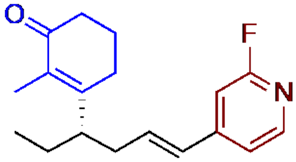
3q

The general procedure C was followed using (*E*)-2-fluoro-4-(hex-3-en-1-yl)pyridine (**1q**, 89.5 mg, 0.50 mmol) and 2-methyl-3-oxocyclohex-1-en-1-yl trifluoromethanesulfonate (**2a**, 52 mg, 0.20 mmol) to afford product **3q** as a colorless oil (37.9 mg, 66% yield): **R*f*** = 0.20 (25% Acetone/hexanes); **^{1}H NMR** (500 MHz, $CDCl_3$) δ 8.09 (d, *J* = 10.0 Hz, 1H), 7.04 (d, *J* = 5.0 Hz, 1H), 6.74 (s, 1H), 6.35-5.30 (m, 2H), 3.00-2.80 (m, 1H), 2.50-2.20 (m, 6H), 2.00-1.85 (m, 2H), 1.80 (s, 3H), 1.70-1.30 (m, 2H), 0.86 (t, *J* = 7.5 Hz, 3H); **^{13}C NMR** (125 MHz, $CDCl_3$) δ 199.2, 164.5 (d, *J* = 236.2 Hz), 159.2, 150.2, 147.7 (d, *J* = 16.2 Hz), 134.8, 132.6, 128.4 (d, *J* = 3.8 Hz), 118.3 (d, *J* = 3.8 Hz), 105.9 (d, *J* = 38.8 Hz), 44.4, 38.1, 36.6, 25.8, 24.9, 22.6, 12.0, 10.8; **^{19}F NMR** (282 MHz, $CDCl_3$) δ 69.2 (s, 1F); **IR** (neat) 2933, 2871, 1657, 1607, 1548, 1405, 1346, 1298, 1154, 1111, 968, 875, 824 cm^{-1}; **HRMS** (ESI-TOF) *m/z* calcd for $C_{18}H_{23}NOF$ $(M+H)^+$: 288.1764, found 288.1768; $[\alpha]_D^{20}$ = -9.0° (c = 0.4, $CHCl_3$).

(*S,E*)-3-(6-(4-methoxyphenyl)hex-5-en-3-yl)-5,5-dimethylcyclohex-2-en-1-one (**3r**)

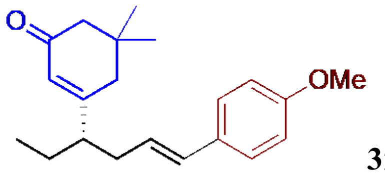

3r

The general procedure C was followed using (*E*)-1-(hex-3-en-1-yl)-4-methoxylbenzene (**1d**, 95 mg, 0.50 mmol) and 5,5-dimethyl-3-oxocyclohex-1-en-1-yl trifluoromethanesulfonate (**2b**, 55 mg, 0.20 mmol) to afford product **3r** as a colorless oil (25.6 mg, 41% yield): $\mathbf{R}_f$ = 0.30 (25% Acetone/hexanes); **^{1}H NMR** (300 MHz, $CDCl_3$) δ 7.26 (d, *J* = 9.0 Hz, 2H), 6.86 (d, *J* = 9.0 Hz, 2H), 6.36 (d, *J* = 15.0 Hz, 1H), 6.03-5.86 (m, 2H), 3.83 (s, 3H), 2.50-2.10 (m, 7H), 1.80-1.40 (m, 2H), 1.06 (s, 6H), 0.90 (t, *J* = 6.0 Hz, 3H); **^{13}C NMR** (75 MHz, $CDCl_3$) δ 200.0, 160.0, 158.9, 131.1, 130.1, 127.1, 125.8, 125.4, 113.9, 55.3, 51.4, 49.6, 41.2, 36.6, 33.5, 28.5, 28.4, 25.5, 11.9; **IR** (neat) 2957, 2928, 2871, 1662, 1607, 1510, 1463, 1368, 1299, 1280, 1174, 1033, 966, 838, 806 cm^{-1}; **HRMS** (ESI-TOF) *m/z* calcd for $C_{21}H_{29}O_2$ $(M+H)^+$: 313.2168, found 313.2169; $[\alpha]_D^{20}$ = +36.6° (c = 0.4, $CHCl_3$).

(*S,E*)-2-bromo-3-(6-(4-methoxyphenyl)hex-5-en-3-yl)cyclohex-2-en-1-one (**3s**)

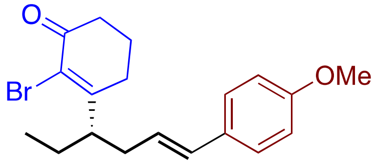

3s

The general procedure C was followed using (*E*)-1-(hex-3-en-1-yl)-4-methoxylbenzene (**1d**, 95 mg, 0.50 mmol) and 2-bromo-3-oxocyclohex-1-en-1-yl trifluoromethanesulfonate (**2c**, 64 mg, 0.20 mmol) to afford product **3s** as a colorless oil (21.7 mg, 30% yield): $\mathbf{R}_f$ = 0.30 (25% Acetone/hexanes); **^{1}H NMR** (300 MHz, $CDCl_3$) δ 7.22 (d, *J* = 9.0 Hz, 2H), 6.82 (d, *J* = 9.0 Hz, 2H), 6.32 (d, *J* = 15.0 Hz, 1H), 6.05-5.80 (m,1H), 3.79 (s, 3H), 3.50-2.75 (m, 1H), 2.65-2.10 (m, 5H), 2.00-1.80 (m, 2H), 1.75-1.30 (m, 3H), 0.91 (t, *J* = 7.5 Hz, 3H); **^{13}C NMR** (75 MHz, $CDCl_3$) δ 191.3, 165.6, 158.9, 131.0, 130.0, 127.2, 125.0, 124.7, 113.9, 55.3, 49.0, 38.4, 36.5, 27.5, 25.6, 22.0, 12.0; **IR** (neat) 2958, 2922, 2854, 1694, 1637, 1510, 1441, 1298, 1244, 1174, 1032, 966,

827, 802 cm^{-1}; **HRMS** (ESI-TOF) *m/z* calcd for $C_{19}H_{24}O_2Br$ $(M+H)^+$: 363.0960, found 363.0957; $[\alpha]_D^{20}$ = -70.5° (c = 0.5, $CHCl_3$).

(*S,E*)-3-(6-(4-methoxyphenyl)hex-5-en-3-yl)-2-methylcyclopent-2-en-1-one (**3t**)

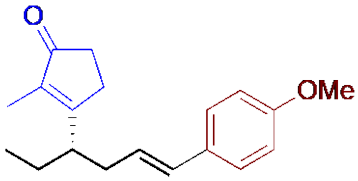

3t

The general procedure C was followed using (*E*)-1-(hex-3-en-1-yl)-4-methoxylbenzene (**1d**, 95 mg, 0.50 mmol) and 2-methyl-3-oxocyclopent-1-en-1-yl trifluoromethanesulfonate (**2d**, 49 mg, 0.20 mmol) to afford product **3t** as a colorless oil (25.6 mg, 45% yield): $\mathbf{R}_f$ = 0.20 (25% Acetone/hexanes); ^{1}**H NMR** (300 MHz, $CDCl_3$) δ 7.22 (d, *J* = 9.0 Hz, 2H), 6.82 (d, *J* = 9.0 Hz, 2H), 6.32 (d, *J* = 15.0 Hz, 1H), 6.00-5.80 (m, 1H), 3.79 (s, 3H), 2.95-2.80 (m, 1H), 2.50-2.10 (m, 6H), 1.80-1.50 (m, 5H), 0.84 (t, *J* = 7.5 Hz, 3H); ^{13}**C NMR** (75 MHz, $CDCl_3$) δ 210.5, 175.8, 158.9, 137.4, 130.9, 130.0, 127.0, 125.4, 113.9, 55.3, 43.0, 36.9, 33.9, 26.1, 25.0, 12.1, 8.4; **IR** (neat) 2958, 2922, 2854, 1694, 1637, 1510, 1441, 1298, 1174, 1032, 966, 827, 802 cm^{-1}; **HRMS** (ESI-TOF) *m/z* calcd for $C_{19}H_{25}O_2$ $(M+H)^+$: 285.1855, found 285.1852; $[\alpha]_D^{20}$ = +28.9° (c = 0.4, $CHCl_3$).

methyl (*S*)-2-(6-(4-methoxyphenyl)hexan-3-yl)cyclopent-1-ene-1-carboxylate (**3u**)

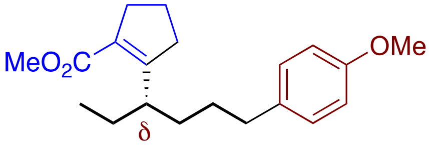

3u

The general procedure C was followed using (*E*)-1-(hex-3-en-1-yl)-4-methoxylbenzene (**1d**, 95 mg, 0.50 mmol) and methyl 2-(((trifluoromethyl)sulfonyl)oxy)cyclopent-1-ene-1-carboxylate (**2e**, 55 mg, 0.20 mmol) to afford a colorless oil. Then 10 mol % of Pd/C was added to this oil with 0.5 mL methanol under a balloon of H_2. After stirring for 1h at rt, the solution was passed through a short silica plug remove any solid to give product **3u** as a colorless oil (32.9 mg, 52% yield, δ:γ = 2.8): $\mathbf{R}_f$ = 0.45 (25% Acetone/hexanes), data bellow belongs to the major product (δ position); ^{1}**H NMR** (300 MHz, $CDCl_3$) δ 7.06 (d, *J* = 9.0 Hz, 2H), 6.82 (d, *J* = 9.0 Hz, 2H), 3.78 (s, 3H), 3.70 (s, 3H), 3.65-3.45 (m, 1H), 2.70-2.25 (m, 6H), 1.90-1.65 (m, 2H), 1.55-1.10 (m, 6H), 0.79 (t, *J* = 7.5 Hz, 3H); ^{13}**C NMR** (75 MHz, $CDCl_3$) δ 166.8, 162.6, 157.5, 134.7, 129.2,

128.8, 113.6, 55.2, 50.8, 39.7, 35.0, 33.6, 33.2, 32.4, 29.6, 26.9, 21.7, 12.1; **IR** (neat) 2928, 2856, 1710, 1630, 1512, 1462, 1434, 1245, 1178, 1120, 1038, 818, 770 cm^{-1}; **HRMS** (ESI-TOF) *m/z* calcd for $C_{20}H_{29}O_3$ $(M+H)^+$: 317.2117, found 317.2120; $[\alpha]_D^{20}$ = -4.8° (c = 0.5, $CHCl_3$).

ethyl (*S,Z*)-4-ethyl-7-(4-methoxyphenyl)-2,3-dimethylhept-2-enoate (**3v**)

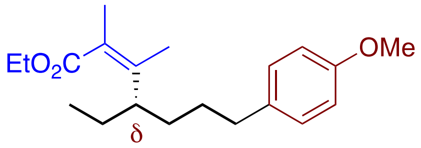

3v

<u>The general procedure C</u> was followed using (*E*)-1-(hex-3-en-1-yl)-4-methoxylbenzene (**1d**, 95 mg, 0.50 mmol) and ethyl (*Z*)-2-methyl-3-(((trifluoromethyl)sulfonyl)oxy)but-2-enoate (**2f**, 55 mg, 0.20 mmol) to afford a colorless oil. Then 10 mol % of Pd/C was added to this oil with 0.5 mL methanol under a balloon of H_2. After stirring for 1h at rt, the solution was passed through a short silica plug remove any solid to give product **3v** as a colorless oil (38.2 mg, 60% yield, δ:γ = 4.3): $\mathbf{R_f}$ = 0.45 (25% Acetone/hexanes), data bellow belongs to the major product (δ position); **^{1}H NMR** (300 MHz, $CDCl_3$) δ 7.07 (d, *J* = 6.0 Hz, 2H), 6.81 (d, *J* = 9.0 Hz, 2H), 4.17 (q, *J* = 7.0 Hz, 2H), 3.78 (s, 3H), 2.95-2.75 (m, 1H), 2.70-2.30 (m, 2H), 1.84 (s, 3H), 1.52 (s, 3H), 1.45-1.15 (m, 9H), 0.77 (t, *J* = 7.5 Hz, 3H); **^{13}C NMR** (75 MHz, $CDCl_3$) δ 170.6, 157.5, 144.5, 134.8, 129.2, 125.0, 113.5, 60.0, 55.2, 44.1, 35.0, 32.7, 29.5, 26.3, 16.0, 14.3, 12.6, 12.0; **IR** (neat) 2957, 2930, 1712, 1612, 1512, 1462, 1380, 1299, 1243, 1175, 1037, 819, 775 cm^{-1}; **HRMS** (ESI-TOF) *m/z* calcd for $C_{20}H_{31}O_3$ $(M+H)^+$: 319.2273, found 319.2275; $[\alpha]_D^{20}$ = -7.6° (c = 0.5, $CHCl_3$).

1-(*tert*-butyl) 4-methyl (*S*)-5-(6-(4-methoxyphenyl)hexan-3-yl)-3,6-dihydropyridine-1,4(2*H*)-dicarboxylate (**3w**)

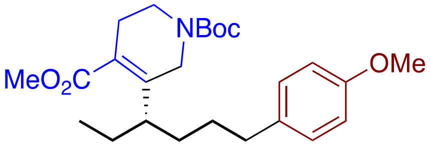

3w

<u>The general procedure C</u> was followed using (*E*)-1-(hex-3-en-1-yl)-4-methoxylbenzene (**1d**, 95 mg, 0.50 mmol) and 1-(*tert*-butyl) 4-methyl 5-(((trifluoromethyl)sulfonyl)oxy)-3,6-dihydropyridine-1,4(2*H*)-dicarboxylate (**2g**, 78 mg, 0.20 mmol) to afford a colorless oil. Then 10 mol % of Pd/C was added to this oil with 0.5 mL methanol under a balloon of H_2. After stirring

for 1h at rt, the solution was passed through a short silica plug remove any solid to give product **3w** as a colorless oil (52.6 mg, 61% yield): **R*f*** = 0.30 (25% Acetone/hexanes); **^{1}H NMR** (300 MHz, $CDCl_3$) δ 7.06 (d, *J* = 9.0 Hz, 2H), 6.80 (d, *J* = 9.0 Hz, 2H), 4.09 (s, 2H), 3.78 (s, 3H), 3.72 (s, 3H), 3.39 (br, 3H), 2.70-2.30 (m, 2H), 2.07 (br, 2H), 1.70-1.15 (m, 15H), 0.79 (t, *J* = 7.5 Hz, 3H); **^{13}C NMR** (75 MHz, $CDCl_3$) δ 167.2, 157.6, 154.6, 150.2, 134.5, 129.2, 129.1, 113.6, 79.8, 55.2, 51.3, 43.9, 42.3, 39.1, 34.9, 32.4, 29.5, 28.4, 26.2, 23.7, 12.0; **IR** (neat) 2929, 2857, 1694, 1512, 1455, 1419, 1295, 1164, 1116, 1037, 952, 830, 768 cm^{-1}; **HRMS** (ESI-TOF) *m/z* calcd for $C_{25}H_{37}NO_5Na$ $(M+Na)^+$: 454.2569, found 454.2576; $[\alpha]_D^{20}$ = -6.0° (c = 0.5, $CHCl_3$).

ethyl (S,2*Z*,6*E*)-2-(3-(1,3-dioxoisoindolin-2-yl)propyl)-4-ethyl-7-(2-fluoropyridin-4-yl)-3-methylhepta-2,6-dienoate (**3x**)

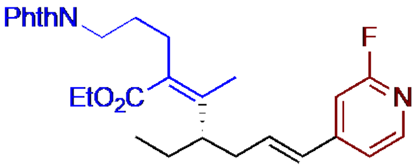

3x

<u>The general procedure C</u> was followed using (*E*)-1-(hex-3-en-1-yl)-4-methoxylbenzene (**1d**, 95 mg, 0.50 mmol) and ethyl (*Z*)-5-(1,3-dioxoisoindolin-2-yl)-2-(1-(((trifluoromethyl)sulfonyl)-oxy)ethylidene)-pentanoate (**2h**, 90 mg, 0.20 mmol) to afford product **3x** as a colorless oil (48.8 mg, 51% yield, using 2% Acetone/toluene for column): **R*f*** = 0.20 (25% Acetone/hexanes); **^{1}H NMR** (500 MHz, $CDCl_3$) δ 8.00 (d, *J* = 5.0 Hz, 1H), 7.82 (t, *J* = 5.0 Hz, 2H), 7.70 (d, *J* = 5.0 Hz, 2H), 7.06 (d, *J* = 5.0 Hz, 1H), 6.75 (s, 1H), 6.50-632 (m, 1H), 6.26 (d, *J* = 20.0 Hz,1H), 4.20-4.10 (m, 2H), 3.63 (t, *J* = 7.5 Hz, 2H), 3.00-2.80 (m, 1H), 2.40-2.20 (m, 4H), 1.85-1.70 (m, 2H), 1.61 (s, 3H), 1.50-1.30 (m, 2H) , 1.24 (t, *J* = 7.5 Hz, 3H), 0.81 (t, *J* = 7.5 Hz, 3H); **^{13}C NMR** (125 MHz, $CDCl_3$) δ 170.0, 168.2, 164.5 (d, *J* = 236.2 Hz), 150.9, 147.4 (d, *J* = 16.2 Hz), 142.6, 136.4, 133.9, 132.1, 130.3, 127.3 (d, *J* = 3.8 Hz), 123.2, 118.4 (d, *J* = 3.8 Hz), 105.9 (d, *J* = 37.5 Hz), 60.3, 44.8, 37.7, 36.9, 27.6, 27.4, 25.8, 14.2, 12.2, 11.9; **^{19}F NMR** (282 MHz, $CDCl_3$) δ 69.7 (s, 1F); **IR** (neat) 2929, 1710, 1609, 1396, 1206, 1088, 1038, 720 cm^{-1}; **HRMS** (ESI-TOF) *m/z* calcd for $C_{28}H_{32}N_2O_4F$ $(M+H)^+$: 479.2346, found 479.2355; $[\alpha]_D^{20}$ = -22.6° (c = 0.4, $CHCl_3$).

(*S*)-3-(7-(4-methoxyphenyl)heptan-3-yl)-2-methylcyclohex-2-en-1-one (**3y**)

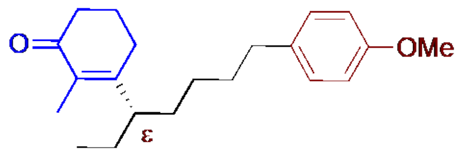

3y

<u>**The general procedure C**</u> was followed using (*E*)-1-(hept-4-en-1-yl)-4-methoxybenzene (**1y**, 102 mg, 0.50 mmol) and 2-methyl-3-oxocyclohex-1-en-1-yl trifluoromethanesulfonate (**2a**, 52 mg, 0.20 mmol) to afford a colorless oil. Then 10 mol % of Pd/C was added to this oil with 0.5 mL methanol under a balloon of H_2. After stirring for 1h at rt, the solution was passed through a short silica plug remove any solid to give product **3y** as a colorless oil (39.3 mg, 63% yield, ε:δ = 2.6): $\mathbf{R}_f$ = 0.30 (25% Acetone/hexanes), data bellow belongs to the major product (ε position); **^{1}H NMR** (300 MHz, $CDCl_3$) δ 7.06 (d, *J* = 5.0 Hz, 2H), 6.81 (d, *J* = 10.0 Hz, 2H), 3.78 (s, 3H), 2.75-2.58 (m, 1H), 2.56-2.28 (m, 4H), 2.25-2.00 (m, 2H), 1.98-1.72 (m, 5H), 1.70-1.05 (m, 8H), 0.80 (t, *J* = 7.5 Hz, 3H); **^{13}C NMR** (75 MHz, $CDCl_3$) δ 199.5, 161.4, 157.6, 134.5, 132.2, 129.2, 113.6, 55.2, 44.4, 38.2, 34.8, 33.1, 31.8, 27.1, 26.4, 24.5, 22.6, 12.1, 10.9; **IR** (neat) 2930, 2856, 1661, 1612, 1512, 1456, 1344, 1300, 1244, 1177, 1036, 818 cm^{-1}; **HRMS** (ESI-TOF) *m/z* calcd for $C_{21}H_{30}O_2Na$ $(M+Na)^+$: 337.2143, found 337.2141; $[\alpha]_D^{20}$ = -0.3° (c = 0.5, $CHCl_3$).

(*S*)-3-(8-(4-methoxyphenyl)octan-3-yl)-2-methylcyclohex-2-en-1-one (**3z**)

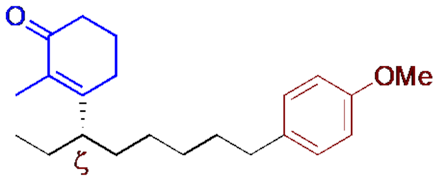

3z

<u>**The general procedure C**</u> was followed using (*Z*)-1-methoxy-4-(oct-5-en-1-yl)benzen (**1z**, 109 mg, 0.50 mmol) and 2-methyl-3-oxocyclohex-1-en-1-yl trifluoromethanesulfonate (**2a**, 52 mg, 0.20 mmol) to afford a colorless oil. Then 10 mol % of Pd/C was added to this oil with 0.5 mL methanol under a balloon of H_2. After stirring for 1h at rt, the solution was passed through a short silica plug remove any solid to give product **3z** as a colorless oil (38.5 mg, 59% yield, ζ:ε = 2.6): $\mathbf{R}_f$ = 0.30 (25% Acetone/hexanes), data bellow belongs to the major product (ζ position); **^{1}H NMR** (500 MHz, $CDCl_3$) δ 7.07 (d, *J* = 10.0 Hz, 2H), 6.82 (d, *J* = 5.0 Hz, 2H), 3.78 (s, 3H), 2.75-2.58 (m, 1H), 2.52 (t, *J* = 7.5 Hz, 2H), 2.40 (t, *J* = 5.0 Hz, 2H), 2.17 (t, *J* = 5.0 Hz,, 2H), 1.98-1.80 (m, 2H), 1.78 (s, 3H), 1.65-1.10 (m, 10H), 0.80 (t, *J* = 7.5 Hz, 3H); **^{13}C NMR** (75

MHz, $CDCl_3$) δ 199.6, 161.5, 157.6, 134.7, 132.2, 129.2, 113.6, 55.2, 44.5, 38.2, 34.9, 33.2, 31.5, 29.4, 27.5, 26.4, 24.5, 22.6, 12.1, 10.8; **IR** (neat) 2928, 2855, 1662, 1612, 1511, 1456, 1344, 1300, 1244, 1176, 1036, 830 cm^{-1}; **HRMS** (ESI-TOF) *m/z* calcd for $C_{22}H_{33}O_2$ $(M+H)^+$: 329.2481, found 329.2488; $[\alpha]_D^{20}$ = +3.5° (c = 0.5, $CHCl_3$).

(*S*)-3-(9-(4-methoxyphenyl)nonan-3-yl)-2-methylcyclohex-2-en-1-one (**3aa**)

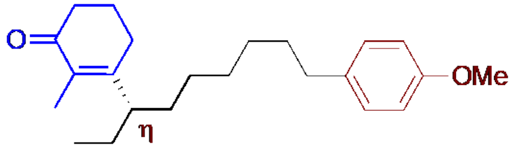

3aa

The general procedure C was followed using (*Z*)-1-methoxy-4-(non-6-en-1-yl)benzene (**1aa**, 116 mg, 0.50 mmol) and 2-methyl-3-oxocyclohex-1-en-1-yl trifluoromethanesulfonate (**2a**, 52 mg, 0.20 mmol) to afford a colorless oil. Then 10 mol % of Pd/C was added to this oil with 0.5 mL methanol under a balloon of H_2. After stirring for 1h at rt, the solution was passed through a short silica plug remove any solid to give product **3aa** as a colorless oil (38.8 mg, 57% yield, η:ζ = 2.0): $\mathbf{R}_f$ = 0.30 (25% Acetone/hexanes), data bellow belongs to the major product (η position); **^{1}H NMR** (300 MHz, $CDCl_3$) δ 7.08 (d, *J* = 9.0 Hz, 2H), 6.82 (d, *J* = 9.0 Hz, 2H), 3.78 (s, 3H), 2.75-2.58 (m, 1H), 2.52 (t, *J* = 6.0 Hz, 2H), 2.40 (t, *J* = 6.0 Hz, 2H), 2.17 (m,, 2H), 1.98-1.80 (m, 2H), 1.79 (s, 3H), 1.65-1.10 (m, 12H), 0.80 (t, *J* = 7.5 Hz, 3H); **^{13}C NMR** (75 MHz, $CDCl_3$) δ 199.6, 161.6, 157.5, 134.8, 132.2, 129.2, 113.6, 55.2, 44.5, 38.2, 34.9, 33.3, 31.7, 29.7, 29.1, 27.6, 26.4, 24.5, 22.6, 12.1, 10.8; **IR** (neat) 2927, 2854, 1661, 1612, 1511, 1456, 1300, 1243, 1176, 1036, 828 cm^{-1}; **HRMS** (ESI-TOF) *m/z* calcd for $C_{23}H_{35}O_2$ $(M+H)^+$: 343.2637, found 343.2638; $[\alpha]_D^{20}$ = +2.5° (c = 0.4, $CHCl_3$).

(*S,E*)-3-(7-(4-methoxyphenyl)hept-6-en-3-yl)-2-methylcyclohex-2-en-1-one (**3y-deuterated)**

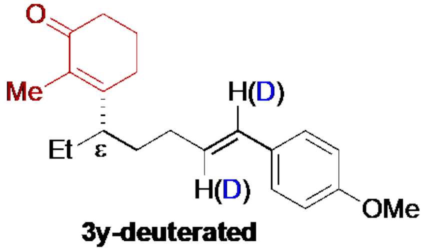

3y-deuterated
47% yield, ε:δ = 2.6:1.0

Product Distrubution

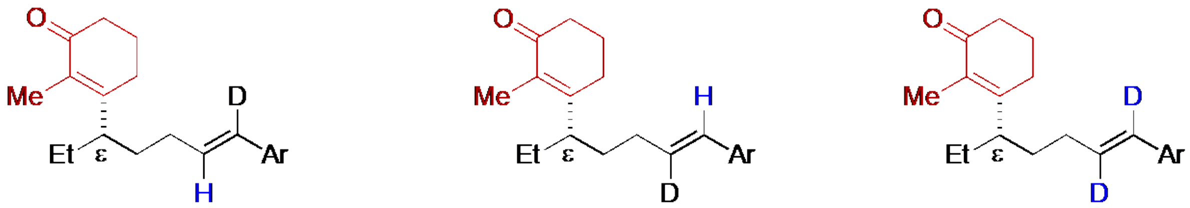

3y(H-D), 23% **3y(D-H)**, 12% **3y(D-D)**, 12%

The general procedure C was followed using (*E*)-1-(hept-4-en-1-yl-1,1-d_2)-4-methoxybenzene (**1y-D$_2$**, 107 mg, 0.50 mmol) and 2-methyl-3-oxocyclohex-1-en-1-yl trifluoromethanesulfonate (**2a**, 52 mg, 0.20 mmol) to afford product (*S,E*)-3-(7-(4-methoxyphenyl)hept-6-en-3-yl)-2-methylcyclohex-2-en-1-oneas a colorless oil (29.7 mg, 47% yield; 63% yield with byproduct **4y**): **R$_f$** = 0.30 (25% Acetone/hexanes); **^{1}H NMR** (500 MHz, $CDCl_3$) δ 7.24 (d, J = 10.0 Hz, 1.48H), 7.13-6.95 (m, 0.56 H), 6.88-6.78 (m, 2H), 6.27 (s, 0.19H, **3y(D-H)**,), 6.01 (t, J = 7.5 Hz, 0.36H, **3y(H-D)**), 5.65-5.05 (m, 0.54H, byproduct **4y**).

(*S,E*)-2-methyl-3-(6-(m-tolyl)hex-5-en-3-yl)cyclohex-2-en-1-one (**3bb**)

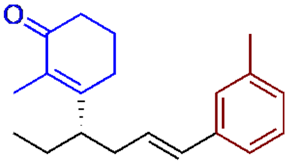

3bb

The general procedure C was followed using (*E*)-1-(hex-3-en-1-yl)-3-methylbenzene (**1bb**, 87 mg, 0.50 mmol) and 2-methyl-3-oxocyclohex-1-en-1-yl trifluoromethanesulfonate (**2a**, 52 mg, 0.20 mmol) to afford product **3bb** as a colorless oil (31.6 mg, 56% yield): **R$_f$** = 0.30 (25% Acetone/hexanes); **^{1}H NMR** (500 MHz, $CDCl_3$) δ 7.25-6.82 (m, 4H), 6.34 (d, J = 15.0 Hz, 1H), 6.12-5.90 (m,1H), 2.95-2.75 (m, 1H), 2.50-2.20 (m, 9H), 2.00-1.84 (m, 2H), 1.82 (s, 3H), 1.80-1.40 (m, 2H), 0.85 (t, J = 7.5 Hz, 3H); **^{13}C NMR** (125 MHz, $CDCl_3$) δ 199.6, 160.5, 138.0, 137.2, 132.3, 131.4, 128.4, 127.9, 127.7, 126.7, 123.1, 44.8, 38.1, 36.8, 25.8, 24.9, 22.6, 21.4, 12.1, 10.8; **IR** (neat) 2928, 2869, 1661, 1614, 1454, 1348, 1300, 1177, 1104, 1038, 965, 774, 694 cm^{-1}; **HRMS** (ESI-TOF) *m/z* calcd for $C_{20}H_{27}O$ $(M+H)^+$: 283.2062, found 283.2070.

(*S,E*)-3-(6-(3,5-dimethylphenyl)hex-5-en-3-yl)-2-methylcyclohex-2-en-1-one (**3cc**)

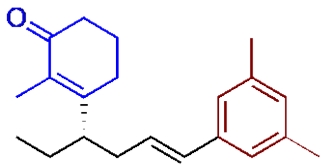

3cc

<u>The general procedure C</u> was followed using (*E*)-1-(hex-3-en-1-yl)-3,5-dimethylbenzene (**1cc**, 94 mg, 0.50 mmol) and 2-methyl-3-oxocyclohex-1-en-1-yl trifluoromethanesulfonate (**2a**, 52 mg, 0.20 mmol) to afford product **3cc** as a colorless oil (35.5 mg, 60% yield): **R_f** = 0.30 (25% Acetone/hexanes); **^{1}H NMR** (300 MHz, $CDCl_3$) δ 6.90 (s, 2H), 6.84 (s, 1H), 6.30 (d, *J* = 15.0 Hz, 1H), 6.12-5.90 (m, 1H), 2.95-2.75 (m, 1H), 2.50-2.20 (m, 12H), 2.00-1.84 (m, 2H), 1.82 (s, 3H), 1.75-1.50 (m, 2H), 0.84 (t, *J* = 7.5 Hz, 3H); **^{13}C NMR** (75 MHz, $CDCl_3$) δ 199.6, 160.6, 138.0, 132.2, 131.5, 128.9, 127.5, 126.2, 123.8, 44.8, 38.2, 36.8, 25.8, 24.9, 22.6, 21.3, 12.1, 10.8; **IR** (neat) 2929, 2866, 1662, 1604, 1455, 1300, 1033, 966, 848, 693 cm^{-1}; **HRMS** (ESI-TOF) *m/z* calcd for $C_{21}H_{29}O$ $(M+H)^+$: 297.2218, found 297.2225.

(*S,E*)-3-(6-(4-methoxyphenyl)hex-5-en-3-yl)-5,5-dimethylcyclohex-2-en-1-one (**3dd**)

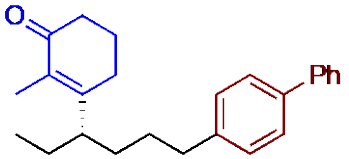

3dd

<u>The general procedure C</u> was followed using (*E*)-4-(hex-3-en-1-yl)-1,1'-biphenyl (**1dd**, 118 mg, 0.50 mmol) and 2-methyl-3-oxocyclohex-1-en-1-yl trifluoromethanesulfonate (**2a**, 52 mg, 0.20 mmol) to afford a colorless oil. Then 10 mol % of Pd/C was added to this oil with 0.5 mL methanol under a balloon of H_2. After stirring for 1h at rt, the solution was passed through a short silica plug remove any solid to give product **3dd** as a colorless oil (48.4 mg, 70% yield): **R_f** = 0.30 (25% Acetone/hexanes); **^{1}H NMR** (500 MHz, $CDCl_3$) δ 7.58 (d, *J* = 10.0 Hz, 2H), 7.51 (d, *J* = 10.0 Hz, 2H), 7.43 (t, *J* = 7.5 Hz, 2H), 7.32 (t, *J* = 7.5 Hz, 1H), 7.21 (d, *J* = 10.0 Hz, 2H), 2.95-2.50 (m, 3H), 2.48-2.30 (m, 2H), 2.30-2.05 (m, 2H), 2.00-1.70 (m, 5H),1.65-1.40 (m, 4H), 1.35-1.20 (m, 2H), 0.81 (t, *J* = 5.0 Hz, 3H); **^{13}C NMR** (75 MHz, $CDCl_3$) δ 199.5, 161.2, 141.2, 141.0, 138.8, 132.4, 128.73, 128.69, 127.04, 127.95, 44.4, 38.2, 35.6, 32.8, 29.4, 26.4, 24.5, 22.6, 12.1, 11.0; **IR** (neat) 3027, 2930, 2859, 1662, 1486, 1456, 1302, 763, 698 cm^{-1}; **HRMS** (ESI-TOF) *m/z* calcd for $C_{25}H_{30}ONa$ $(M+Na)^+$: 369.2194, found 369.2194.

Correlation byproduct **4**.

<u>The general procedure C</u> was followed using (**1**, 0.50 mmol) and 2-methyl-3-oxocyclohex-1-en-1-yl trifluoromethanesulfonate (**2a**, 52 mg, 0.20 mmol) to afford mixture including product **3** and byproduct **4** as a colorless oil. The ratio of **3** to **4** was obtained by the integration of ^{1}H NMR.

Determination of absolute configuration

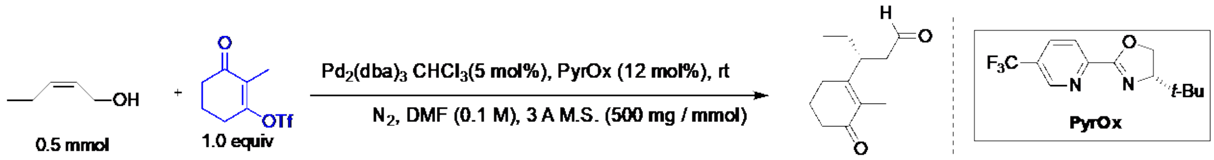

Prepared according to a modified literature procedure.[13] To a dry 20 mL scintillation vial, equipped with a stir bar was added, $Pd_2dba_3{\cdot}CHCl_3$ (26 mg, 0.025 mmol, 5 mol %), magic ligand (PyrOx, 16.5 mg, 0.06 mmol, 12 mol %), 3Å MS (250mg, 50mg/mmol) and sealed using a rubber septum. The reaction vial was evacuated and refilled with N_2 three times. To this, 3 mL dimethyl formamide (DMF) was added under nitrogen and the resulting mixture was stirred for 10 min at room temperature and for another 10 min at rt. To this, a DMF solution (3 mL) of (*Z*)-pent-2-en-1-ol (43 mg, 0.5 mmol) and 2-methyl-3-oxocyclohex-1-en-1-yl trifluoromethanesulfonate (**2a**, 130 mg, 0.50 mmol) were added via syringe. After that, the resulting mixture was stirred at rt for 16 h. The mixture was diluted with ethyl acetate (150 mL) and water (100 mL). The aqueous layer was extracted with ethyl acetate (2 x 100 mL). The combined organic layers were washed with water (2 x 50 mL), brine (1 x 50 mL), and dried over sodium sulfate. The organic extracts were concentrated under reduced pressure, and the resulting residue was purified by silica gel flash chromatography using 0 to 30% ethyl acetate in hexanes, to give the corresponding products **3ee-ref** as a colorless oil (48.4 mg, 70% yield): **R***f* = 0.30 (25% Acetone/hexanes);

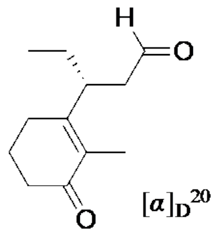

er 90:10
3ee-ref
$[\alpha]_D^{20}$ = +12° (c = 0.1, $CHCl_3$)

(*R*)-3-(2-methyl-3-oxocyclohex-1-en-1-yl)pentanal

^{1}**H NMR** (500 MHz, $CDCl_3$) δ 9.67 (t, J = 3.0 Hz, 1H), 3.35-3.20 (m, 1H), 2.62-2.26 (m, 4H), 2.25-2.10 (m, 2H), 2.00-1.85 (m, 2H), 1.83 (s, 3H),1.65-1.40 (m, 2H), 0.85 (t, J = 6.0 Hz, 3H); ^{13}**C NMR** (75 MHz, $CDCl_3$) δ 200.6, 199.2, 157.9, 132.5, 47.0, 38.6, 37.9, 25.9, 25.0, 22.5, 11.8, 10.8; **IR** (neat) 2932, 2873, 1721, 1658, 1616, 1457, 1380, 1327, 1301, 1125, 1051, 1028, 877 cm^{-1}; **HRMS** (ESI-TOF) *m/z* calcd for $C_{12}H_{19}O_2$ $(M+H)^+$: 195.1385, found 195.1383; $[\alpha]_D^{20}$ = +12° (c = 0.1, $CHCl_3$).

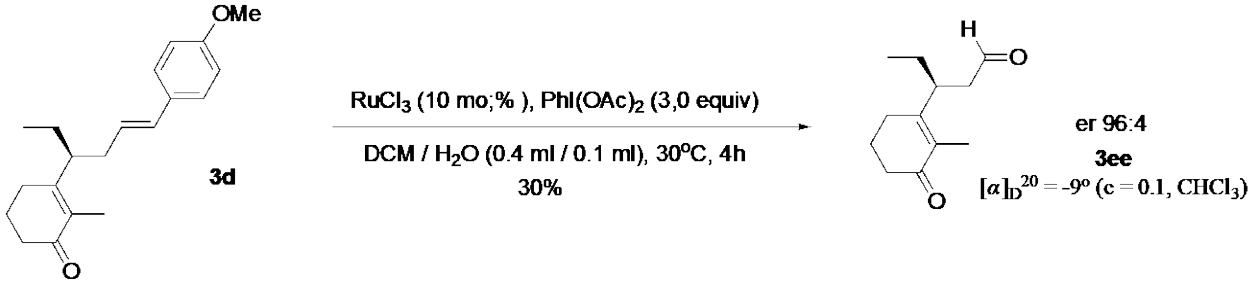

Prepared according to a modified literature procedure.[14] The typical solution containing 0.2 mmol of **3d**, 1 mol % $RuCl_3$ and 3 equiv of $PhI(OAc)_2$, was heated to 30 °C. After Reaction for 4 h at the same temperature, the mixture was then separated with separatory funnel and the DCM phase was dried over anhydrous Na_2SO_4, filtered and the solvent removed under vacuum. The crude product was purified via a standard silica gel chromatography using hexanes/ethyl acetate as eluent to give the desired aldehyde **3ee** ($[\alpha]_D^{20}$ = -9° (c = 0.1, $CHCl_3$)).

SFC Analysis of **3ee** and **3ee-ref**:

The SFC trace of **3ee** was compared to **3ee-ref**.

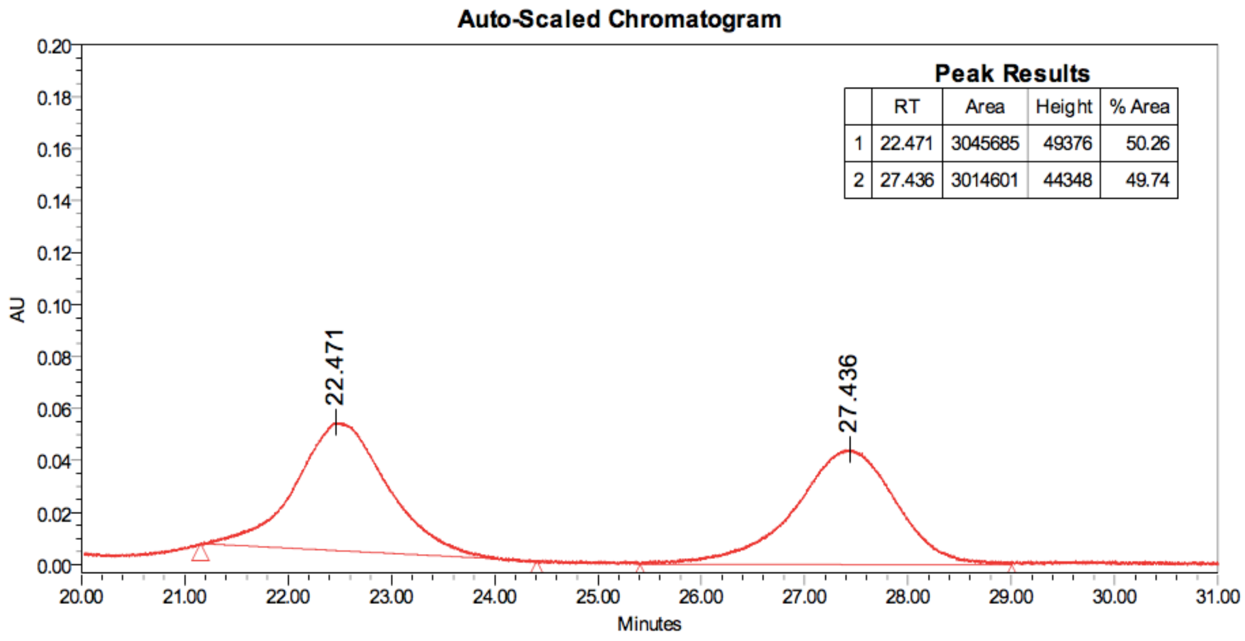

3ee-Rac sample (Conditions): OZ-H column, 40.0 °C, *i*-PrOH: CO_2 = 4:96, 2 mL/min, 138 bar, 40 min; t_1 = 22.471 min, t_2 = 27.436 min.

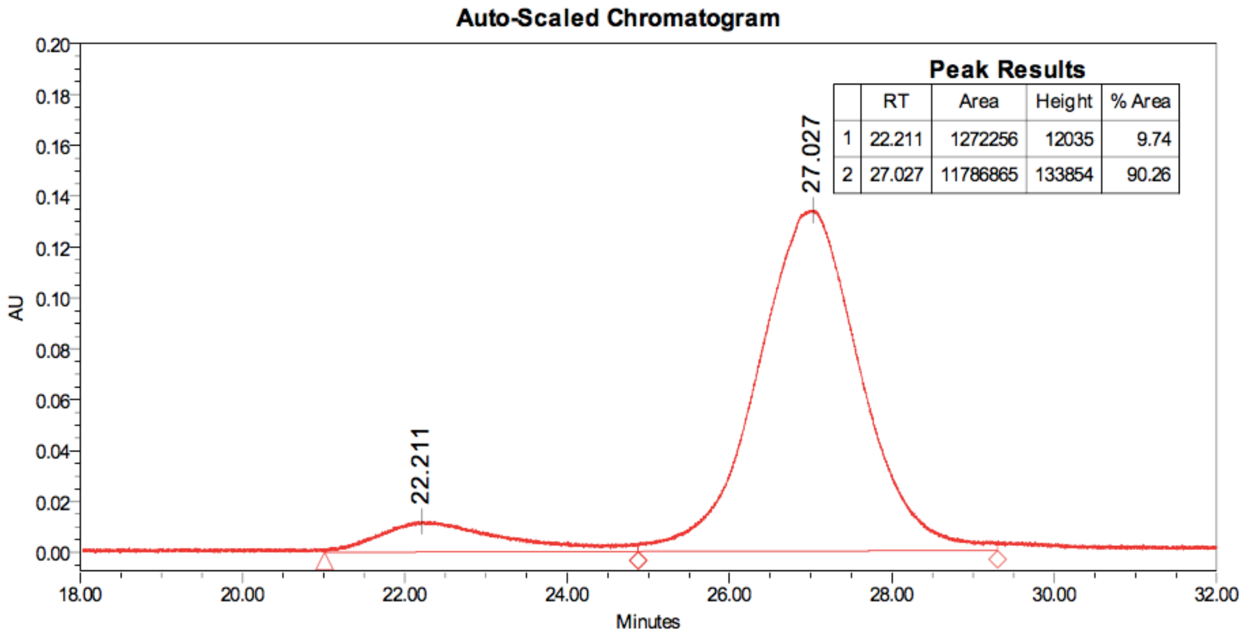

3ee-ref sample (Conditions): OZ-H column, 40.0 °C, *i*-PrOH: CO_2 = 4:96, 2 mL/min, 138 bar, 40 min; t_1 = 22.211 min, t_2 = 27.027 min, er = 10:90.

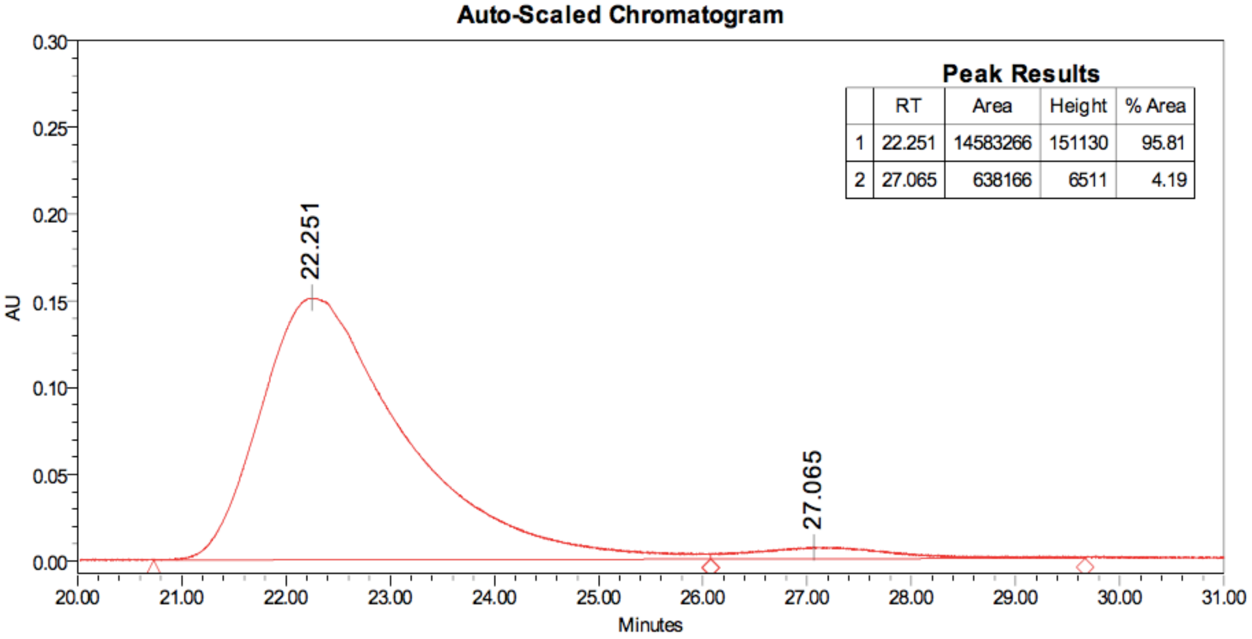

3ee sample (Conditions): OZ-H column, 40.0 °C, *i*-PrOH: CO_2 = 4:96, 2 mL/min, 138 bar, 40 min; t_1 = 22.251 min, t_2 = 27.065 min, er = 96:4.

Computational Analysis of Side Product Formation

Apart from the energy of the unfilled π* orbital, the energy of the filled π orbital at the same position, which is highly collinear with E(π^*_{ortho}) (R^2 = 0.95), unavoidably has a similar correlation with the product selectivity (**Figure S1A**). However, evaluation of non-covalent interactions utilizing symmetry-adapted perturbation theory[15] (SAPT) revealed a positive correlation of side product formation and the π interaction with anion (model system: Br^-), while interaction with cation (model system: B^+) is inversely correlated (**Figure S1B**), which suggests that despite the cationic nature of the organopalladium intermediates, the terminal aryl group of the substrate effects product selectivity by allowing back-bonding into the empty orbital.

Intermediate calculation demonstrated the structural viability for the proposed E2 elimination (**Figure S1C**. **int-1a**: dist(C_α-$H_{\alpha 1}$) = 1.1015 Å, dist(C_α-$H_{\alpha 2}$) = 1.095 Å, φ($H_{\alpha 1}$-C_α-C-Pd) = 160.8°. **int-1b**: dist(C_α-$H_{\alpha 1}$) = 1.093 Å, dist(C_α-$H_{\alpha 2}$) = 1.101 Å, φ($H_{\alpha 2}$-C_α-C-Pd) = 178.5°. It is hypothesized that DMF will act as the deprotonating base. The activated electron-deficient hydrogen is accessible in both cases). Additionally, it is confirmed that the alkenyl-Pd interaction is the main stabilizing force of the γ-Pd intermediate.

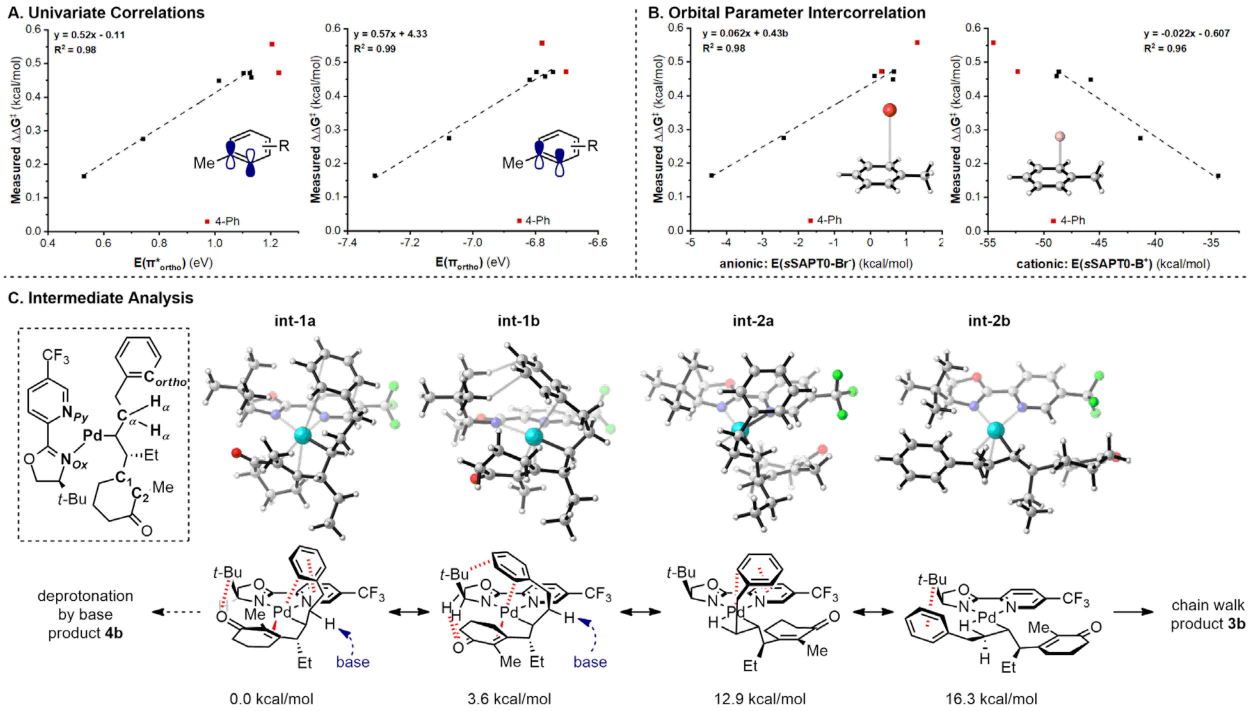

Figure S1. Additional analysis on the chemoselectivity.

On the other hand, through examination of intermediate structural changes during optimization, it was revealed that the alkenyl-Pd interaction is mutually exclusive with the Pd-H_{α} interaction leading up to β-hydride elimination. Furthermore, the stabilizing Pd-π_{Ar} interaction also disfavors, is not prohibits, the β-hydride elimination (**int-2a**: dist(C_{α}-$H_{\alpha 2}$) = 1.168 Å, dist(Pd-$H_{\alpha 2}$) = 1.965 Å, φ($H_{\alpha 2}$-C_{α}-C-Pd) = 19.4°. **int-2b**: dist(C_{α}-$H_{\alpha 2}$) = 1.2125 Å, dist(Pd-$H_{\alpha 2}$) = 1.755 Å, φ($H_{\alpha 2}$-C_{α}-C-Pd) = 1.7°).

Computational Methods

All geometry optimizations were performed under ideal gas phase approximation and ultrafine integration grid using the Gaussian 16[16] software. Toluene derivatives were optimized in the gas phase with the functional B3LYP-D3(BJ)[17] and the basis set 6-31+G(d,p).[18] Single-point energies and molecular descriptors were acquired from the optimized structures using the functional M06[19] and a triple-ζ basis set def2-TZVP.[20] NBO[21a, 18c, 21b] partial charges and orbitals were computed using NBO 3.1[22] (accessed through Gaussian 16). SAPT calculations were performed with the optimized structures using PSI4,[23] with the benchmarked error-cancelling computational level *s*SAPT0/jun-cc-pVDZ.[15d] Boltzmann averages were taken for molecules with multiple conformers. The intermediate complexes were optimized with the functional B3LYP-D3(BJ), the basis set LanL2DZ and its corresponding effective core potential[24] (ECP) for Pd, and the basis set 6-31G(d,p) for all other atoms. Geometry information was extracted from the optimized structures. Single-point energies were acquired from these structures utilizing the functional M06,[19b] the basis set SDD and its ECP for Pd, and the basis set def2TZVP for all other atoms.

References:

1. E. W. Werner, T.-S. Mei, A. J. Burckle, and M. S. Sigman, *Science* 2012, **338**, 1455.
2. T. T. Hoang, and G. B. Dudley, *Org. Lett.* 2013, **15**, 4026.
3. (a) G. Blond, C. Bour, B. Salem, and Suffert, J. *Org. Lett.* 2008, **10**, 1075; (b) P. von. Zezschwitz, F. Petry, and A. de. Meijere, *Chem. Eur. J.* 2001, **7**, 4035.

4. A. Abad, C. Agullo, A. C. Cunat, D. Jimenez, and R. H. Perni, *Tetrahedron* 2001, **57**, 9727.
5. J. R. Calvin, M. O. Frederick, D. L. T. Laird, J. R. Remacle, and Scott A. May, *Org. Lett.* 2012, **14**, 1038.
6. D. Babinski, O. Soltani, and E. D. Frantz, *Org. Lett.* 2008, **10**, 2901.
7. L.-C. Campeau, S. J. Dolman, D. Gauvreau, E. Corley, J. Liu, E. N. Guidry, S. G. Ouellet, D. Steinhuebel, M. Weisel, and P. D. O'Shea, *Org. Proc. Res. Dev.* 2011, **15**, 1138.
8. V. Saini, M. O'Dair, and M. S. Sigman, *J. Am. Chem. Soc.* 2015, **137**, 608.
9. W. M. Czaplik, M. Mayer, A. J. von Wangelin, *Angew. Chem. Int. Ed.* 2009, **48**, 607.
10. G. A. Molander, and R. Figueroa, *Org. Lett.* 2006, **8**, 75-78;
11. P. Yates, and T. S. Macas, *Can. J. Chem.* 1988, **66**, 1.
12. S. A. Snyder, D. S. Treitler, A. P. Brucks, and W. Sattler, *J. Am. Chem. Soc.* 2011, **133**, 15898.
13. H. H. Patel, and M. S. Sigman, *J. Am. Chem. Soc.* 2015, **137**, 3462.
14. C. Mi, L. Li, X.-G. Meng, R.-Q. Yang, and X.-H. Liao, *Tetrahedron* 2016, **72**, 6705.
15. (a) B. Jeziorski, R. Moszynski and K. Szalewicz, *Chem. Rev.*, 1994, **94**, 1887-1930; (b) B. Jeziorski, R. Moszynski, A. Ratkiewicz, S. Rybak, K. Szalewicz and H. L. Williams, 1998; (c) A. J. Misquitta, R. Podeszwa, B. Jeziorski and K. Szalewicz, *J. Chem. Phys.*, 2005, **123**, 214103; (d) T. M. Parker, L. A. Burns, R. M. Parrish, A. G. Ryno and C. D. Sherrill, *J. Chem. Phys.*, 2014, **140**, 094106; (e) R. Bukowski, W. Cencek, P. Jankowski, M. l. Jeziorska, B. Jeziorski, S. A. Kucharski, V. F. Lotrich, M. P. Metz, A. J. Misquitta, R. Moszyński, K. Patkowski, Rafał Podeszwa, F. Rob, S. Rybak, K. Szalewicz, H. L. Williams, R. J. Wheatley, P. E. S. Wormer and P. S. Żuchowski, *sapt2016: An Ab Initio Program for Symmetry-Adapted Perturbation Theory Calculations of Intermolecular Interaction Energies. Sequential and parallel versions.*, 2016.
16. M. J. Frisch, G. W. Trucks, H. B. Schlegel, G. E. Scuseria, M. A. Robb, J. R. Cheeseman, G. Scalmani, V. Barone, G. A. Petersson, H. Nakatsuji, X. Li, M. Caricato, A. V. Marenich, J. Bloino, B. G. Janesko, R. Gomperts, B. Mennucci, H. P. Hratchian, J. V. Ortiz, A. F. Izmaylov, J. L. Sonnenberg, D. Williams-Young, F. Ding, F. Lipparini, F. Egidi, J. Goings, B. Peng, A. Petrone, T. Henderson, D. Ranasinghe, V. G. Zakrzewski, J. Gao, N. Rega, G. Zheng, W. Liang, M. Hada, M. Ehara, K. Toyota, R. Fukuda, J. Hasegawa, M. Ishida, T. Nakajima, Y. Honda, O. Kitao, H. Nakai, T. Vreven, K. Throssell, J. J. A. Montgomery, J. E.

Peralta, F. Ogliaro, M. J. Bearpark, J. J. Heyd, E. N. Brothers, K. N. Kudin, V. N. Staroverov, T. A. Keith, R. Kobayashi, J. Normand, K. Raghavachari, A. P. Rendell, J. C. Burant, S. S. Iyengar, J. Tomasi, M. Cossi, J. M. Millam, M. Klene, C. Adamo, R. Cammi, J. W. Ochterski, R. L. Martin, K. Morokuma, O. Farkas, J. B. Foresman and D. J. Fox, *Gaussian 16, Revision A.03*, 2016.

17. (a) S. H. Vosko, L. Wilk and M. Nusair, *Can. J. Phys.*, 1980, **58**, 1200-1211; (b) C. Lee, W. Yang and R. G. Parr, *Phys. Rev. B*, 1988, **37**, 785-789; (c) A. D. Becke, *J. Chem. Phys.*, 1993, **98**, 5648-5652; (d) P. J. Stephens, F. J. Devlin, C. F. Chabalowski and M. J. Frisch, *J. Phys. Chem.*, 1994, **98**, 11623-11627; (e) S. Grimme, S. Ehrlich and L. Goerigk, *J. Comput. Chem.*, 2011, **32**, 1456-1465.
18. (a) G. W. Spitznagel, T. Clark, J. Chandrasekhar and P. V. R. Schleyer, *J. Comput. Chem.*, 1982, **3**, 363-371; (b) B. J. Lynch, Y. Zhao and D. G. Truhlar, *J. Phys. Chem. A*, 2003, **107**, 1384-1388; (c) L. Goodman and R. R. Sauers, *J. Comput. Chem.*, 2007, **28**, 269-275.
19. (a) Y. Zhao and D. G. Truhlar, *Acc. Chem. Res.*, 2008, **41**, 157-167; (b) Y. Zhao and D. G. Truhlar, *Theor. Chem. Acc.*, 2008, **120**, 215-241.
20. F. Weigend and R. Ahlrichs, *Phys. Chem. Chem. Phys.*, 2005, **7**, 3297-3305.
21. (a) A. E. Reed, L. A. Curtiss and F. Weinhold, *Chem. Rev.*, 1988, **88**, 899-926; (b) E. D. Glendening, C. R. Landis and F. Weinhold, *Wiley Interdiscip. Rev. Comput. Mol. Sci.*, 2012, **2**, 1-42.
22. E. D. Glendening, A. E. Reed, J. E. Carpenter and F. Weinhold, *NBO Version 3.1*
23. R. M. Parrish, L. A. Burns, D. G. A. Smith, A. C. Simmonett, A. E. DePrince, E. G. Hohenstein, U. Bozkaya, A. Y. Sokolov, R. Di Remigio, R. M. Richard, J. F. Gonthier, A. M. James, H. R. McAlexander, A. Kumar, M. Saitow, X. Wang, B. P. Pritchard, P. Verma, H. F. Schaefer, K. Patkowski, R. A. King, E. F. Valeev, F. A. Evangelista, J. M. Turney, T. D. Crawford and C. D. Sherrill, *J. Chem. Theory Comput.*, 2017, **13**, 3185-3197.
24. (a) P. J. Hay and W. R. Wadt, *J. Chem. Phys.*, 1985, **82**, 299-310; (b) P. J. Hay and W. R. Wadt, *J. Chem. Phys.*, 1985, **82**, 270-283.

Table of Descriptors

entry	**E(π^*_{ortho})** (eV)	**E(π_{ortho})** (eV)	**E(*s*SAPT0-Br$^-$)** (kcal/mol)	**E(*s*SAPT0-B$^+$)** (kcal/mol)	**$\Delta\Delta G^{\ddagger}$** (kcal/mol)
4-OMe-C_6H_4	1.126	-6.796	0.329	-48.608	0.472

4-Me-C_6H_4	1.103	-6.743	0.664	-48.684	0.472
3-Me-C_6H_4	1.131	-6.768	0.120	-48.840	0.459
C_6H_5	1.014	-6.819	0.635	-45.779	0.448
4-CO_2Et-C_6H_4	0.743	-7.076	-2.413	-41.335	0.275
4-CF_3-C_6H_4	0.528	-7.314	-4.420	-34.373	0.164
3,5-OMe-C_6H_3	1.205	-6.780	1.304	-54.554	0.558
3,5-Me-C_6H_3	1.229	-6.703	0.299	-52.360	0.473
4-Ph-C_6H_4	0.971	-6.851	-1.669	-49.157	0.03

Cartesians

4-CO_2Et-C_6H_4_conf1

C	-4.411207	-0.674149	0.016856
H	-4.803172	-0.630479	1.040630
H	-5.000093	0.022853	-0.587316
H	-4.584973	-1.686021	-0.359867
C	-2.945276	-0.320274	-0.009408
C	-1.961436	-1.319135	-0.011650
C	-0.605948	-0.995253	-0.006818
C	-0.205954	0.347600	-0.002003
C	-1.180981	1.354672	-0.003388
C	-2.531667	1.021112	-0.008203
H	-2.261711	-2.363420	-0.018997
H	0.144592	-1.776837	-0.010112
H	-0.860084	2.390671	-0.003849
H	-3.278853	1.810236	-0.012972
C	1.224276	0.753391	0.000925
O	1.613404	1.908399	0.004036
O	2.064805	-0.305865	0.000423
C	3.478479	0.002214	0.003815
H	3.705420	0.608021	-0.878524
H	3.701905	0.604786	0.889258
C	4.229133	-1.314530	0.002886
H	3.985536	-1.903357	-0.886551
H	5.307238	-1.123992	0.005382
H	3.981998	-1.906621	0.889176

4-CO_2Et-C_6H_4_conf2

C	-4.445894	-0.379919	-0.182231
H	-4.915695	-0.229665	0.797731
H	-4.906318	0.329956	-0.876302
H	-4.691803	-1.392540	-0.514047
C	-2.954148	-0.174894	-0.096491
C	-2.082627	-1.264056	0.044335
C	-0.706007	-1.075939	0.152712
C	-0.170404	0.218419	0.119181
C	-1.032119	1.314653	-0.025303
C	-2.404957	1.116477	-0.132327
H	-2.488144	-2.271896	0.067891
H	-0.042950	-1.926212	0.259761
H	-0.606391	2.311716	-0.054300
H	-3.063158	1.973538	-0.247462
C	1.288838	0.480764	0.226966
O	1.790305	1.591472	0.201021
O	2.010057	-0.656246	0.362499
C	3.448256	-0.508587	0.454321
H	3.672286	0.372892	1.059388
H	3.774128	-1.407665	0.981749
C	4.080135	-0.408779	-0.924866
H	3.748277	0.497742	-1.436892
H	5.170690	-0.369501	-0.828336
H	3.819730	-1.279001	-1.535115

4-Me-C_6H_4

C	-2.931919	-0.001731	0.005180
H	-3.314131	-0.117573	1.027172
H	-3.337410	0.933325	-0.392716
H	-3.336762	-0.827008	-0.589304
C	-1.422148	0.001656	-0.013831
C	-0.697373	-1.198279	-0.007876
C	0.697392	-1.198257	0.007820
C	1.422160	0.001691	0.013792
C	0.698348	1.199921	0.007767
C	-0.698391	1.199909	-0.007820
H	-1.232040	-2.145118	-0.013683
H	1.232064	-2.145097	0.013597
H	1.232600	2.146818	0.013371
H	-1.232680	2.146787	-0.013465
C	2.931938	-0.001715	-0.005080
H	3.336734	-0.825336	0.591728
H	3.314234	-0.120394	-1.026698
H	3.337350	0.934429	0.390294

3-Me-C_6H_4

C	-2.527668	-1.035051	0.000212
H	-3.133448	-0.781482	-0.877489
H	-2.361145	-2.115991	-0.008000
H	-3.125570	-0.793962	0.886920
C	-1.221313	-0.276846	-0.000240
C	0.003784	-0.949885	-0.000399
C	1.228773	-0.264568	-0.000127
C	1.208944	1.133516	-0.000027
C	-0.006928	1.824824	0.000131
C	-1.212717	1.126885	-0.000051
H	0.007914	-2.038176	-0.000614
H	2.145640	1.684384	-0.000041
H	-0.010331	2.911091	0.000150
H	-2.154498	1.669891	-0.000168
C	2.530433	-1.030481	0.000185
H	3.389852	-0.354190	-0.005930
H	2.613408	-1.671094	0.885822
H	2.608337	-1.680835	-0.878753

4-Ph-C_6H_4

C	-4.620507	-0.007329	0.016218
H	-4.995927	-0.478249	0.933385
H	-5.027892	1.007162	-0.022908
H	-5.028583	-0.570409	-0.829200
C	-3.111435	0.004560	-0.008064
C	-2.387142	-1.126555	-0.409490
C	-0.993650	-1.130185	-0.406762
C	-0.267509	0.001917	-0.001632
C	-0.992769	1.135410	0.397462
C	-2.387321	1.134997	0.391686
H	-2.921759	-2.013524	-0.740352
H	-0.460738	-2.011740	-0.749939
H	-0.460072	2.018852	0.736053
H	-2.921862	2.025855	0.711521
C	1.215100	0.000476	-0.000019
C	1.938419	1.136831	-0.399996
C	1.934536	-1.137724	0.401780
C	3.333481	1.136437	-0.396394
H	1.401822	2.017558	-0.739140
C	3.329591	-1.140653	0.402477
H	1.394771	-2.017305	0.738928
C	4.036071	-0.002923	0.004134
H	3.872105	2.023426	-0.716844
H	3.865138	-2.028983	0.724366
H	5.121795	-0.004207	0.005724

C_6H_5

C	-2.424697	0.000965	0.009465
H	-2.803014	-0.136707	1.029994
H	-2.829327	0.944991	-0.367045
H	-2.831555	-0.811149	-0.601404
C	-0.914727	0.002211	-0.011664
C	-0.196902	-1.202316	-0.008887
C	1.199270	-1.207281	0.002023
C	1.905781	-0.001675	0.008354
C	1.203072	1.205229	0.001966
C	-0.193976	1.203999	-0.008801
H	-0.738043	-2.145303	-0.017351
H	1.734960	-2.152157	0.002128
H	2.991531	-0.003218	0.014153
H	1.741322	2.148637	0.001770
H	-0.732799	2.148105	-0.016978

4-OMe-C_6H_4

C	-3.330991	-0.299383	0.000192
H	-3.794122	0.158579	0.882089
H	-3.793975	0.157009	-0.882607
H	-3.591808	-1.361617	0.001105
C	-1.833690	-0.104162	0.000116
C	-0.953701	-1.187257	-0.000176
C	0.436753	-1.012066	-0.000408
C	0.966777	0.280759	-0.000318
C	0.099443	1.383351	-0.000010
C	-1.275667	1.186459	0.000196
H	-1.351750	-2.198729	-0.000217
H	1.080543	-1.883068	-0.000682
H	0.526266	2.380873	0.000077
H	-1.932936	2.052591	0.000425
O	2.304252	0.573213	-0.000677
C	3.223558	-0.508944	0.000730
H	4.216544	-0.058171	0.001507
H	3.105237	-1.132443	0.896147
H	3.107089	-1.133273	-0.894360

4-CF_3-C_6H_4

C	3.922793	-0.004580	0.004076
H	4.304979	-0.129616	-1.016464
H	4.327690	0.932767	0.395584
H	4.321158	-0.827482	0.605308
C	2.414368	0.001348	0.012715
C	1.692930	-1.201539	0.003272
C	0.300362	-1.205806	-0.020487
C	-0.397613	0.005798	-0.035319
C	0.303002	1.213890	-0.020191
C	1.697292	1.204907	0.003244
H	2.228742	-2.146610	0.013778
H	-0.242559	-2.144621	-0.030138
H	-0.236590	2.154288	-0.029360

H	2.235729	2.148305	0.013666
C	-1.898756	0.001250	-0.004643
F	-2.424433	-1.047357	-0.688200
F	-2.431472	1.130277	-0.535062
F	-2.382474	-0.091658	1.266776

3,5-diMe-C_6H_3

C	0.102120	2.918327	-0.000052
H	-0.403481	3.330312	0.880968
H	1.132245	3.285399	0.002003
H	-0.399798	3.330021	-0.883318
C	0.056396	1.408701	0.000070
C	-1.177673	0.738092	-0.000056
C	-1.248185	-0.655519	-0.000081
C	-0.050386	-1.388950	0.000021
C	1.191788	-0.753196	0.000081
C	1.228058	0.650835	0.000167
H	-2.098045	1.318348	-0.000128
H	-0.092689	-2.476139	0.000008
H	2.190756	1.157783	0.000212
C	-2.578438	-1.370712	0.000025
H	-2.683799	-2.011792	0.882916
H	-3.411370	-0.662098	-0.001346
H	-2.682697	-2.014027	-0.881351
C	2.476315	-1.547599	-0.000090
H	3.085254	-1.316830	0.881666
H	2.279161	-2.623246	0.000446
H	3.084491	-1.317603	-0.882595

3,5-diOMe-C_6H_3_conf1

C	-1.037942	3.076645	0.000037
H	-1.662199	3.257743	0.882662
H	-0.231907	3.814926	-0.000026
H	-1.662418	3.257792	-0.882416
C	-0.492946	1.668232	-0.000039
C	-1.387128	0.579340	-0.000172
C	-0.885337	-0.721042	-0.000195
C	0.498984	-0.963665	-0.000094
C	1.371202	0.120892	0.000003
C	0.877209	1.437693	0.000039
H	-2.453277	0.770406	-0.000299
H	0.837833	-1.990882	-0.000100
H	1.586469	2.258311	0.000119
O	-1.663139	-1.847041	-0.000374
C	-3.074558	-1.686944	0.000390
H	-3.489098	-2.695625	0.000750
H	-3.414608	-1.151618	0.895700
H	-3.415624	-1.151874	-0.894692
O	2.732828	0.004240	0.000103
C	3.293591	-1.301327	0.000075
H	4.375071	-1.161607	0.000164
H	2.996829	-1.862105	0.895148
H	2.996962	-1.862005	-0.895106

3,5-diOMe-C_6H_3_conf2

C	-3.455542	-0.000033	0.013431
H	-3.825553	-0.001719	1.046081
H	-3.862313	0.887451	-0.480153
H	-3.862428	-0.885846	-0.483055
C	-1.945557	-0.000044	-0.013517
C	-1.241753	-1.208007	-0.011143
C	0.155641	-1.204081	-0.000505
C	0.873630	0.000007	0.004865
C	0.155580	1.204111	-0.000735
C	-1.241772	1.207991	-0.011366
H	-1.759808	-2.161117	-0.018693
H	1.953062	0.000051	0.008886
H	-1.759967	2.161014	-0.019079
O	0.753055	-2.435564	0.000540
C	2.170956	-2.501930	0.002590
H	2.419912	-3.563667	0.001395
H	2.595358	-2.028144	-0.891795
H	2.592796	-2.030964	0.899685
O	0.753009	2.435587	-0.000015
C	2.170905	2.501963	0.003159
H	2.419840	3.563706	0.002251
H	2.592032	2.030914	0.900541
H	2.596030	2.028283	-0.890941

int-1a

C	-1.086755	-1.426044	-1.365009
C	-2.131944	-2.060747	-2.029675
C	-3.398030	-1.480953	-1.989702
C	-3.558425	-0.286394	-1.296874
C	-2.454743	0.294674	-0.670416
N	-1.245099	-0.259980	-0.697202
C	1.963708	-3.283114	-1.981683
H	2.144812	-4.349636	-1.870770
H	2.385162	-2.931029	-2.926985
O	0.510868	-3.087444	-2.060521
N	1.239027	-1.473114	-0.668947
C	0.270941	-1.984598	-1.343726
H	-1.943732	-2.986462	-2.558600
Pd	0.636361	0.568290	0.125874
C	2.382984	-2.421879	-0.771785
C	2.601064	-3.223190	0.545619
H	3.295830	-1.863765	-0.978714
C	2.767343	-2.241150	1.716593
H	3.617787	-1.577702	1.546312
H	1.872632	-1.628302	1.848721
H	2.942551	-2.793601	2.644682
C	1.425342	-4.175274	0.822039
H	1.347924	-4.970843	0.075216
H	1.564659	-4.661364	1.792097
H	0.472050	-3.642751	0.854055
C	3.901985	-4.029365	0.385034
H	4.101294	-4.602712	1.294769
H	3.848714	-4.744522	-0.442764
H	4.755454	-3.366641	0.211137
H	-4.240857	-1.944700	-2.489294
H	-2.556185	1.230661	-0.139613
C	-4.918794	0.340386	-1.128924
F	-4.822099	1.672746	-0.951685
F	-5.701820	0.105313	-2.192423
F	-5.539247	-0.168657	-0.040535
C	-0.155742	2.353854	0.855298
H	-1.023925	2.506022	0.206046
C	-0.514507	2.314213	2.334531
H	-0.574876	3.365447	2.658002
H	0.314931	1.893170	2.912493
C	-1.829824	1.634329	2.723805
H	-2.655307	2.102530	2.173400
H	-2.019746	1.863392	3.780592
C	-1.890011	0.136366	2.532933
C	-0.781546	-0.684875	2.776304
C	-3.088096	-0.466356	2.131550
C	-0.871819	-2.068381	2.615324
H	0.151285	-0.254546	3.127102
C	-3.174118	-1.844839	1.941598
H	-3.963334	0.151375	1.954676
C	-2.061142	-2.652845	2.180101
H	-0.012825	-2.688318	2.841174
H	-4.111641	-2.282447	1.613382
H	-2.125955	-3.728650	2.051778
C	0.944085	3.359765	0.524991
H	1.490403	3.616145	1.437339
C	0.432672	4.672235	-0.075605
C	1.835155	2.471544	-0.341873
H	-0.357616	5.043410	0.587990
H	-0.053587	4.485016	-1.040318
C	1.518084	5.744153	-0.207544
C	2.687843	1.547251	0.290303
C	1.833439	2.676825	-1.850356
H	1.938691	5.991041	0.772539
H	1.111897	6.663223	-0.637537
H	2.346994	5.418790	-0.844846
C	3.596687	0.700174	-0.563023
C	3.096238	1.591810	1.747861
C	3.117772	2.187078	-2.518560
H	0.964230	2.178485	-2.295872
H	1.697857	3.740900	-2.051618
C	3.441083	0.764898	-2.069412
O	4.436440	-0.017596	-0.041438
H	2.357599	2.062770	2.390729
H	3.289277	0.584614	2.113894
H	4.036352	2.147594	1.842384
H	3.016523	2.238006	-3.606032
H	3.945596	2.852201	-2.245025
H	4.361583	0.380029	-2.513670
H	2.621866	0.091887	-2.356880

int-1b

C	-1.574695	-1.788756	-0.865898
C	-2.659006	-2.584097	-1.235017
C	-3.947480	-2.111304	-1.001850
C	-4.098223	-0.853588	-0.424786
C	-2.960891	-0.115305	-0.098942
N	-1.724095	-0.571973	-0.301035
C	1.393360	-3.467004	-2.053665
H	1.660722	-4.513340	-1.925248
H	1.574155	-3.155358	-3.084901
O	-0.043857	-3.352128	-1.810666
N	0.859406	-1.633349	-0.654539
C	-0.191141	-2.233802	-1.089830
H	-2.480537	-3.546037	-1.698161
Pd	0.208617	0.575379	-0.076773
C	2.020594	-2.502955	-1.023806
C	2.659590	-3.200273	0.209281
H	2.777110	-1.887915	-1.509709
C	3.276479	-2.127930	1.113874
H	4.086321	-1.602884	0.597782
H	2.528151	-1.401705	1.429871
H	3.700528	-2.583705	2.013493
C	1.614770	-4.008698	0.994023
H	1.133824	-4.771976	0.373805
H	2.092085	-4.524872	1.831992
H	0.839444	-3.355965	1.402955
C	3.785808	-4.126851	-0.284902
H	4.334858	-4.528579	0.571478
H	3.412166	-4.981930	-0.855423
H	4.500392	-3.584592	-0.913570
H	-4.816635	-2.698478	-1.274732
H	-3.056698	0.876330	0.322881
C	-5.460245	-0.290637	-0.099385
F	-5.469162	1.050498	-0.243048
F	-6.405665	-0.810501	-0.897553
F	-5.792641	-0.564854	1.176456
C	-0.713574	2.423311	0.108261
H	-1.486127	2.359832	-0.664976
C	-1.319203	2.646705	1.486094
H	-2.168762	1.978296	1.649851
H	-1.751552	3.659316	1.478142
C	-0.361078	2.538077	2.676792
H	-0.906893	2.853767	3.574891
H	0.459368	3.254670	2.571358
C	0.213387	1.159651	2.927696
C	1.513575	1.025120	3.439080
C	-0.568137	0.000099	2.794155
C	2.004719	-0.216219	3.834654
H	2.129105	1.911693	3.563987
C	-0.073180	-1.247921	3.181398
H	-1.593066	0.073543	2.449848
C	1.208571	-1.357781	3.713351
H	3.007388	-0.294218	4.242481
H	-0.703185	-2.127093	3.089373
H	1.589076	-2.324113	4.026051
C	0.408193	3.383264	-0.278739
H	0.722808	3.932953	0.613143
C	0.068117	4.444653	-1.338503
C	1.542746	2.409393	-0.595393
H	-0.674260	5.119620	-0.896149
H	-0.414009	3.988687	-2.205912
C	1.294253	5.241968	-1.786656
C	1.501299	1.558295	-1.702907
C	2.773603	2.549361	0.267991
H	1.782355	5.733893	-0.938562
H	1.015570	6.017085	-2.504783
H	2.034291	4.595377	-2.270905
C	2.708959	0.696673	-2.000837
C	0.533202	1.625256	-2.866105
C	3.688345	1.334974	0.252836
H	3.304607	3.441719	-0.100716
H	2.470266	2.783167	1.291191
C	3.965858	0.897599	-1.183411
O	2.669436	-0.106505	-2.920331
H	-0.459804	1.969443	-2.592120
H	0.933895	2.302307	-3.630841
H	0.459919	0.638560	-3.323175
H	4.626154	1.556287	0.770370
H	3.200491	0.527216	0.803313

H	4.562219	-0.015926	-1.244743
H	4.541833	1.679840	-1.700900

int-2a

C	0.397832	-1.917692	-0.885508
C	-0.159086	-3.179443	-1.056832
C	-1.435498	-3.417448	-0.546586
C	-2.094262	-2.386309	0.112512
C	-1.492221	-1.130272	0.192851
N	-0.279844	-0.898171	-0.306547
C	3.824449	-1.921503	-2.117972
H	4.551878	-2.632591	-1.728513
H	3.969690	-1.799881	-3.192040
O	2.497567	-2.512284	-1.922259
N	2.362782	-0.509480	-0.903898
C	1.786646	-1.604609	-1.249902
H	0.410162	-3.954455	-1.554797
Pd	0.890342	0.894062	0.034901
C	3.771700	-0.572383	-1.344168
C	4.765494	-0.446168	-0.157125
H	3.954326	0.266099	-2.024793
C	4.602408	0.941976	0.484550
H	4.746580	1.741071	-0.250721
H	3.609604	1.057959	0.926552
H	5.342240	1.080707	1.278224
C	4.495837	-1.531860	0.896158
H	4.627058	-2.542145	0.495303
H	5.192042	-1.424960	1.732654
H	3.478825	-1.450284	1.289500
C	6.196213	-0.573134	-0.708514
H	6.921820	-0.434619	0.097701
H	6.387711	-1.556496	-1.149058
H	6.397249	0.186252	-1.471669
H	-1.901589	-4.391057	-0.646199
H	-1.995135	-0.304094	0.667349
C	-3.395776	-2.641794	0.829341
F	-4.077963	-1.503458	1.054421
F	-4.176969	-3.488432	0.148417
F	-3.141089	-3.197369	2.039965
C	-0.470646	2.312196	0.634690
H	-1.254430	1.829300	1.212656
C	0.655034	2.864516	1.439760
H	0.825902	3.931898	1.265102
H	1.642739	2.443166	0.980721
C	0.653174	2.483457	2.939059
H	-0.101058	3.099005	3.439584
H	1.621370	2.746962	3.374829
C	0.351369	1.016914	3.147519
C	1.360790	0.053346	3.003789
C	-0.958296	0.580441	3.389876
C	1.062350	-1.308963	3.070512
H	2.388814	0.371385	2.855074
C	-1.261750	-0.780570	3.446694
H	-1.747099	1.313639	3.536083
C	-0.252166	-1.729564	3.275224
H	1.858808	-2.039844	2.971963
H	-2.283433	-1.102601	3.618633
H	-0.491915	-2.786870	3.316228
C	-0.978389	3.076452	-0.584395
H	-0.116507	3.599119	-1.020871
C	-2.006521	4.186487	-0.234487
C	-1.464734	2.077520	-1.637331
H	-2.863983	3.758791	0.286915
H	-2.386959	4.573724	-1.186619
C	-1.433457	5.338203	0.592495
C	-2.595110	1.338489	-1.527280
C	-0.527168	1.957460	-2.823336
H	-1.171991	5.025677	1.608301
H	-2.169665	6.141126	0.684649
H	-0.538744	5.765393	0.125726
C	-2.872510	0.268107	-2.537504
C	-3.623136	1.422020	-0.423431
C	-0.686774	0.679949	-3.644798
H	-0.691523	2.839779	-3.461874
H	0.508835	2.043730	-2.469972
C	-2.167051	0.366882	-3.871935
O	-3.613251	-0.669073	-2.268527
H	-3.206757	1.677914	0.551742
H	-4.378660	2.178680	-0.661817
H	-4.138470	0.465891	-0.344339
H	-0.158060	0.772151	-4.598226
H	-0.225324	-0.154353	-3.102720
H	-2.320570	-0.567206	-4.415901
H	-2.632888	1.176690	-4.450213

int-2b

C	0.346818	-2.820671	0.233138
C	1.211409	-3.906059	0.170328
C	2.543980	-3.678594	-0.173722
C	2.950918	-2.375666	-0.437907
C	2.022196	-1.334744	-0.359761
N	0.746671	-1.550415	-0.029719
C	-2.964413	-3.919375	1.266608
H	-3.581632	-4.638882	0.732382
H	-3.020087	-4.108144	2.340309
O	-1.572535	-4.156322	0.859661
N	-1.861826	-1.939386	0.621919
C	-1.073374	-2.952238	0.582951
H	0.839152	-4.899374	0.388693
Pd	-0.786577	-0.072788	0.243105
C	-3.228802	-2.436116	0.888969
C	-4.176983	-2.220373	-0.324356
H	-3.635950	-1.885006	1.741733
C	-4.264967	-0.720097	-0.639748
H	-4.556968	-0.130347	0.234879
H	-3.311765	-0.332127	-1.008547
H	-5.011079	-0.539792	-1.417233
C	-3.664123	-2.968613	-1.565257
H	-3.604007	-4.050414	-1.410145
H	-4.342976	-2.801362	-2.405838
H	-2.674952	-2.608030	-1.864827
C	-5.573233	-2.732197	0.070821
H	-6.276653	-2.566217	-0.749625
H	-5.580306	-3.804597	0.290622
H	-5.955400	-2.202386	0.949750
H	3.251343	-4.497302	-0.237706
H	2.318820	-0.311340	-0.545288
C	4.393450	-2.050921	-0.748723
F	5.048540	-1.705604	0.369753
F	5.019774	-3.102841	-1.298172
F	4.467639	-1.013792	-1.612234
C	0.097833	1.778209	0.001134
H	0.334171	1.881261	-1.057186
C	-1.279575	2.193232	0.329840
H	-1.375668	2.663394	1.308355
H	-1.950599	1.206003	0.542673
C	-2.088813	2.908089	-0.762889
H	-1.798106	2.506419	-1.738384
H	-1.776373	3.960356	-0.753215
C	-3.589038	2.800873	-0.600249
C	-4.202120	2.960908	0.649296
C	-4.393591	2.536178	-1.713464
C	-5.585768	2.845422	0.782880
H	-3.604360	3.189557	1.527372
C	-5.778165	2.429421	-1.584849
H	-3.931595	2.410877	-2.688993
C	-6.377831	2.577885	-0.334342
H	-6.045122	2.974566	1.757754
H	-6.386628	2.227341	-2.460636
H	-7.454654	2.492897	-0.231457
C	1.278661	2.009436	0.949550
H	1.293075	1.182985	1.668139
C	1.135983	3.286587	1.834580
C	2.617953	2.020416	0.224710
H	1.988344	3.297895	2.520460
H	0.248909	3.166002	2.467139
C	1.067933	4.621115	1.091779
C	3.717923	1.482395	0.808896
C	2.689215	2.692447	-1.132273
H	2.007511	4.846991	0.581571
H	0.877242	5.434897	1.796541
H	0.267218	4.639538	0.344879
C	5.055653	1.548431	0.132999
C	3.705624	0.780402	2.145464
C	4.067442	3.293267	-1.408169
H	2.458002	1.954843	-1.918249
H	1.915597	3.460858	-1.213294
C	5.143582	2.227856	-1.220869
O	6.041119	1.045564	0.649041
H	3.001786	-0.060560	2.158145
H	4.703531	0.394311	2.350961
H	3.423341	1.452407	2.961811
H	4.105249	3.710371	-2.418713
H	4.242485	4.121689	-0.710968
H	6.156024	2.622425	-1.332578
H	5.024796	1.439609	-1.977479

SAPT Energies

4-CO_2Et-C_6H_4_conf1

4-CO_2Et-C_6H_4_conf2

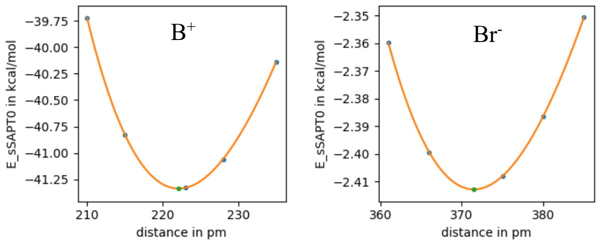

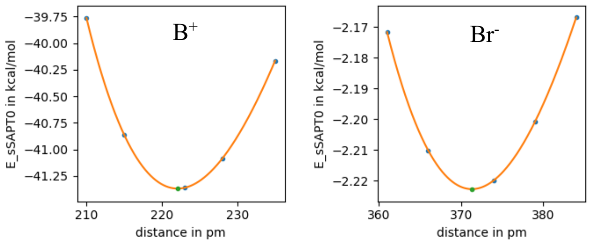

4-Me-C_6H_4

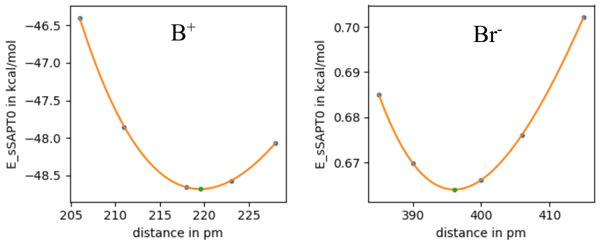

3-Me-C_6H_4

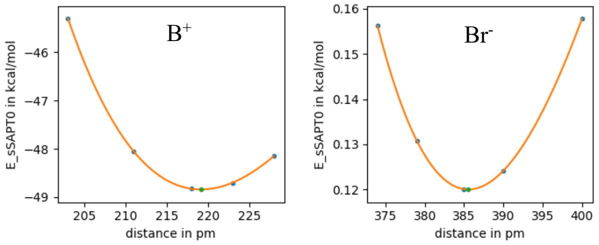

4-Ph-C_6H_4

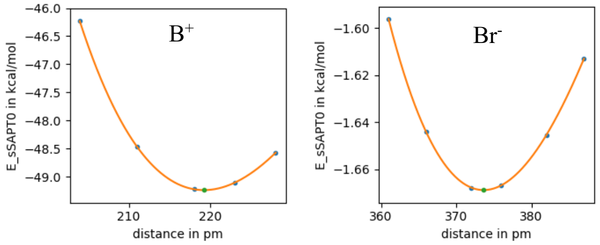

C_6H_5

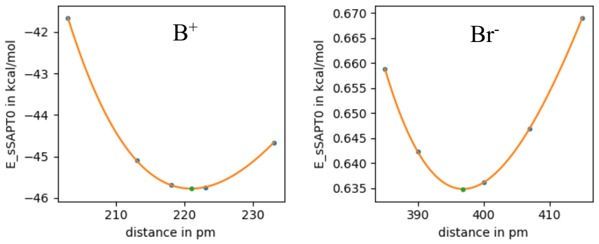

4-OMe-C_6H_4

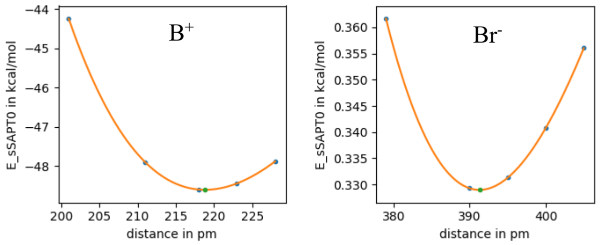

4-CF_3-C_6H_4

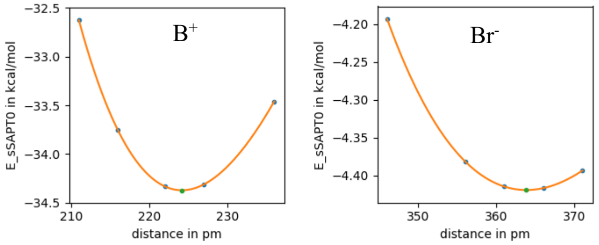

3,5-diMe-C_6H_3

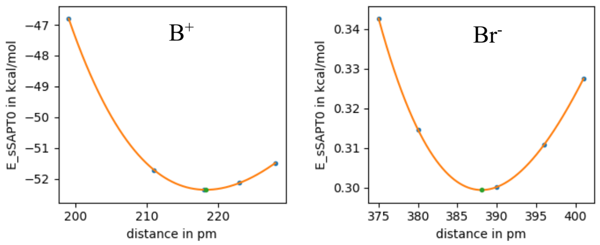

3,5-diOMe-C_6H_3-conf1

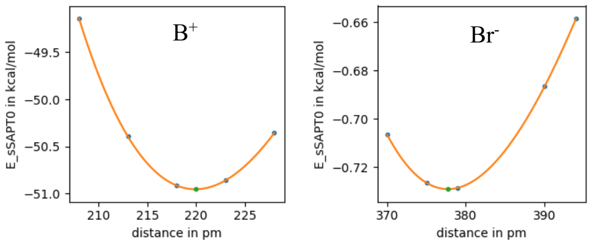

3,5-diOMe-C_6H_3-conf2

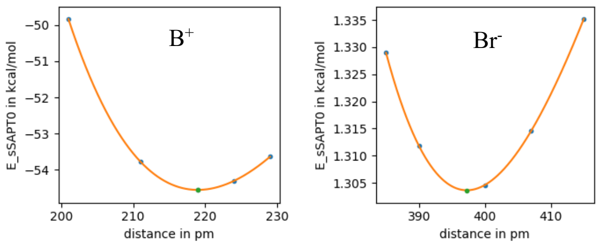

Determination of enantiomeric ratios and SFC traces

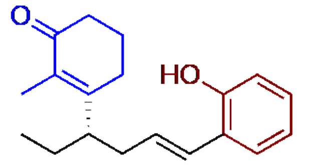

3a

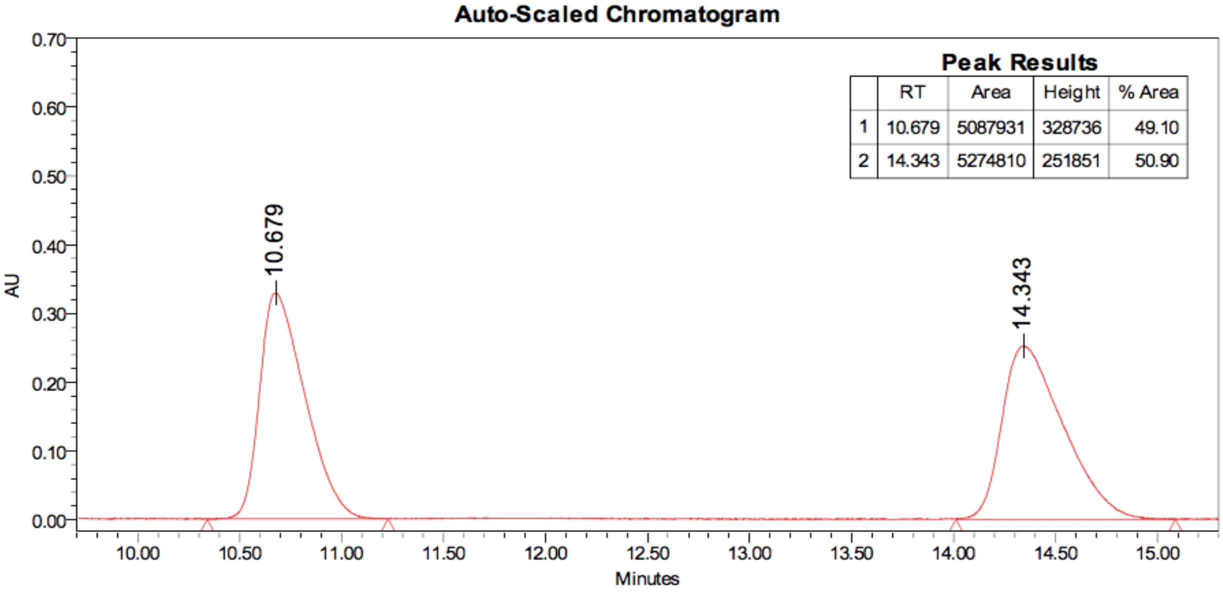

Separation by SFC, Chiralcel® **3a**-Rac sample (Conditions): Cel2 column, 40.0 °C, *i*-PrOH: CO_2 = 5:95, 2 mL/min, 138 bar, 40 min; t_1 = 10.679 min, t_2 = 14.343 min.

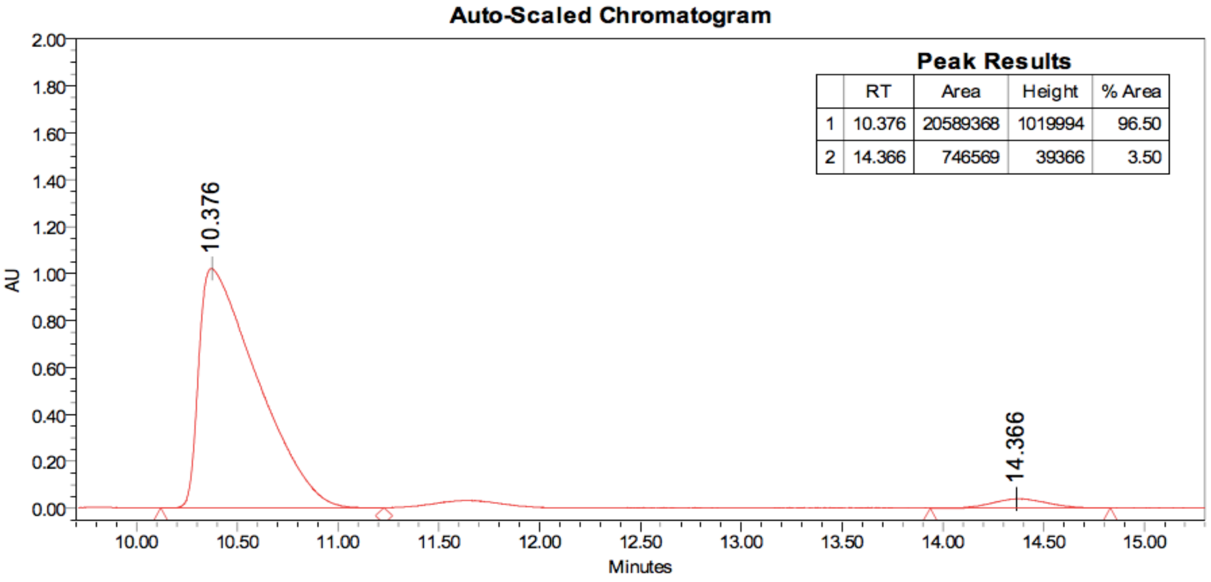

3a sample (Conditions): Cel2 column, 40.0 °C, *i*-PrOH: CO_2 = 5:95, 2 mL/min, 138 bar, 40 min; t_1 = 10.376 min, t_2 = 14.366 min, er = 96.5:3.5.

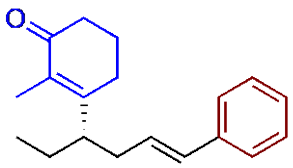

3b

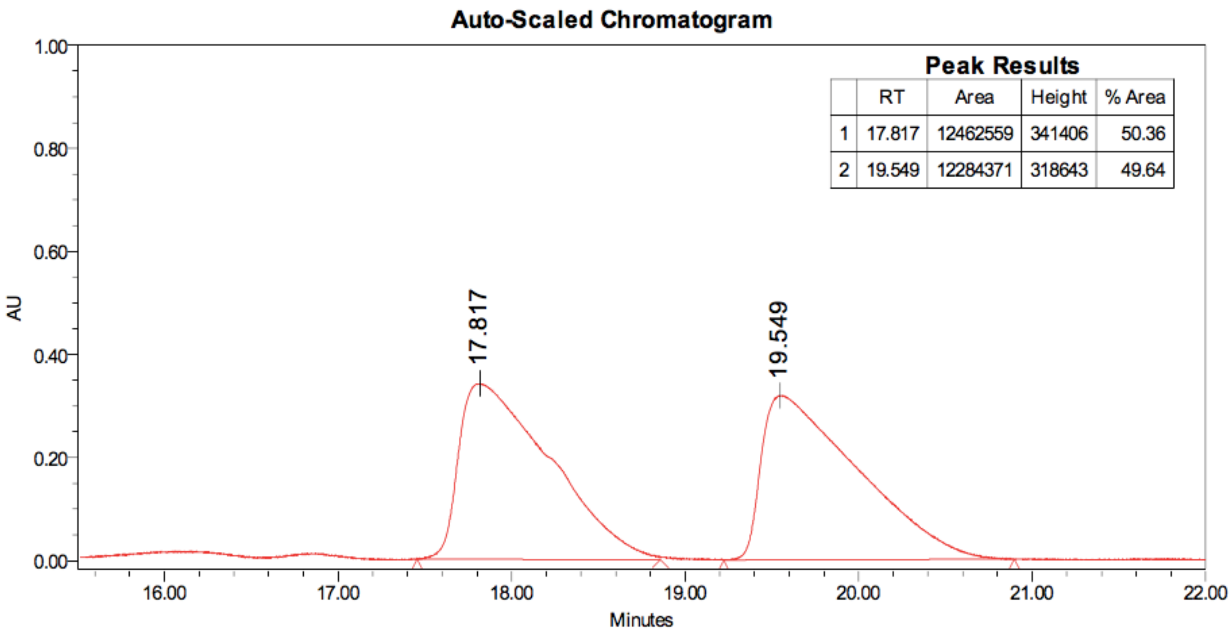

Separation by SFC, Chiralcel® **3b**-Rac sample (Conditions): Cel2 column, 40.0 °C, MeOH: CO_2 = 1:99, 2 mL/min, 138 bar, 40 min; t_1 = 17.817 min, t_2 = 19.549 min.

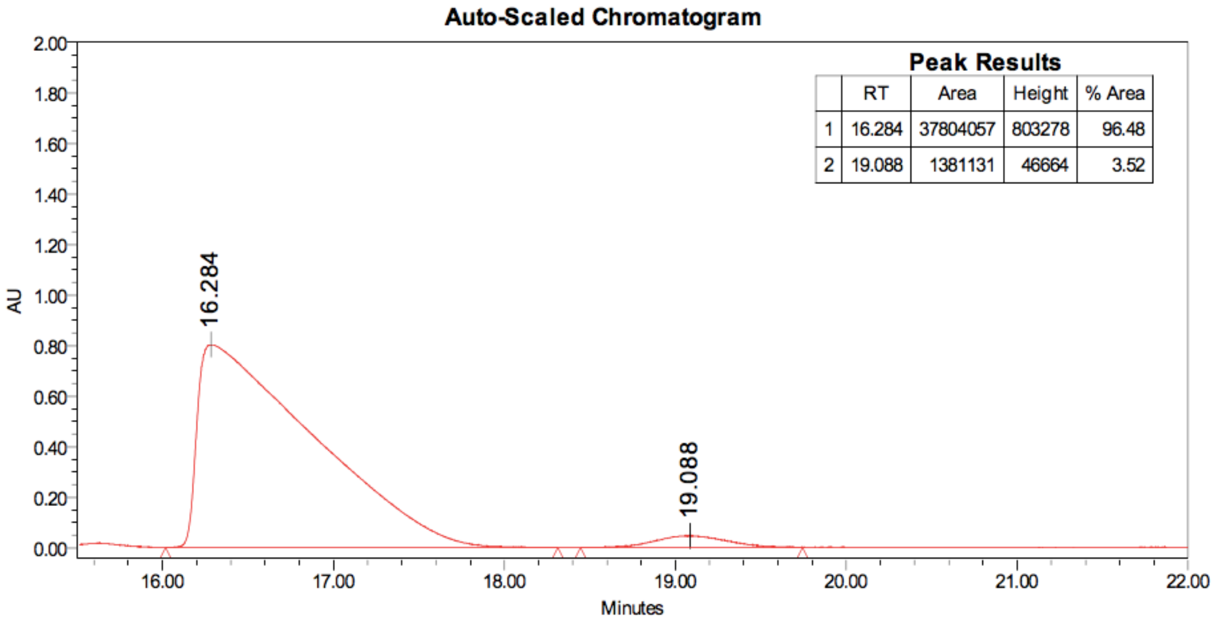

3b sample (Conditions): Cel2 column, 40.0 °C, MeOH: CO_2 = 1:99, 2 mL/min, 138 bar, 40 min; t_1 = 16.284 min, t_2 = 19.088 min, er = 96.5:3.5.

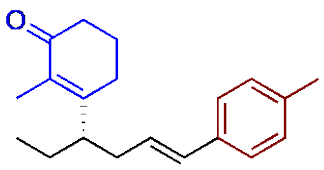

3c

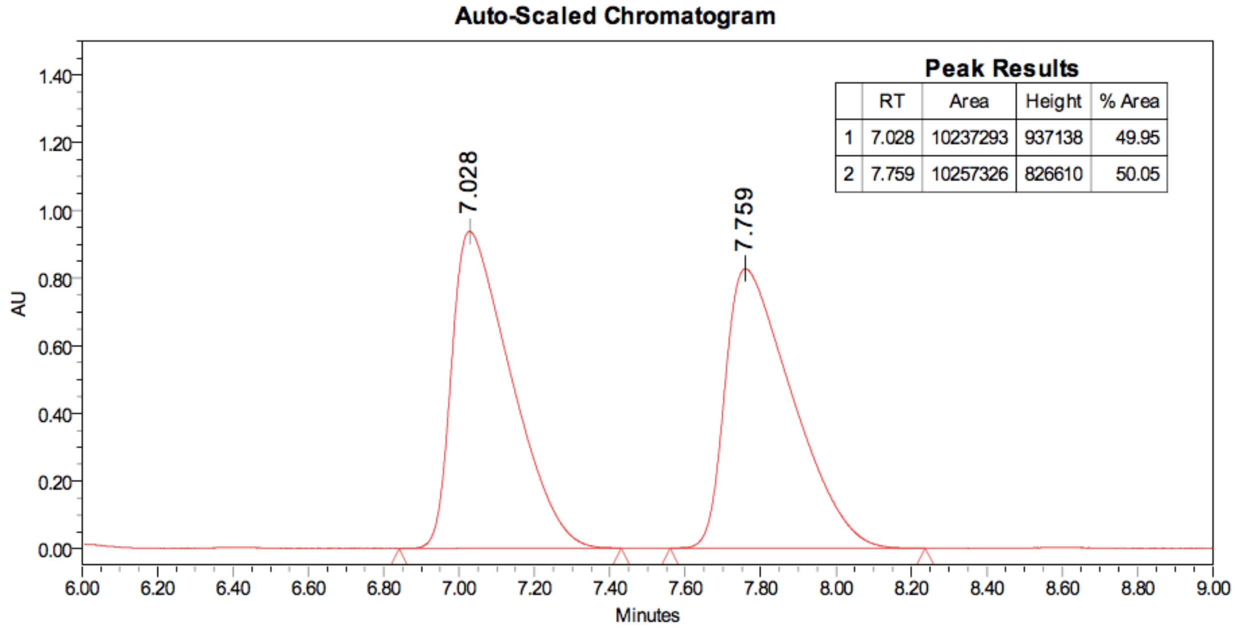

	RT	Area	Height	% Area
1	7.028	10237293	937138	49.95
2	7.759	10257326	826610	50.05

Separation by SFC, Chiralcel® **3c**-Rac sample (Conditions): Cel2 column, 40.0 °C, MeOH: CO_2 = 2:98, 2 mL/min, 138 bar, 20 min; t_1 = 7.028 min, t_2 = 7.759 min.

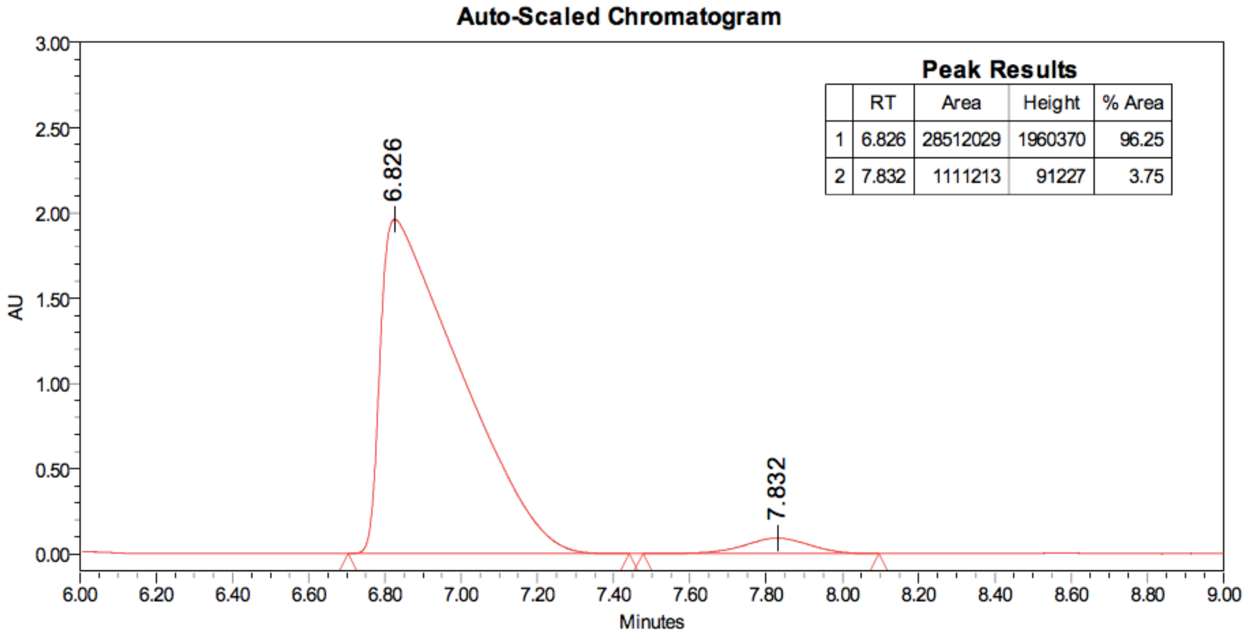

	RT	Area	Height	% Area
1	6.826	28512029	1960370	96.25
2	7.832	1111213	91227	3.75

3c sample (Conditions): Cel2 column, 40.0 °C, MeOH: CO_2 = 2:98, 2 mL/min, 138 bar, 40 min; t_1 = 6.826 min, t_2 = 7.832 min, er = 96:4.

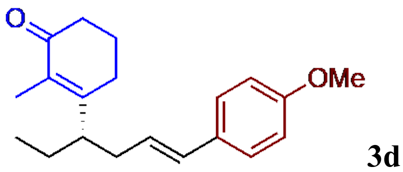

3d

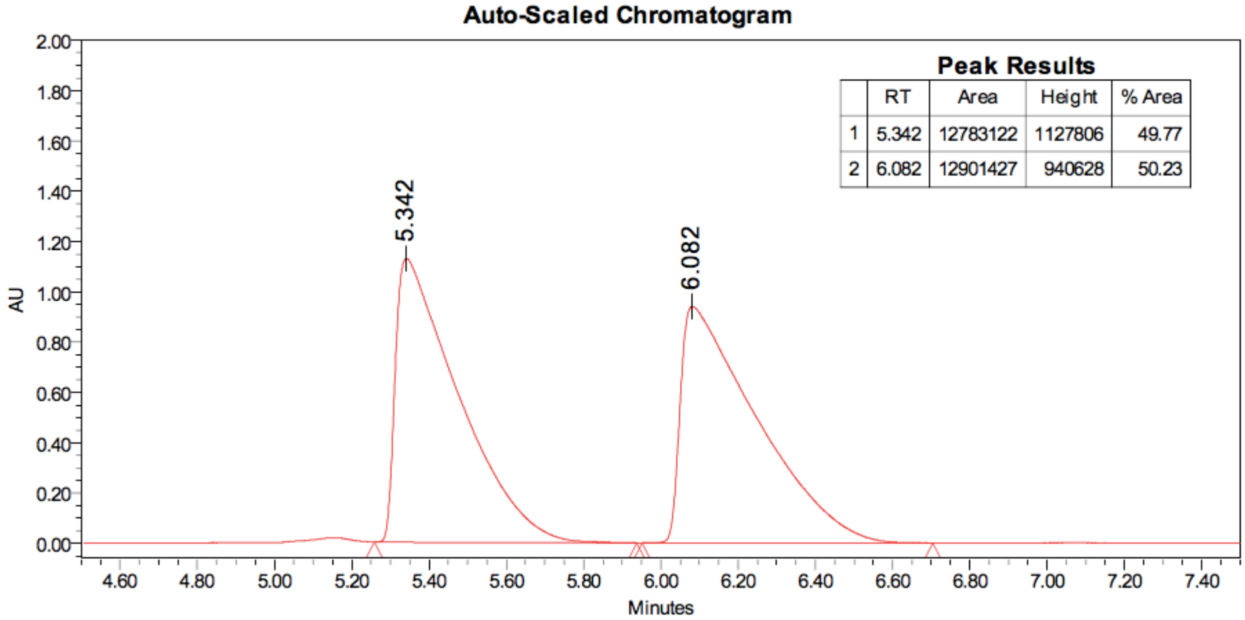

	RT	Area	Height	% Area
1	5.342	12783122	1127806	49.77
2	6.082	12901427	940628	50.23

Separation by SFC, Chiralcel® **3d**-Rac sample (Conditions): Cel1 column, 40.0 °C, *i*-PrOH: CO_2 = 3:97, 2 mL/min, 138 bar, 40 min; t_1 = 5.342 min, t_2 = 6.082 min.

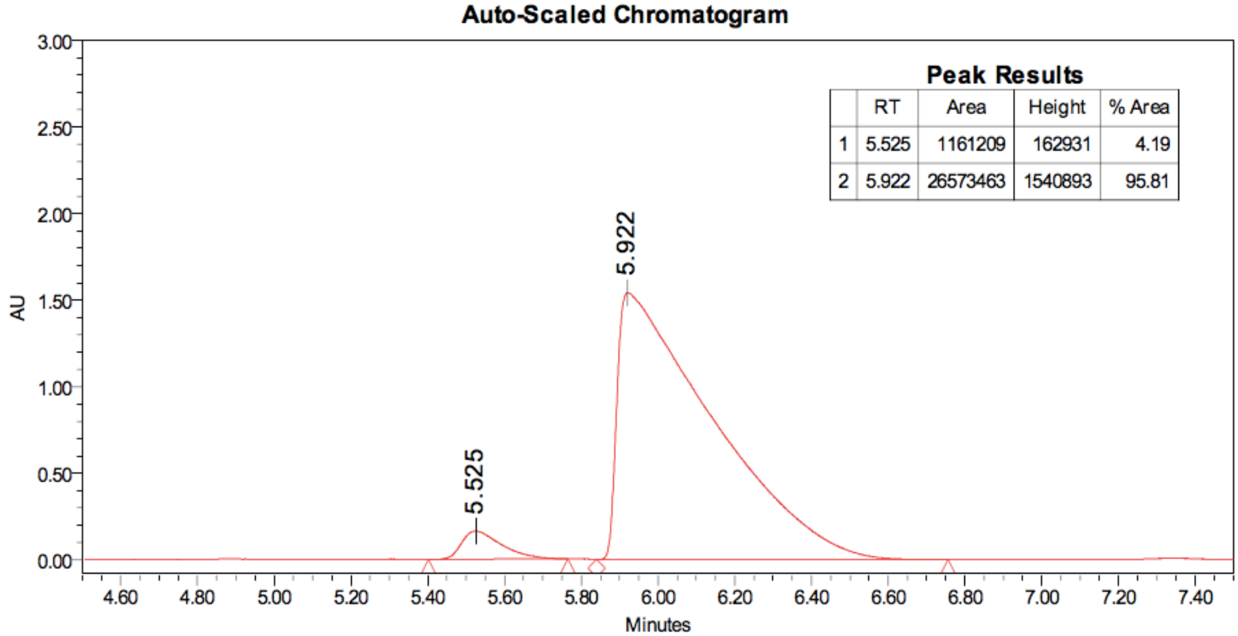

	RT	Area	Height	% Area
1	5.525	1161209	162931	4.19
2	5.922	26573463	1540893	95.81

3d sample (Conditions): Cel1 column, 40.0 °C, *i*-PrOH: CO_2 = 3:97, 2 mL/min, 138 bar, 40 min; t_1 = 5.525 min, t_2 = 5.922 min, er = 4:96.

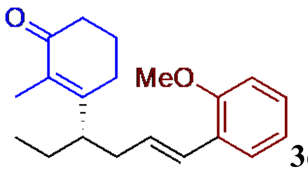

3e

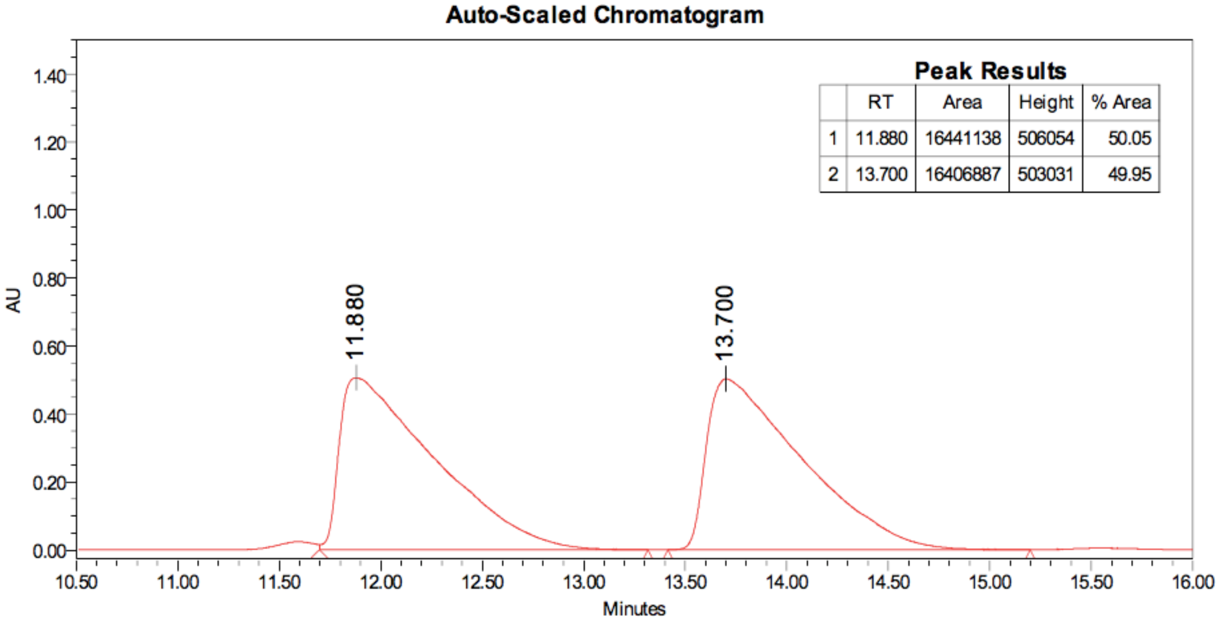

Separation by SFC, Chiralcel® **3e**-Rac sample (Conditions): Cel2 column, 40.0 °C, *i*-PrOH: CO_2 = 3:97, 2 mL/min, 138 bar, 40 min; t_1 = 11.880 min, t_2 = 13.700 min.

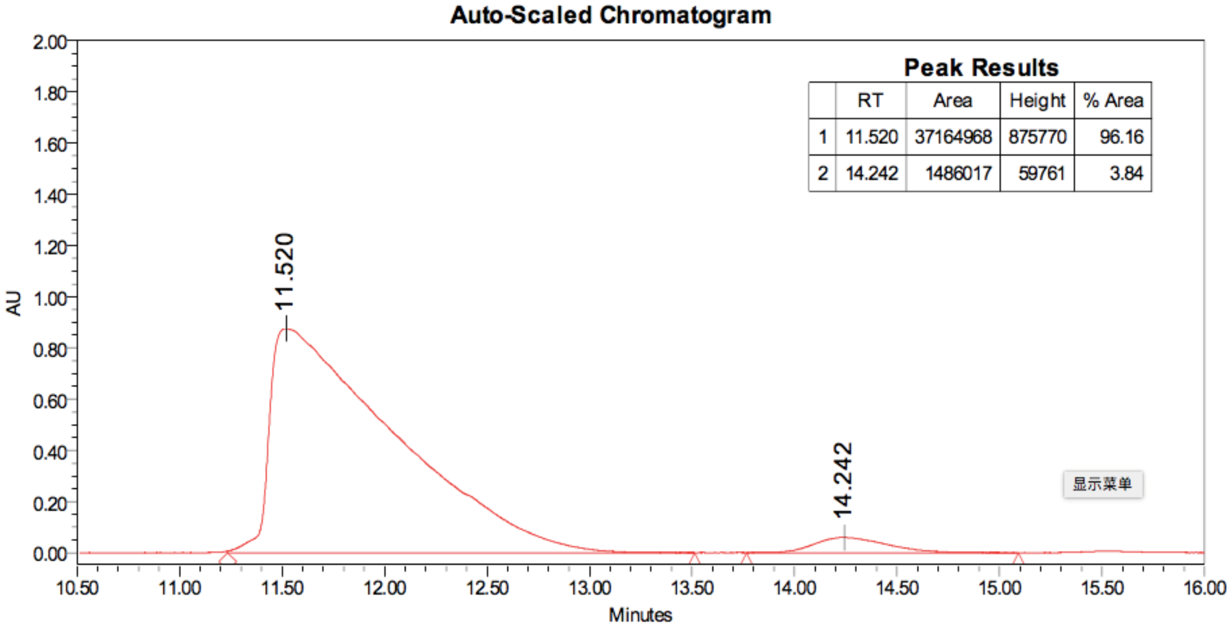

3e sample (Conditions): Cel2 column, 40.0 °C, *i*-PrOH: CO_2 = 3:97, 2 mL/min, 138 bar, 40 min; t_1 = 11.520 min, t_2 = 14.242 min, er = 96:4.

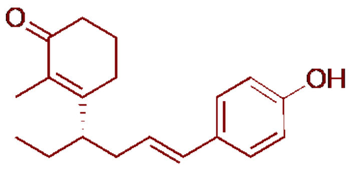

3f' er was got after methylation

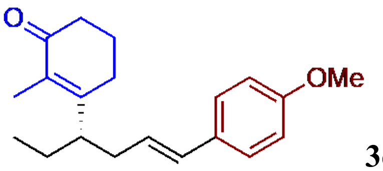

3d

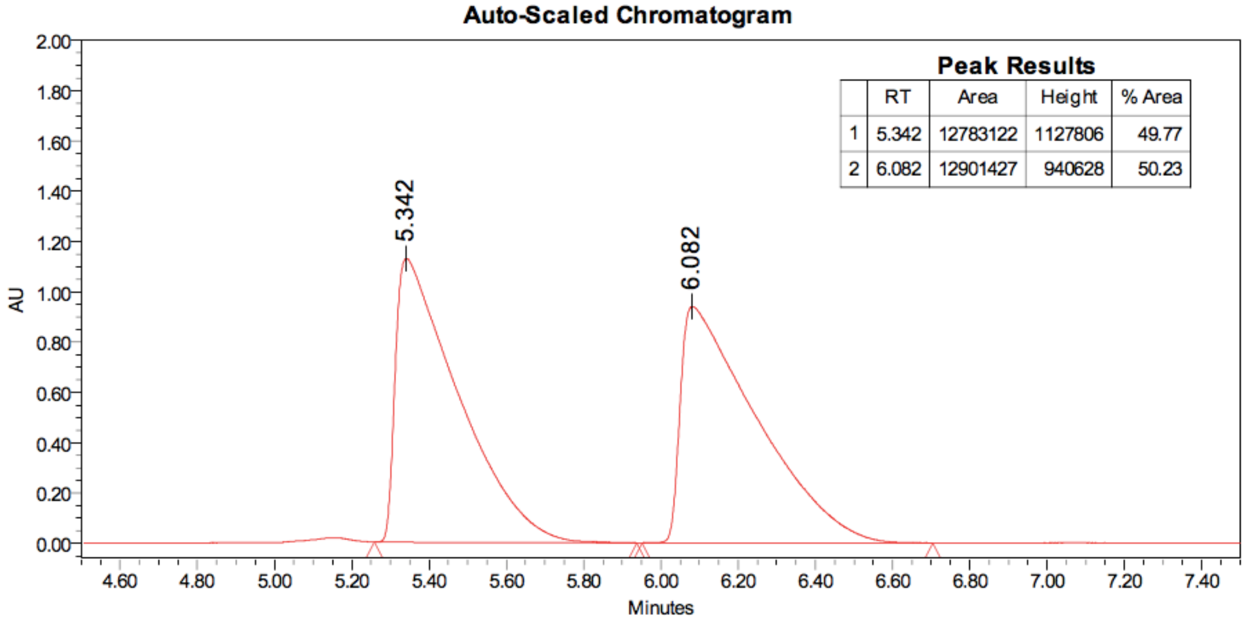

Separation by SFC, Chiralcel® **3d**-Rac sample (Conditions): Cel1 column, 40.0 °C, *i*-PrOH: CO_2 = 3:97, 2 mL/min, 138 bar, 40 min; t_1 = 5.342 min, t_2 = 6.082 min.

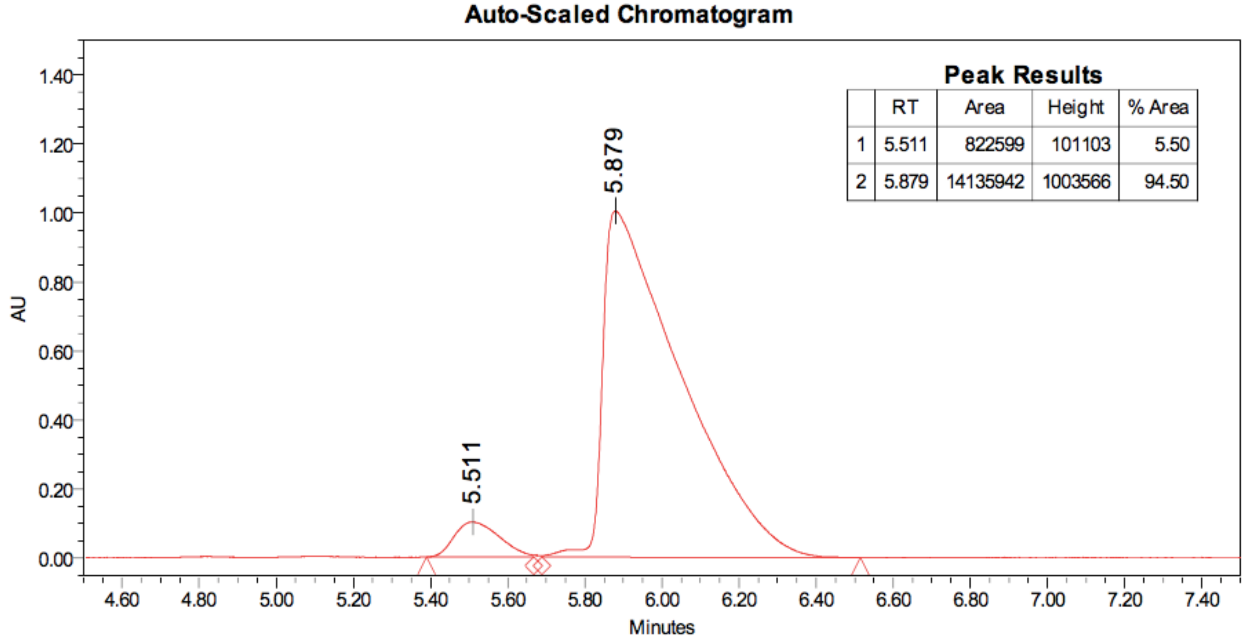

3d sample (Conditions): Cel1 column, 40.0 °C, *i*-PrOH: CO_2 = 3:97, 2 mL/min, 138 bar, 40 min; t_1 = 5.511min, t_2 = 5.879 min, er = 5.5:94.5.

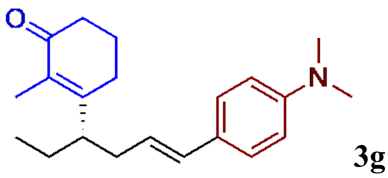

3g

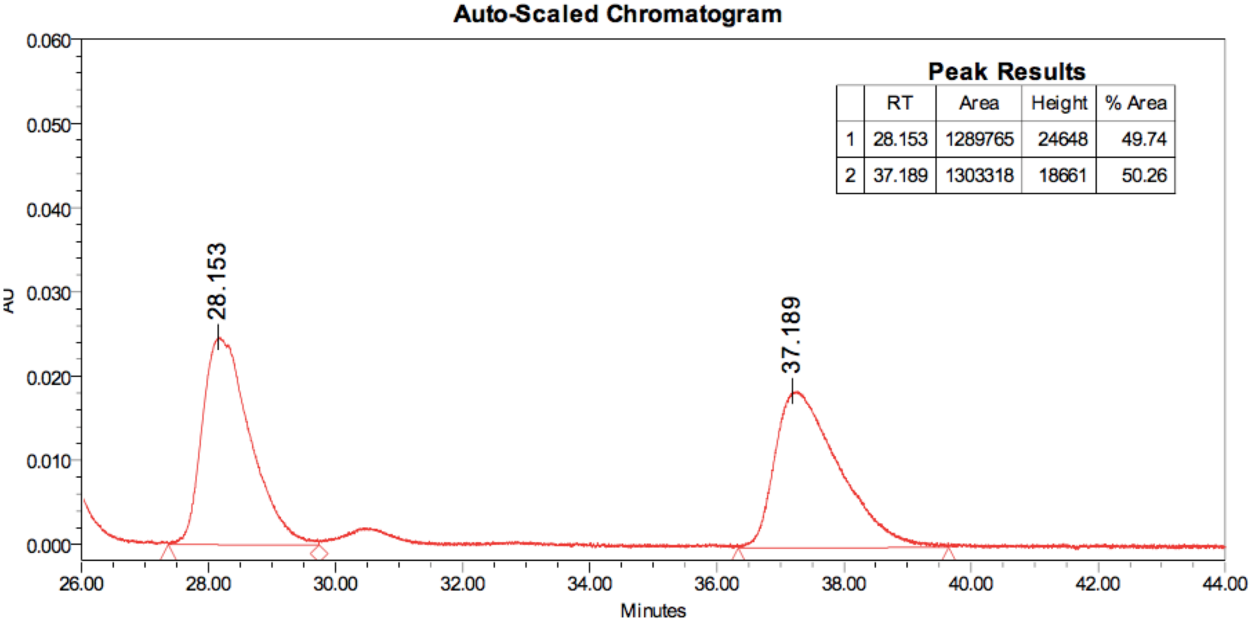

Separation by SFC, Chiralcel® **3g**-Rac sample (Conditions): Cel2 column, 40.0 °C, *i*-PrOH: CO_2 = 3:97, 2 mL/min, 138 bar, 40 min; t_1 = 28.153 min, t_2 = 37.189 min.

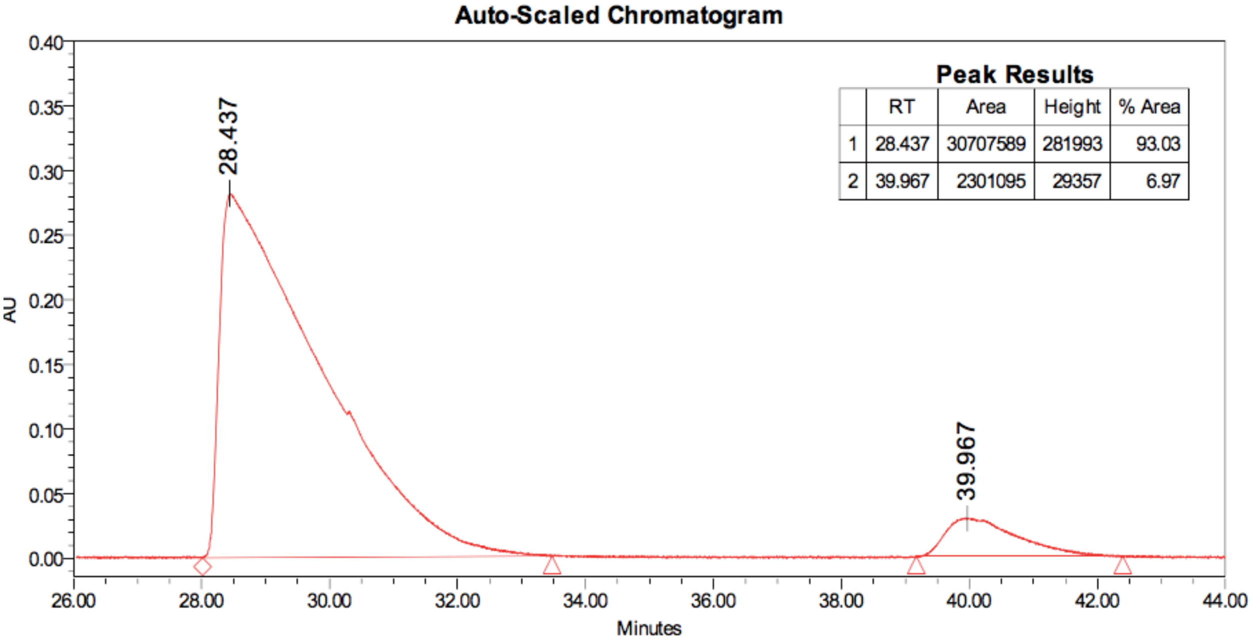

3g sample (Conditions): Cel2 column, 40.0 °C, *i*-PrOH: CO_2 = 3:97, 2 mL/min, 138 bar, 40 min; t_1 = 28.437 min, t_2 = 39.967 min, er = 93:7.

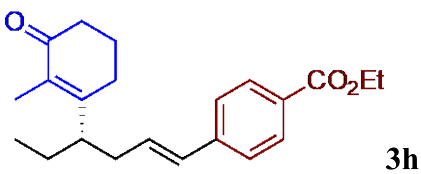

3h

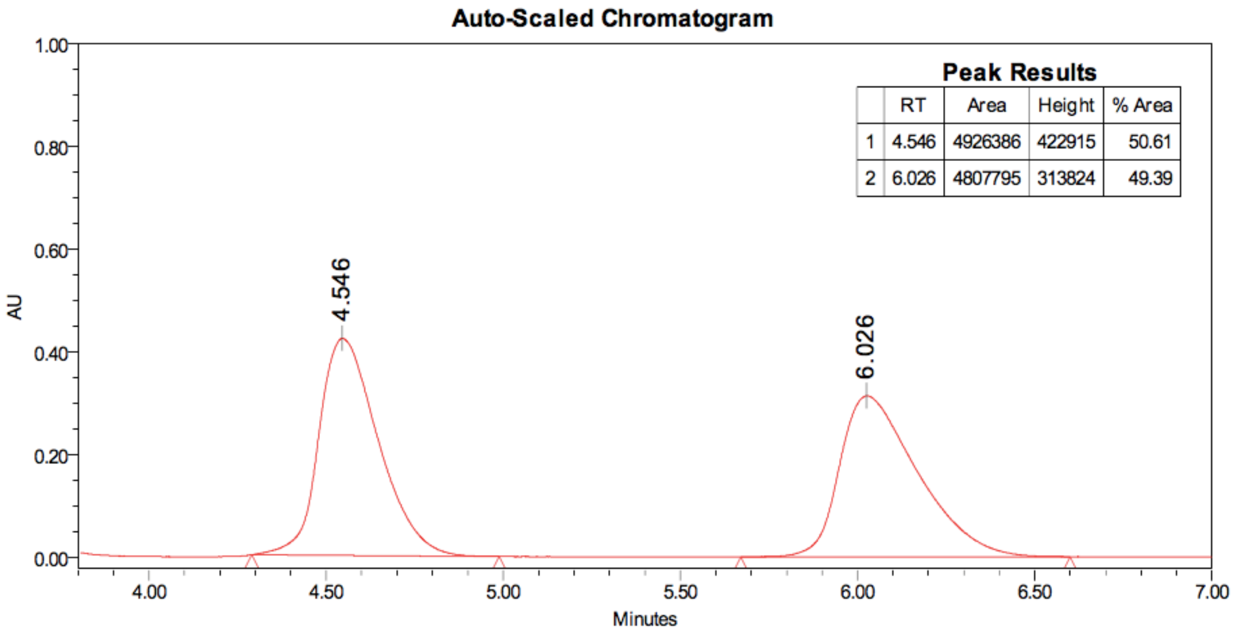

Peak Results

	RT	Area	Height	% Area
1	4.546	4926386	422915	50.61
2	6.026	4807795	313824	49.39

Separation by SFC, Chiralcel® **3h**-Rac sample (Conditions): Amy1 column, 40.0 °C, *i*-PrOH: CO_2 = 8:92, 2 mL/min, 138 bar, 20 min; t_1 = 4.546 min, t_2 = 6.026 min.

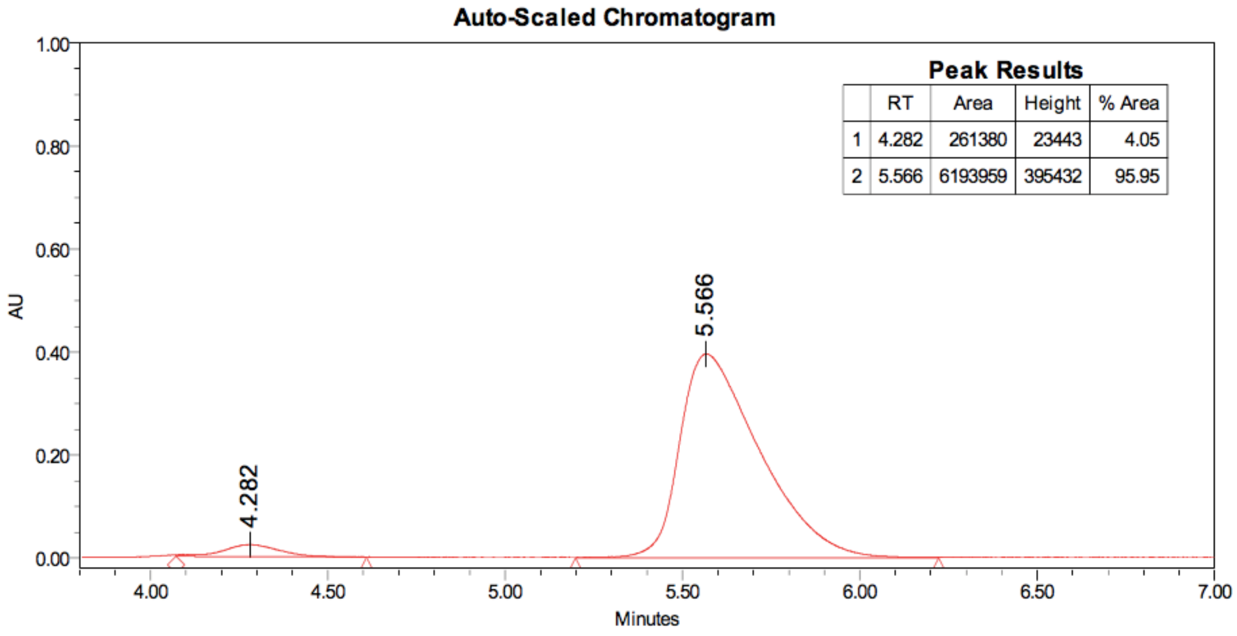

Peak Results

	RT	Area	Height	% Area
1	4.282	261380	23443	4.05
2	5.566	6193959	395432	95.95

3h sample (Conditions): Amy1 column, 40.0 °C, *i*-PrOH: CO_2 = 8:92, 2 mL/min, 138 bar, 20 min; t_1 = 4.282 min, t_2 = 5.566 min, er = 4:96.

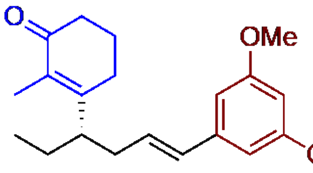

3i

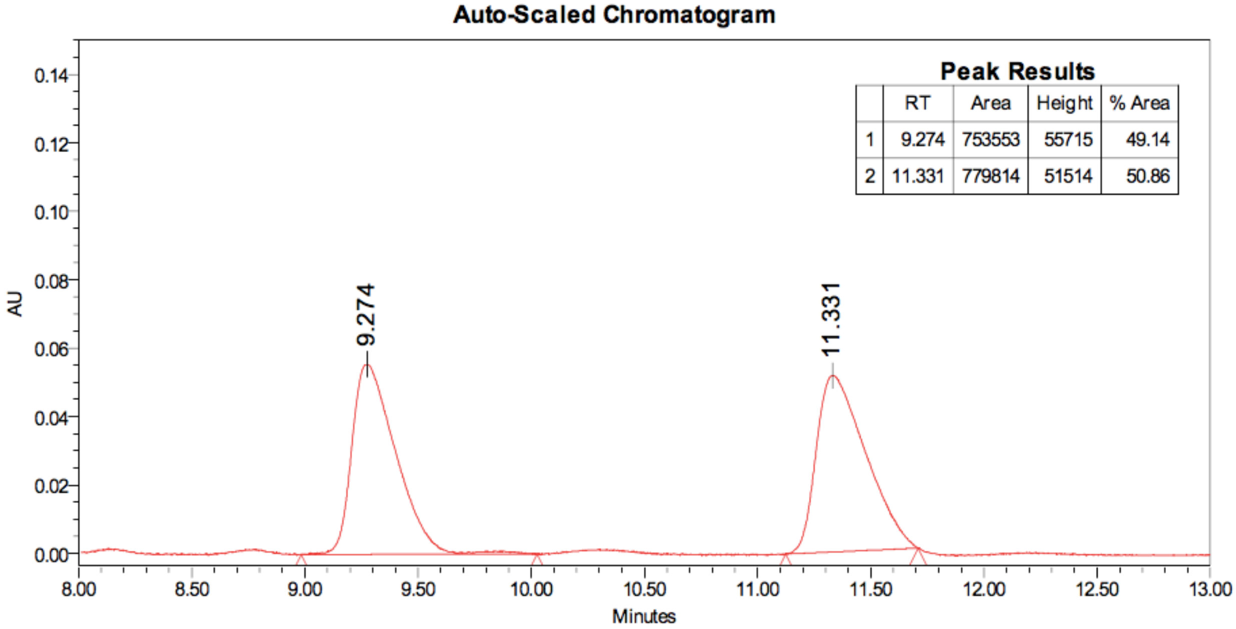

	RT	Area	Height	% Area
1	9.274	753553	55715	49.14
2	11.331	779814	51514	50.86

Separation by SFC, Chiralcel® **3i**-Rac sample (Conditions): Cel1 column, 40.0 °C, MeOH: CO_2 = 2:98, 2 mL/min, 138 bar, 40 min; t_1 = 9.274 min, t_2 = 11.331 min.

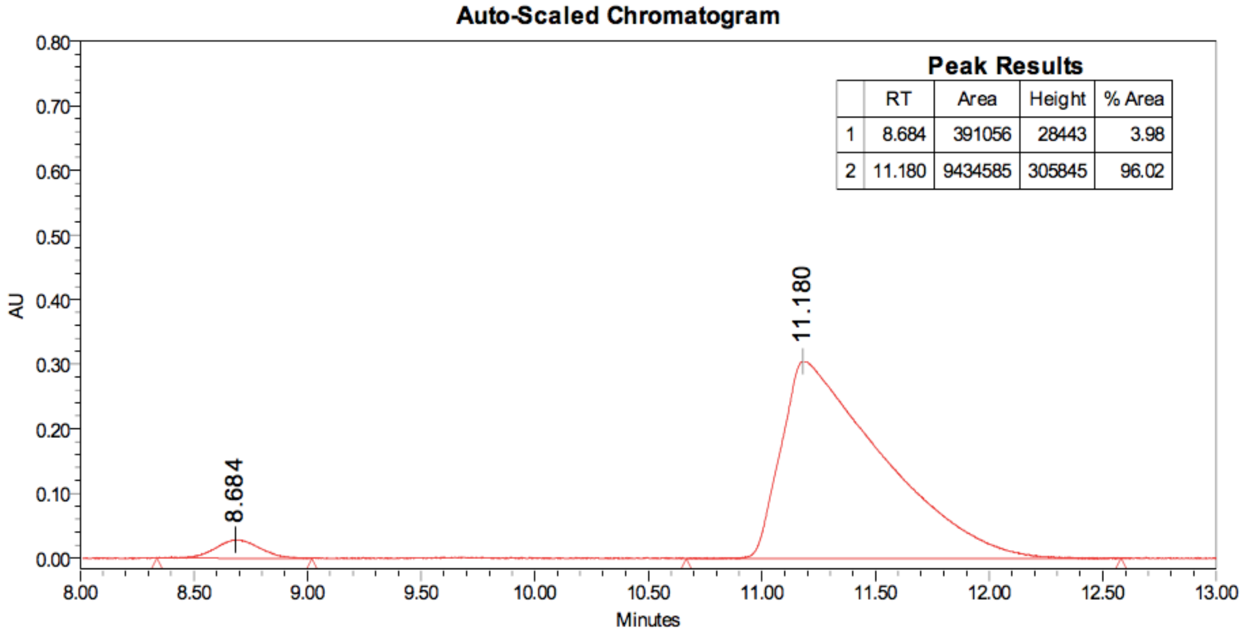

	RT	Area	Height	% Area
1	8.684	391056	28443	3.98
2	11.180	9434585	305845	96.02

3i sample (Conditions): Cel1 column, 40.0 °C, MeOH: CO_2 = 2:98, 2 mL/min, 138 bar, 40 min; t_1 = 8.684 min, t_2 = 11.180 min, er = 4:96.

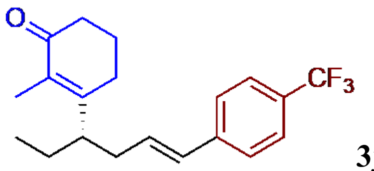

3j

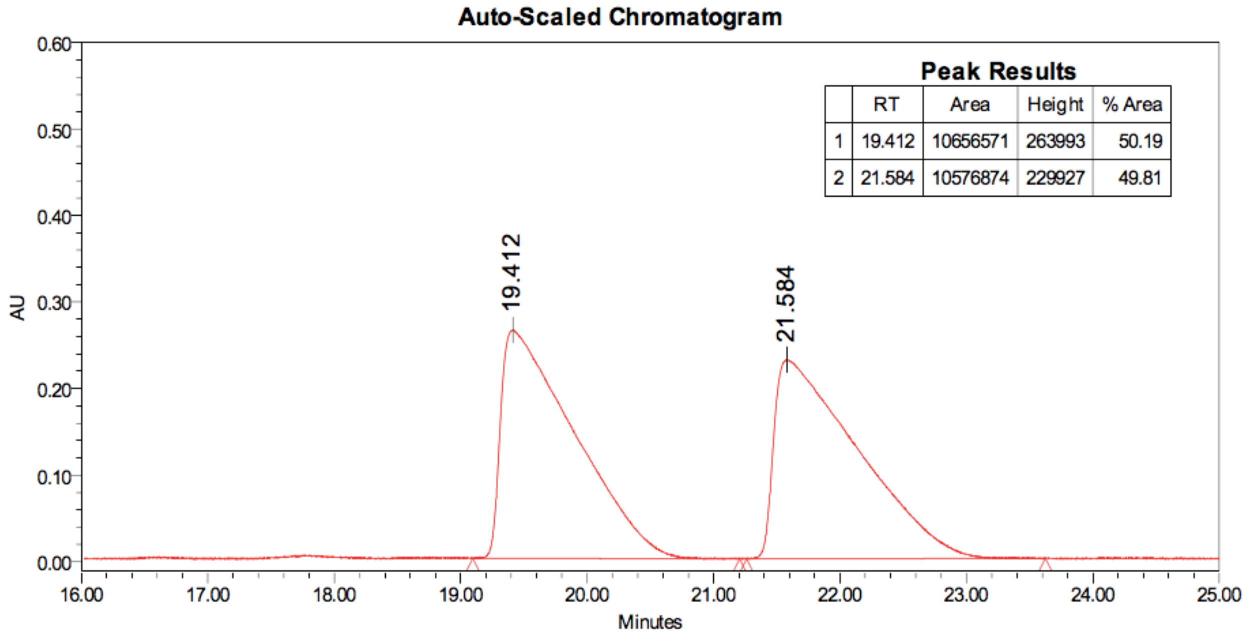

Separation by SFC, Chiralcel® **3j**-Rac sample (Conditions): Cel2 column, 40.0 °C, MeOH: CO_2 = 1:99, 1 mL/min, 138 bar, 40 min; t_1 = 19.412 min, t_2 = 21.584 min.

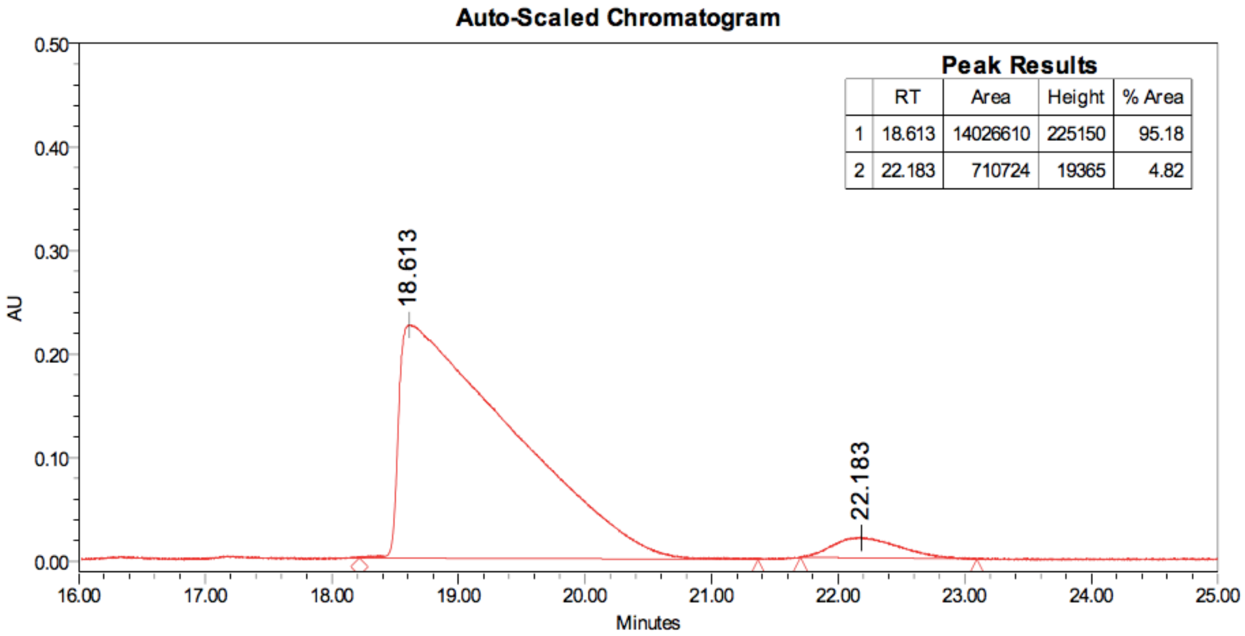

3j sample (Conditions): Cel2 column, 40.0 °C, MeOH: CO_2 = 1:99, 1 mL/min, 138 bar, 40 min; t_1 = 18.613 min, t_2 = 22.183 min, er = 95:5.

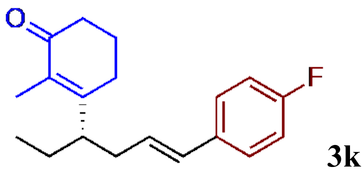

3k

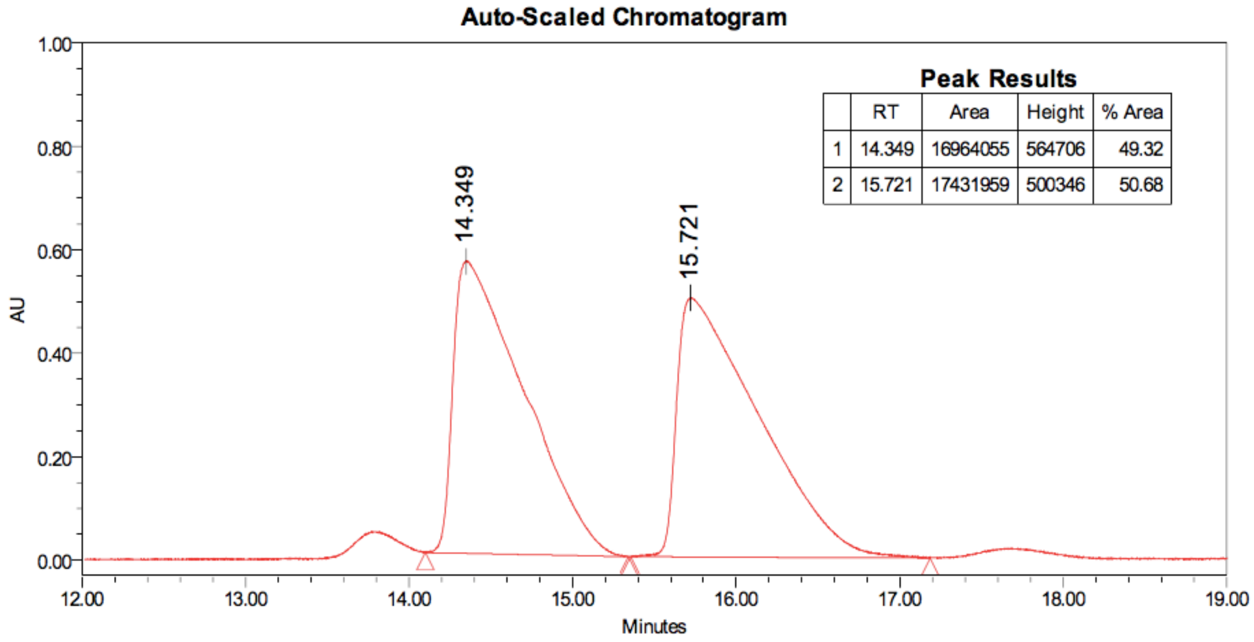

Separation by SFC, Chiralcel® **3k**-Rac sample (Conditions): Cel2 column, 40.0 °C, MeOH: CO_2 = 1:99, 2 mL/min, 138 bar, 40 min; t_1 = 14.349 min, t_2 = 15.721 min.

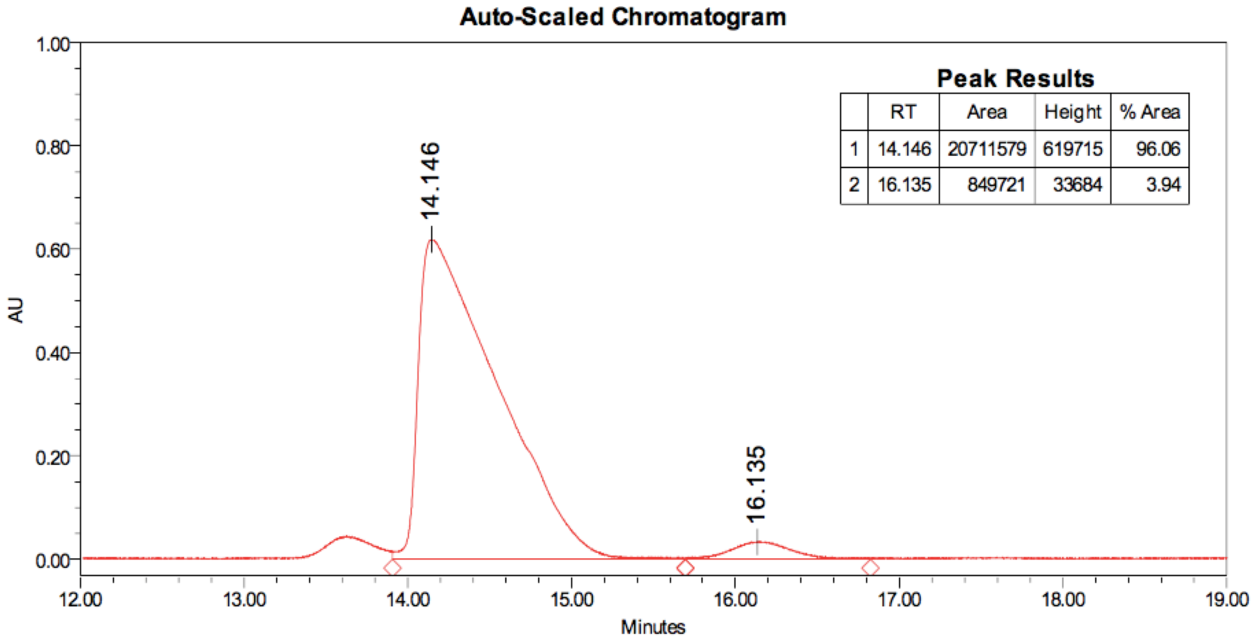

3k sample (Conditions): Cel2 column, 40.0 °C, MeOH: CO_2 = 1:99, 2 mL/min, 138 bar, 40 min; t_1 = 14.146 min, t_2 = 16.135 min, er = 96:4.

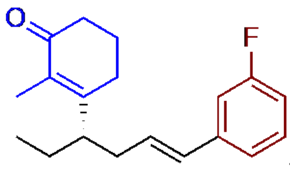

3l

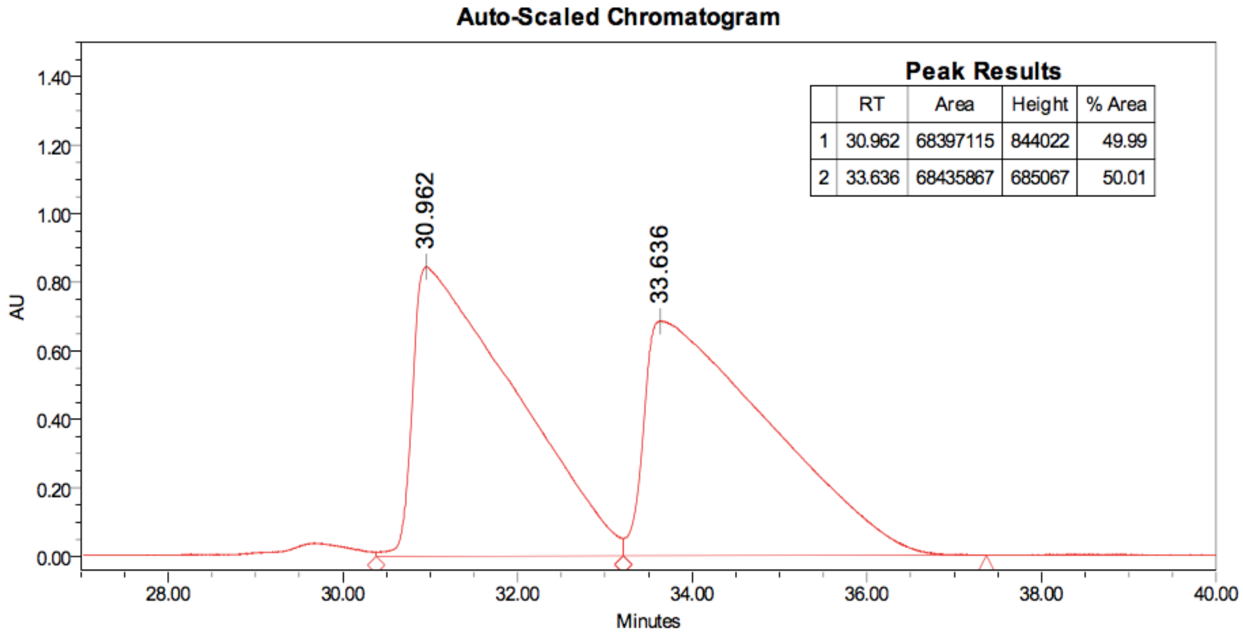

Separation by SFC, Chiralcel® **3l**-Rac sample (Conditions): Cel2 column, 40.0 °C, MeOH: CO_2 = 1:99, 1 mL/min, 138 bar, 40 min; t_1 = 30.962 min, t_2 = 33.636 min.

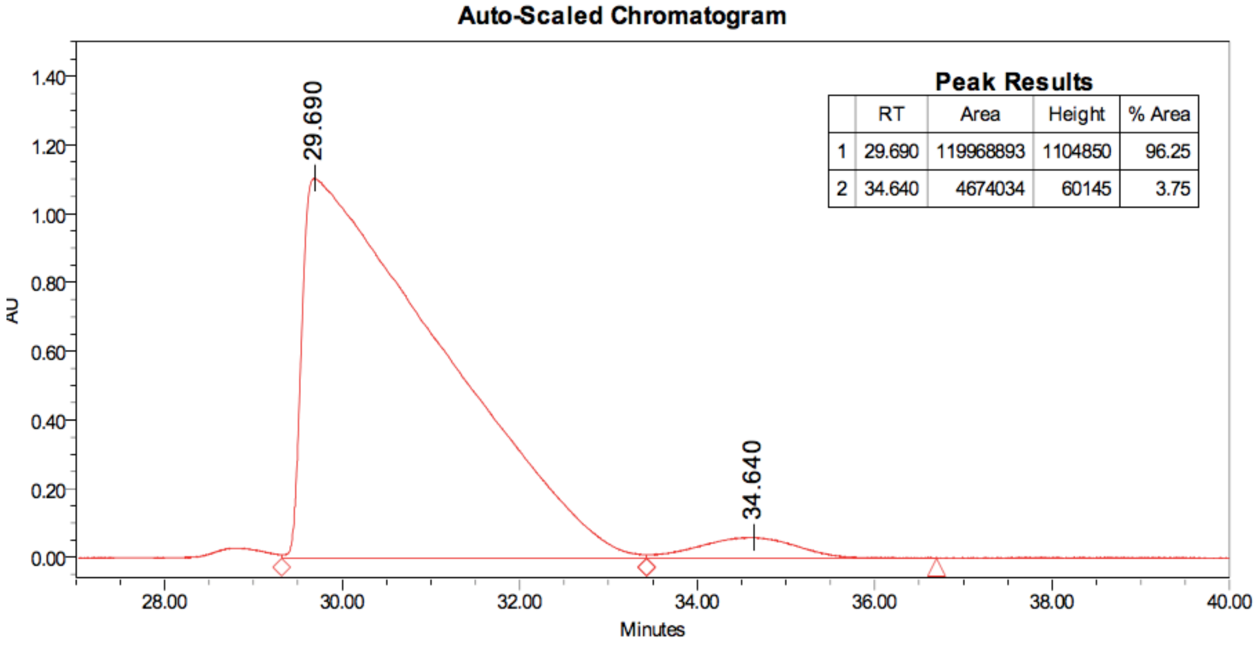

3l sample (Conditions): Cel2 column, 40.0 °C, MeOH: CO_2 = 1:99, 1 mL/min, 138 bar, 40 min; t_1 = 29.690 min, t_2 = 34.640 min, er = 96:4.

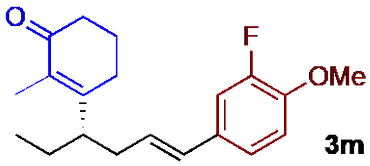

3m

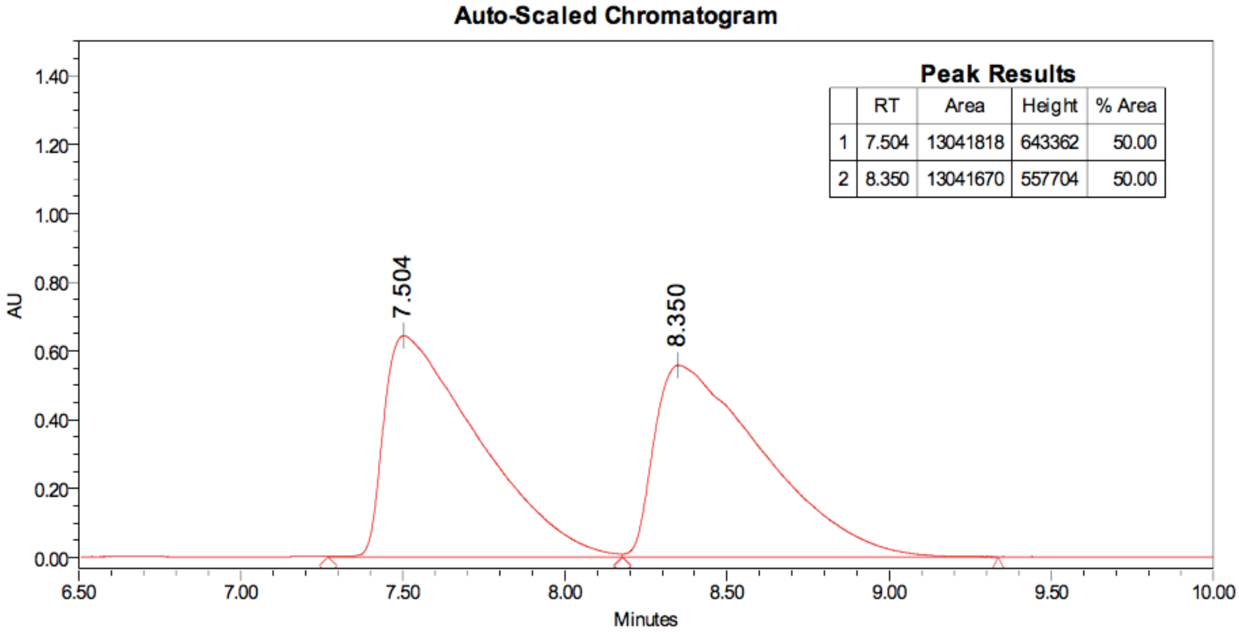

	RT	Area	Height	% Area
1	7.504	13041818	643362	50.00
2	8.350	13041670	557704	50.00

Separation by SFC, Chiralcel® **3m**-Rac sample (Conditions): Amy1 column, 40.0 °C, *i*-PrOH: CO_2 = 3:97, 2 mL/min, 138 bar, 40 min; t_1 = 7.504 min, t_2 = 8.350 min.

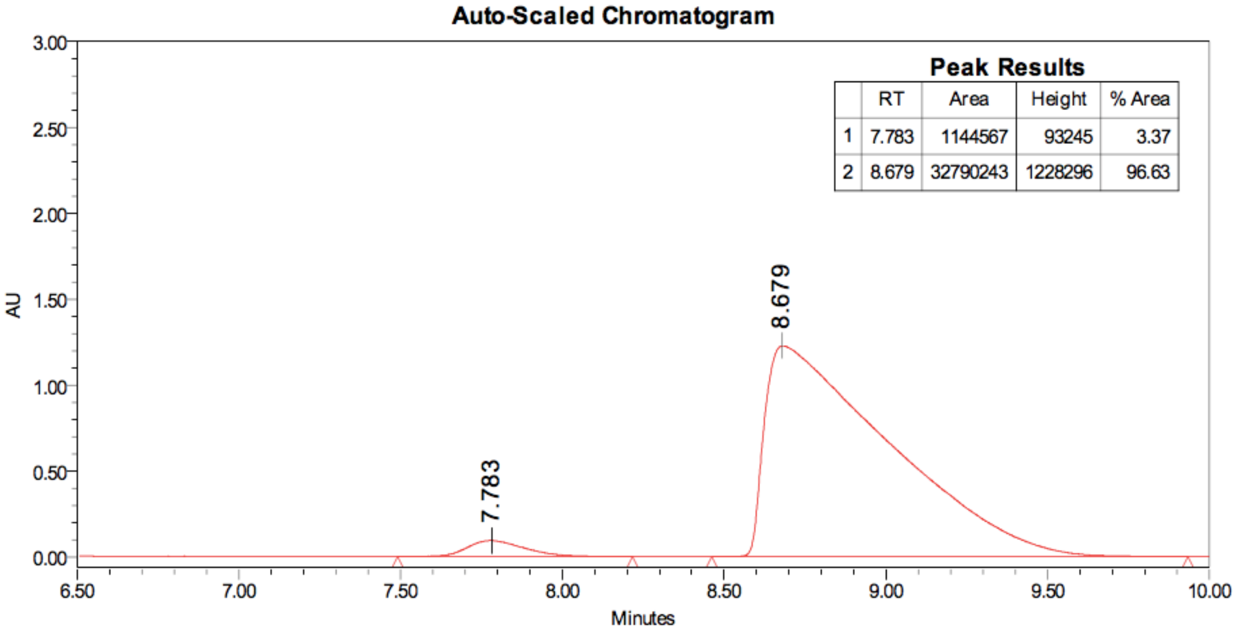

	RT	Area	Height	% Area
1	7.783	1144567	93245	3.37
2	8.679	32790243	1228296	96.63

3m sample (Conditions): Amy1 column, 40.0 °C, *i*-PrOH: CO_2 = 3:97, 2 mL/min, 138 bar, 40 min; t_1 = 7.783 min, t_2 = 8.679 min, er = 3.5:96.5.

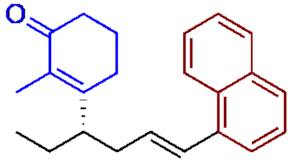

3n

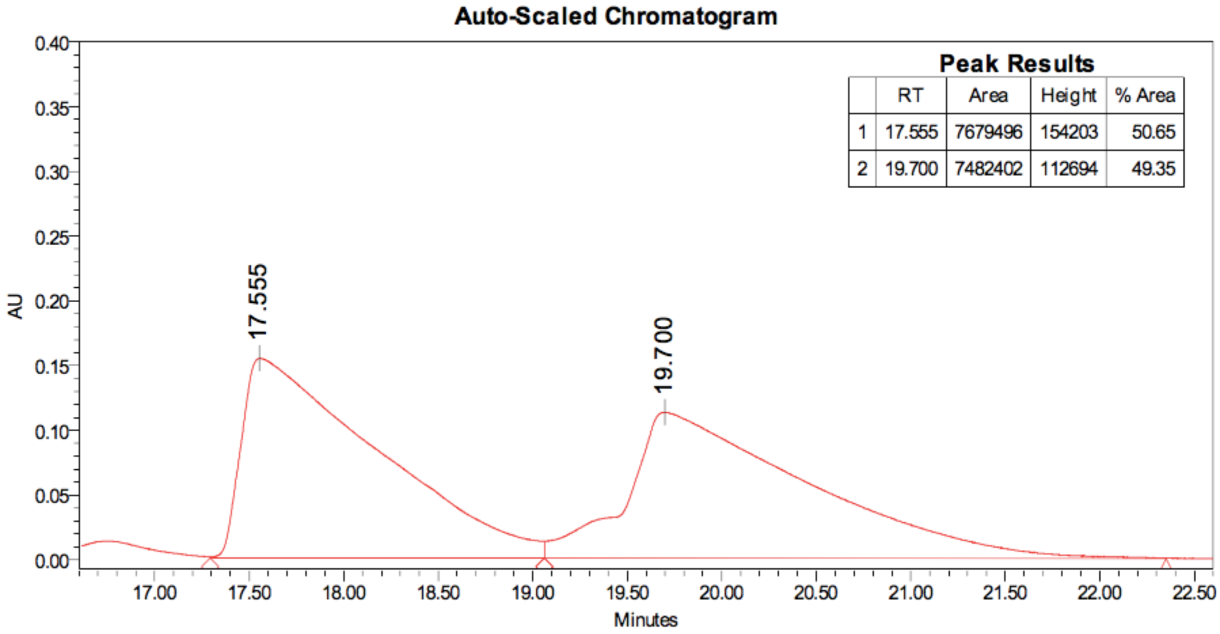

Separation by SFC, Chiralcel® **3n**-Rac sample (Conditions): Cel1 column, 40.0 °C, MeOH: CO_2 = 2:98, 2 mL/min, 138 bar, 40 min; t_1 = 17.555 min, t_2 = 19.700 min.

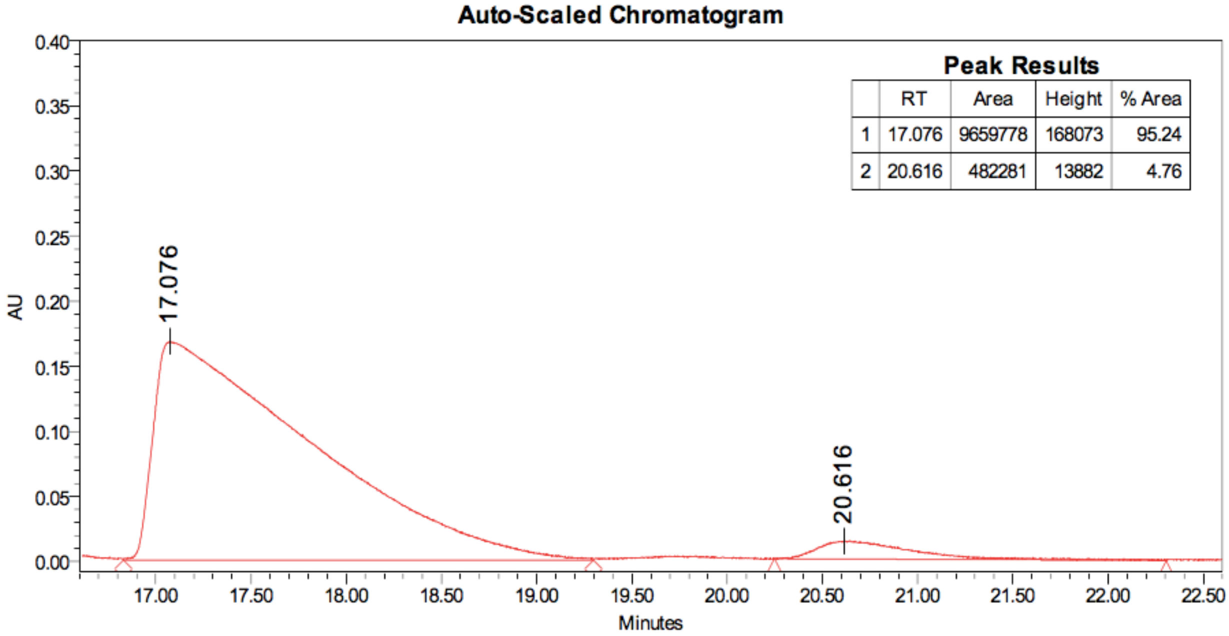

3n sample (Conditions): Cel1 column, 40.0 °C, MeOH: CO_2 = 2:98, 2 mL/min, 138 bar, 40 min; t_1 = 17.076 min, t_2 = 20.616 min, er = 95:5.

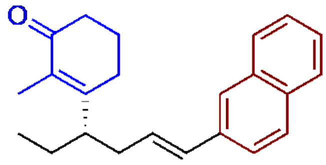

3o

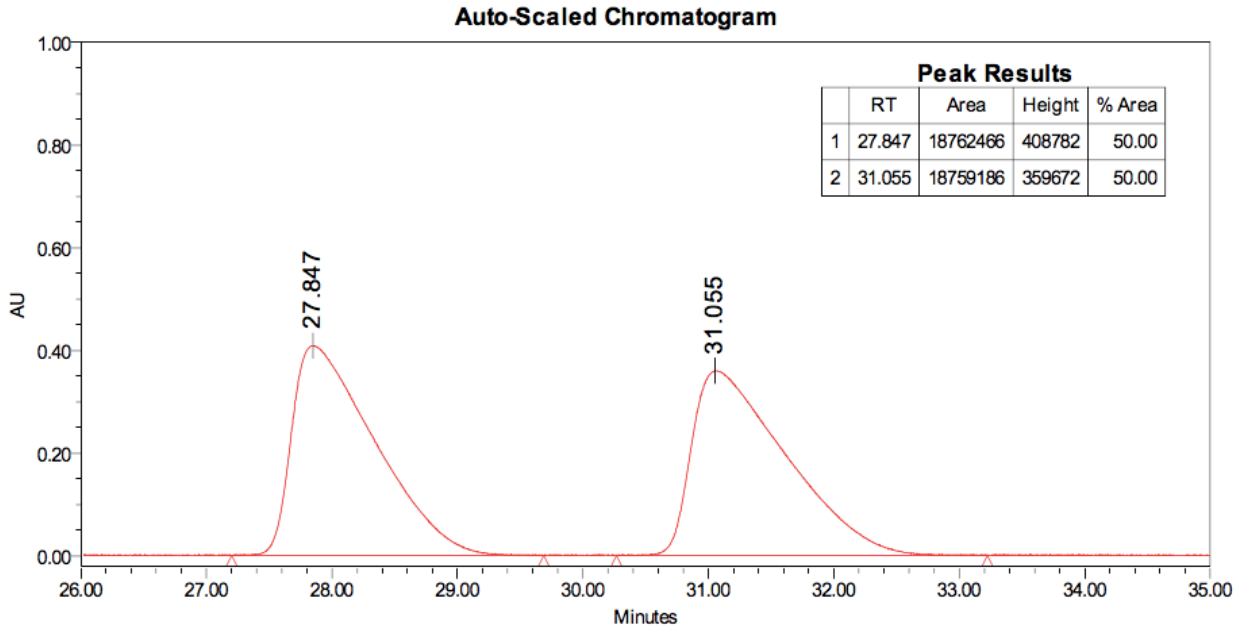

	RT	Area	Height	% Area
1	27.847	18762466	408782	50.00
2	31.055	18759186	359672	50.00

Separation by SFC, Chiralcel® **3o**-Rac sample (Conditions): Cel2 column, 40.0 °C, MeOH: CO_2 = 2:98, 2 mL/min, 138 bar, 40 min; t_1 = 27.847 min, t_2 = 31.055 min.

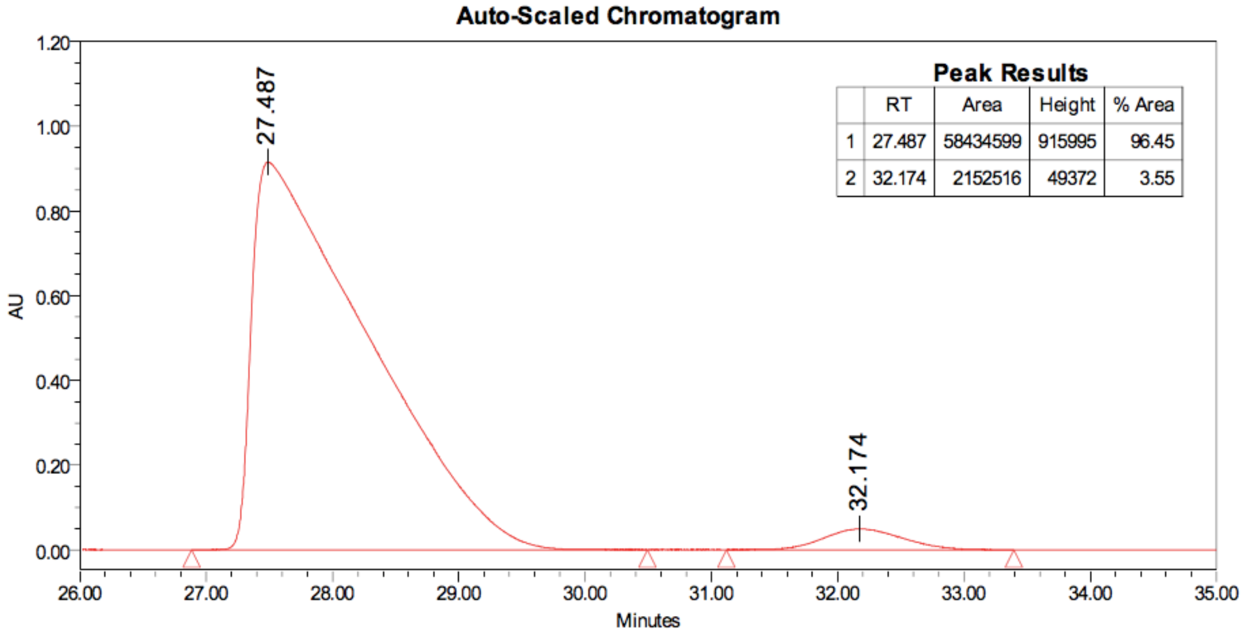

	RT	Area	Height	% Area
1	27.487	58434599	915995	96.45
2	32.174	2152516	49372	3.55

3o sample (Conditions): Cel2 column, 40.0 °C, MeOH: CO_2 = 2:98, 2 mL/min, 138 bar, 40 min; t_1 = 27.487 min, t_2 = 32.174 min, er = 96:4.

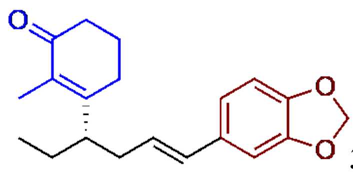

3p

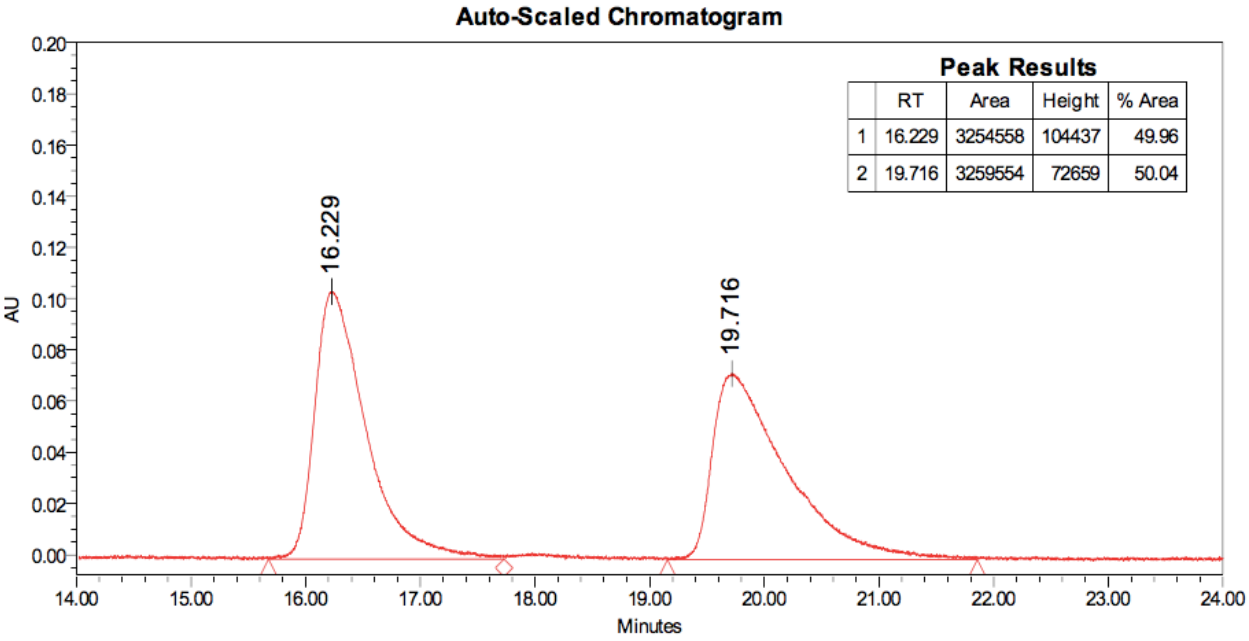

Separation by SFC, Chiralcel® **3p**-Rac sample (Conditions): Cel2 column, 40.0 °C, *i*-PrOH: CO_2 = 3:97, 2 mL/min, 138 bar, 40 min; t_1 = 16.229 min, t_2 = 19.716 min.

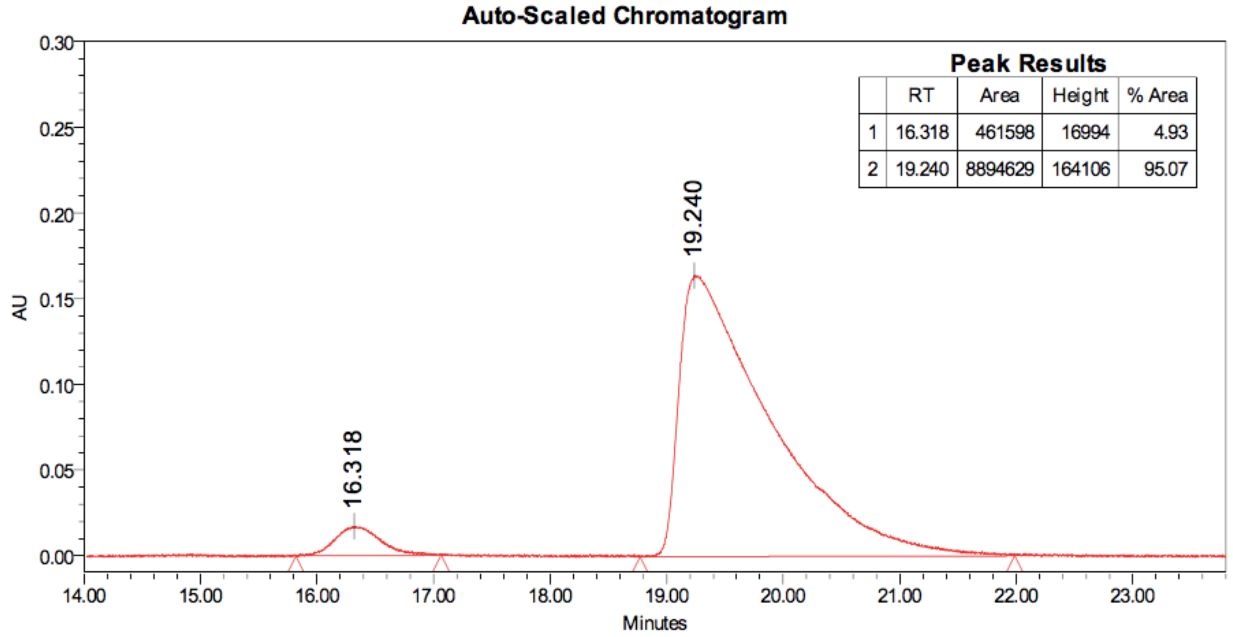

3p sample (Conditions): Cel2 column, 40.0 °C, *i*-PrOH: CO_2 = 3:97, 2 mL/min, 138 bar, 40 min; t_1 = 16.318 min, t_2 = 19.240 min, er = 5:95.

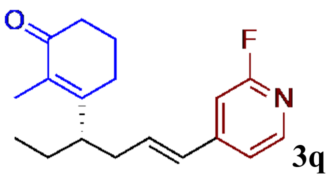

3q

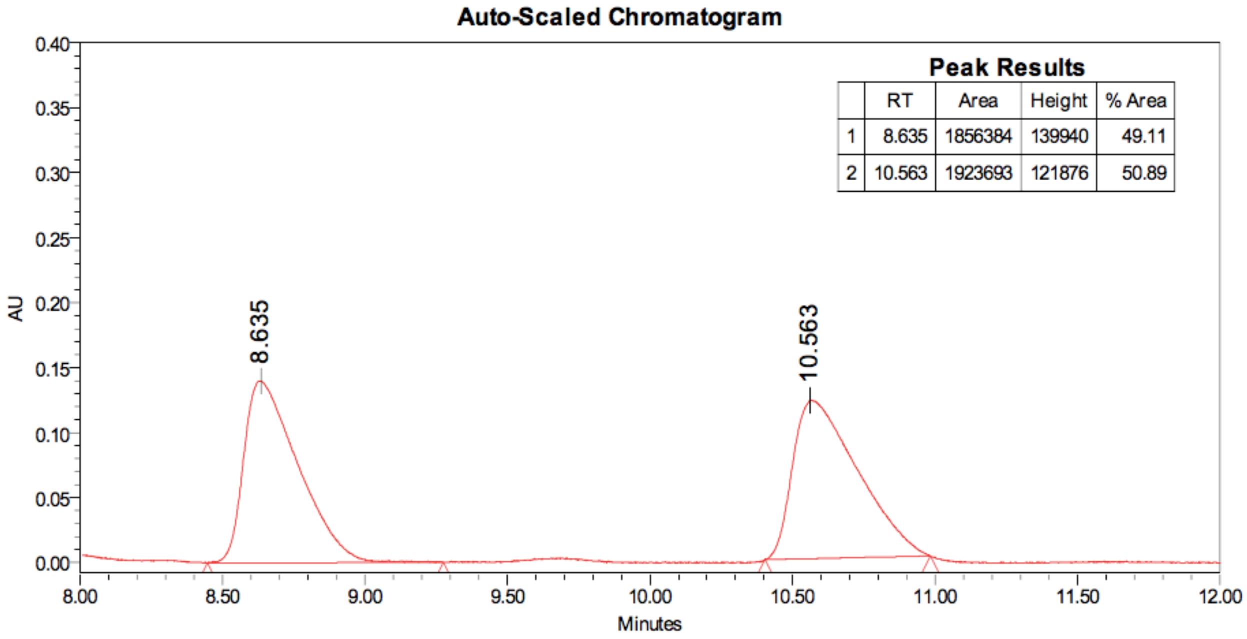

	RT	Area	Height	% Area
1	8.635	1856384	139940	49.11
2	10.563	1923693	121876	50.89

Separation by SFC, Chiralcel® **3q**-Rac sample (Conditions): Cel2 column, 40.0 °C, *i*-PrOH: CO_2 = 5:95, 2 mL/min, 138 bar, 40 min; t_1 = 8.635 min, t_2 = 10.563 min.

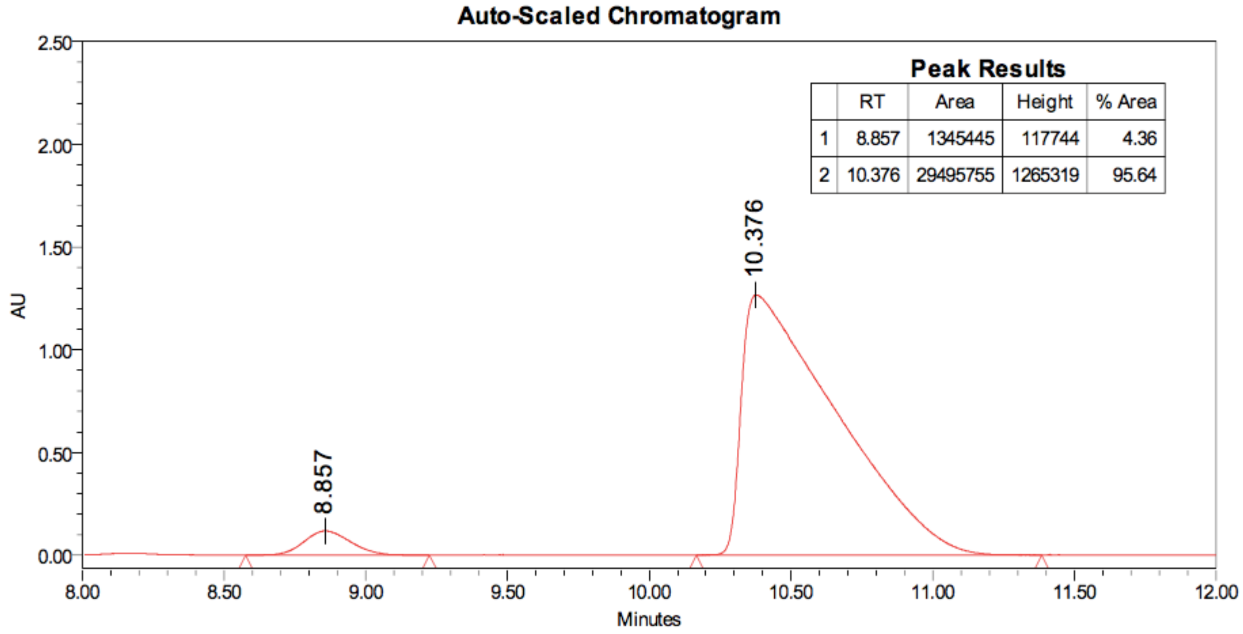

	RT	Area	Height	% Area
1	8.857	1345445	117744	4.36
2	10.376	29495755	1265319	95.64

3q sample (Conditions): Cel2 column, 40.0 °C, *i*-PrOH: CO_2 = 5:95, 2 mL/min, 138 bar, 40 min; t_1 = 8.857 min, t_2 = 10.376 min, er = 4.5:95.5.

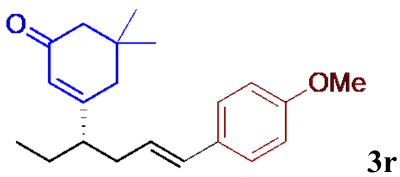

3r

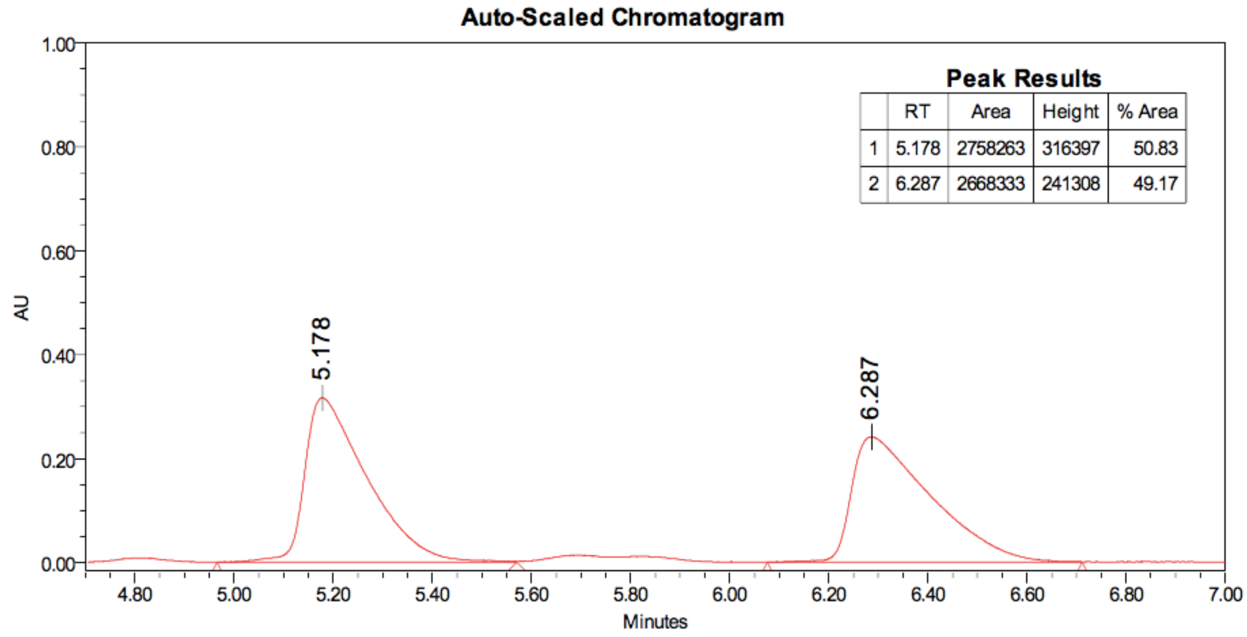

Separation by SFC, Chiralcel® **3r**-Rac sample (Conditions): Cel1 column, 40.0 °C, *i*-PrOH: CO_2 = 3:97, 2 mL/min, 138 bar, 40 min; t_1 = 5.178 min, t_2 = 6.287 min.

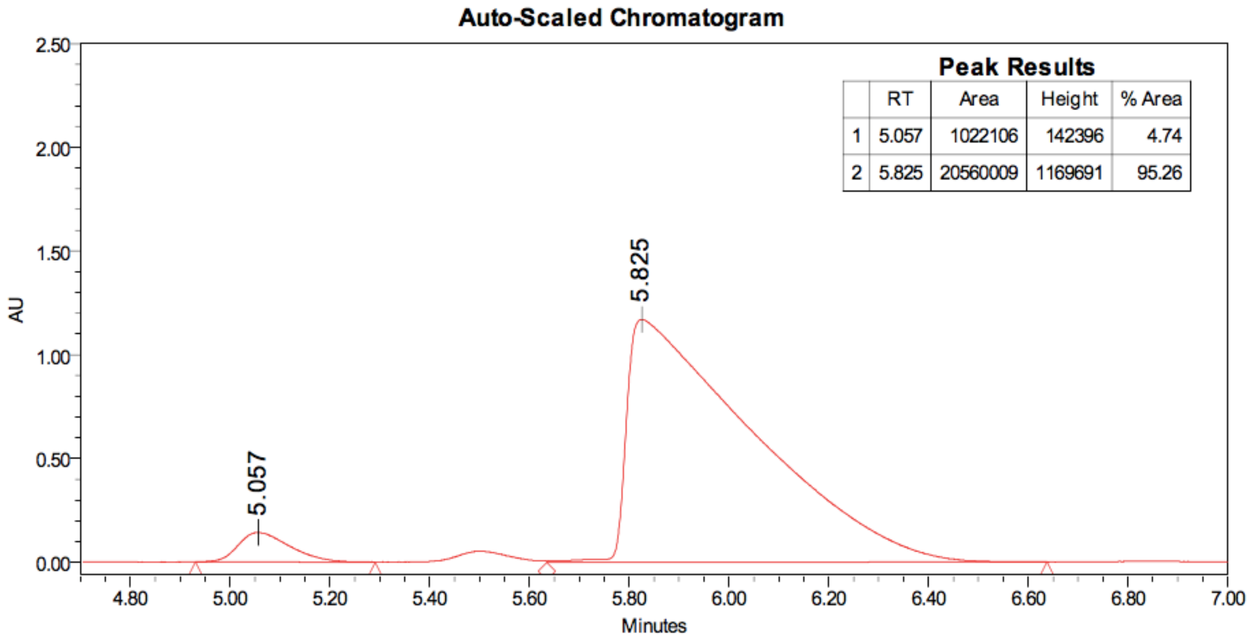

3r sample (Conditions): Cel1 column, 40.0 °C, *i*-PrOH: CO_2 = 3:97, 2 mL/min, 138 bar, 40 min; t_1 = 5.057 min, t_2 = 5.825 min, er = 5:95.

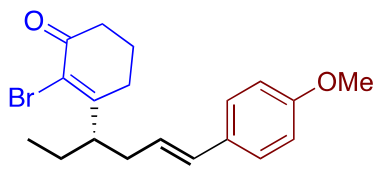

3s

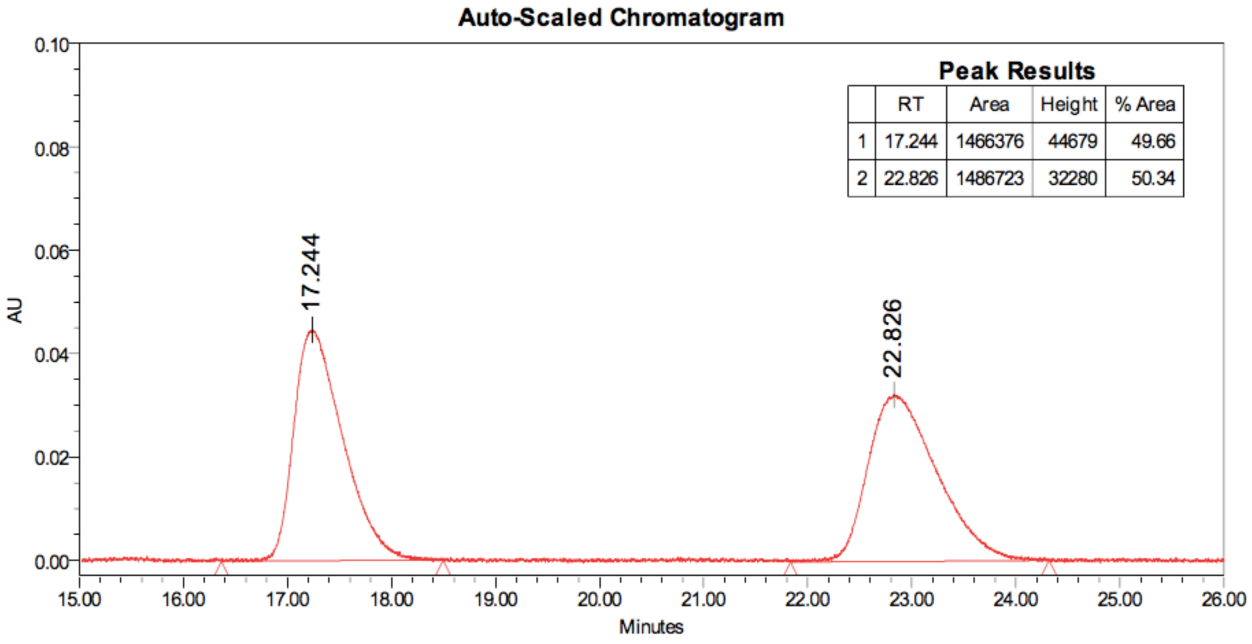

Separation by SFC, Chiralcel® **3s**-Rac sample (Conditions): Amy1 column, 40.0 °C, *i*-PrOH: CO_2 = 3:97, 2 mL/min, 138 bar, 40 min; t_1 = 17.244 min, t_2 = 22.826 min.

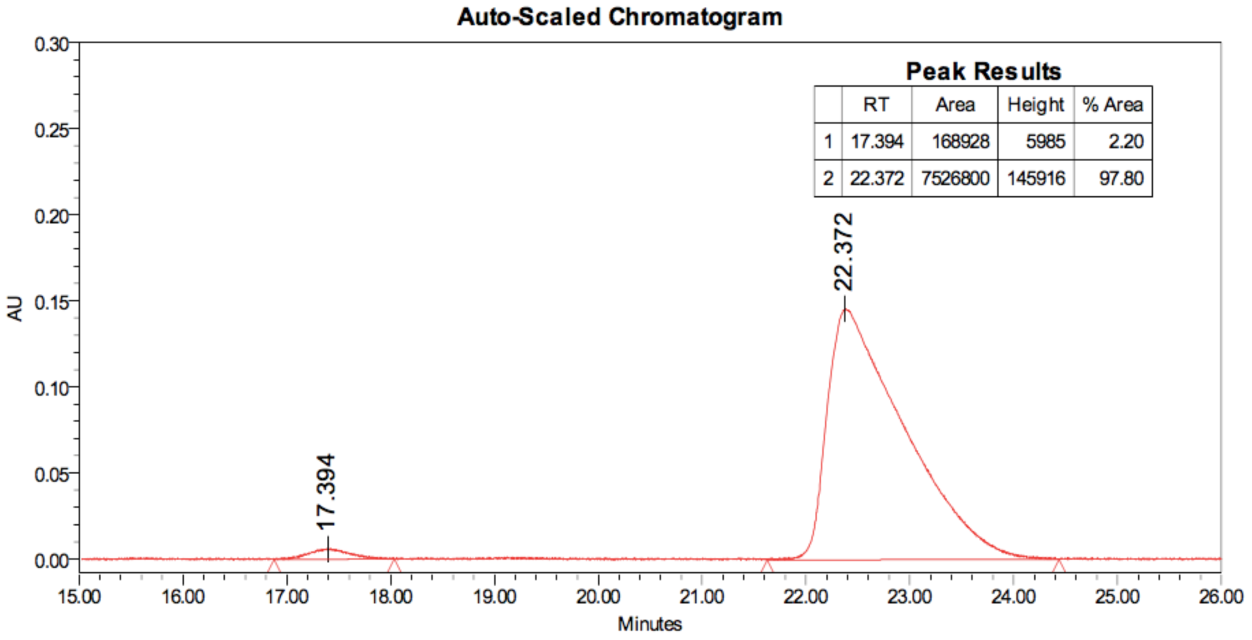

3s sample (Conditions): Amy1 column, 40.0 °C, *i*-PrOH: CO_2 = 3:97, 2 mL/min, 138 bar, 40 min; t_1 = 17.394 min, t_2 = 22.826 min, er = 2:98.

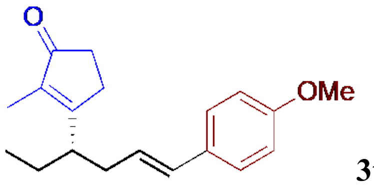

3t

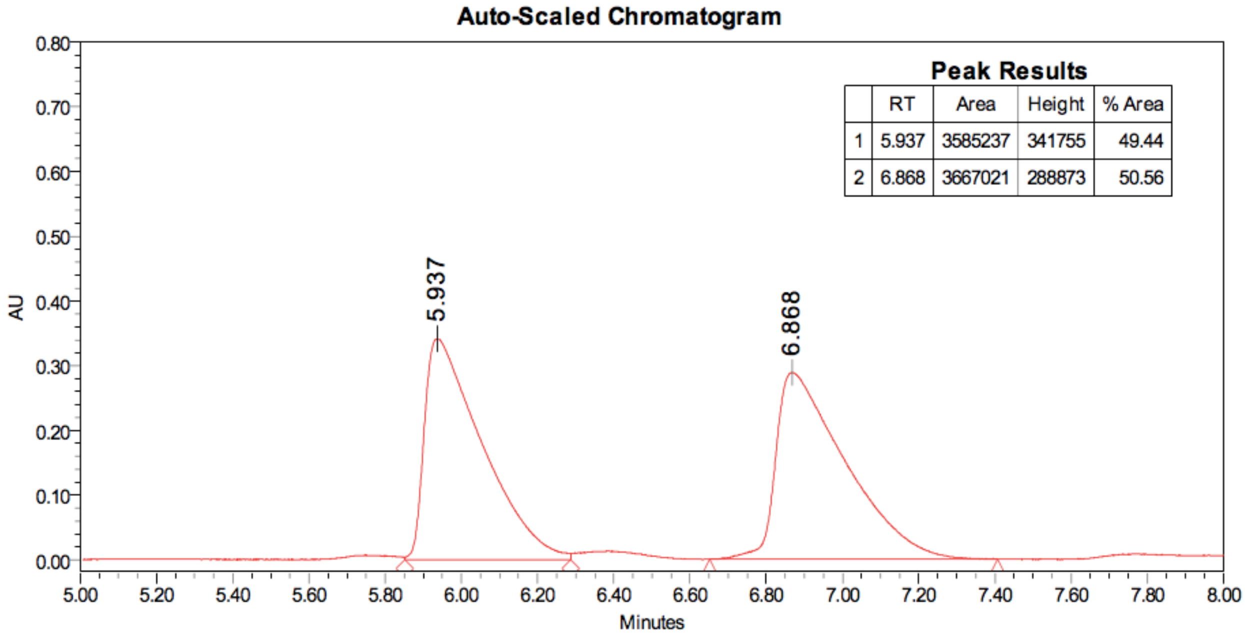

Separation by SFC, Chiralcel® **3t**-Rac sample (Conditions): Cel1 column, 40.0 °C, *i*-PrOH: CO_2 = 3:97, 2 mL/min, 138 bar, 40 min; t_1 = 5.937 min, t_2 = 6.868 min.

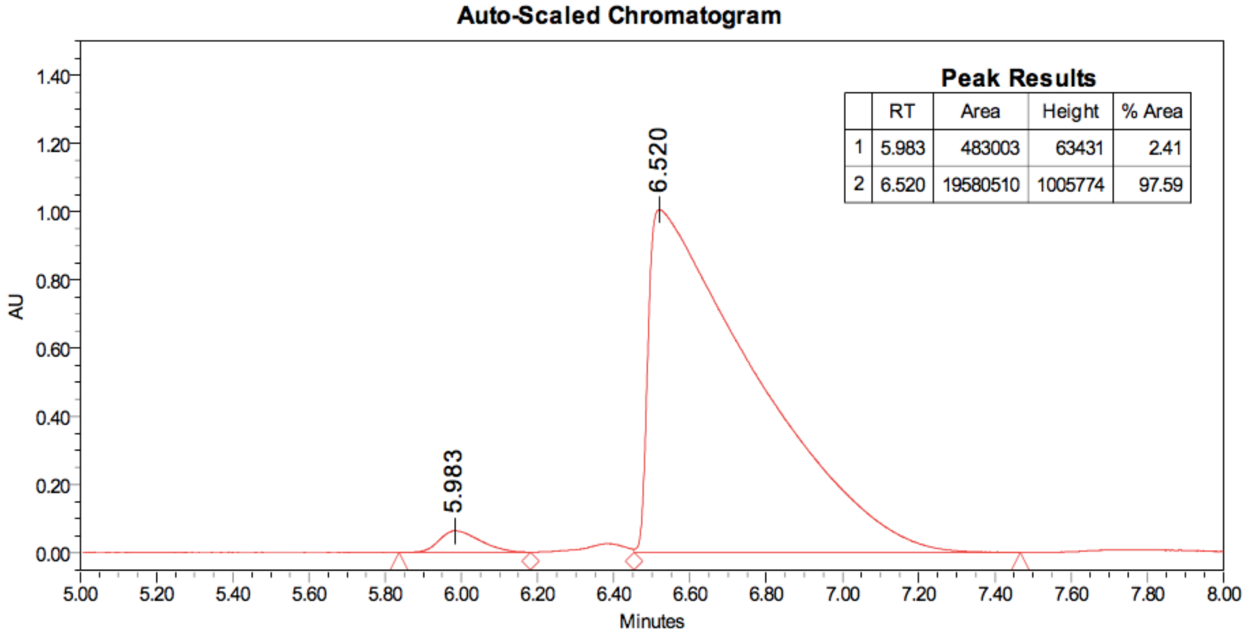

3t sample (Conditions): Cel1 column, 40.0 °C, *i*-PrOH: CO_2 = 3:97, 2 mL/min, 138 bar, 40 min; t_1 = 5.983 min, t_2 = 6.520 min, er = 2.5:97.5.

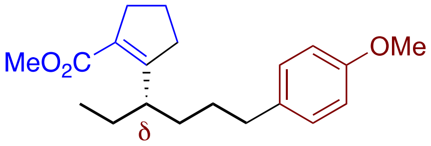

3u. 52% yield
er = 98:2, δ:γ = 2.8
After hydrogenation

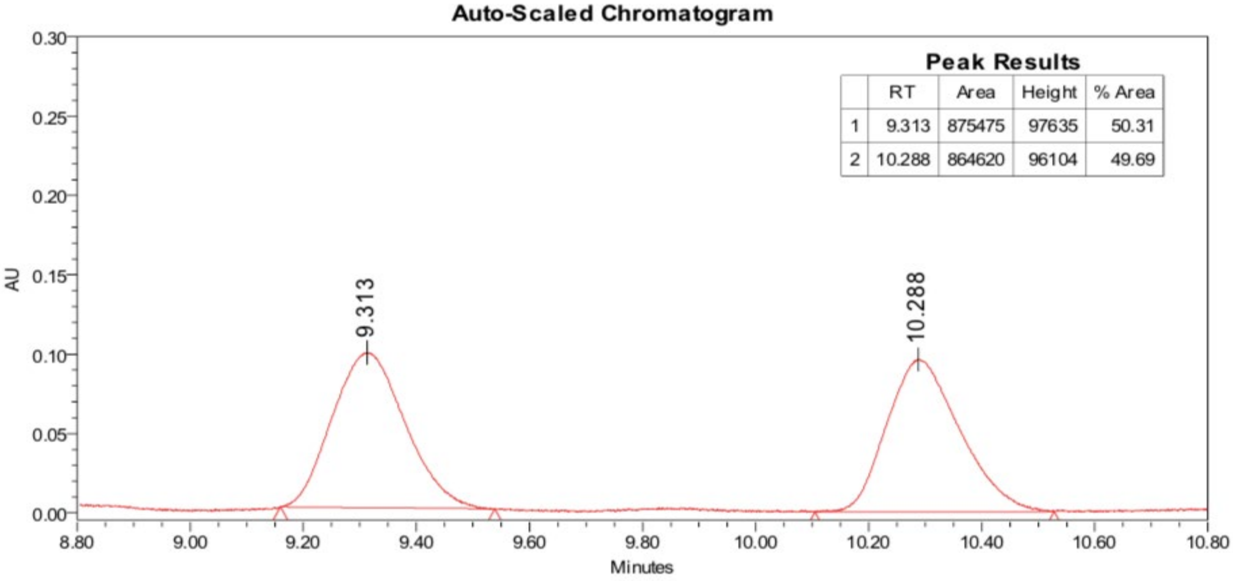

Separation by SFC, Chiralcel® **3u**-Rac sample (Conditions): Cel1 column, 40.0 °C, Heptane: CO_2 = 6:94, 1 mL/min, 138 bar, 40 min; t_1 = 9.313 min, t_2 = 10.288 min.

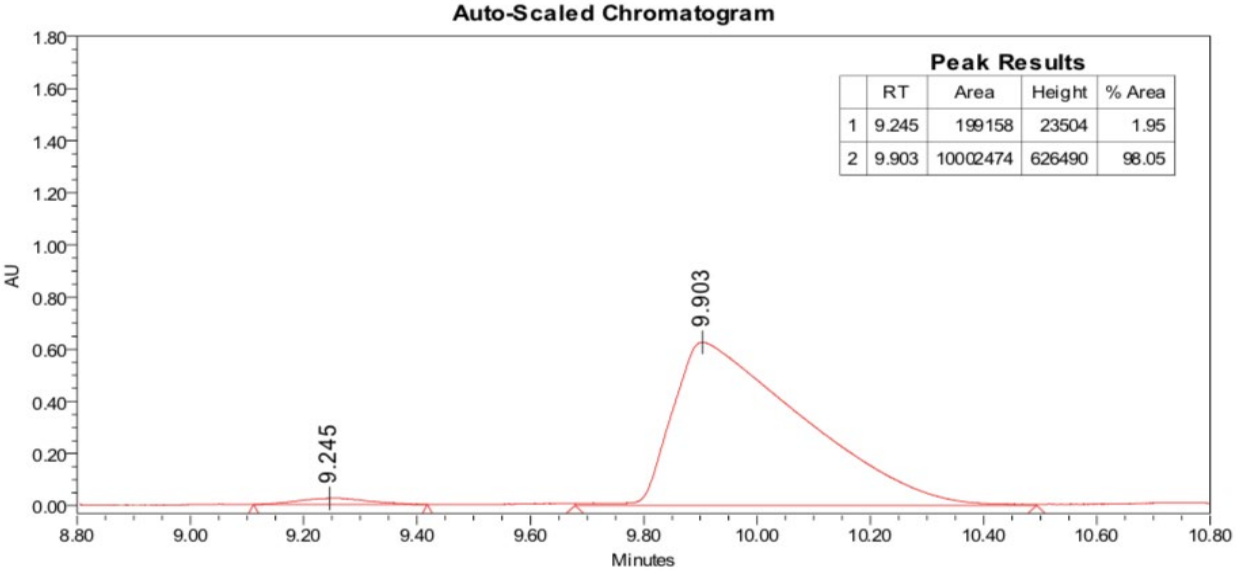

3u sample (Conditions): Cel1 column, 40.0 °C, Heptane: CO_2 = 6:94, 1 mL/min, 138 bar, 40 min; t_1 = 9.245 min, t_2 = 9.903 min, er = 2:98.

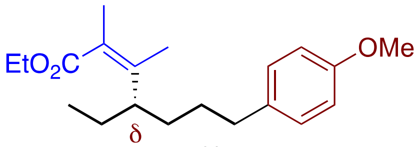

3v. 60% yield
er = 96.5:3.5, δ:γ = 4.3
After hydrogenation

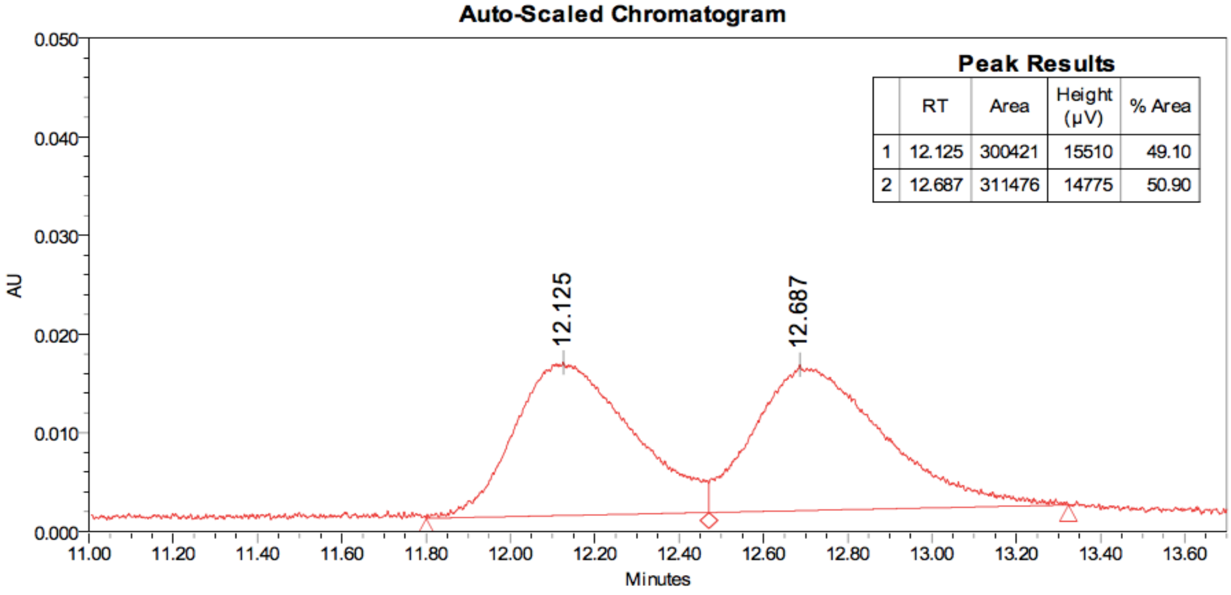

Peak Results

	RT	Area	Height (μV)	% Area
1	12.125	300421	15510	49.10
2	12.687	311476	14775	50.90

Separation by SFC, Chiralcel® **3v**-Rac sample (Conditions): Cel1 column, 40.0 °C, Heptane: CO_2 = 1:99, 1 mL/min, 138 bar, 40 min; t_1 = 12.125 min, t_2 = 12.687 min.

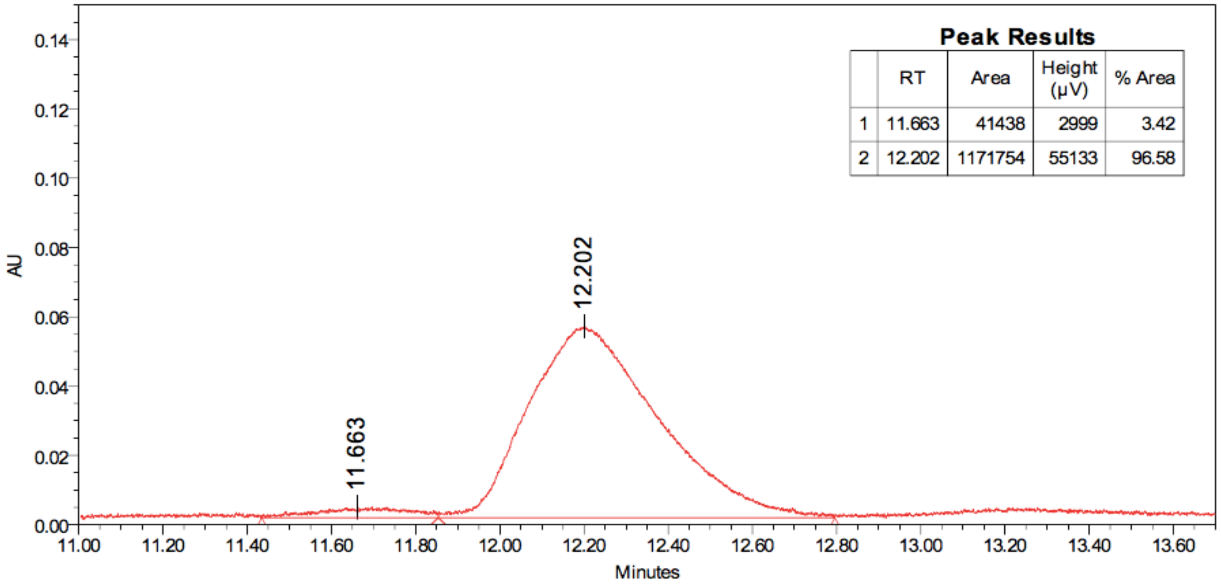

Peak Results

	RT	Area	Height (μV)	% Area
1	11.663	41438	2999	3.42
2	12.202	1171754	55133	96.58

3v sample (Conditions): Cel1 column, 40.0 °C, Heptane: CO_2 = 1:99, 1 mL/min, 138 bar, 40 min; t_1 = 11.663 min, t_2 = 12.202 min, er = 96.5:3.5.

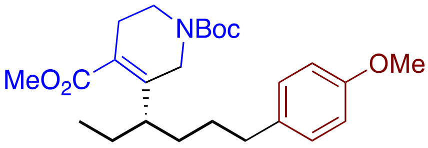

3w. 61% yield
er = 98:2
After hydrogenation

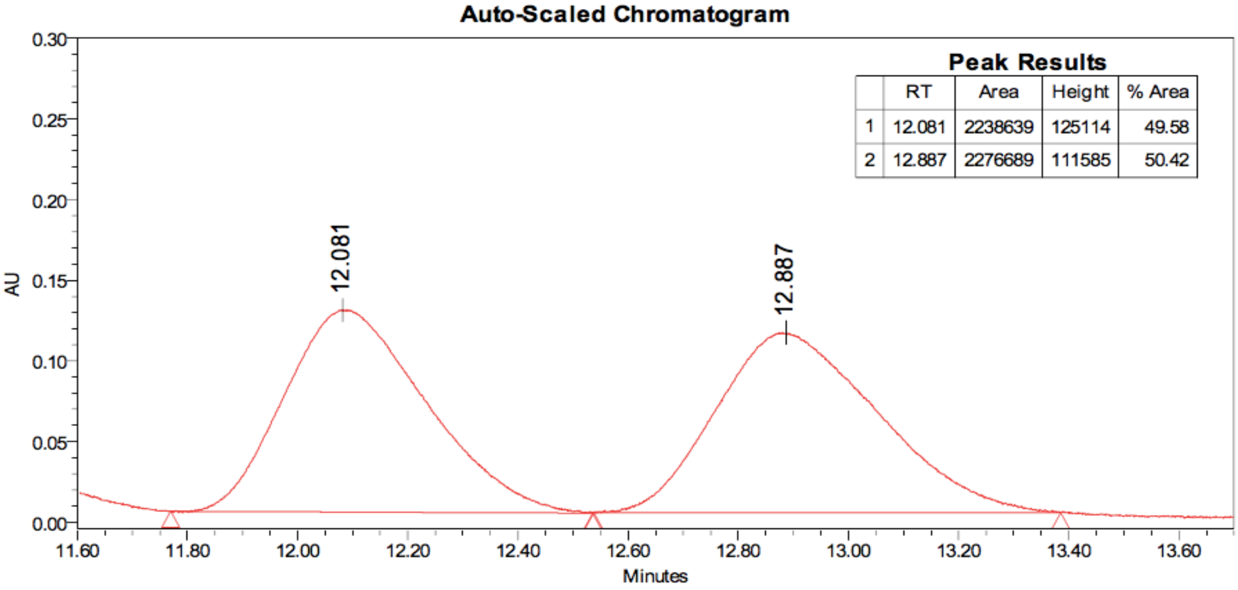

Separation by SFC, Chiralcel® **3w**-Rac sample (Conditions): Cel1 column, 40.0 °C, *i*-PrOH: CO_2 = 3:97, 1 mL/min, 138 bar, 40 min; t_1 = 12.081 min, t_2 = 12.887 min.

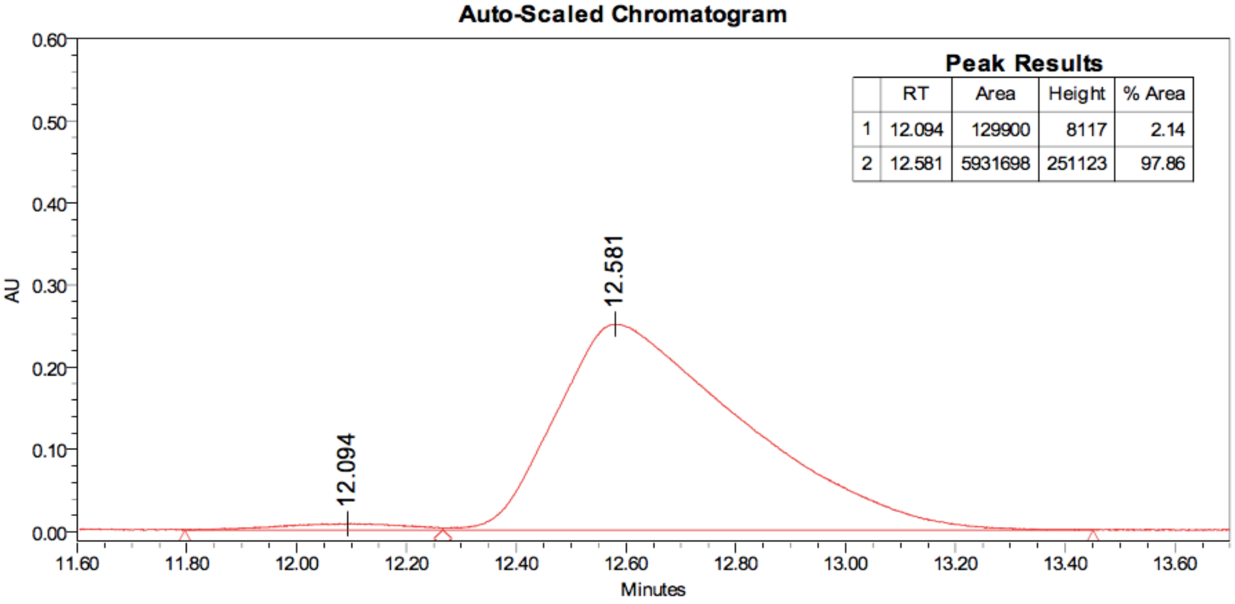

3w sample (Conditions): Cel1 column, 40.0 °C, *i*-PrOH: CO_2 = 3:97, 1 mL/min, 138 bar, 40 min; t_1 = 12.094 min, t_2 = 12.581 min, er = 2:98.

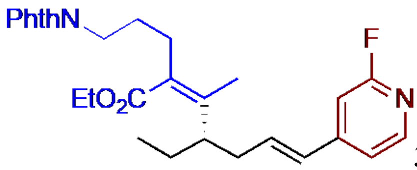

3x

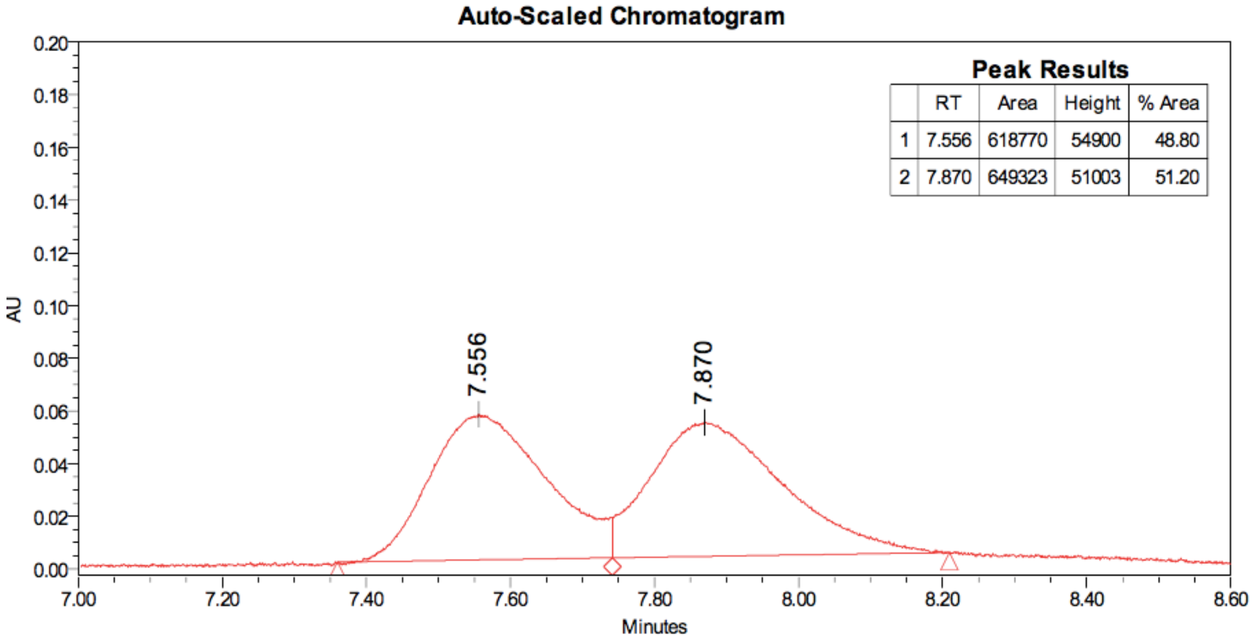

Separation by SFC, Chiralcel® **3x**-Rac sample (Conditions): Cel1 column, 40.0 °C, *i*-PrOH: CO_2 = 4:96, 2 mL/min, 138 bar, 40 min; t_1 = 7.556 min, t_2 = 7.870 min.

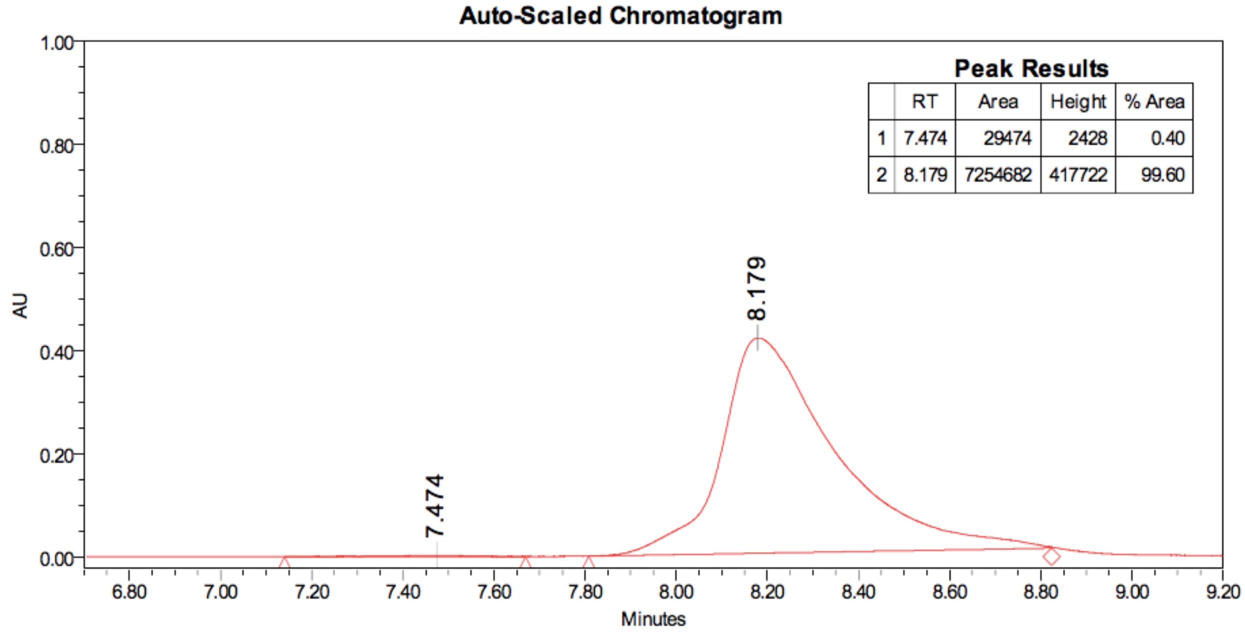

3x sample (Conditions): Cel1 column, 40.0 °C, *i*-PrOH: CO_2 = 4:96, 2 mL/min, 138 bar, 40 min; t_1 = 7.474 min, t_2 = 8.179 min, er = 0.4:99.6.

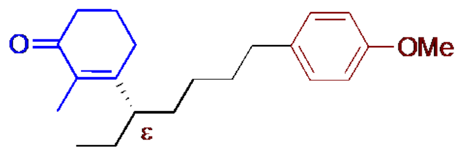

from E-alkene
63% yield, er = 94.5:5.5, ε:δ = 2.6
After hydrogenation

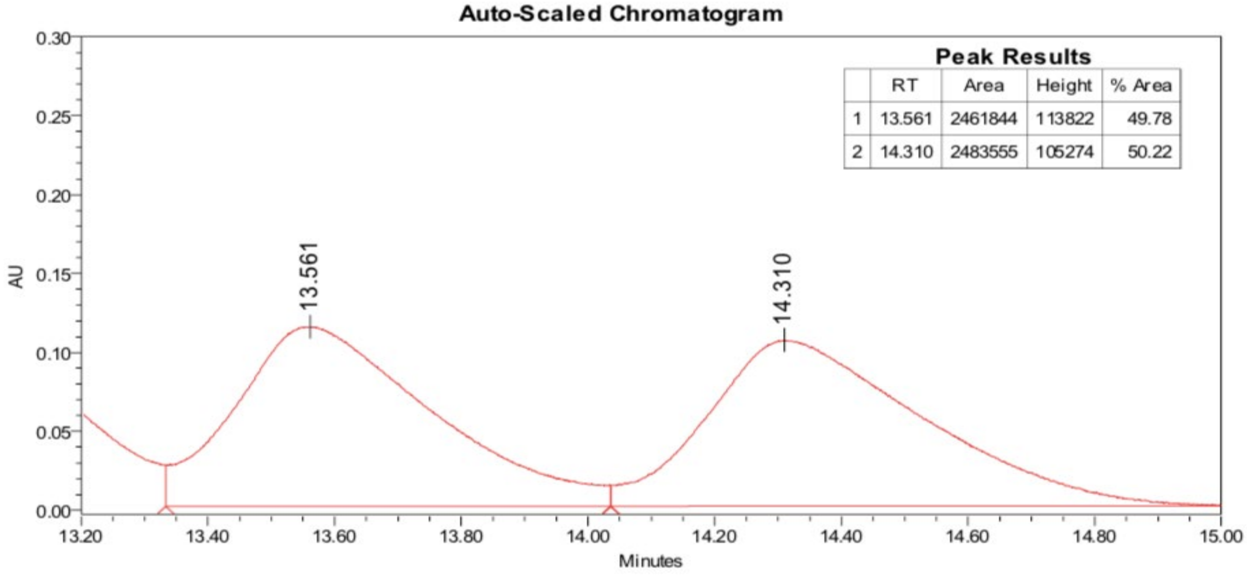

Separation by SFC, Chiralcel® **3y**-Rac sample (Conditions): Cel1 column, 40.0 °C, *i*-PrOH: CO_2 = 3:97, 1 mL/min, 138 bar, 40 min; t_1 = 13.561 min, t_2 = 14.310 min.

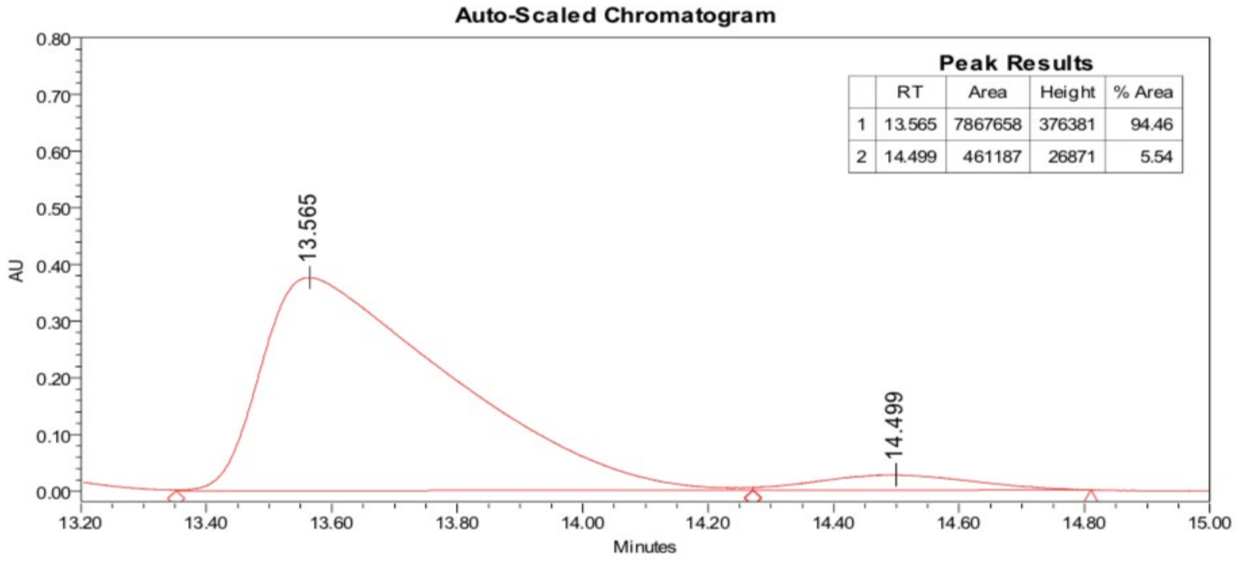

3y sample (Conditions): Cel1 column, 40.0 °C, *i*-PrOH: CO_2 = 3:97, 1 mL/min, 138 bar, 40 min; t_1 = 13.565 min, t_2 = 14.499 min, er = 94.5:5.5.

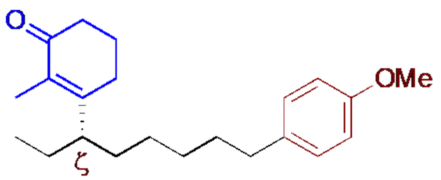

from Z-alkene
59% yield, er = 10:90, ζ:ε = 2.6
After hydrogenation

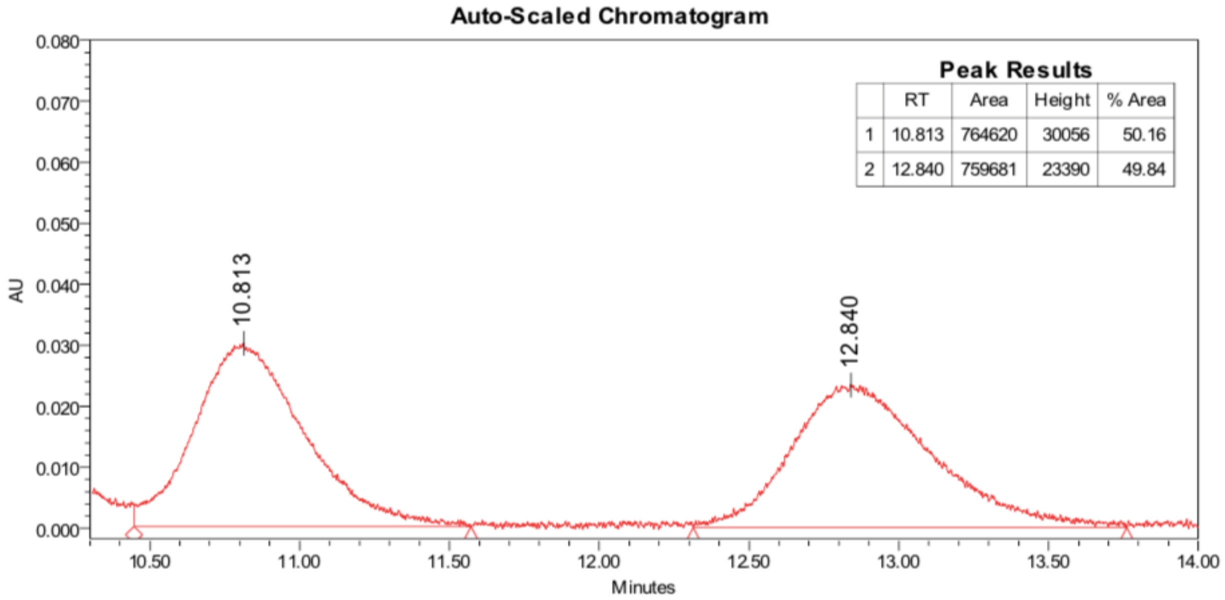

	RT	Area	Height	% Area
1	10.813	764620	30056	50.16
2	12.840	759681	23390	49.84

Separation by SFC, Chiralcel® **3z**-Rac sample (Conditions): AD-H column, 40.0 °C, *i*-PrOH: CO_2 = 3:97, 2 mL/min, 138 bar, 40 min; t_1 = 10.813 min, t_2 = 12.840 min.

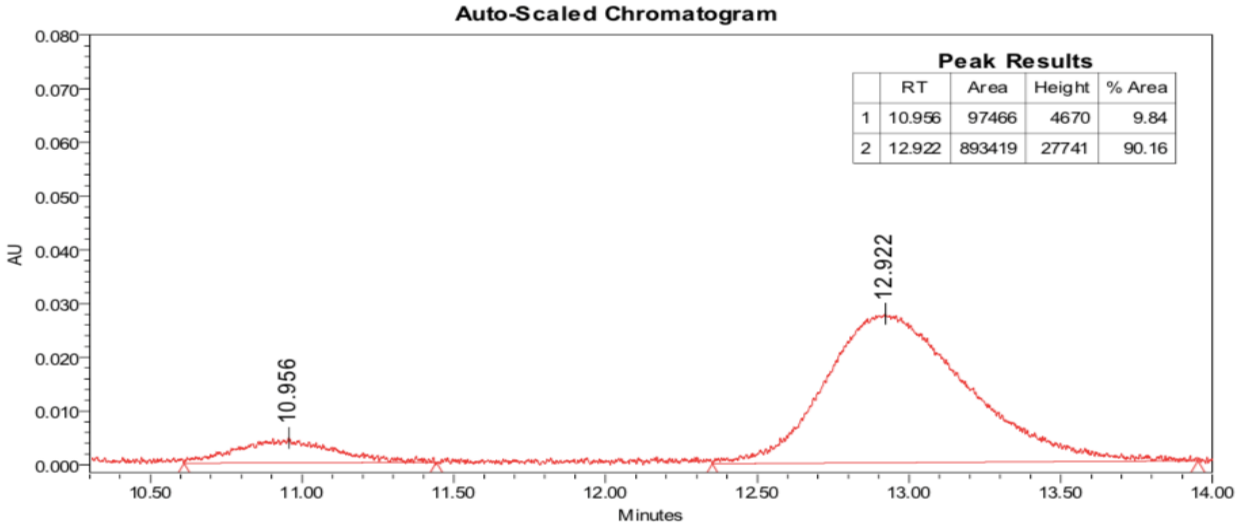

	RT	Area	Height	% Area
1	10.956	97466	4670	9.84
2	12.922	893419	27741	90.16

3z sample (Conditions): AD-H column, 40.0 °C, *i*-PrOH: CO_2 = 3:97, 2 mL/min, 138 bar, 40 min; t_1 = 10.956 min, t_2 = 12.922 min, er = 10:90.

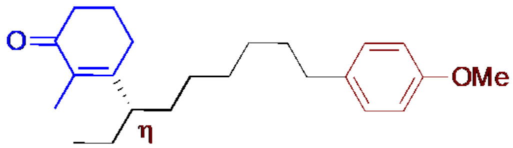

3aa

from Z-alkene
57% yield, er = 11:89, η:ζ = 2.0
After hydrogenation

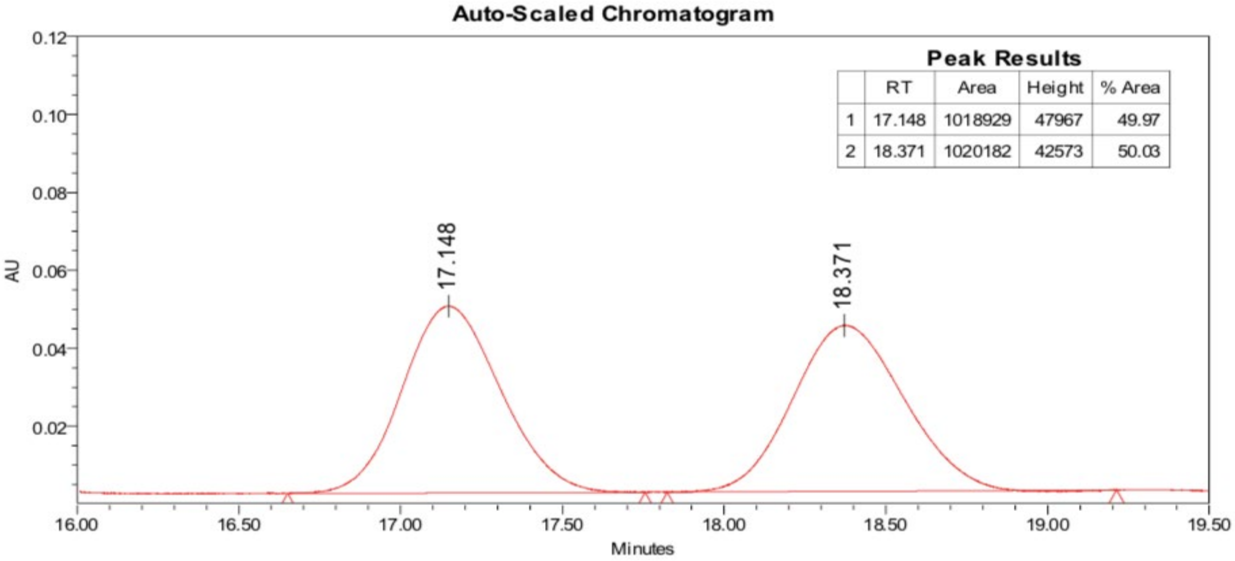

Separation by SFC, Chiralcel® **3z**-Rac sample (Conditions): Cel1 column, 40.0 °C, *i*-PrOH: CO_2 = 3:97, 1 mL/min, 138 bar, 40 min; t_1 = 17.148 min, t_2 = 18.371 min.

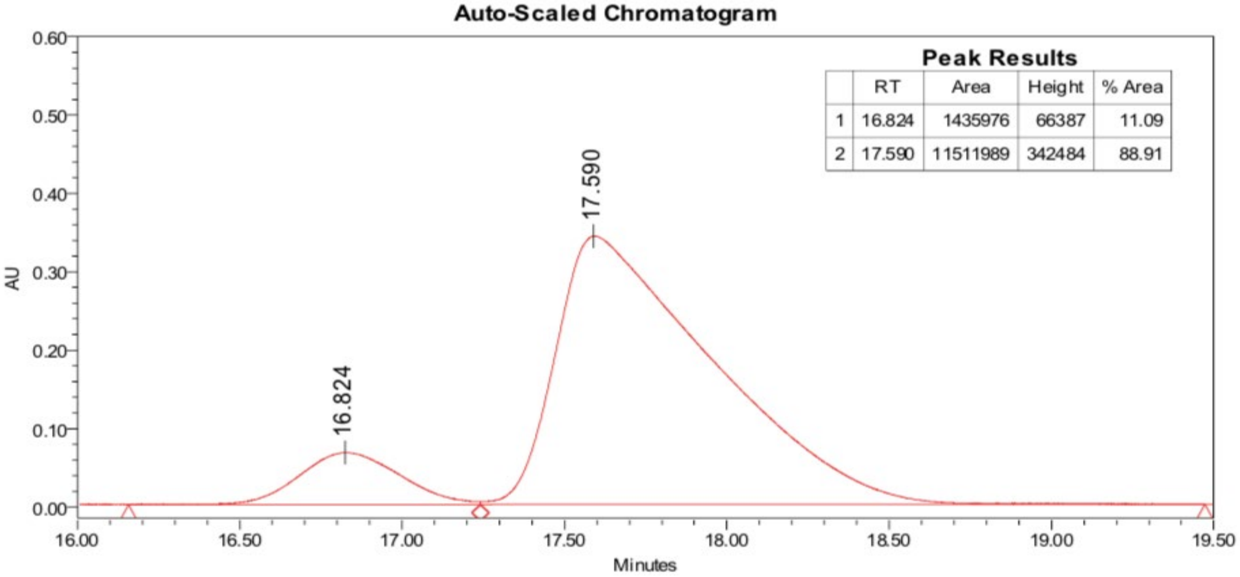

3z sample (Conditions): Cel1 column, 40.0 °C, *i*-PrOH: CO_2 = 3:97, 1 mL/min, 138 bar, 40 min; t_1 = 16.824 min, t_2 = 17.590 min, er = 11:89.

^{1}H and ^{13}C NMR Spectra (Substrates):

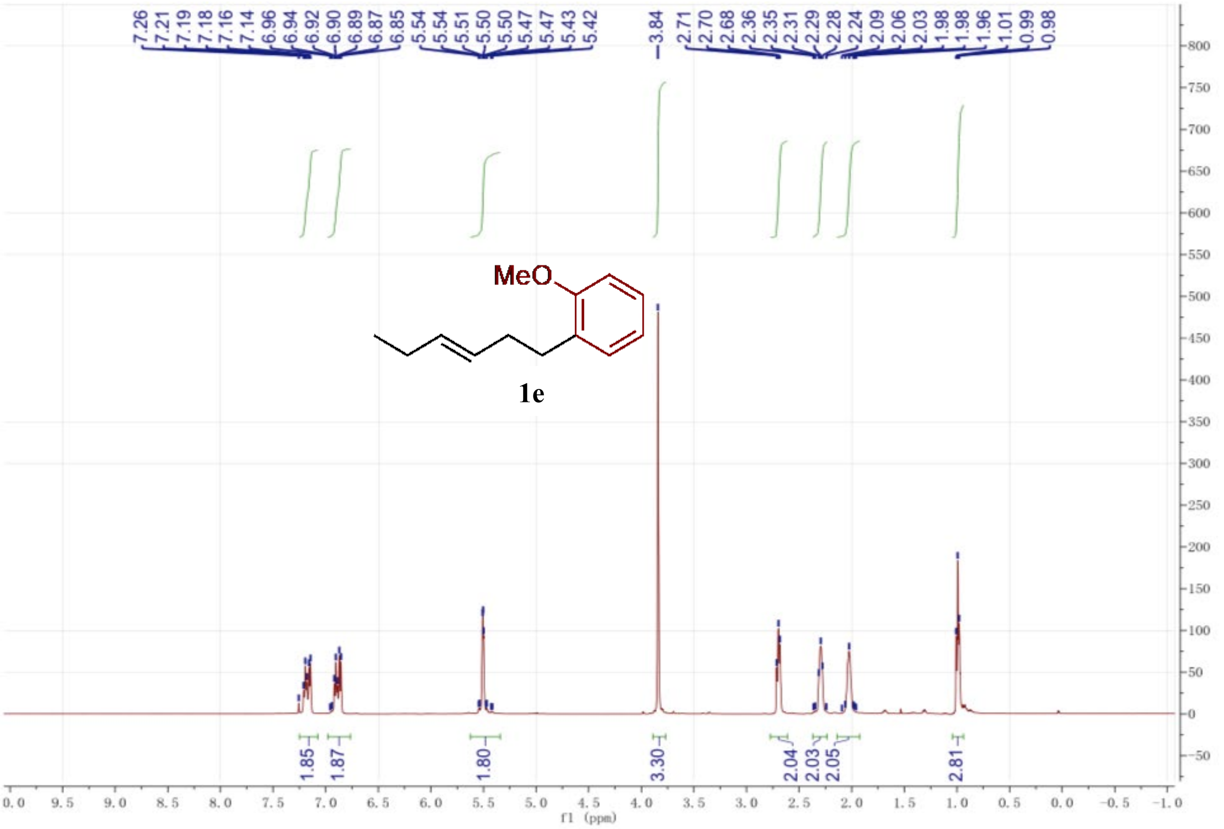

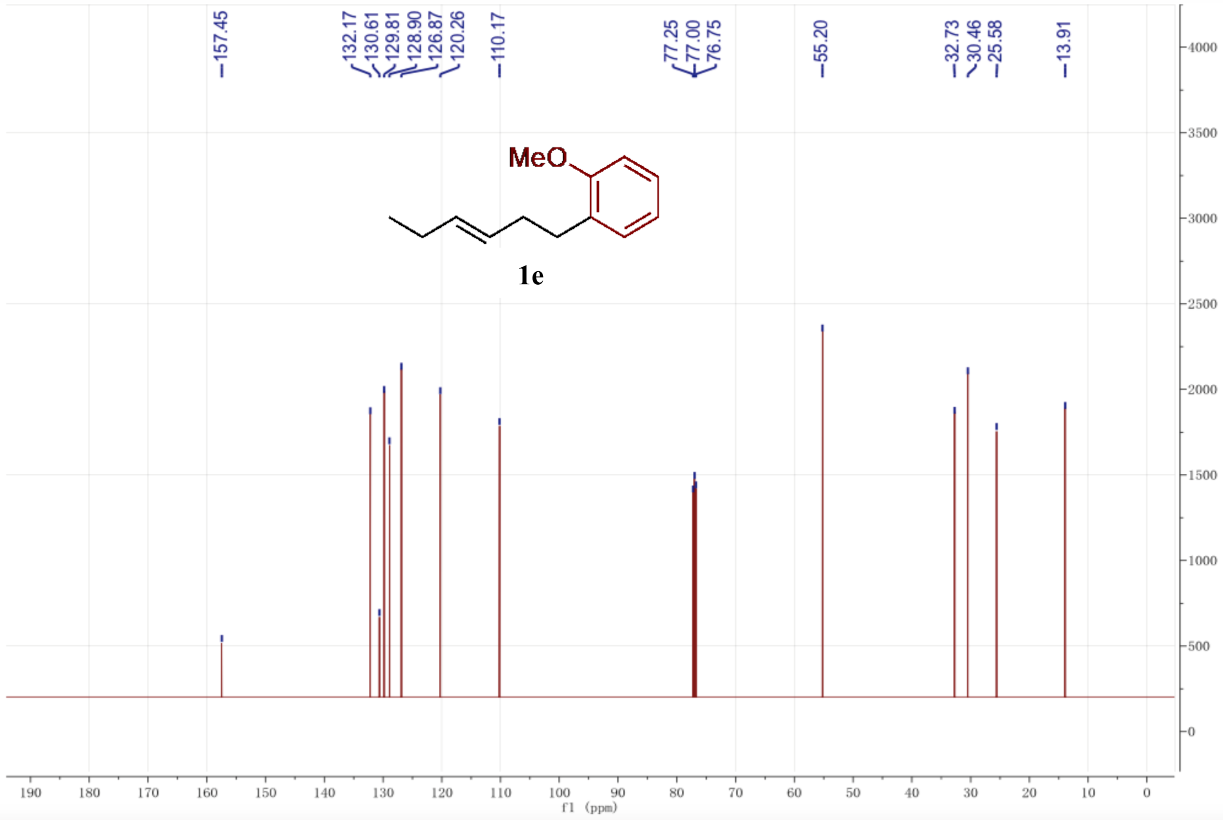

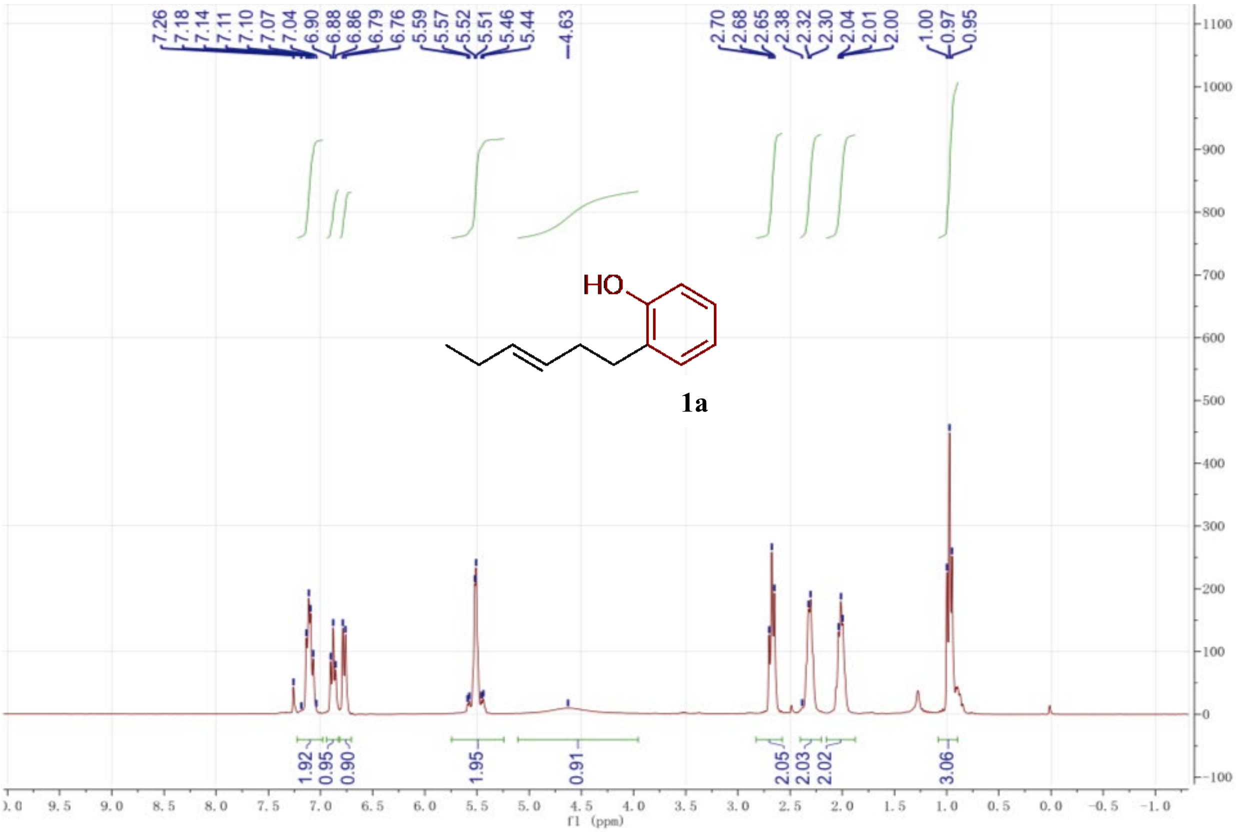

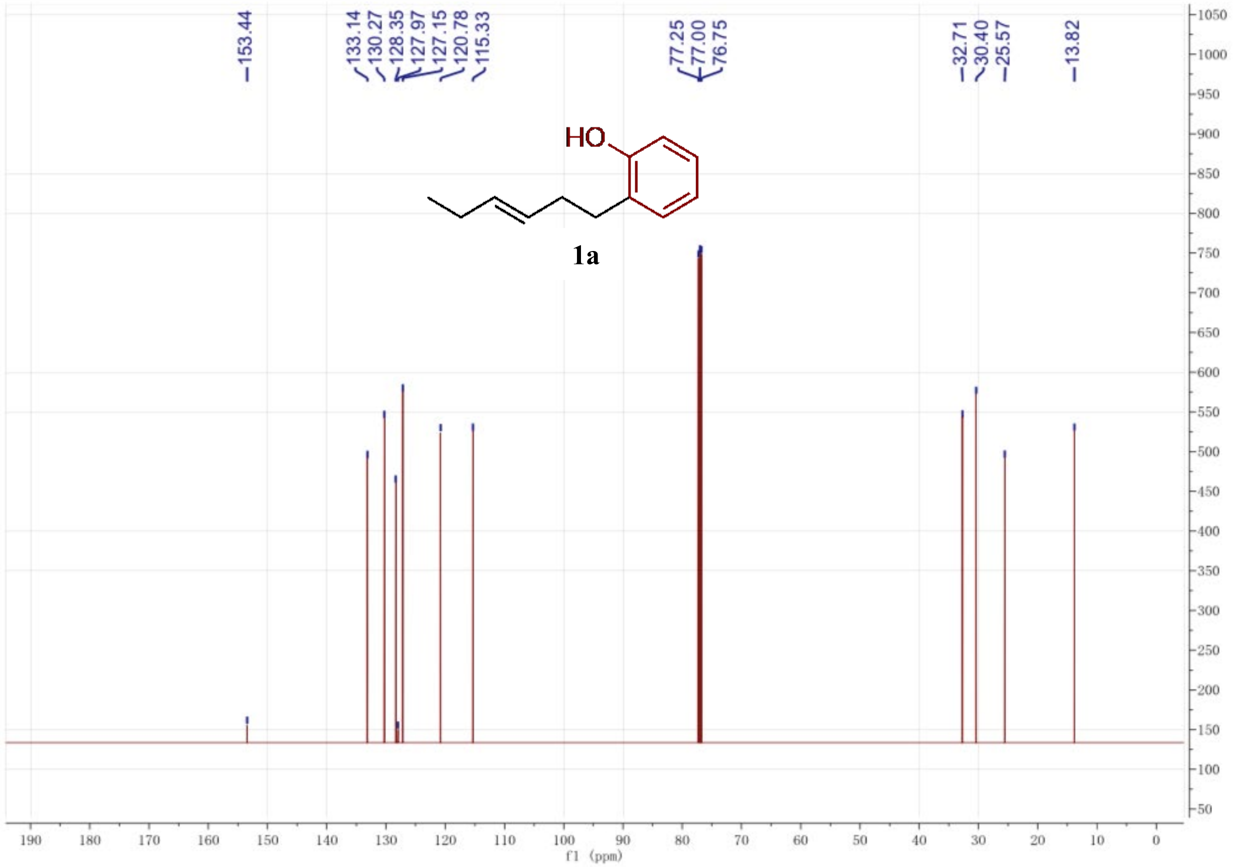

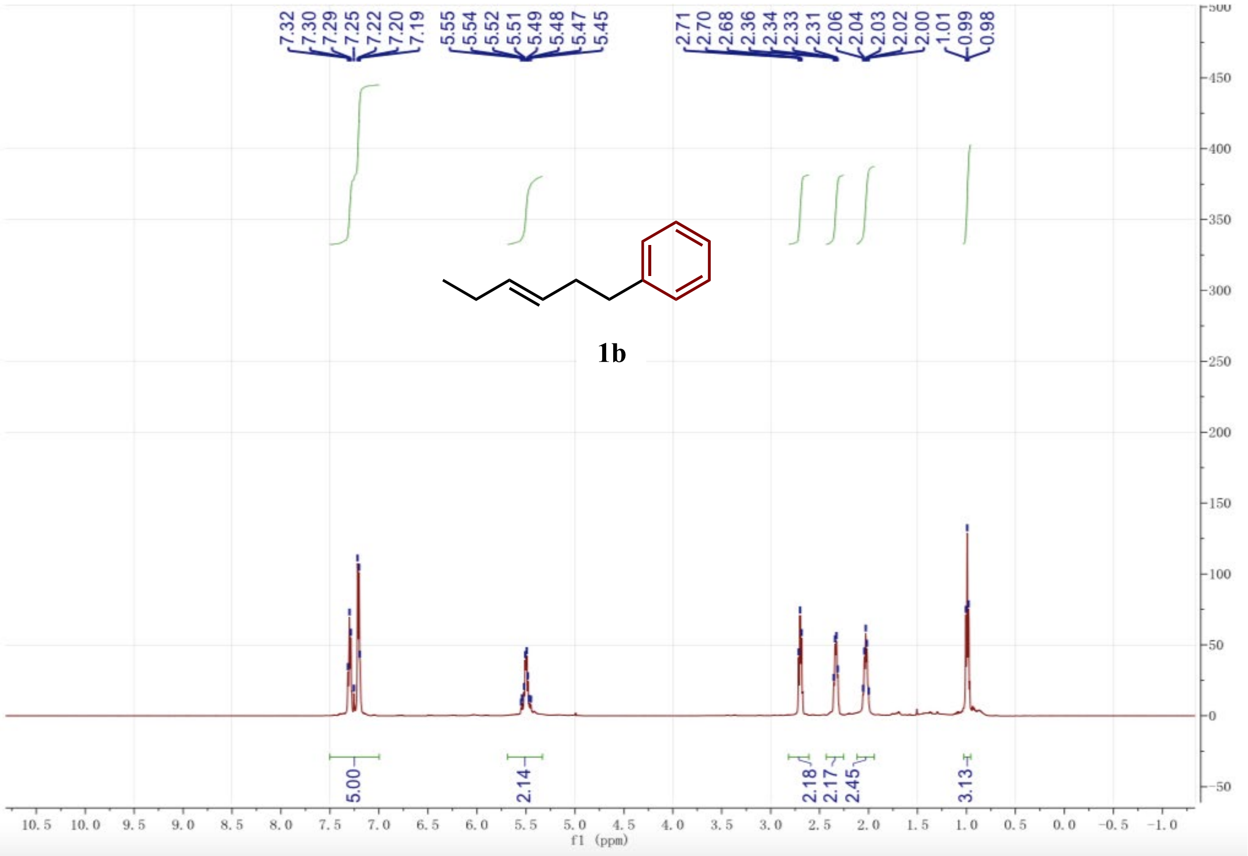
7.32
7.30
7.29
7.25
7.22
7.20
7.19
5.55
5.54
5.52
5.51
5.49
5.48
5.47
5.45
2.71
2.70
2.68
2.36
2.34
2.33
2.31
2.06
2.04
2.03
2.02
2.00
1.01
0.99
0.98
1b
5.00
2.14
2.18
2.17
2.45
3.13
f1 (ppm)

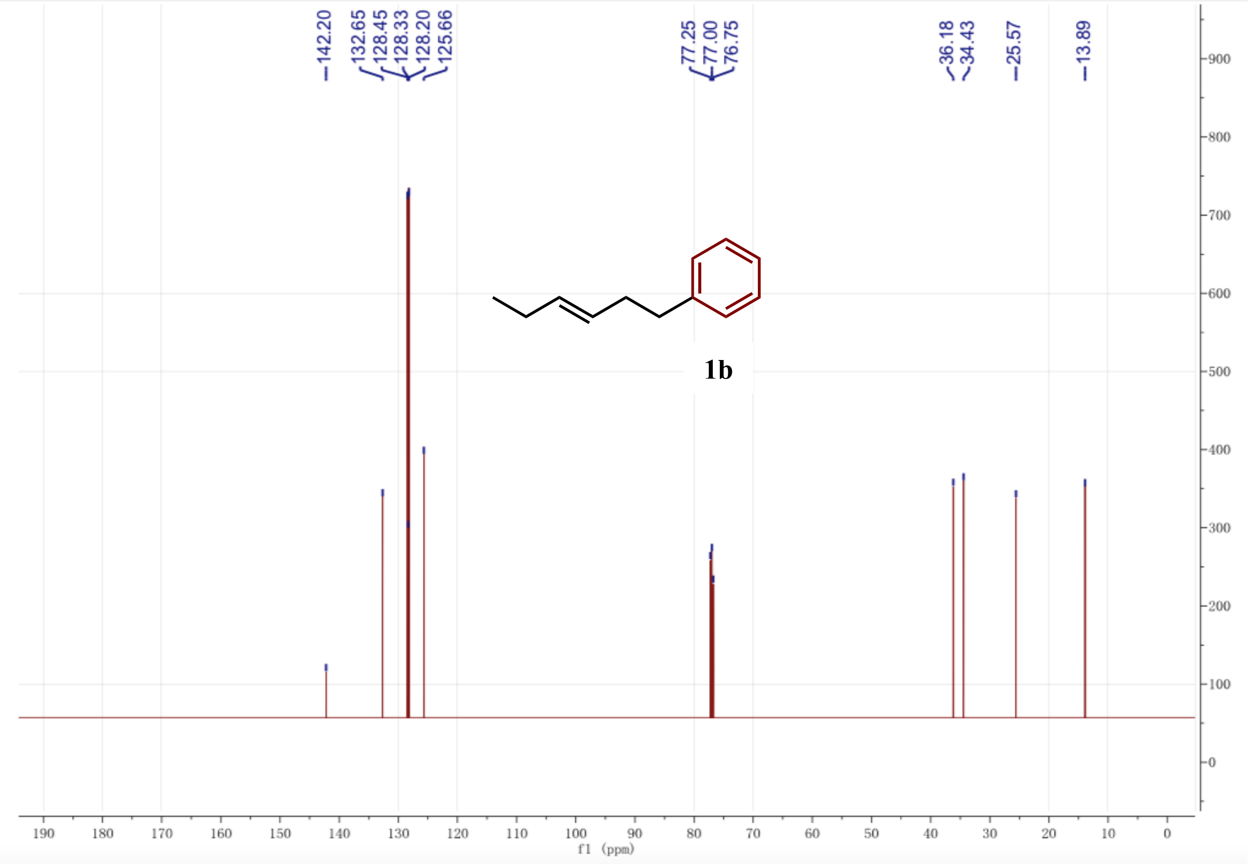
142.20
132.65
128.45
128.33
128.20
125.66
77.25
77.00
76.75
36.18
34.43
25.57
13.89
1b
f1 (ppm)

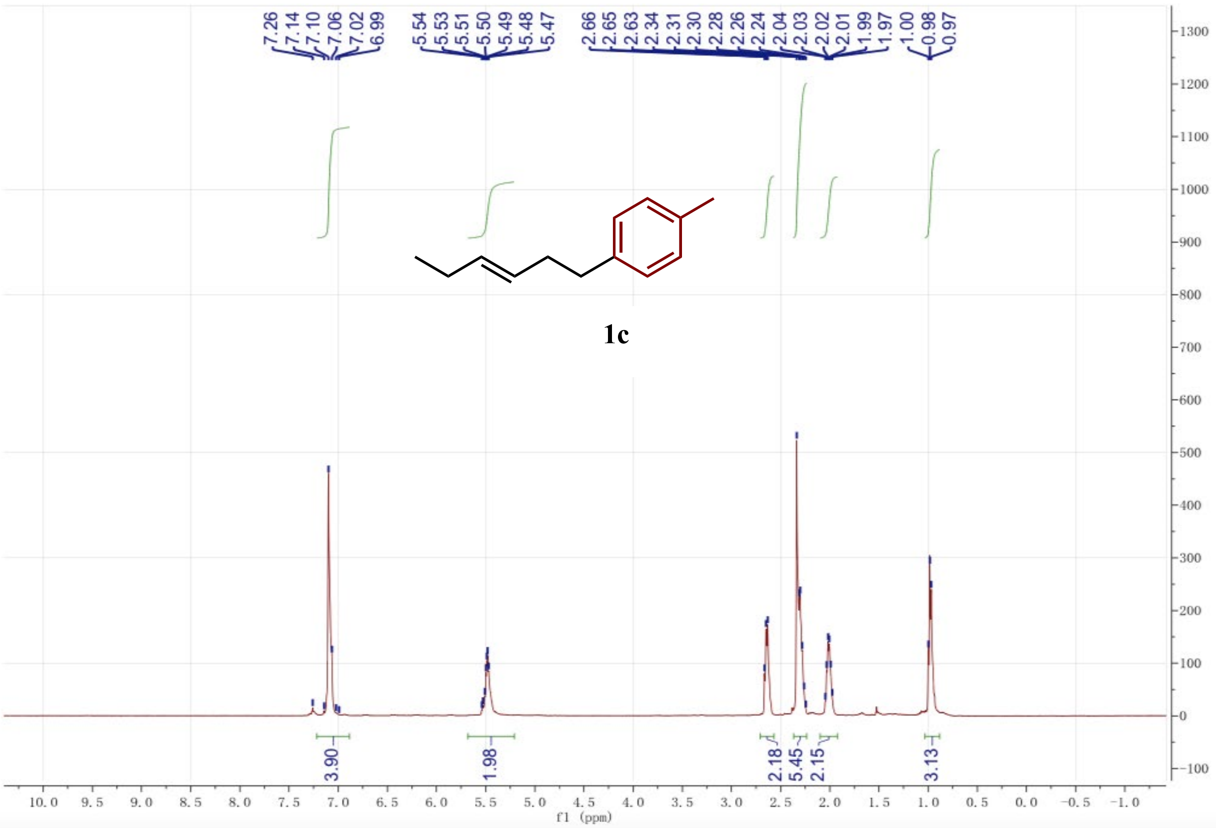

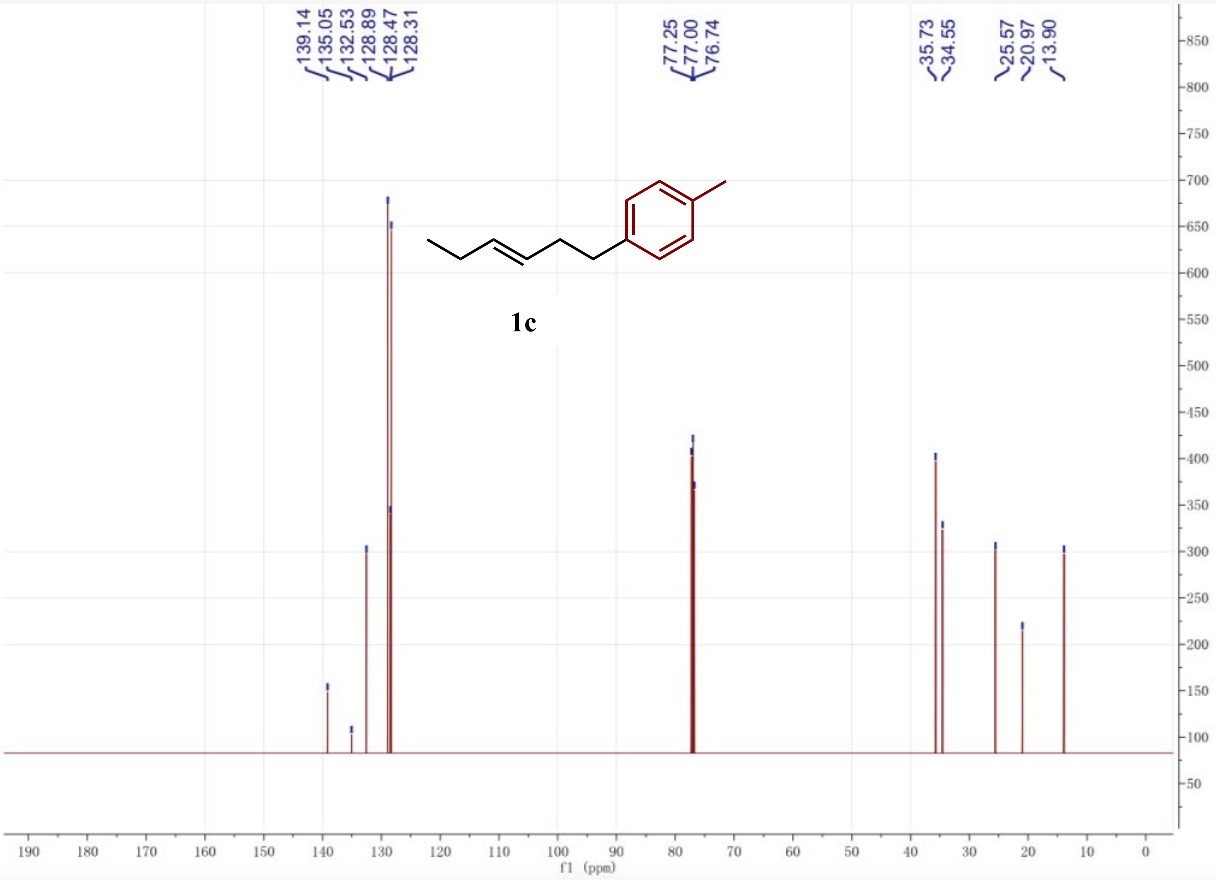

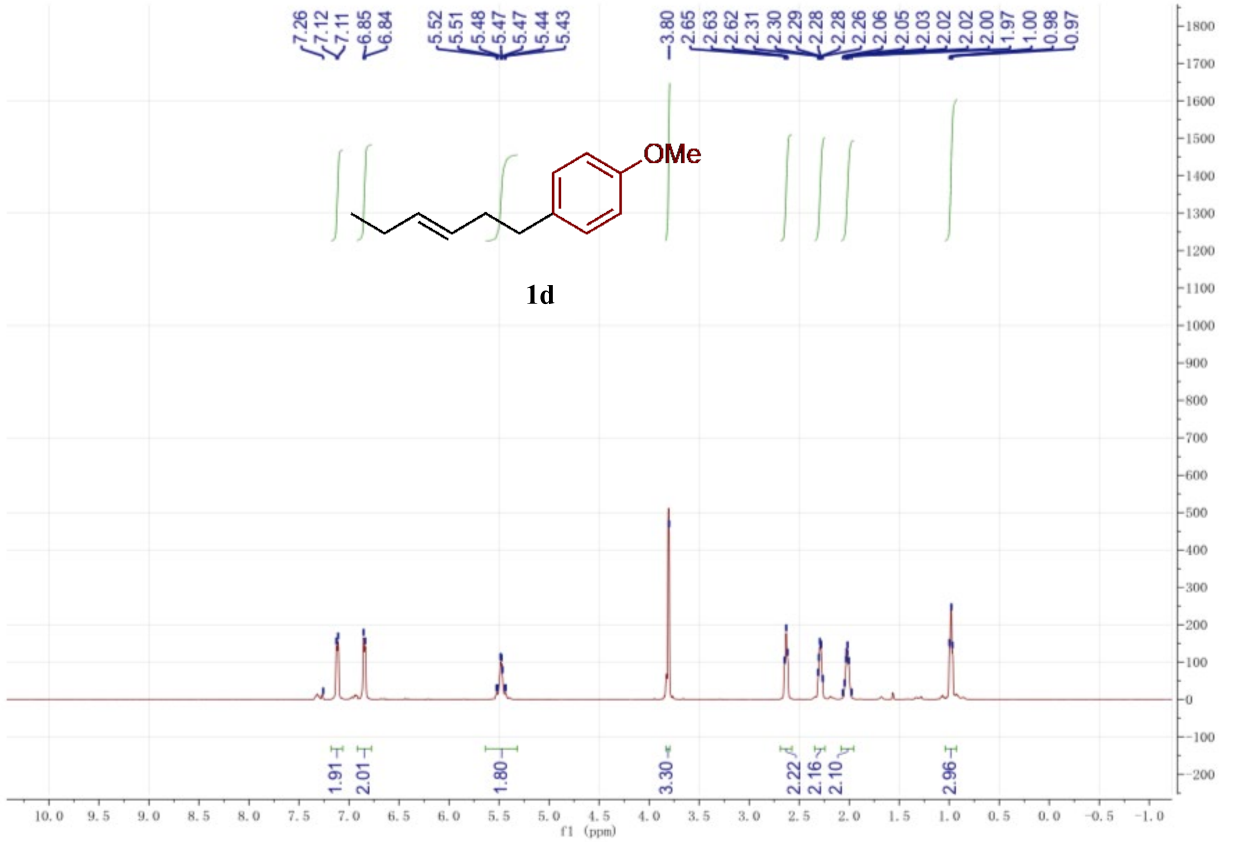
7.26
7.12
7.11
6.85
6.84
5.52
5.51
5.48
5.47
5.47
5.44
5.43
3.80
2.65
2.63
2.62
2.31
2.30
2.29
2.28
2.28
2.26
2.06
2.05
2.03
2.02
2.02
2.00
1.97
1.00
0.98
0.97
OMe
1d
1.91
2.01
1.80
3.30
2.22
2.16
2.10
2.96
f1 (ppm)

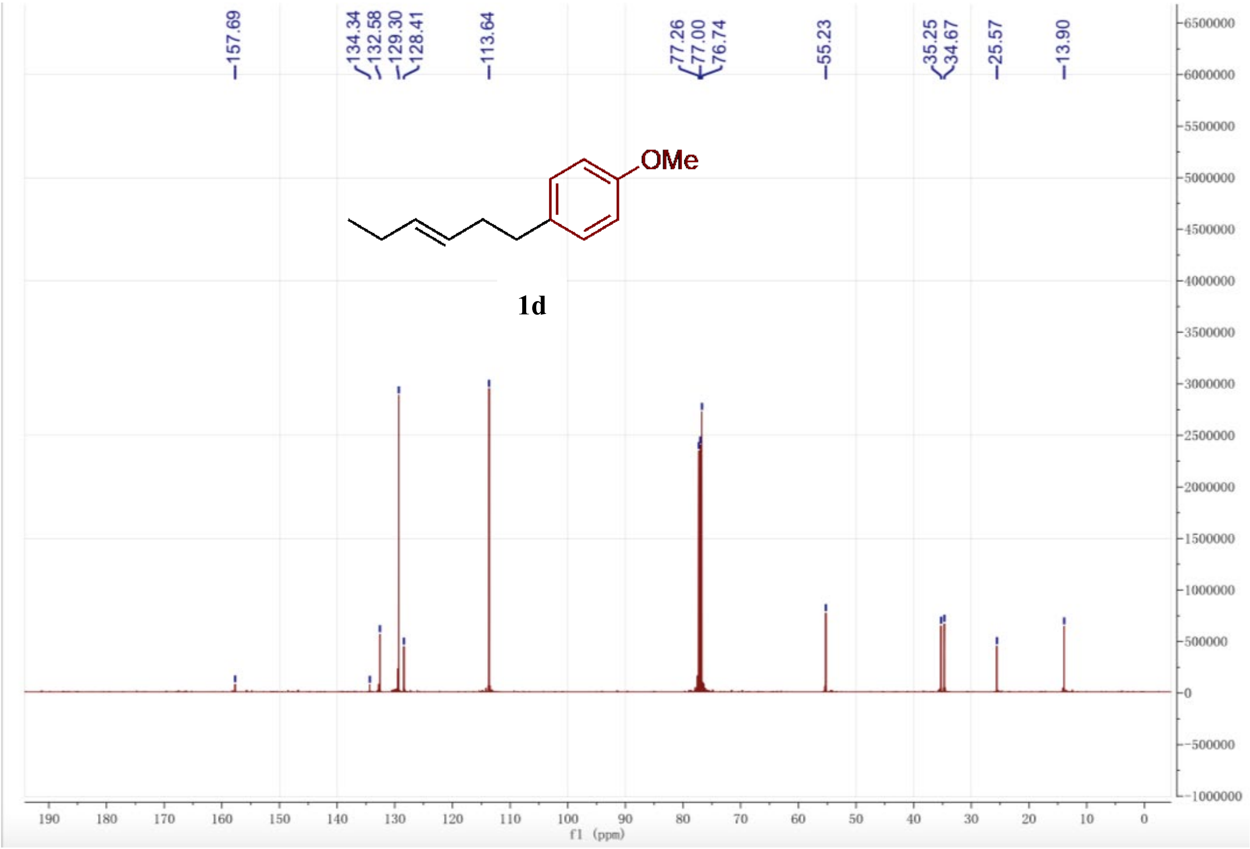
157.69
134.34
132.58
129.30
128.41
113.64
77.26
77.00
76.74
55.23
35.25
34.67
25.57
13.90
OMe
1d
f1 (ppm)

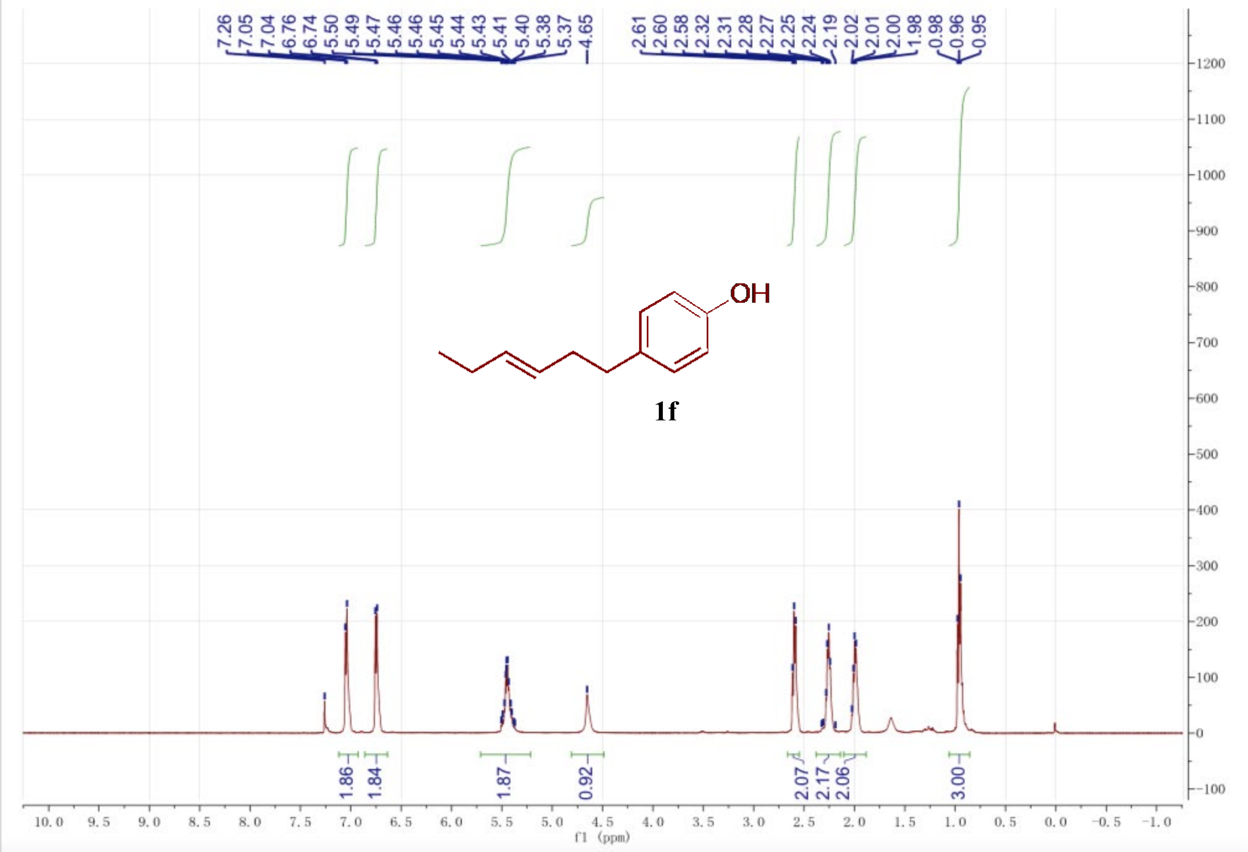

7.26
7.05
7.04
6.76
6.74
5.50
5.49
5.47
5.46
5.46
5.45
5.44
5.43
5.41
5.40
5.38
5.37
4.65
2.61
2.60
2.58
2.32
2.31
2.28
2.27
2.25
2.24
2.19
2.02
2.01
2.00
1.98
0.98
0.96
0.95
OH
1f
1.86
1.84
1.87
0.92
2.07
2.17
2.06
3.00
f1 (ppm)

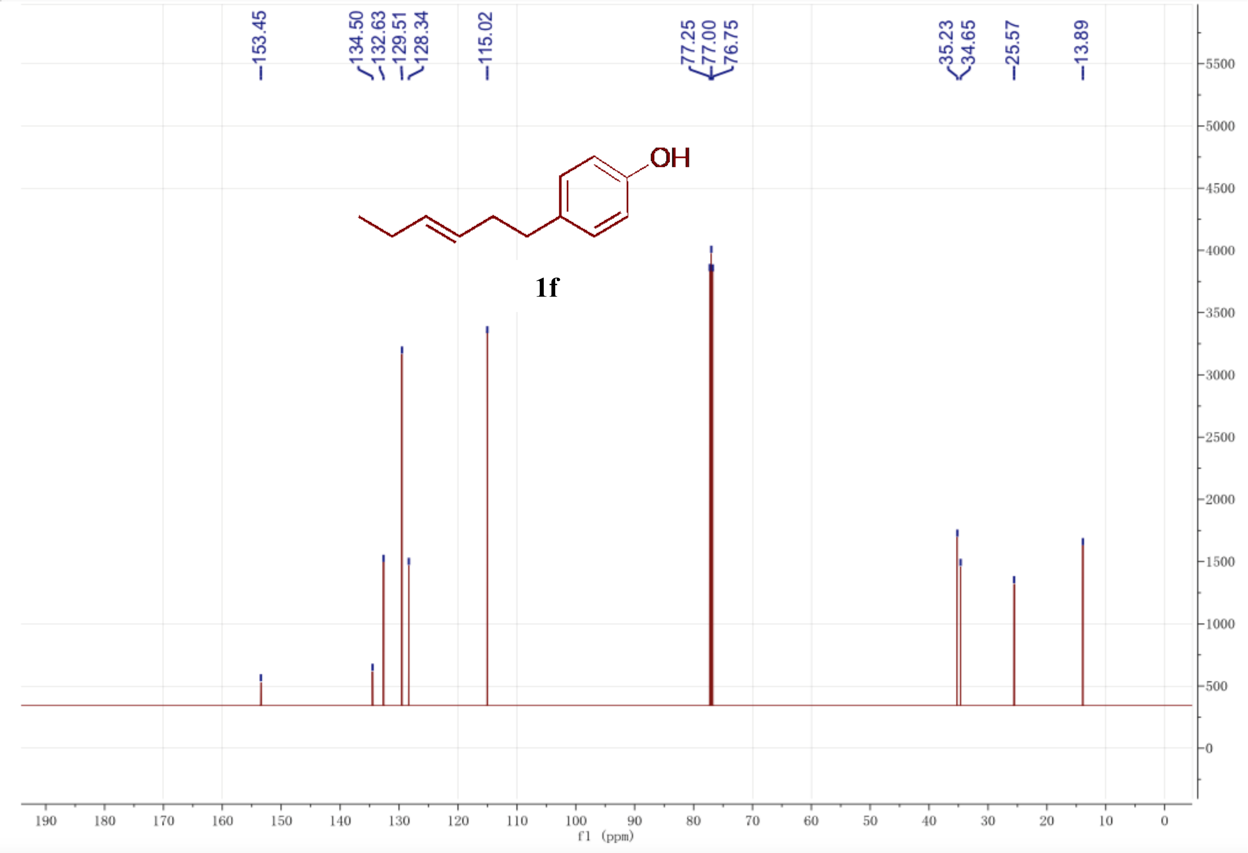

153.45
134.50
132.63
129.51
128.34
115.02
77.25
77.00
76.75
35.23
34.65
25.57
13.89
OH
1f
f1 (ppm)

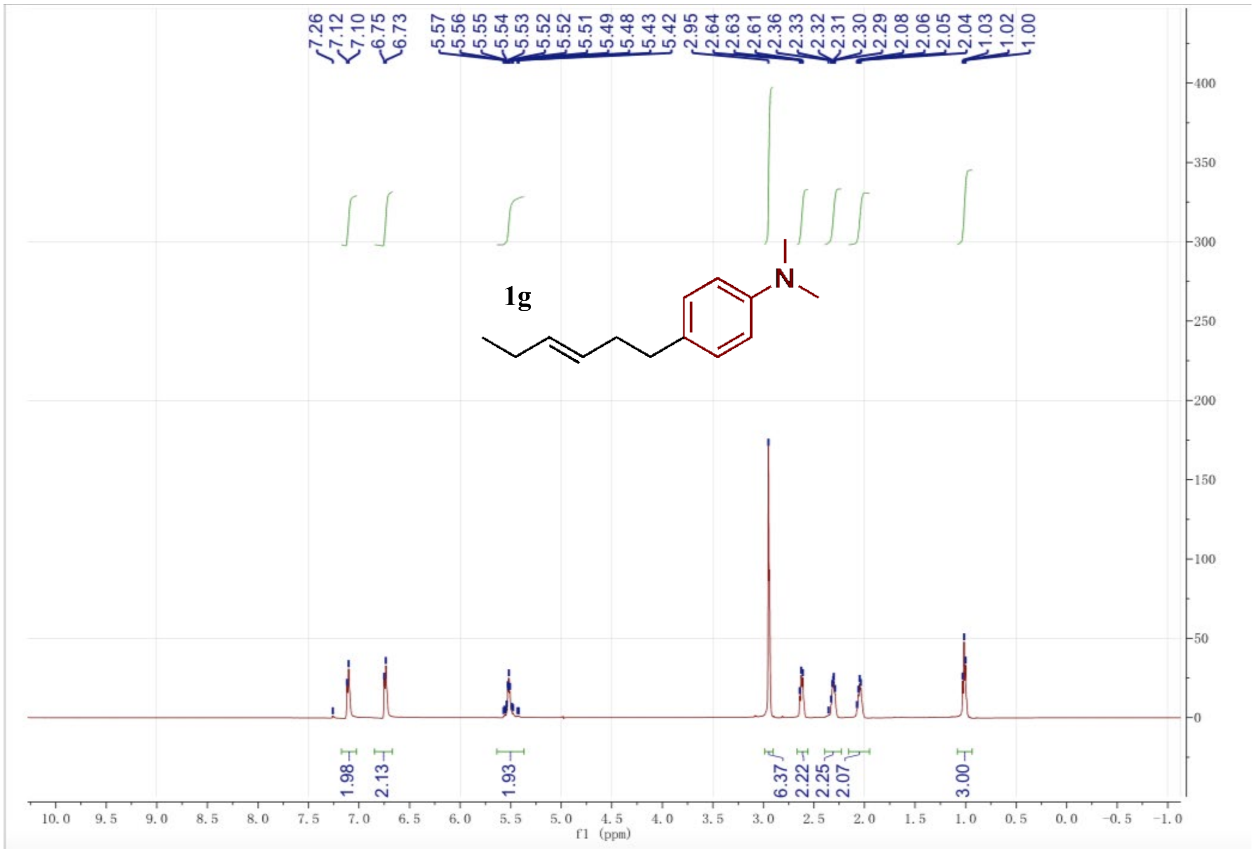
1g
N
f1 (ppm)
1.98
2.13
1.93
6.37
2.22
2.25
2.07
3.00

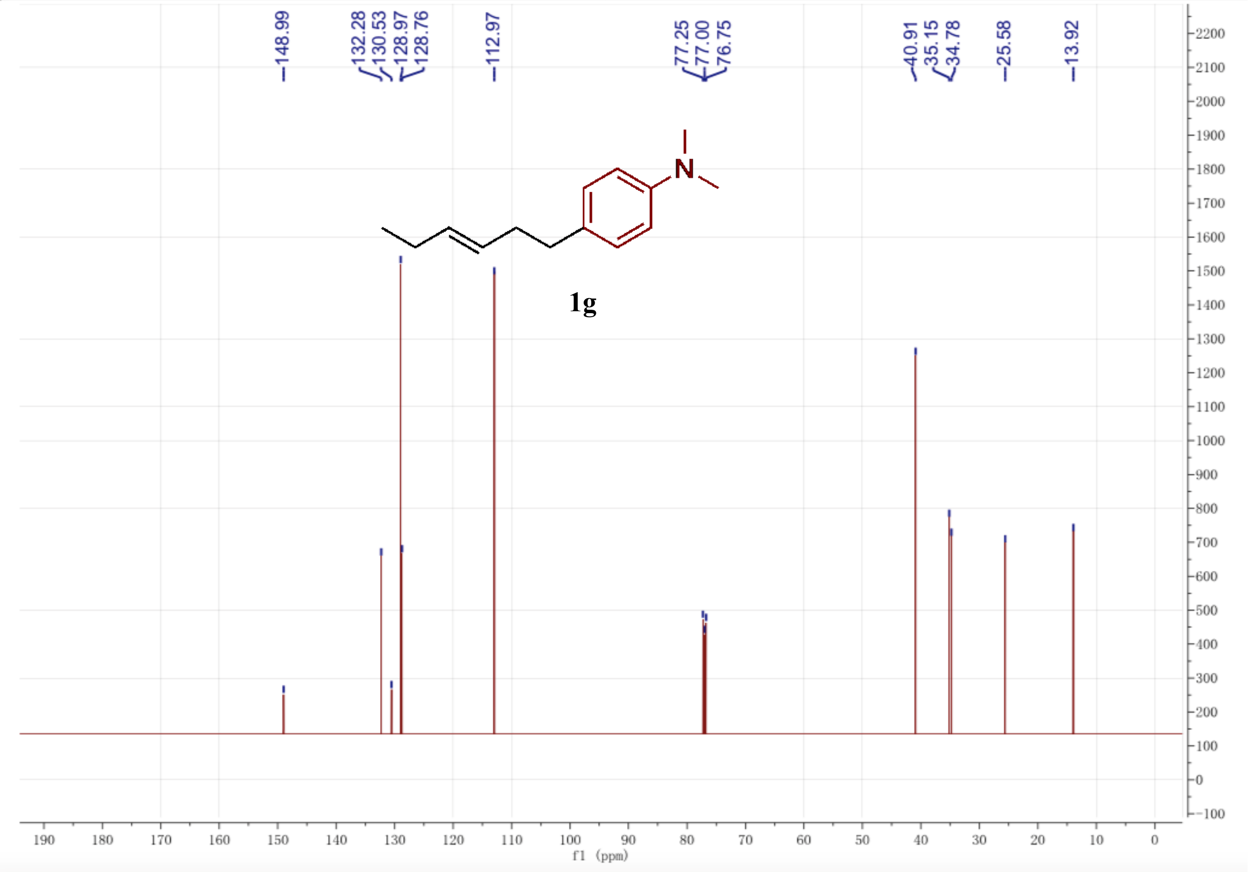
148.99
132.28
130.53
128.97
128.76
112.97
77.25
77.00
76.75
40.91
35.15
34.78
25.58
13.92
N
1g
f1 (ppm)

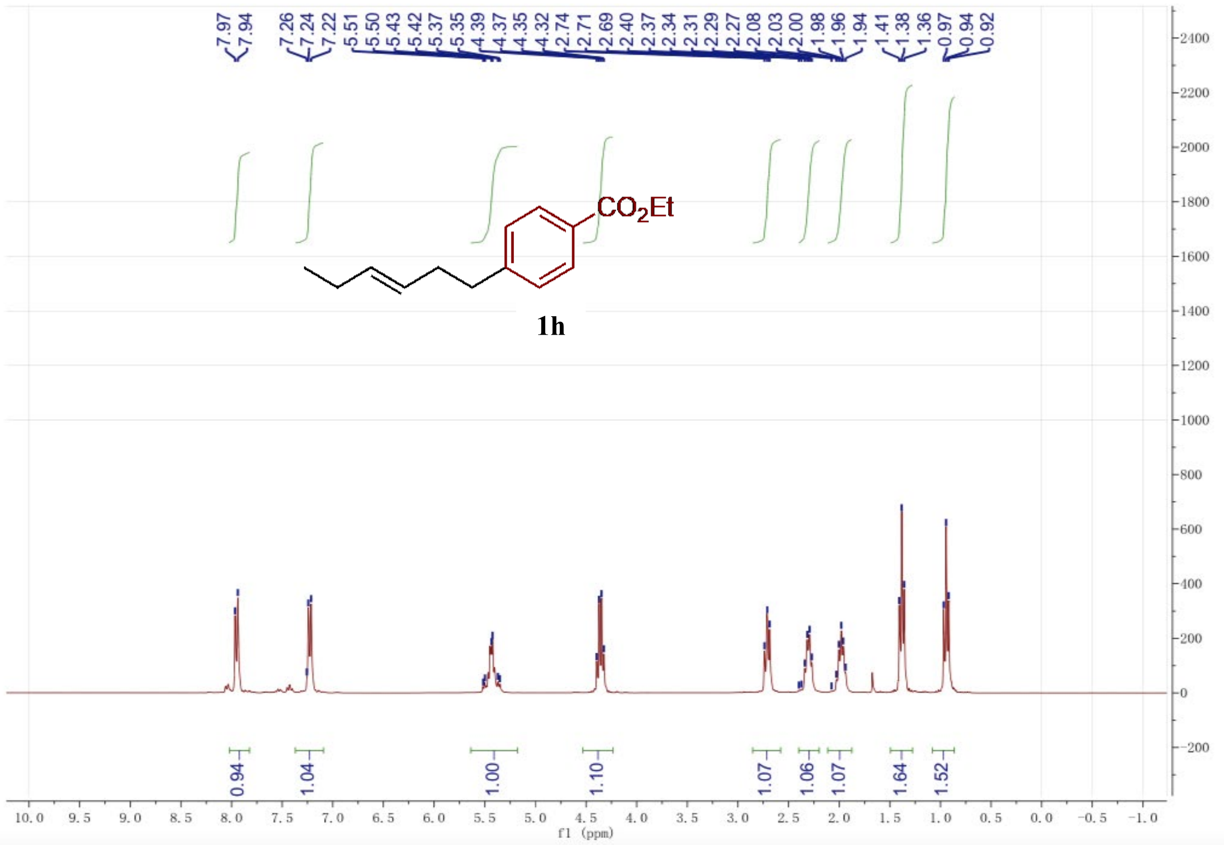
7.97
7.94
7.26
7.24
7.22
5.51
5.50
5.43
5.42
5.37
5.35
4.39
4.37
4.35
4.32
2.74
2.71
2.69
2.40
2.37
2.34
2.31
2.29
2.27
2.08
2.03
2.00
1.98
1.96
1.94
1.41
1.38
1.36
0.97
0.94
0.92
CO2Et
1h
0.94
1.04
1.00
1.10
1.07
1.06
1.07
1.64
1.52
f1 (ppm)

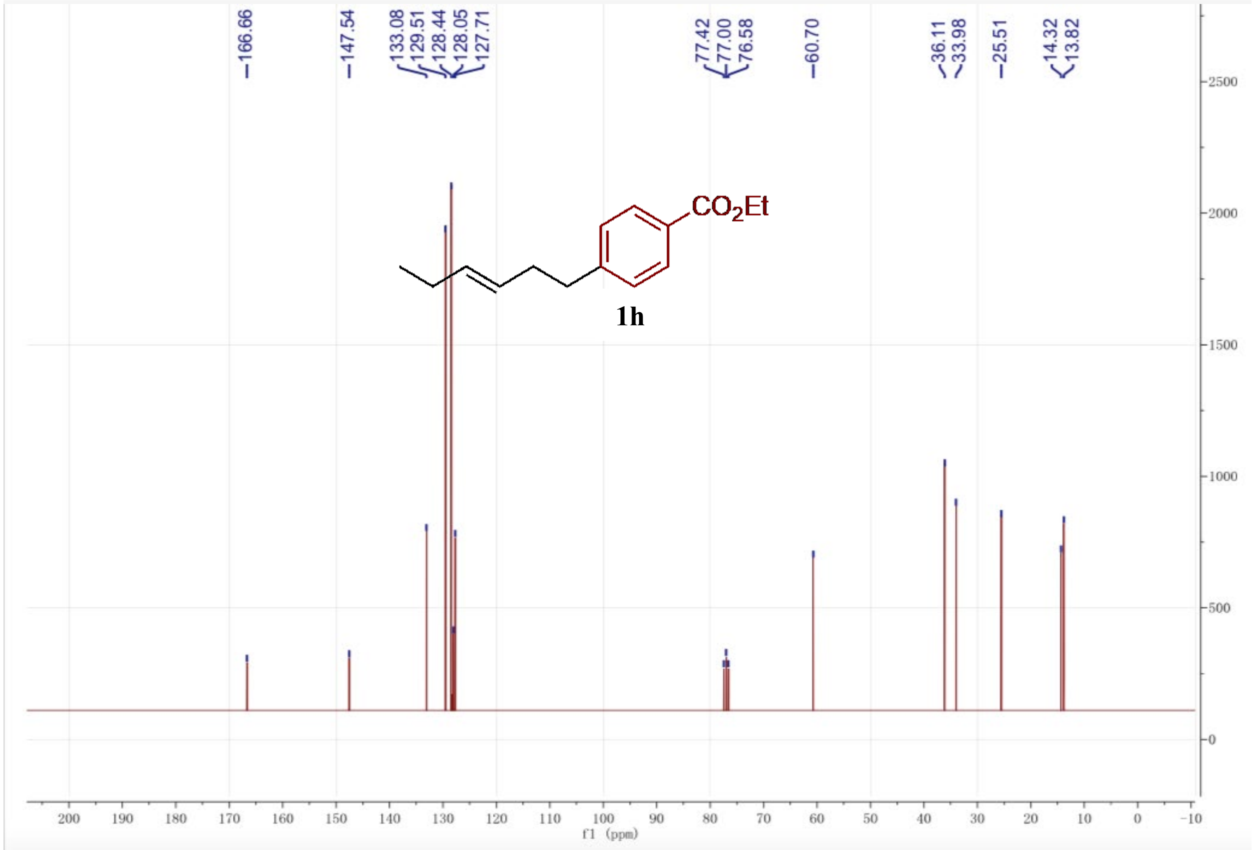
166.66
147.54
133.08
129.51
128.44
128.05
127.71
77.42
77.00
76.58
60.70
36.11
33.98
25.51
14.32
13.82
CO2Et
1h
f1 (ppm)

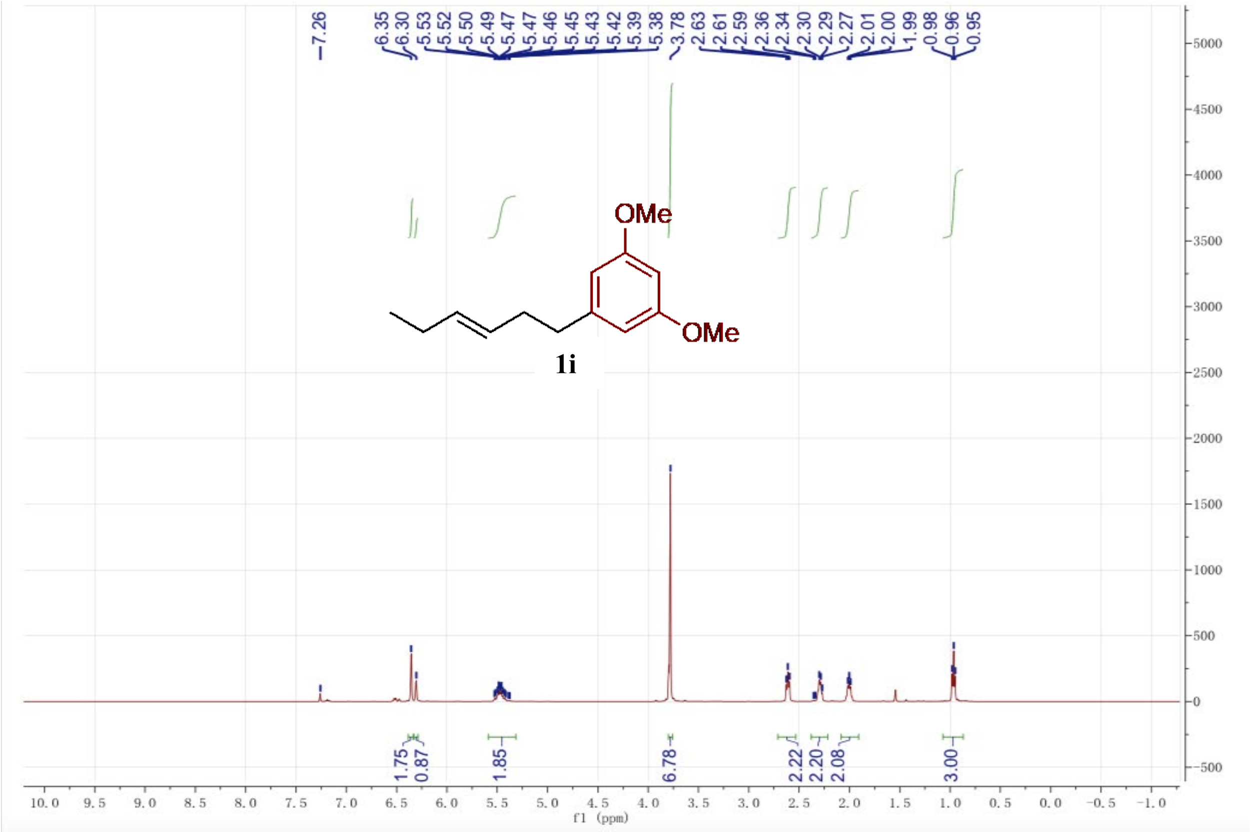

7.26
6.35
6.30
5.53
5.52
5.50
5.49
5.47
5.46
5.45
5.43
5.42
5.39
5.38
3.78
2.63
2.61
2.59
2.36
2.34
2.30
2.29
2.27
2.01
2.00
1.99
0.98
0.96
0.95
OMe
OMe
1i
1.75
0.87
1.85
6.78
2.22
2.20
2.08
3.00
f1 (ppm)

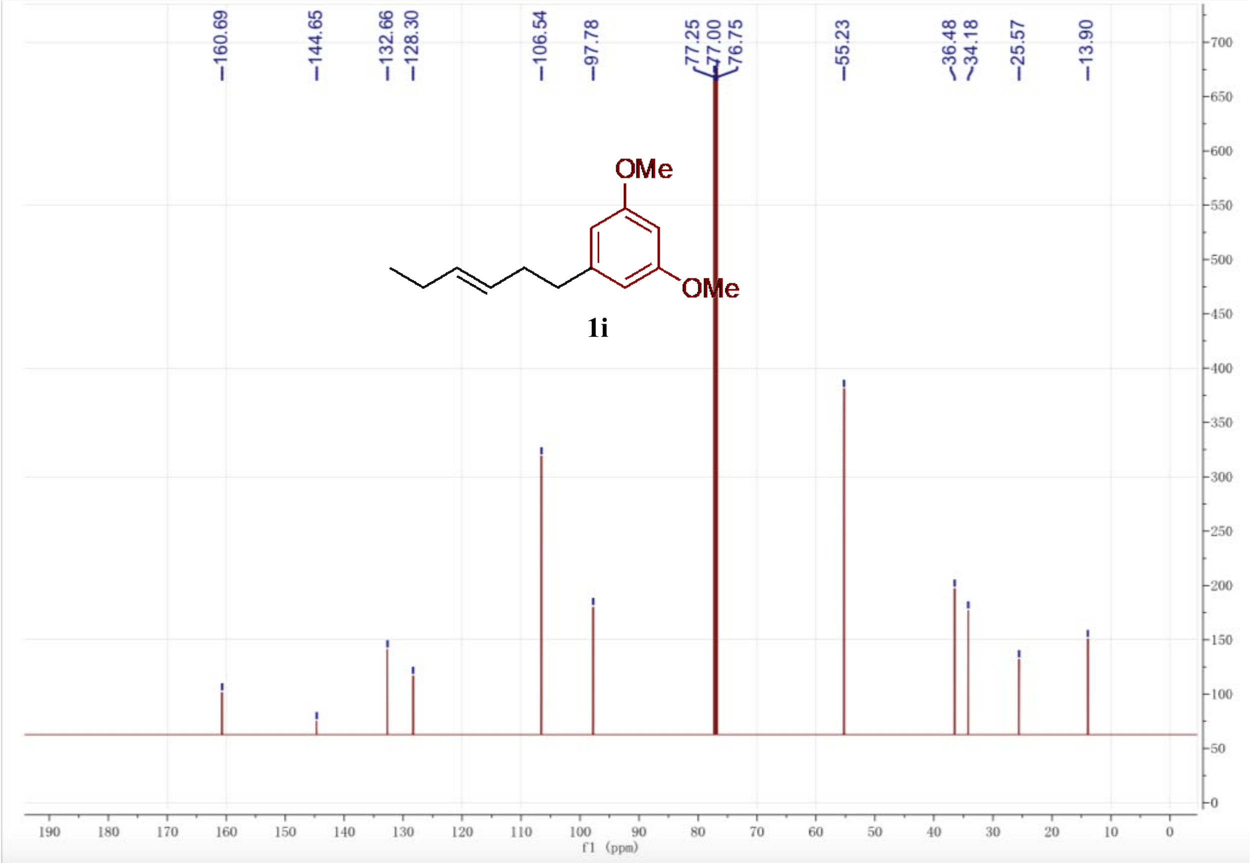

160.69
144.65
132.66
128.30
106.54
97.78
77.25
77.00
76.75
55.23
36.48
34.18
25.57
13.90
OMe
OMe
1i
f1 (ppm)

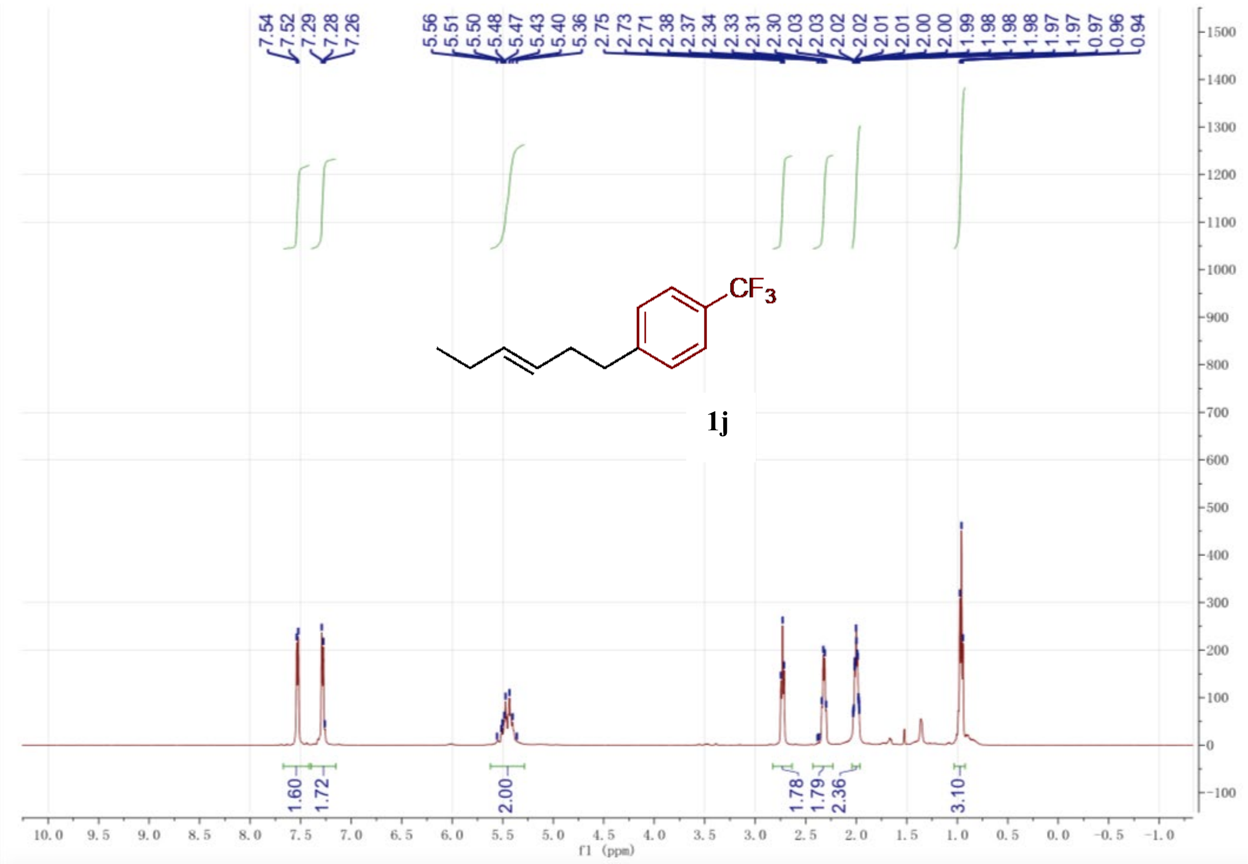
7.54
7.52
7.29
7.28
7.26
5.56
5.51
5.50
5.48
5.47
5.43
5.40
5.36
2.75
2.73
2.71
2.38
2.37
2.34
2.33
2.31
2.30
2.03
2.03
2.02
2.02
2.01
2.01
2.00
2.00
1.99
1.98
1.98
1.98
1.97
1.97
0.97
0.96
0.94
CF3
1j
1.60
1.72
2.00
1.78
1.79
2.36
3.10
f1 (ppm)

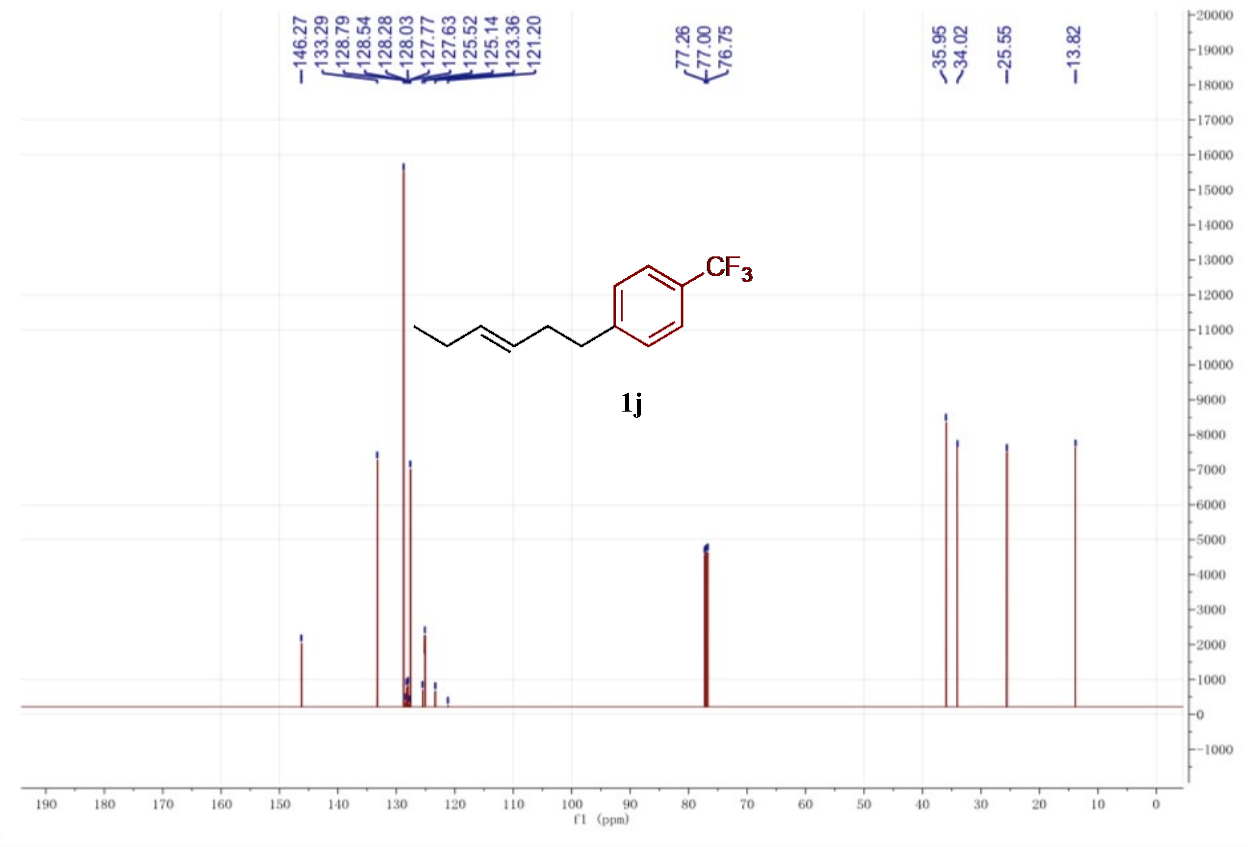
146.27
133.29
128.79
128.54
128.28
128.03
127.77
127.63
125.52
125.14
123.36
121.20
77.26
77.00
76.75
35.95
34.02
25.55
13.82
CF3
1j
f1 (ppm)

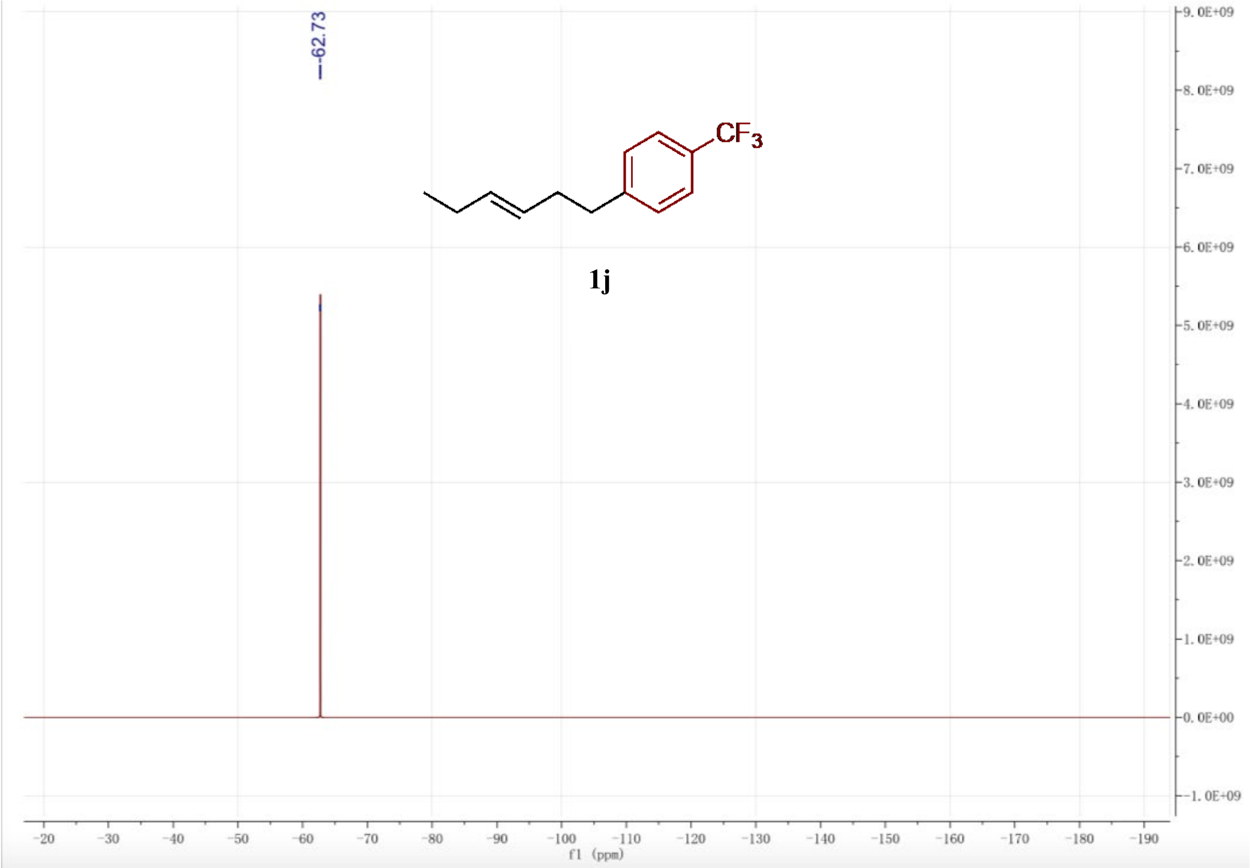

1j

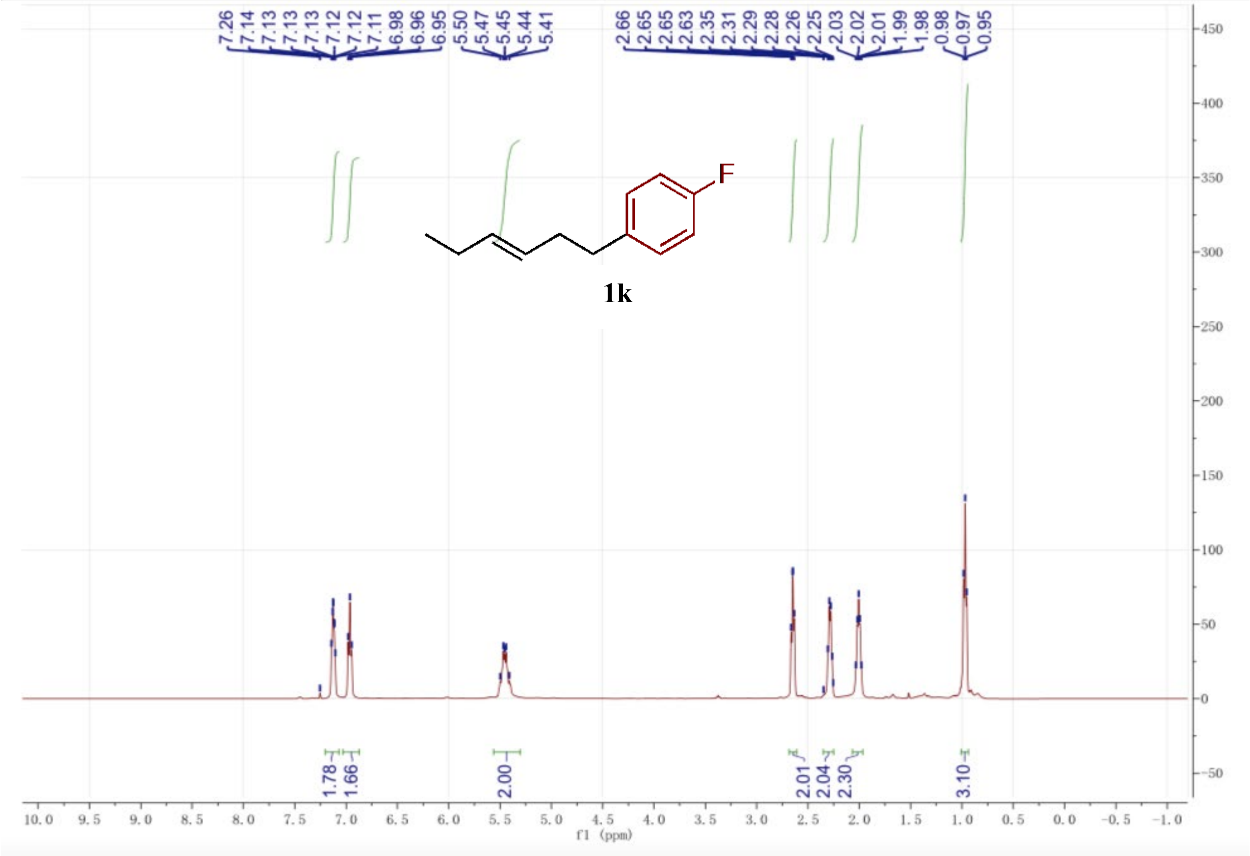

1k

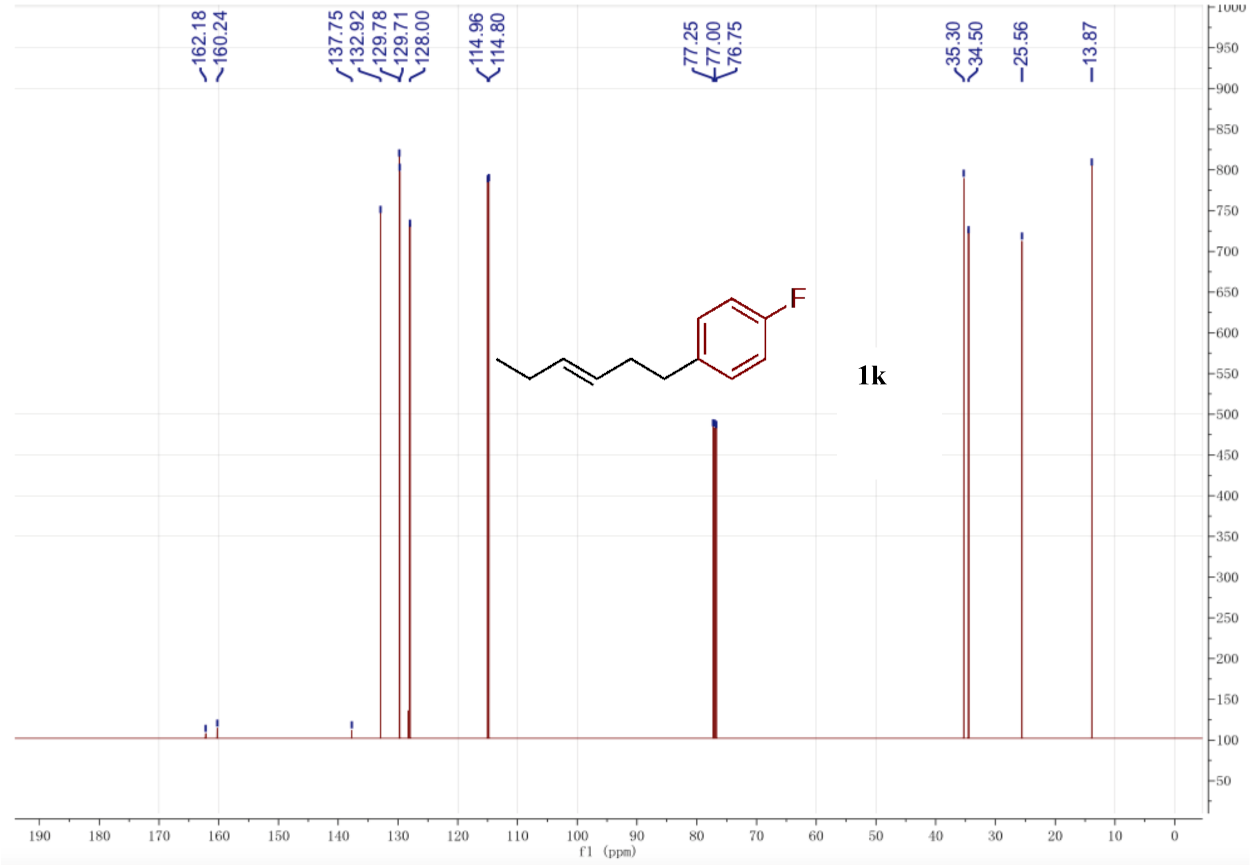
162.18
160.24
137.75
132.92
129.78
129.71
128.00
114.96
114.80
77.25
77.00
76.75
35.30
34.50
25.56
13.87
F
1k
f1 (ppm)

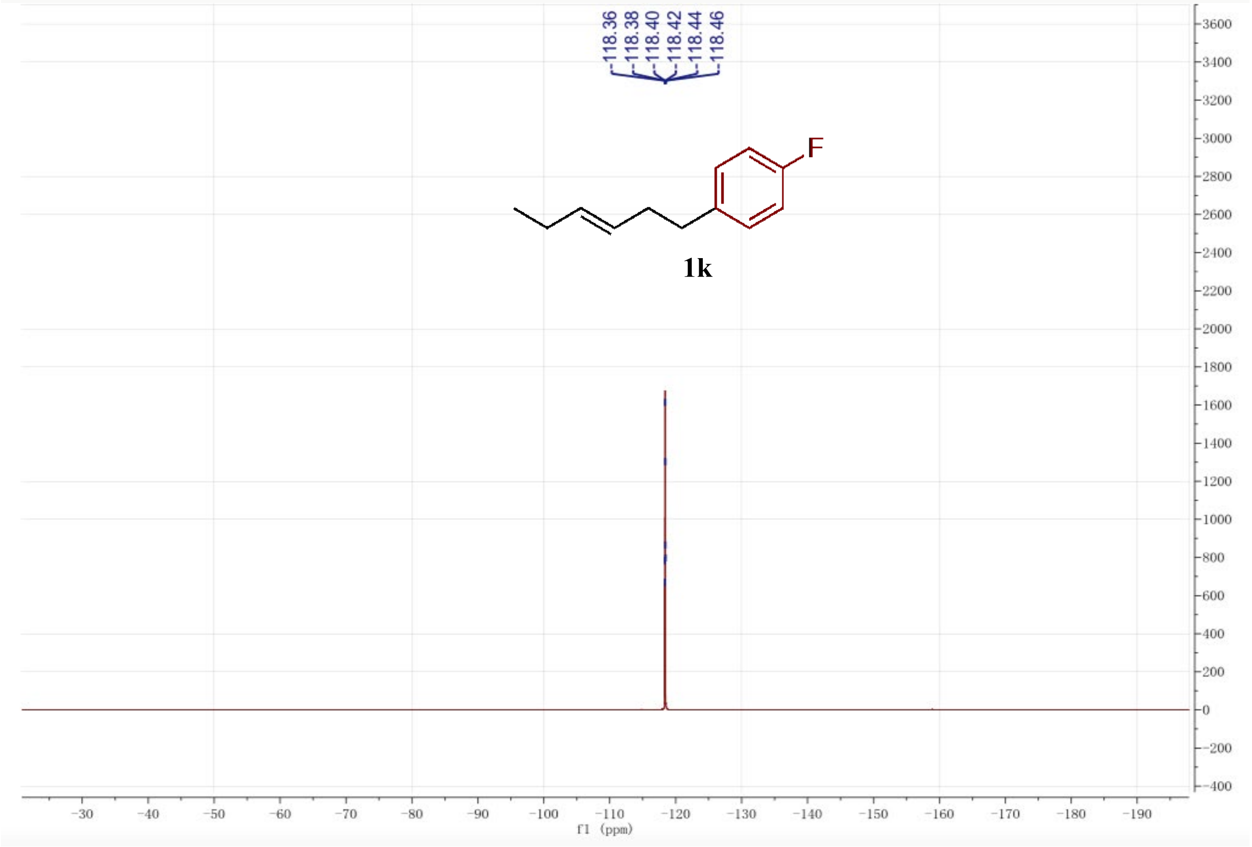
-118.36
-118.38
-118.40
-118.42
-118.44
-118.46
F
1k
f1 (ppm)

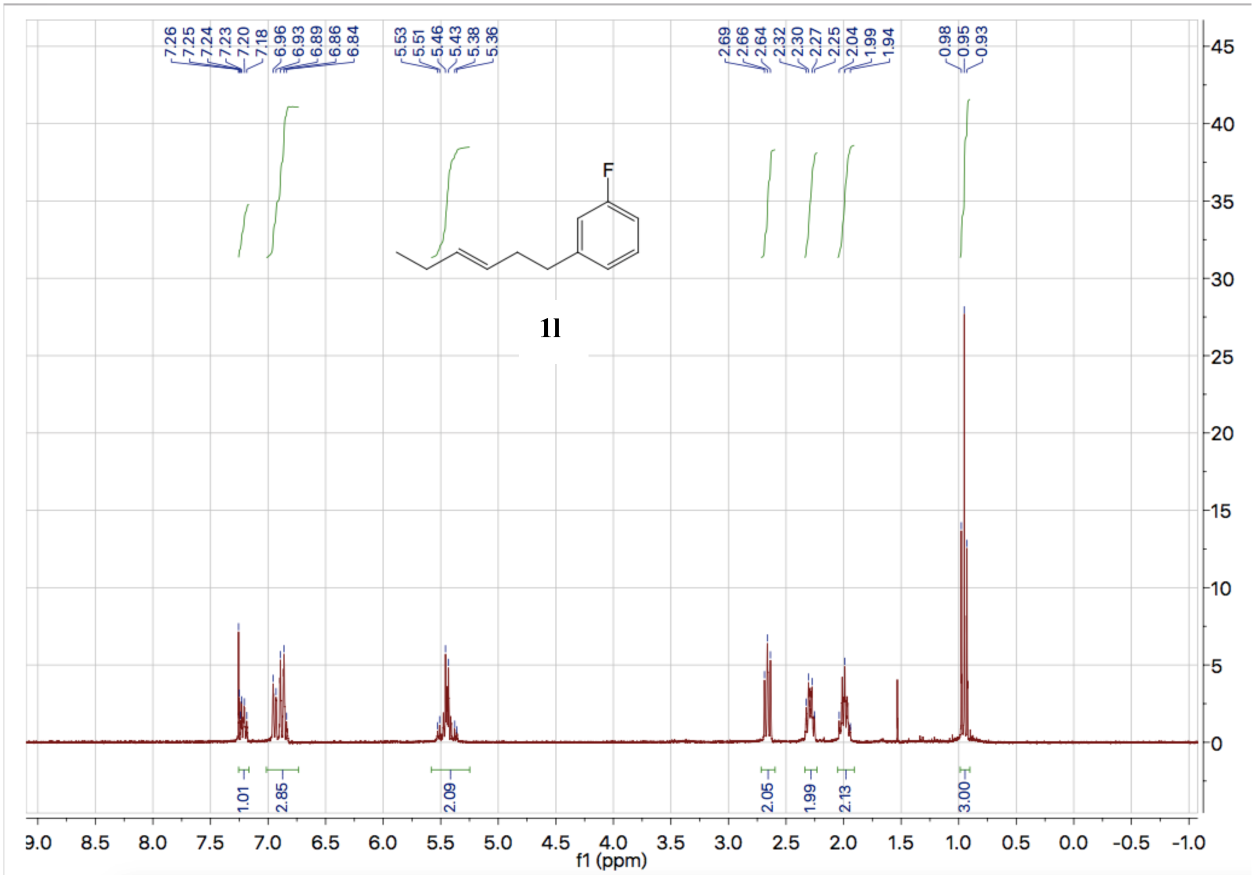
7.26
7.25
7.24
7.23
7.20
7.18
6.96
6.93
6.89
6.86
6.84
5.53
5.51
5.46
5.43
5.38
5.36
2.69
2.66
2.64
2.32
2.30
2.27
2.25
2.04
1.99
1.94
0.98
0.95
0.93
F
1l
1.01
2.85
2.09
2.05
1.99
2.13
3.00
f1 (ppm)

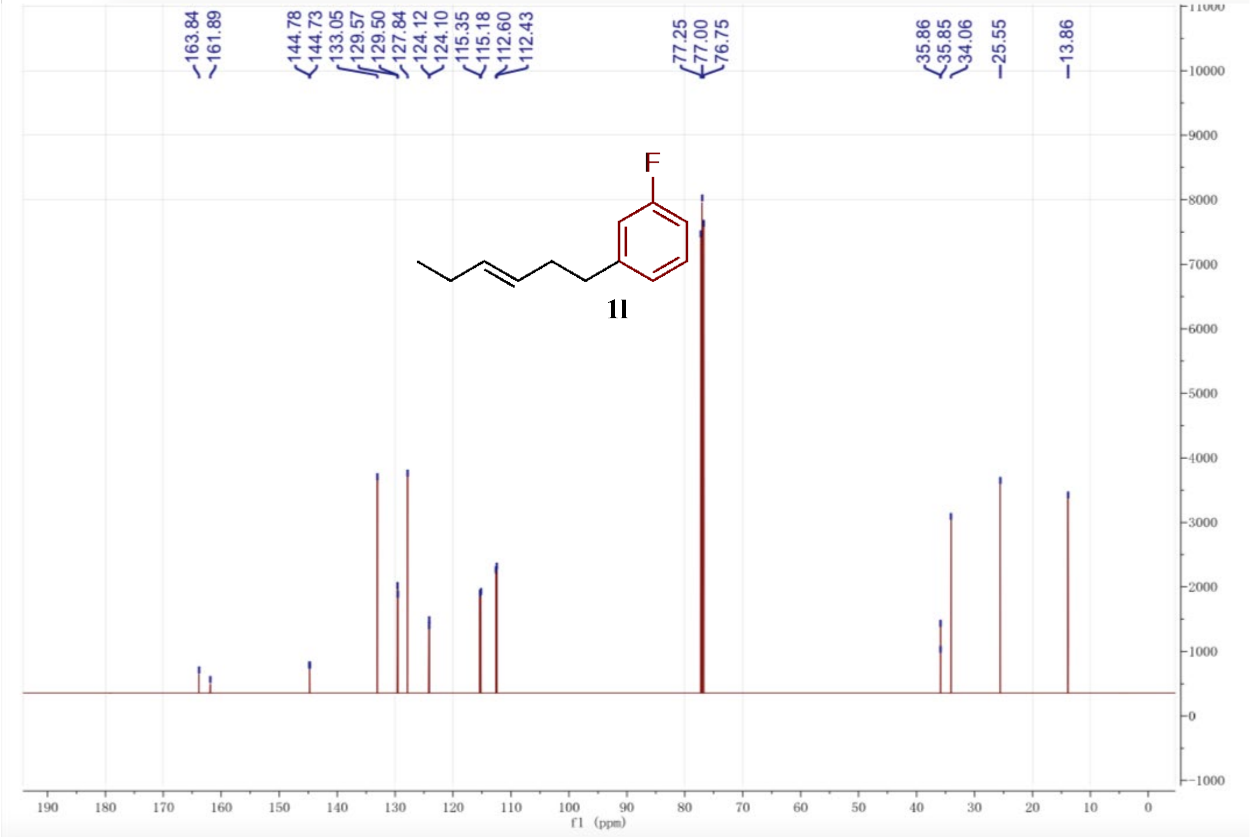
163.84
161.89
144.78
144.73
133.05
129.57
129.50
127.84
124.12
124.10
115.35
115.18
112.60
112.43
77.25
77.00
76.75
35.86
35.85
34.06
25.55
13.86
F
1l
f1 (ppm)

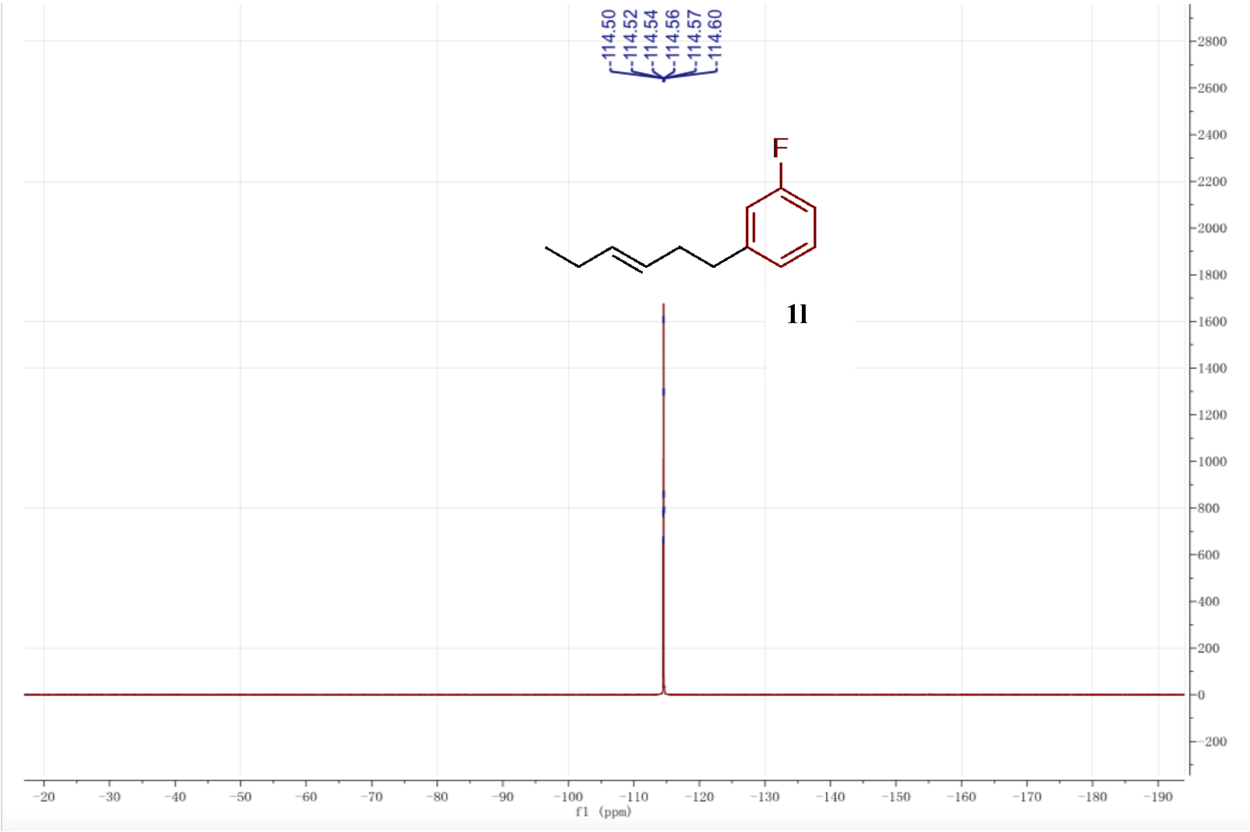
-114.50
-114.52
-114.54
-114.56
-114.57
-114.60
F
1l
f1 (ppm)

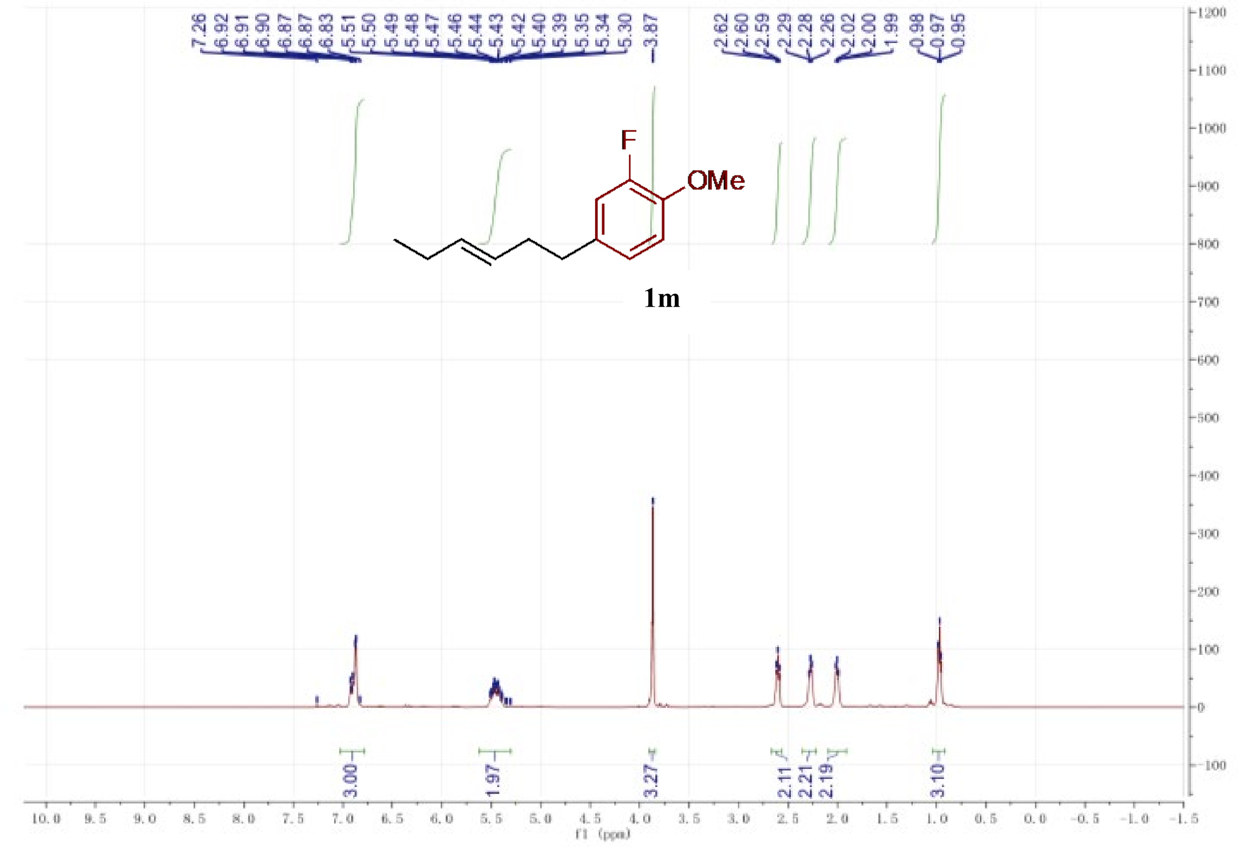
F
OMe
1m
3.00
1.97
3.27
2.11
2.21
2.19
3.10
f1 (ppm)

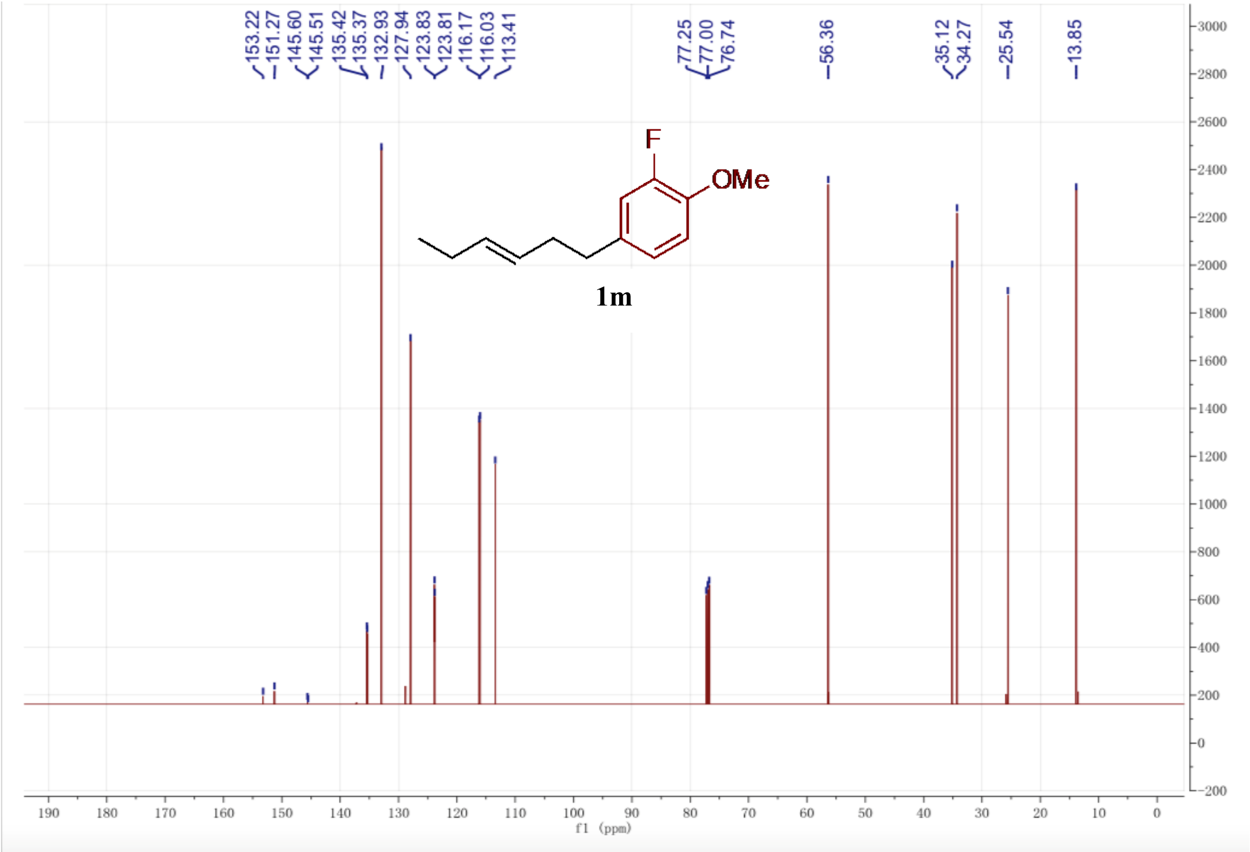
153.22
151.27
145.60
145.51
135.42
135.37
132.93
127.94
123.83
123.81
116.17
116.03
113.41
77.25
77.00
76.74
56.36
35.12
34.27
25.54
13.85
F
OMe
1m
f1 (ppm)

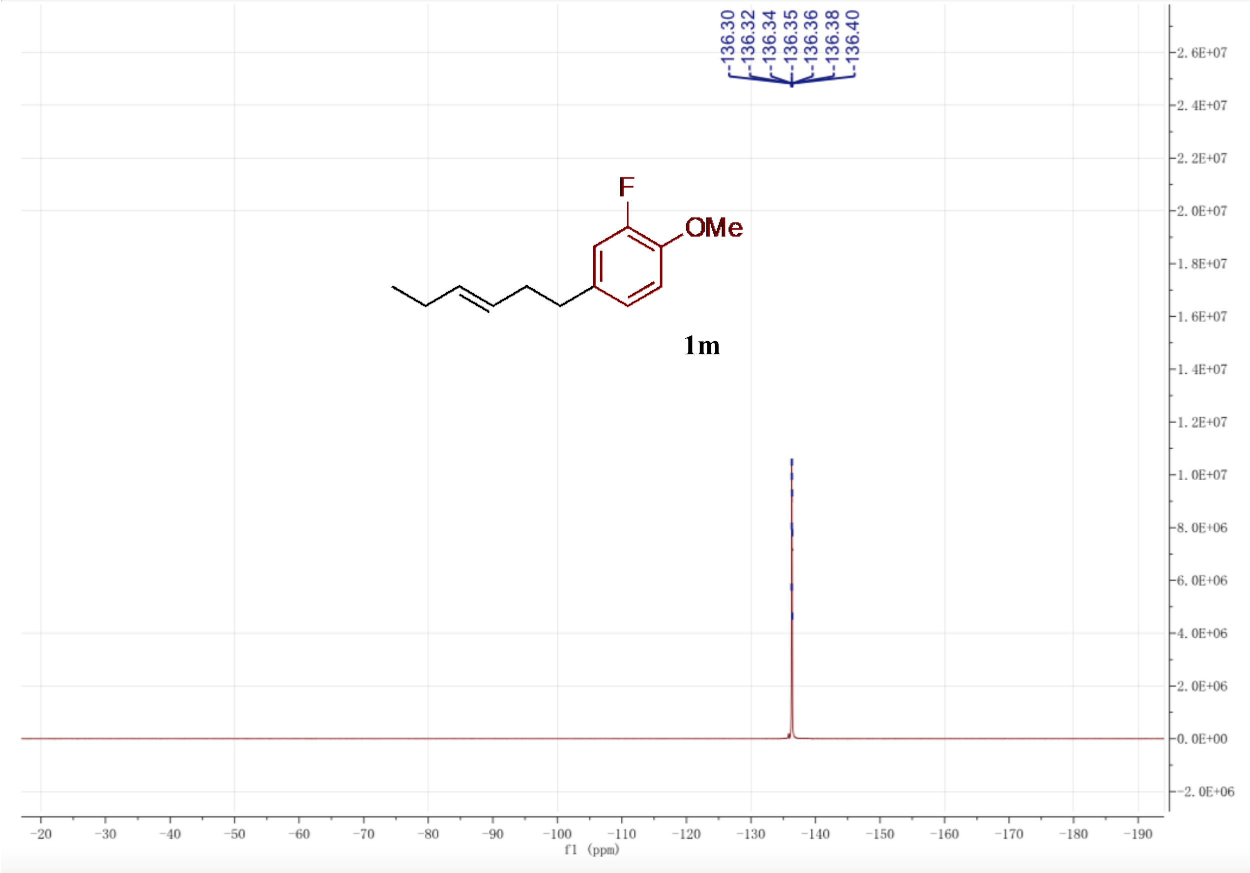
-136.30
-136.32
-136.34
-136.35
-136.36
-136.38
-136.40
F
OMe
1m
f1 (ppm)

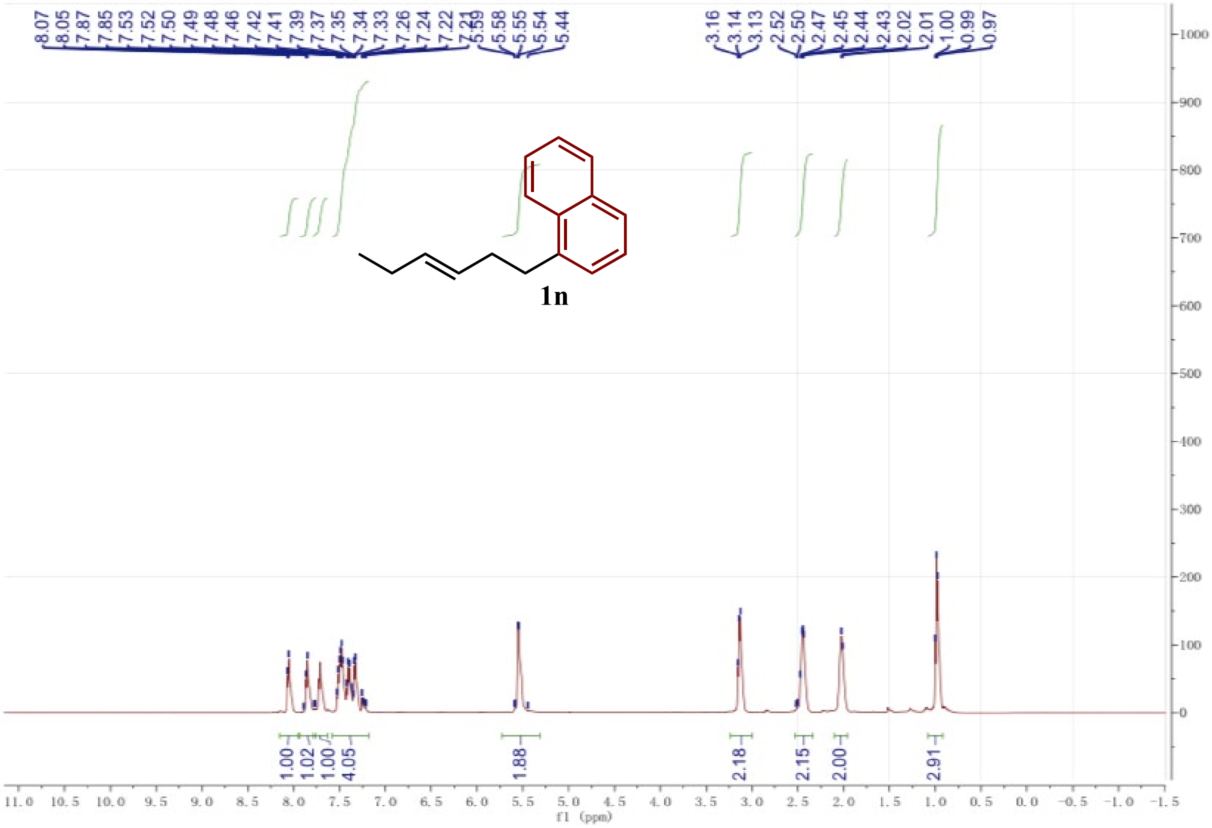
8.07
8.05
7.87
7.85
7.53
7.52
7.50
7.49
7.48
7.46
7.42
7.41
7.39
7.37
7.35
7.34
7.33
7.26
7.24
7.22
7.21
5.59
5.58
5.55
5.54
5.44
3.16
3.14
3.13
2.52
2.50
2.47
2.45
2.44
2.43
2.02
2.01
1.00
0.99
0.97
1n
1.00
1.02
1.00
4.05
1.88
2.18
2.15
2.00
2.91
f1 (ppm)

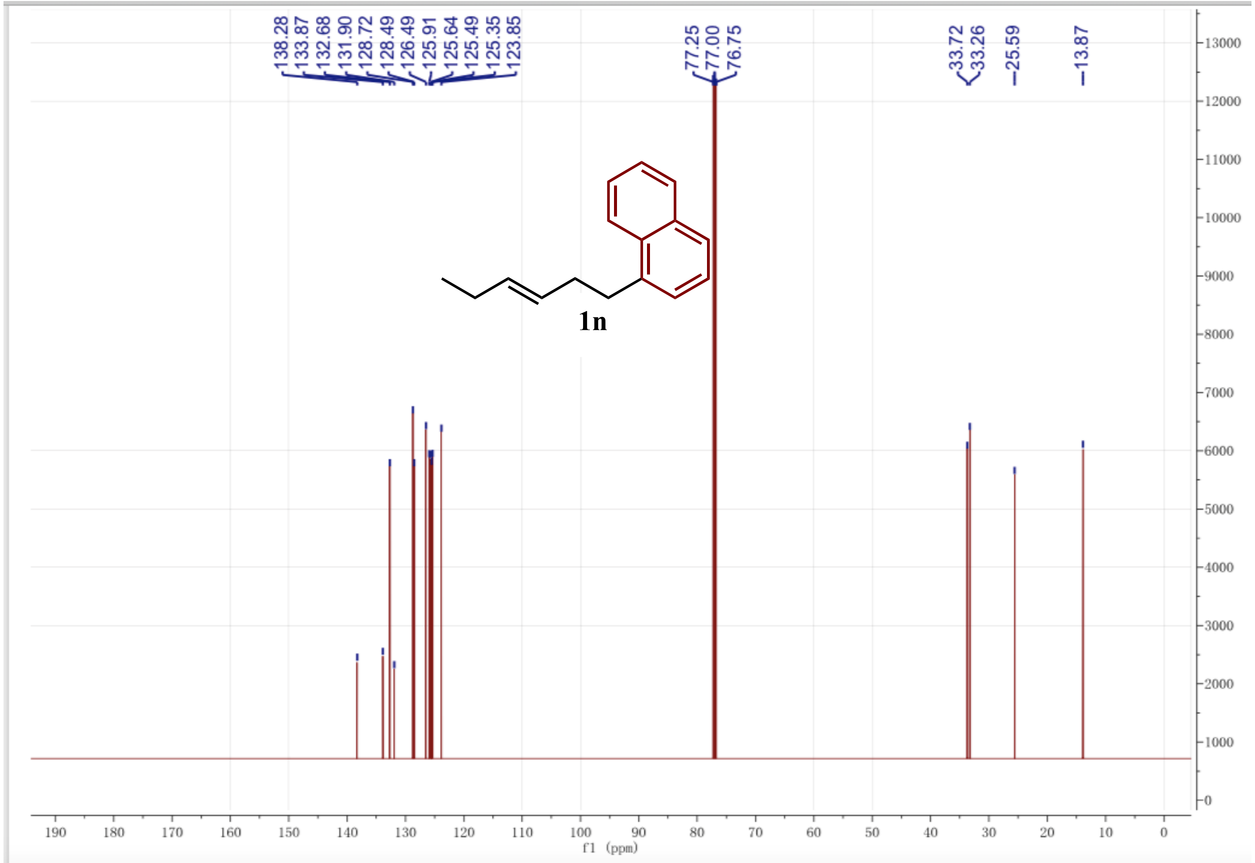
138.28
133.87
132.68
131.90
128.72
128.49
126.49
125.91
125.64
125.49
125.35
123.85
77.25
77.00
76.75
33.72
33.26
25.59
13.87
1n
f1 (ppm)

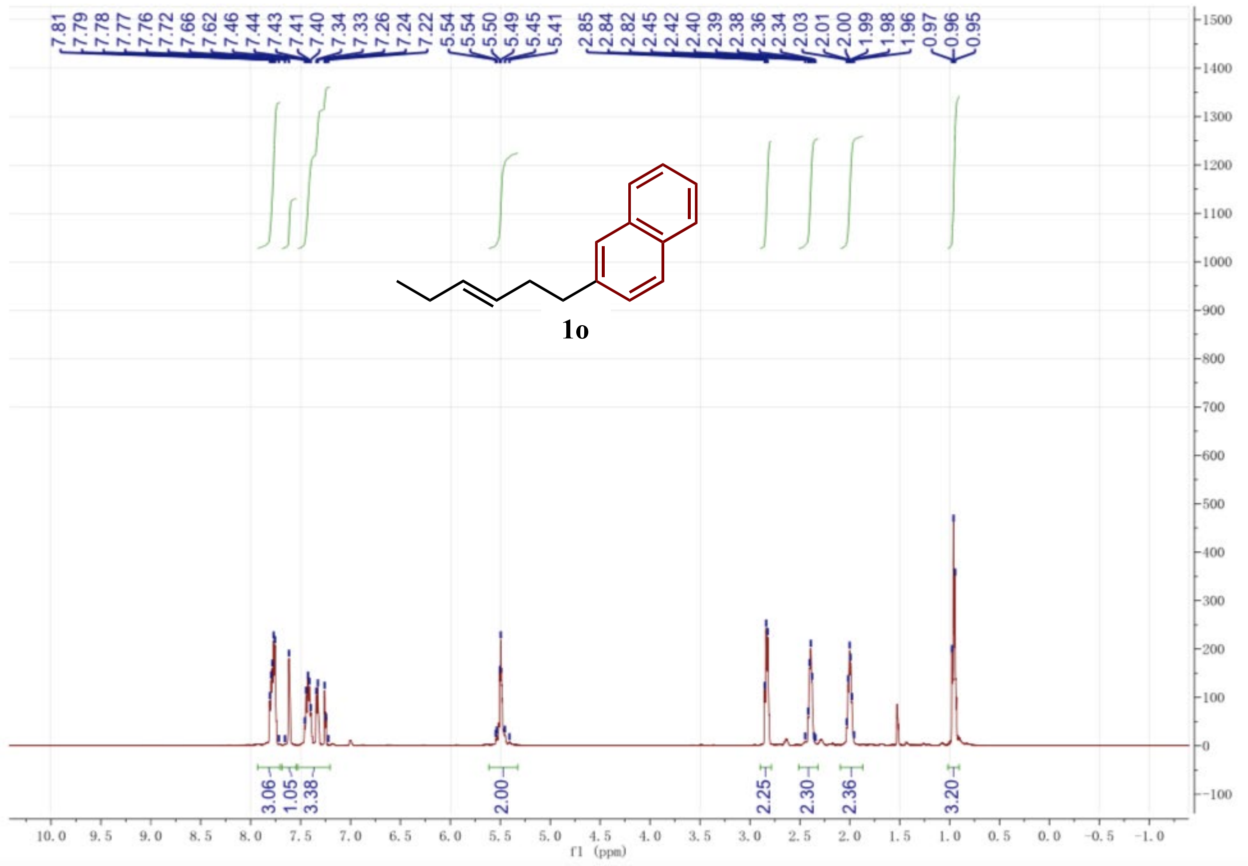
7.81 7.79 7.78 7.77 7.76 7.72 7.66 7.62 7.46 7.44 7.43 7.41 7.40 7.34 7.33 7.26 7.24 7.22 5.54 5.54 5.50 5.49 5.45 5.41 2.85 2.84 2.82 2.45 2.42 2.40 2.39 2.38 2.36 2.34 2.03 2.01 2.00 1.99 1.98 1.96 0.97 0.96 0.95
1o
3.06 1.05 3.38 2.00 2.25 2.30 2.36 3.20
f1 (ppm)

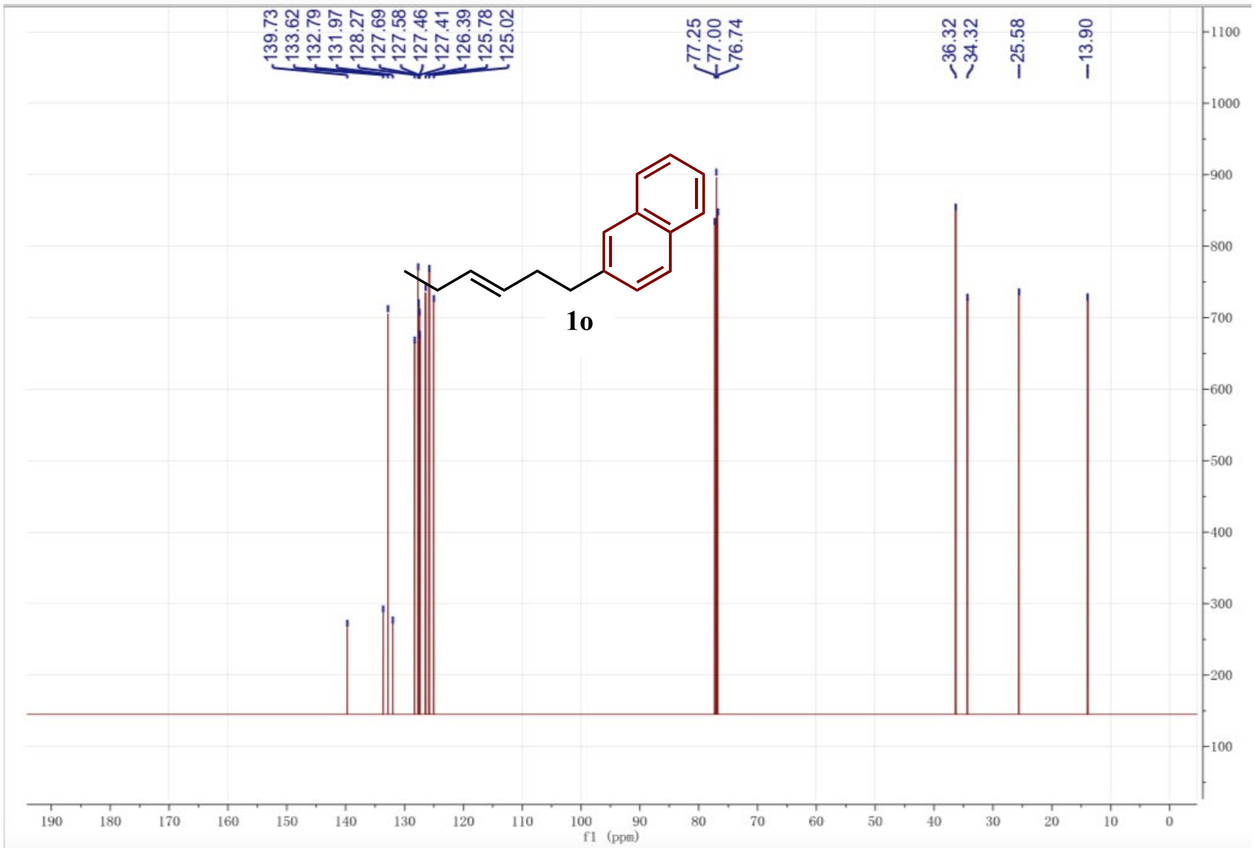
139.73 133.62 132.79 131.97 128.27 127.69 127.58 127.46 127.41 126.39 125.78 125.02
77.25 77.00 76.74
36.32 34.32 25.58 13.90
1o
f1 (ppm)

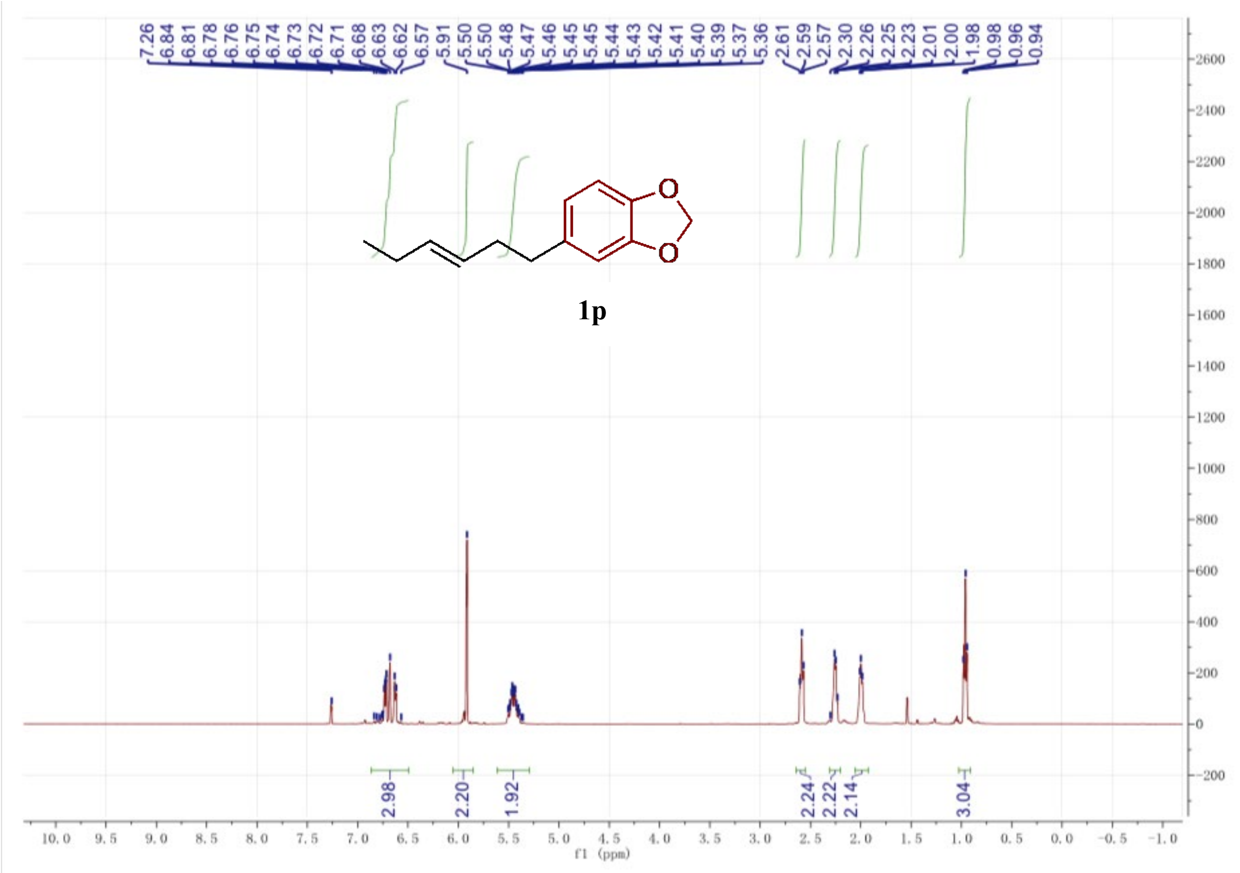
7.26
6.84
6.81
6.78
6.76
6.75
6.74
6.73
6.72
6.71
6.68
6.63
6.62
6.57
5.91
5.50
5.50
5.48
5.47
5.46
5.45
5.45
5.44
5.43
5.42
5.41
5.40
5.39
5.37
5.36
2.61
2.59
2.57
2.30
2.26
2.25
2.23
2.01
2.00
1.98
0.98
0.96
0.94
1p
2.98
2.20
1.92
2.24
2.22
2.14
3.04
f1 (ppm)

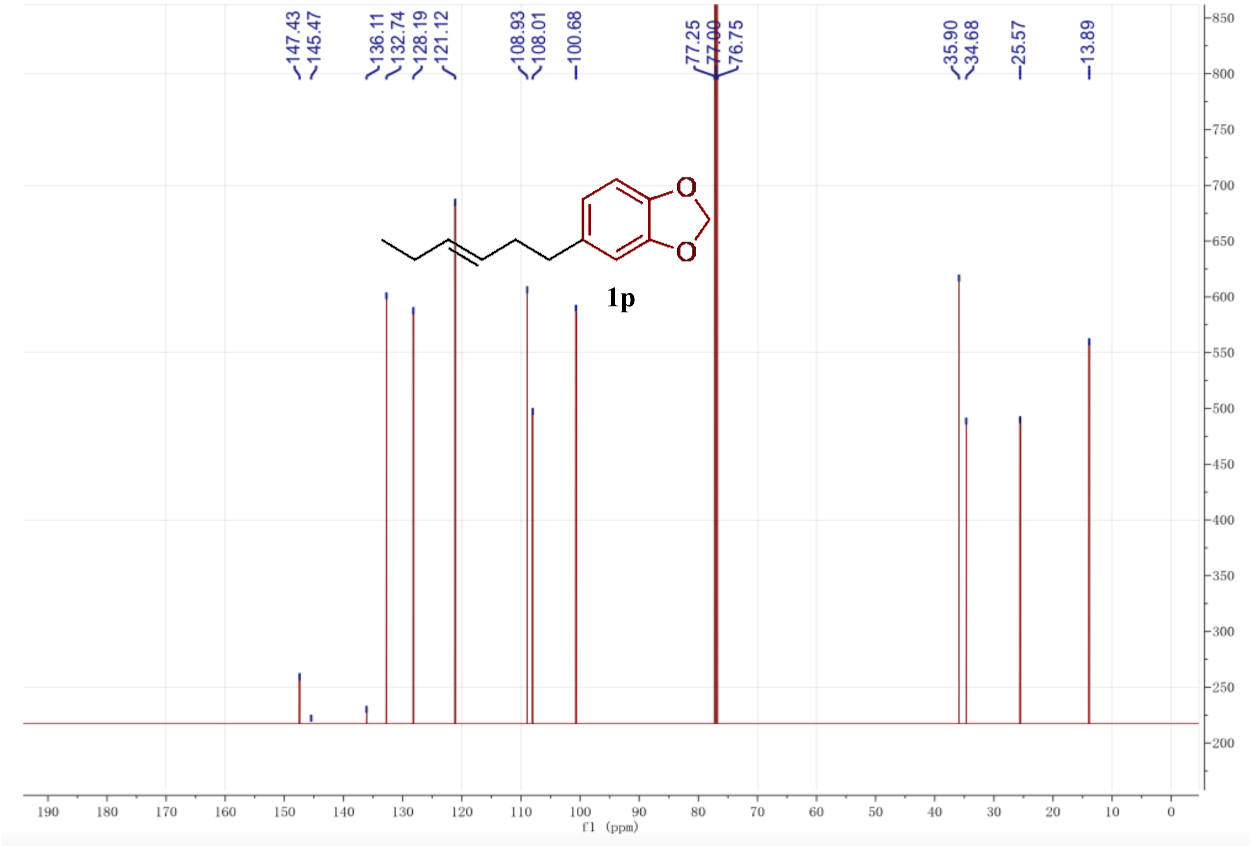
147.43
145.47
136.11
132.74
128.19
121.12
108.93
108.01
100.68
77.25
77.00
76.75
35.90
34.68
25.57
13.89
1p
f1 (ppm)

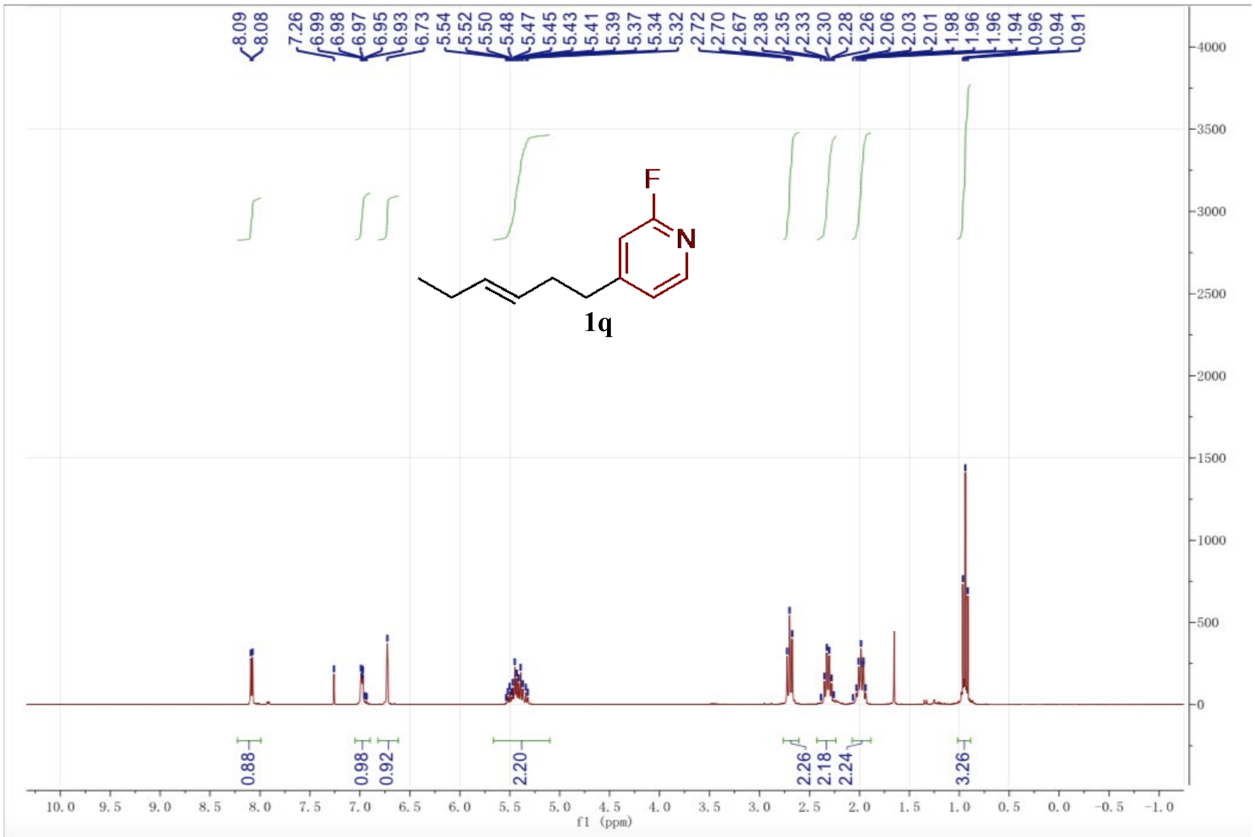
8.09
8.08
7.26
6.99
6.98
6.97
6.95
6.93
6.73
5.54
5.52
5.50
5.48
5.47
5.45
5.43
5.41
5.39
5.37
5.34
5.32
2.72
2.70
2.67
2.38
2.35
2.33
2.30
2.28
2.26
2.06
2.03
2.01
1.98
1.96
1.94
0.96
0.94
0.91
F
N
1q
0.88
0.98
0.92
2.20
2.26
2.18
2.24
3.26
f1 (ppm)

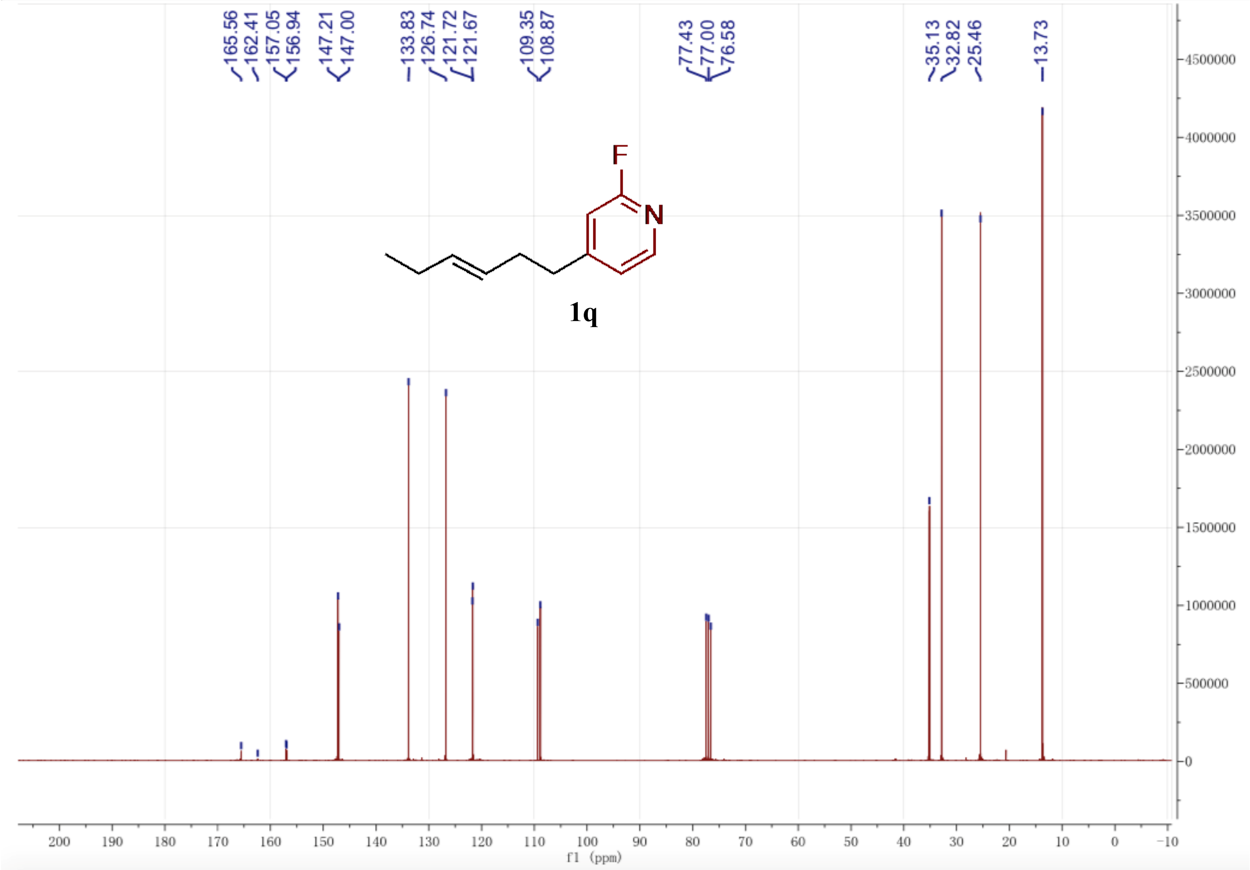
165.56
162.41
157.05
156.94
147.21
147.00
133.83
126.74
121.72
121.67
109.35
108.87
77.43
77.00
76.58
35.13
32.82
25.46
13.73
F
N
1q
f1 (ppm)

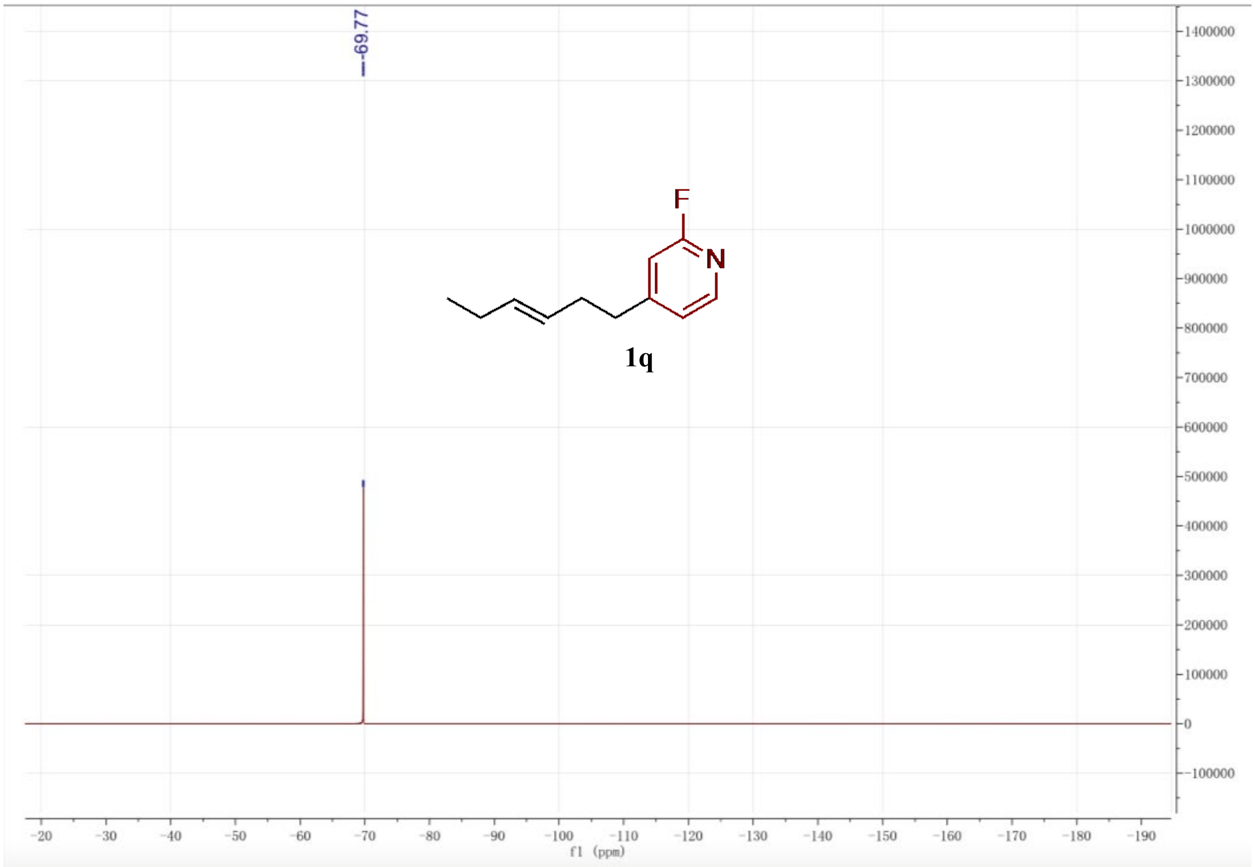

-69.77
1q
f1 (ppm)

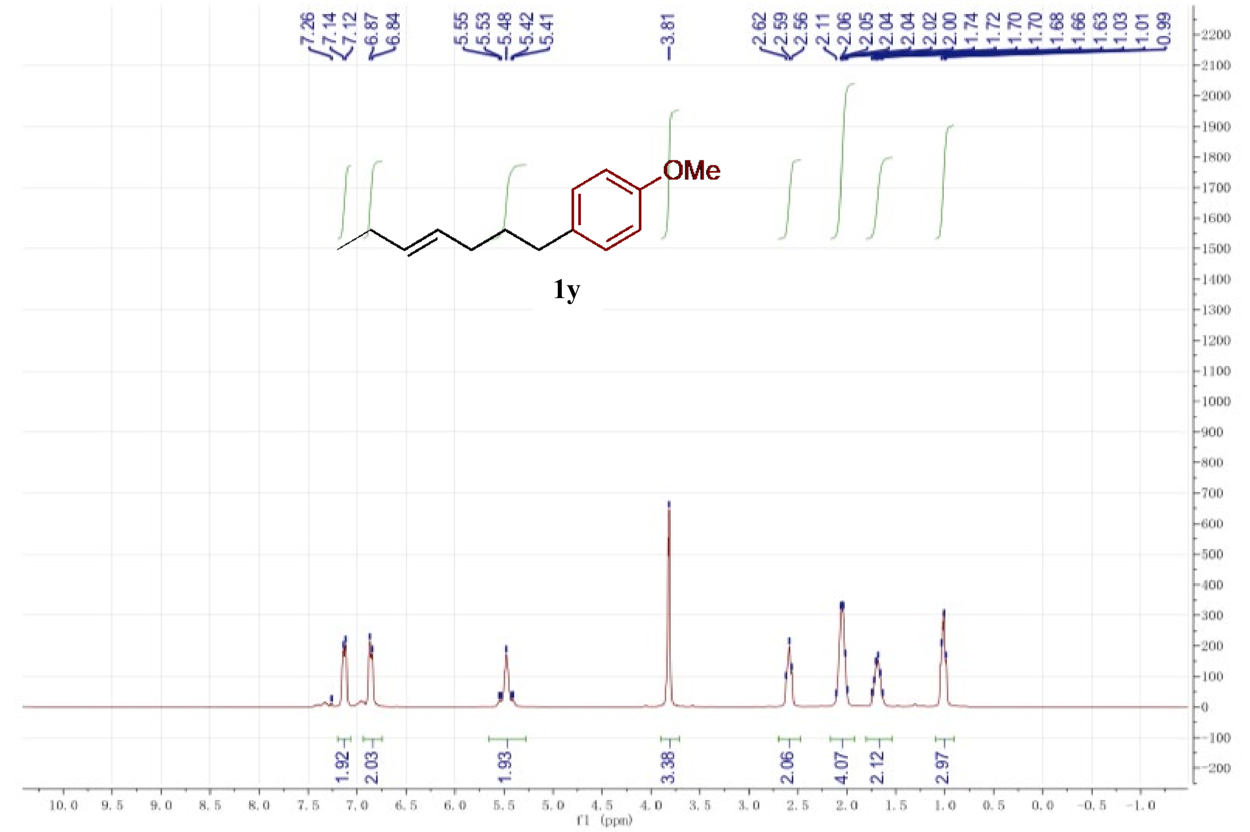

OMe
1y
f1 (ppm)

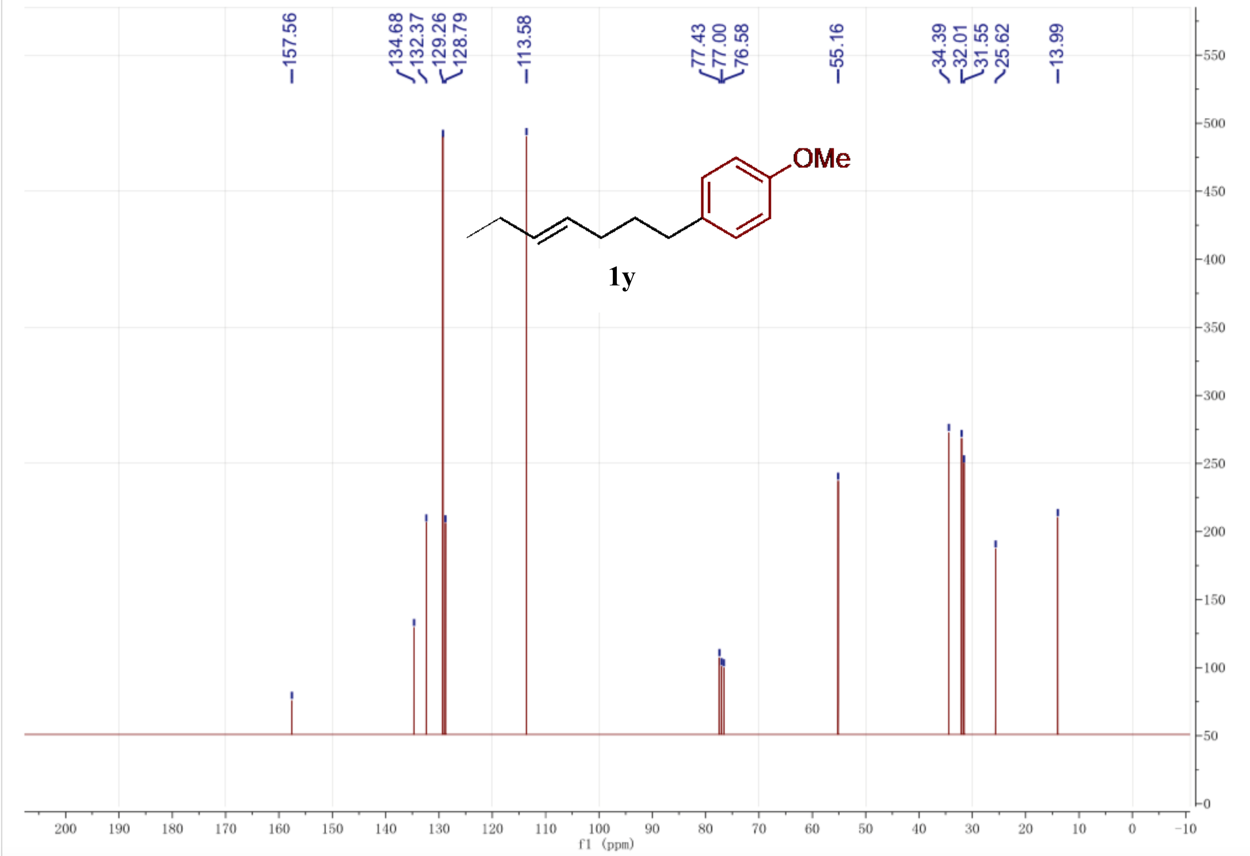

157.56
134.68
132.37
129.26
128.79
113.58
77.43
77.00
76.58
55.16
34.39
32.01
31.55
25.62
13.99
OMe
1y
f1 (ppm)

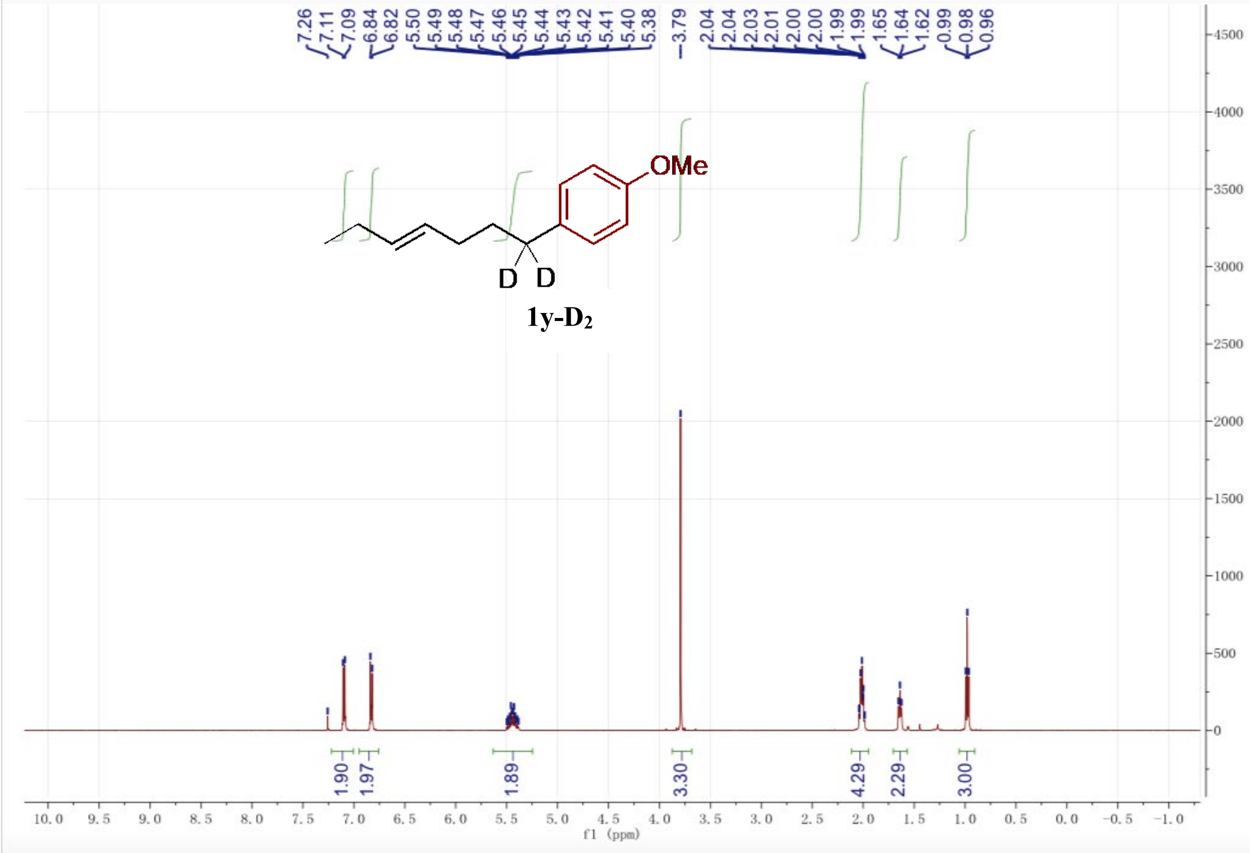

7.26
7.11
7.09
6.84
6.82
5.50
5.49
5.48
5.47
5.46
5.45
5.44
5.43
5.42
5.41
5.40
5.38
3.79
2.04
2.04
2.03
2.01
2.00
2.00
1.99
1.65
1.64
1.62
0.99
0.98
0.96
OMe
D D
1y-D2
1.90
1.97
1.89
3.30
4.29
2.29
3.00
f1 (ppm)

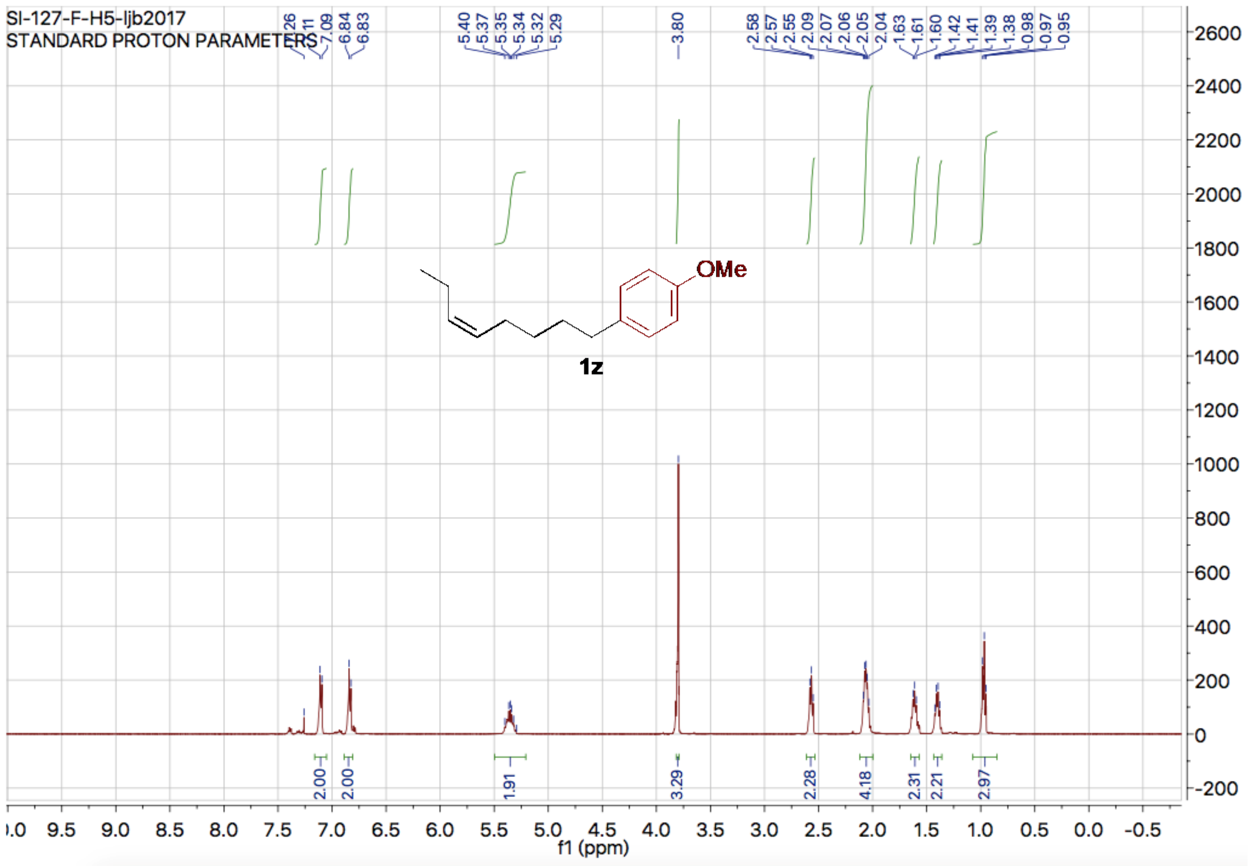
SI-127-F-H5-ljb2017
STANDARD PROTON PARAMETERS
OMe
1z
f1 (ppm)

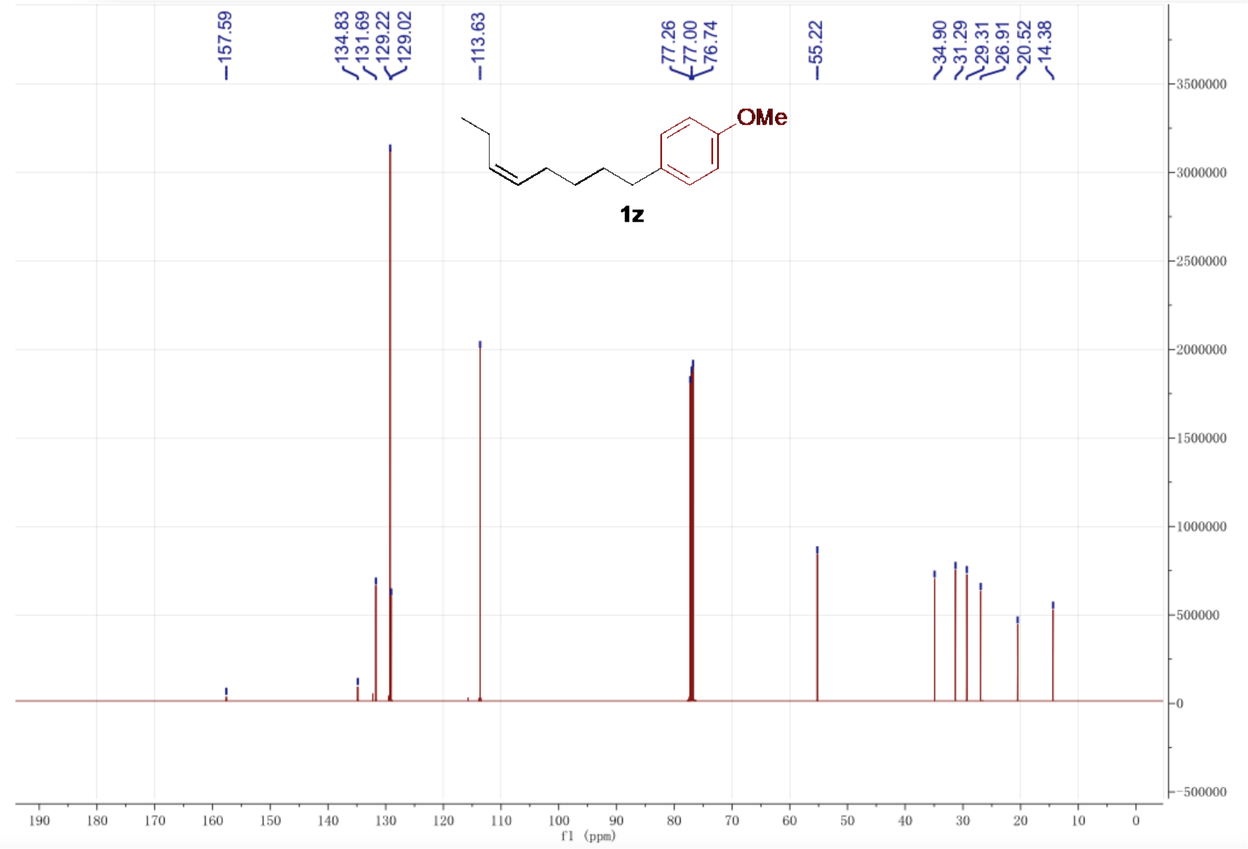
OMe
1z
f1 (ppm)

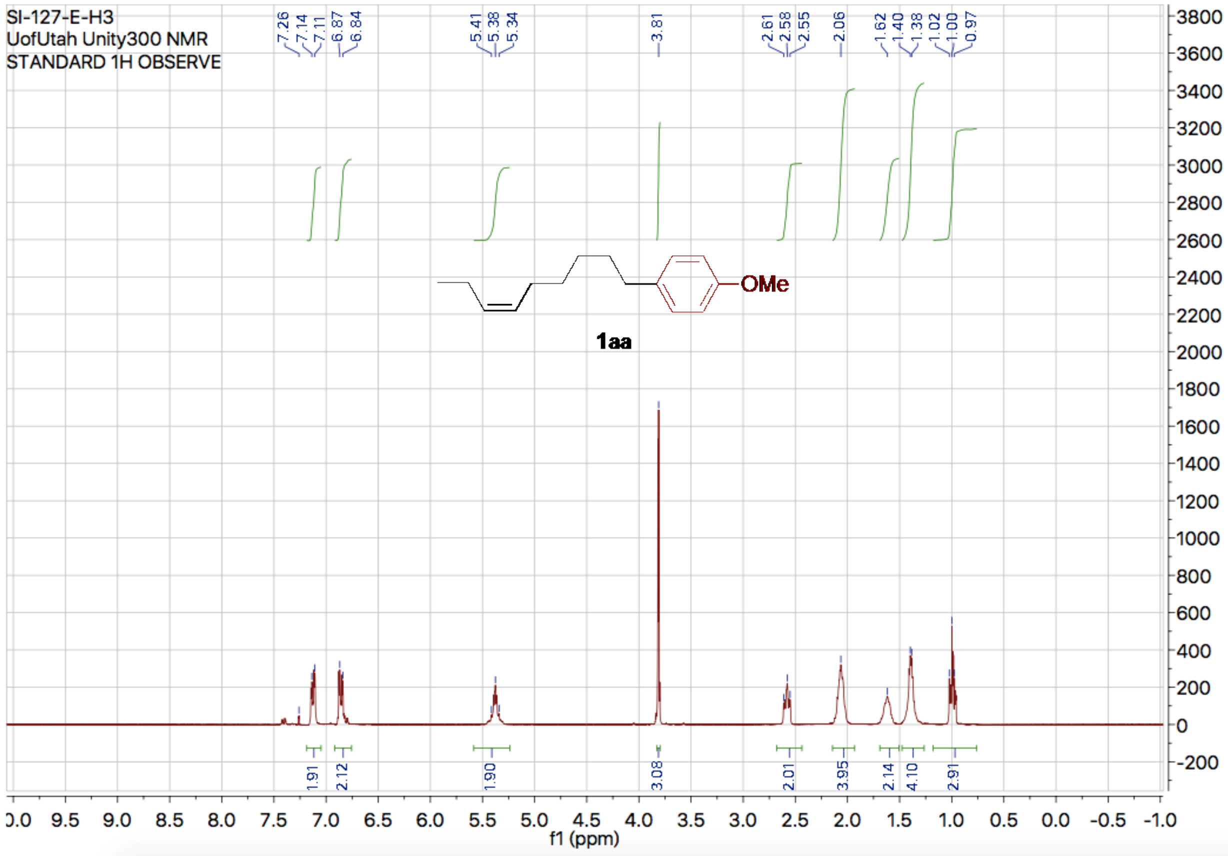
SI-127-E-H3
UofUtah Unity300 NMR
STANDARD 1H OBSERVE
OMe
1aa
f1 (ppm)

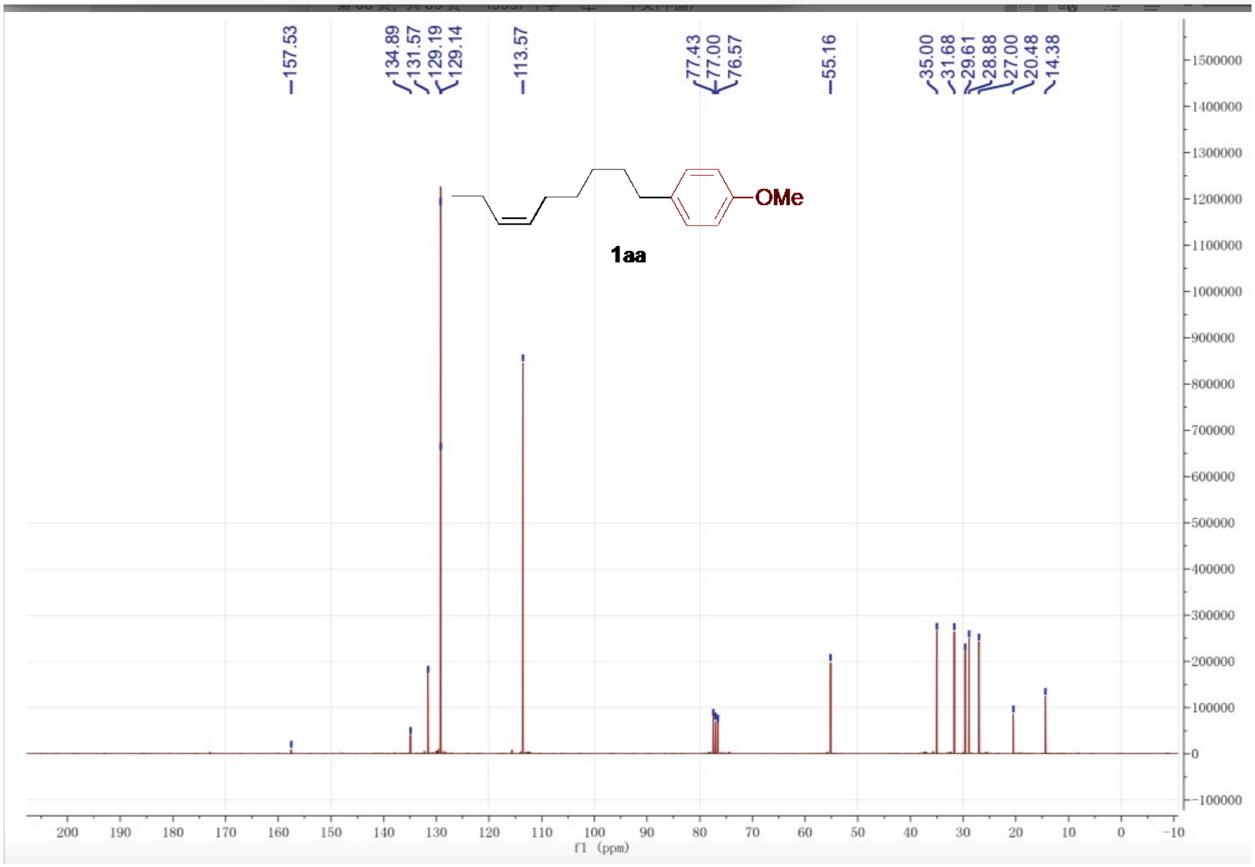
OMe
1aa
f1 (ppm)

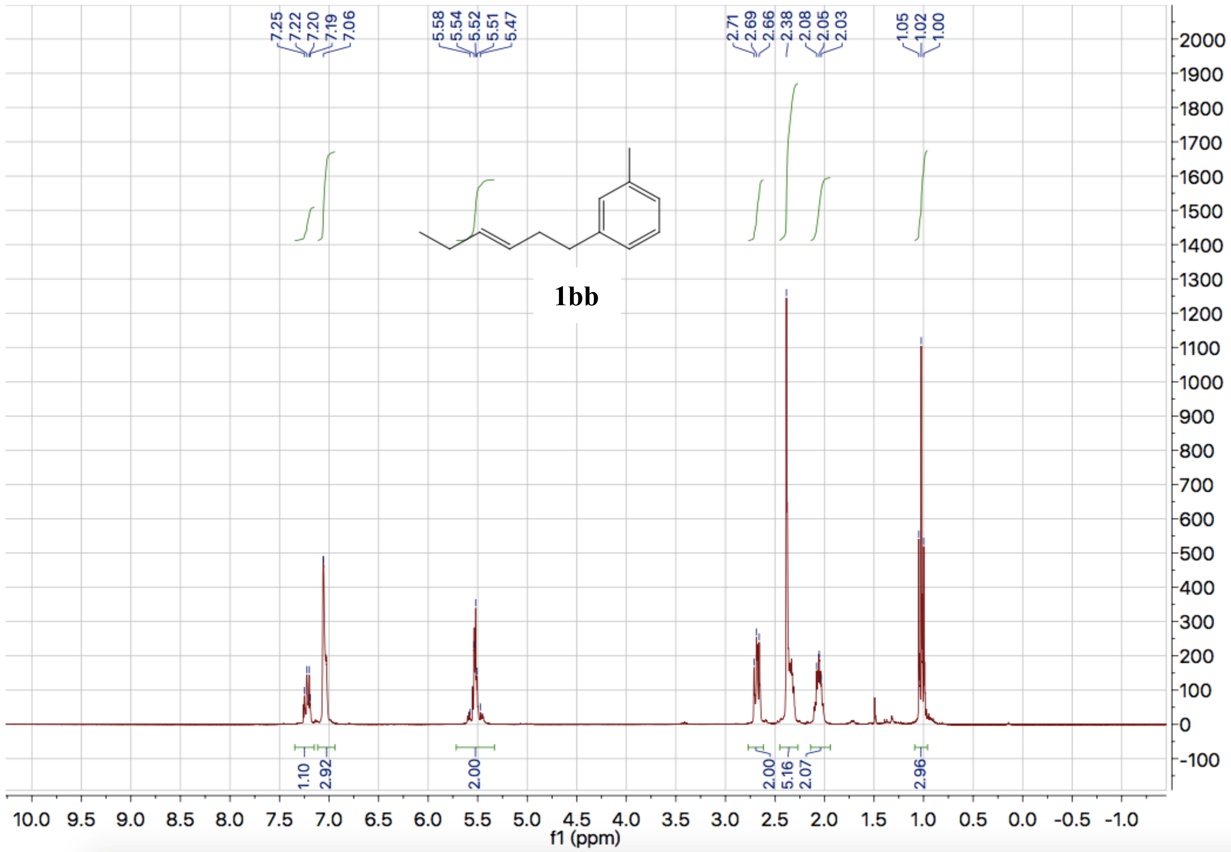

7.25
7.22
7.20
7.19
7.06
5.58
5.54
5.52
5.51
5.47
2.71
2.69
2.66
2.38
2.08
2.05
2.03
1.05
1.02
1.00
1bb
1.10
2.92
2.00
2.00
5.16
2.07
2.96
f1 (ppm)

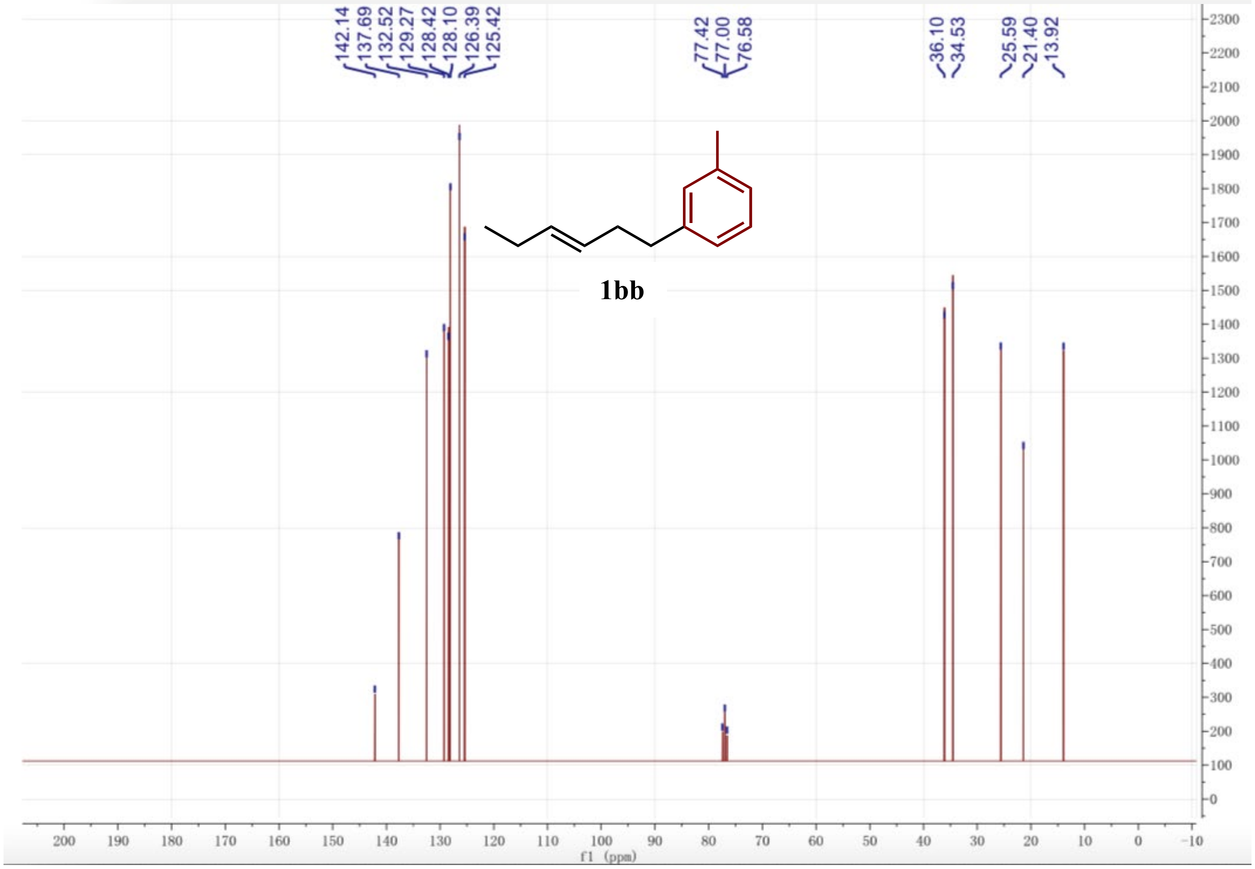

142.14
137.69
132.52
129.27
128.42
128.10
126.39
125.42
77.42
77.00
76.58
36.10
34.53
25.59
21.40
13.92
1bb
f1 (ppm)

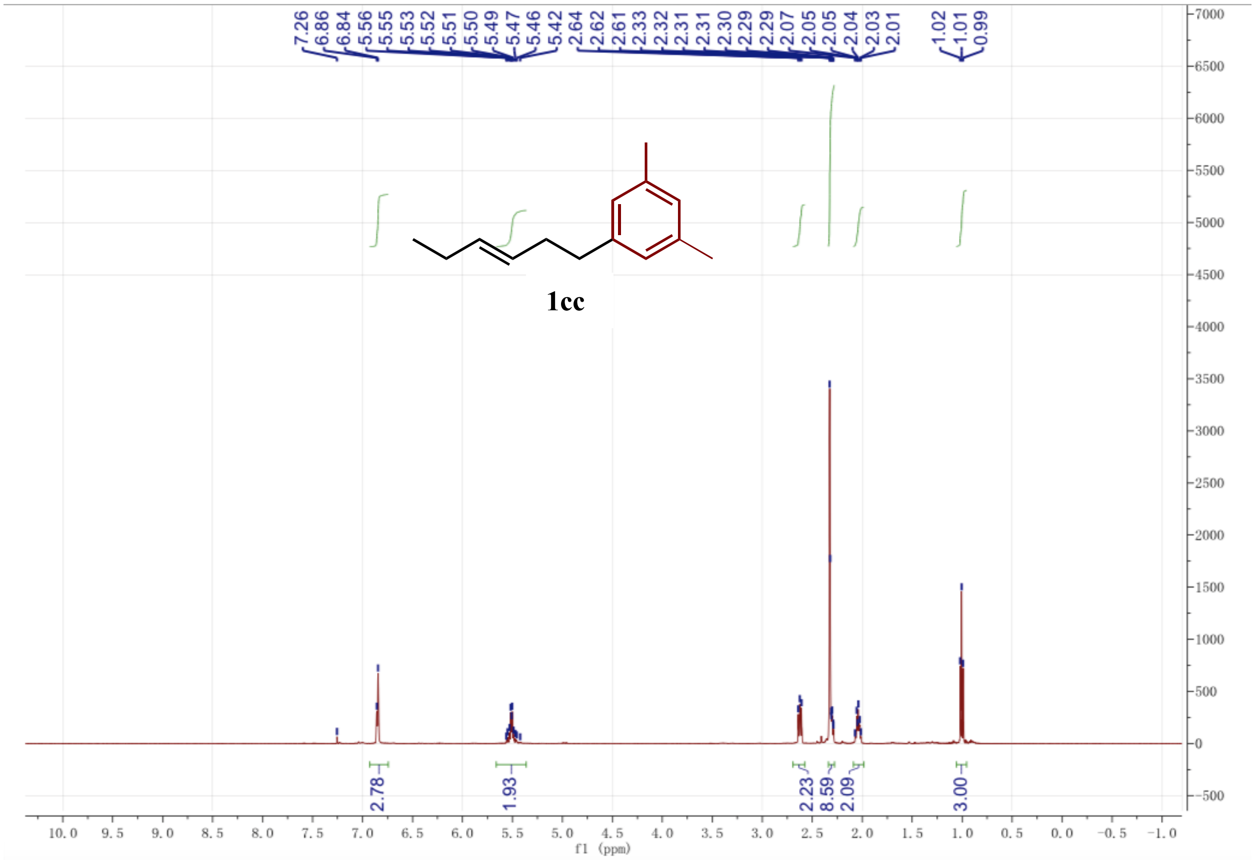
7.26
6.86
6.84
5.56
5.55
5.53
5.52
5.51
5.50
5.49
5.47
5.46
5.42
2.64
2.62
2.61
2.33
2.32
2.31
2.31
2.30
2.29
2.29
2.07
2.05
2.05
2.04
2.03
2.01
1.02
1.01
0.99
1cc
2.78
1.93
2.23
8.59
2.09
3.00
f1 (ppm)

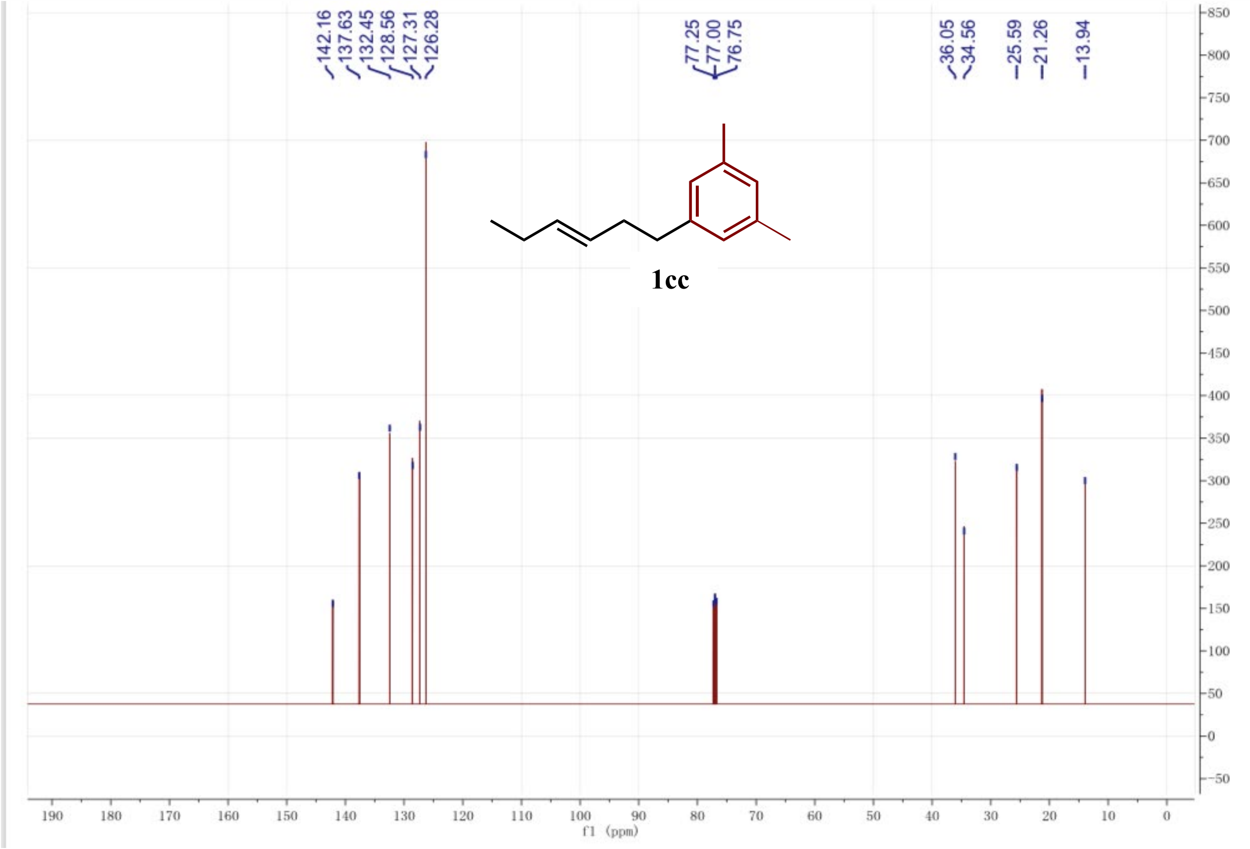
142.16
137.63
132.45
128.56
127.31
126.28
77.25
77.00
76.75
36.05
34.56
25.59
21.26
13.94
1cc
f1 (ppm)

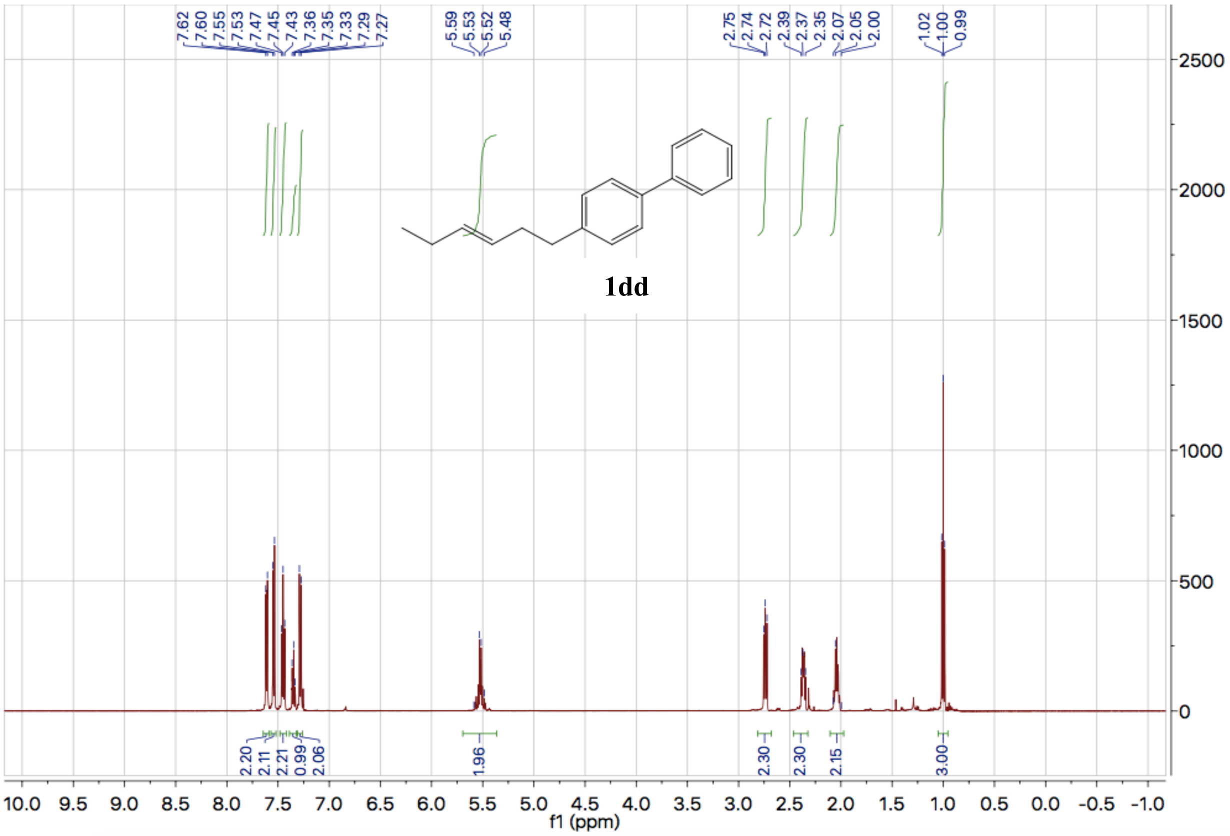
7.62
7.60
7.55
7.53
7.47
7.45
7.43
7.36
7.35
7.33
7.29
7.27
5.59
5.53
5.52
5.48
2.75
2.74
2.72
2.39
2.37
2.35
2.07
2.05
2.00
1.02
1.00
0.99
1dd
2.20
2.11
2.21
0.99
2.06
1.96
2.30
2.30
2.15
3.00
f1 (ppm)

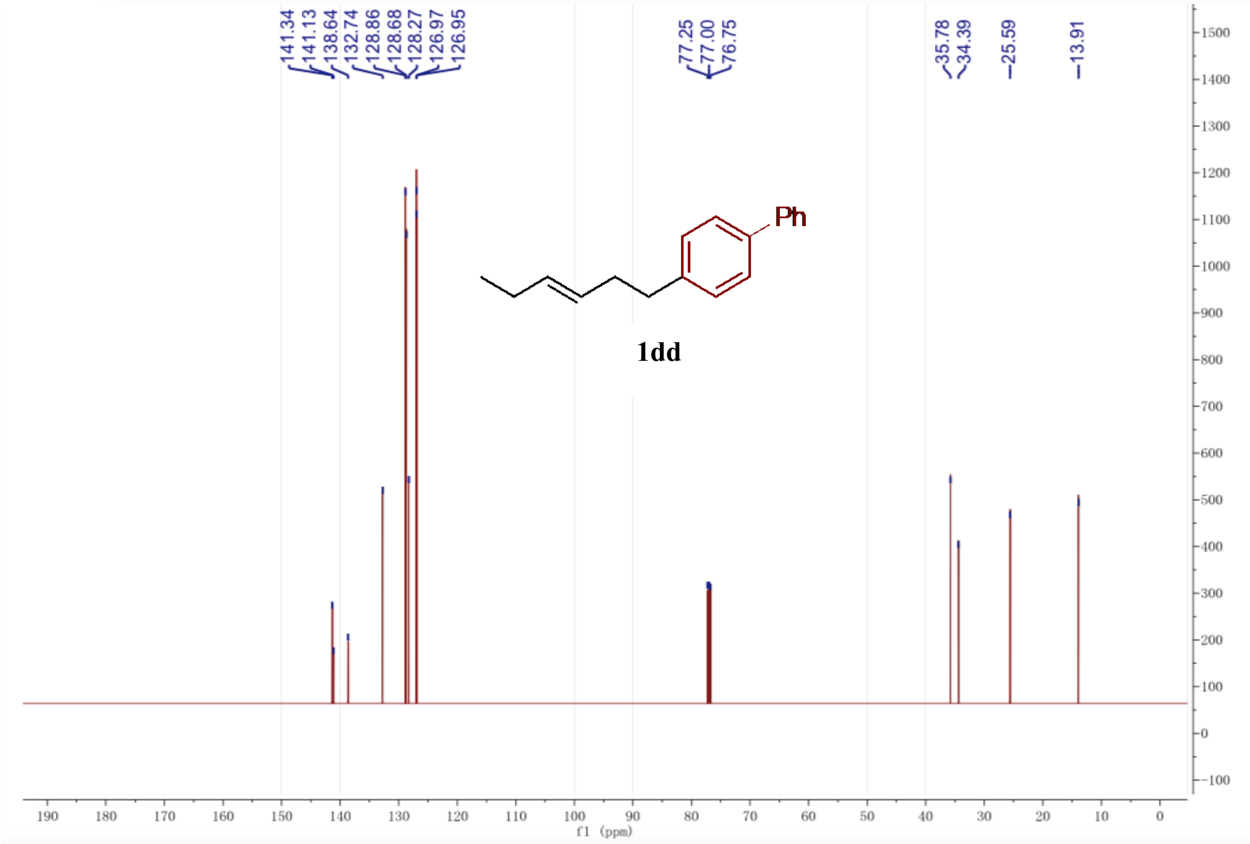
141.34
141.13
138.64
132.74
128.86
128.68
128.27
126.97
126.95
77.25
77.00
76.75
35.78
34.39
25.59
13.91
Ph
1dd
f1 (ppm)

^{1}H and ^{13}C NMR Spectra (Products):

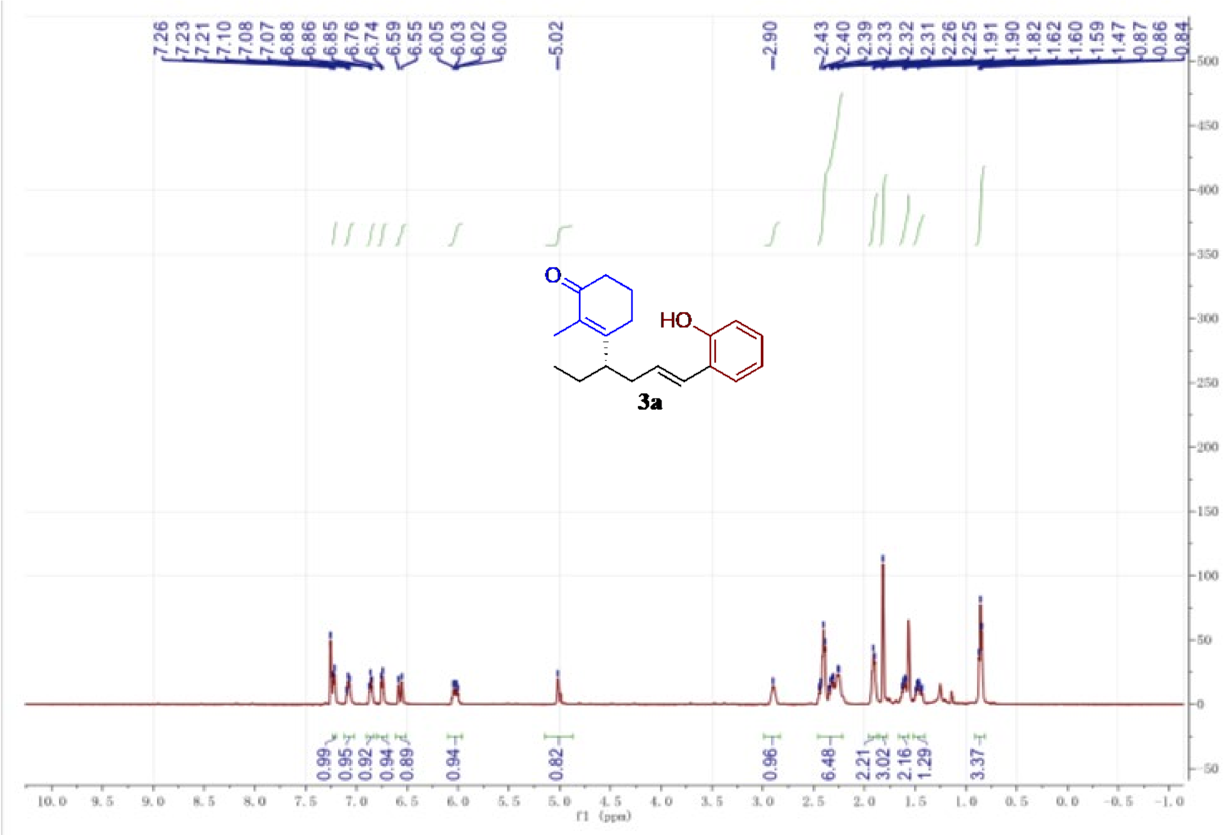

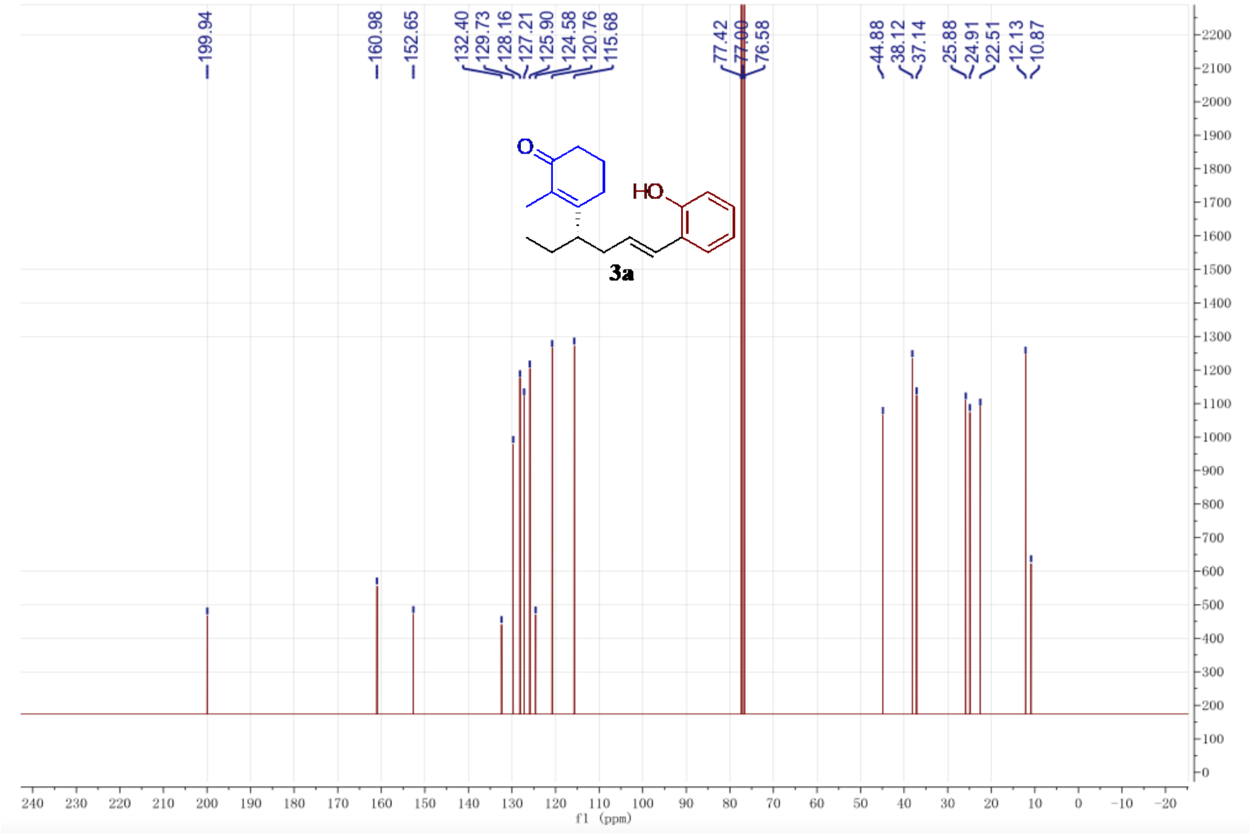

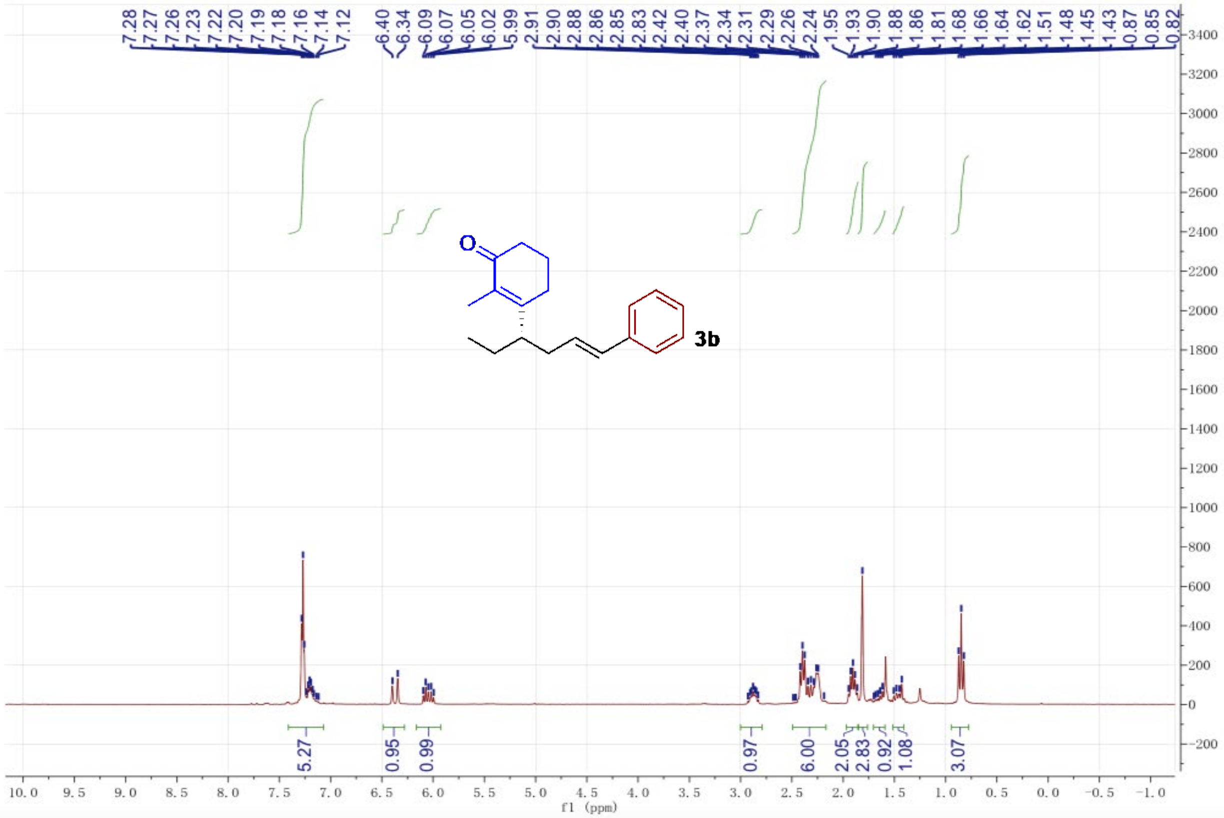
7.28
7.27
7.26
7.23
7.22
7.20
7.19
7.18
7.16
7.14
7.12
6.40
6.34
6.09
6.07
6.05
6.02
5.99
2.91
2.90
2.88
2.86
2.85
2.83
2.42
2.40
2.37
2.34
2.31
2.29
2.26
2.24
1.95
1.93
1.90
1.88
1.86
1.81
1.68
1.66
1.64
1.62
1.51
1.48
1.45
1.43
0.87
0.85
0.82
3b
5.27
0.95
0.99
0.97
6.00
2.05
2.83
0.92
1.08
3.07
f1 (ppm)

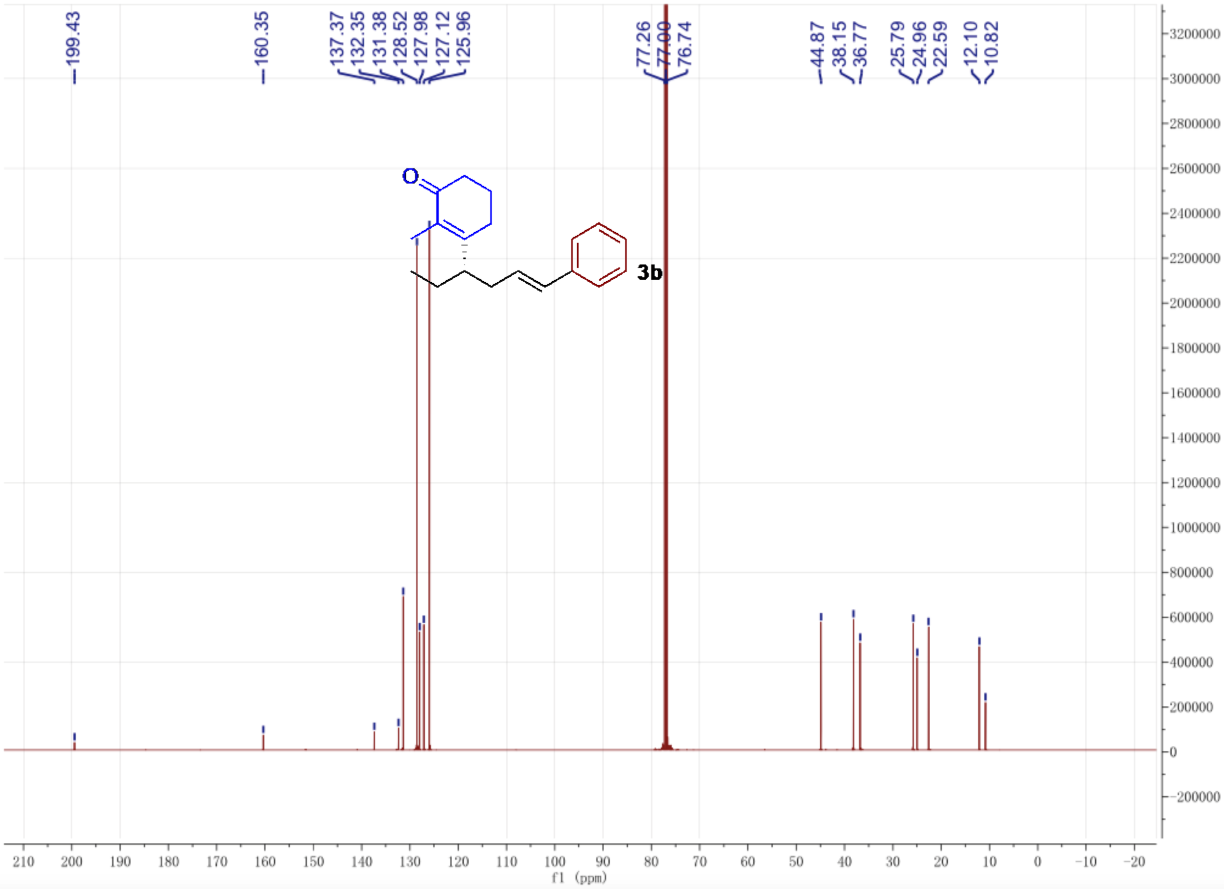
199.43
160.35
137.37
132.35
131.38
128.52
127.98
127.12
125.96
77.26
77.00
76.74
44.87
38.15
36.77
25.79
24.96
22.59
12.10
10.82
3b
f1 (ppm)

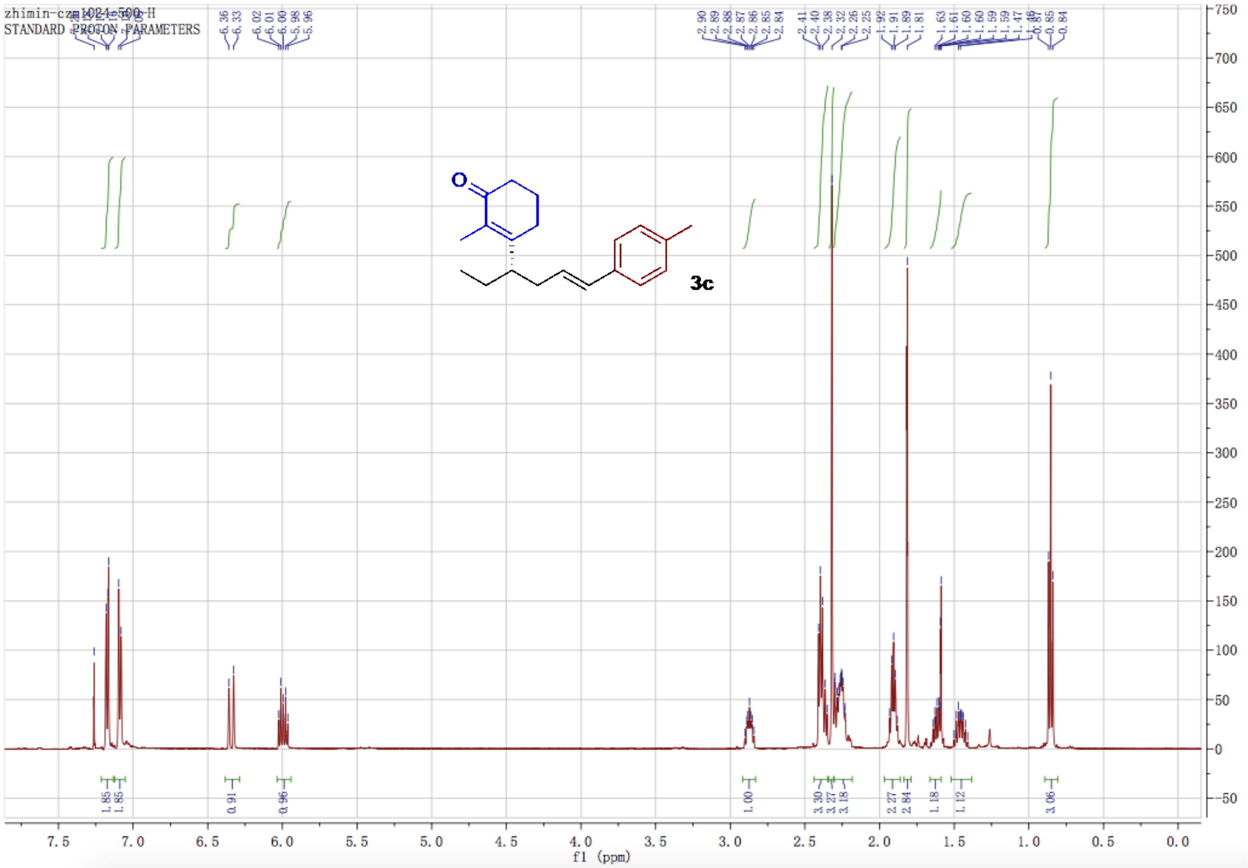
zhimin-czm1024-500-H
STANDARD PROTON PARAMETERS
3c
f1 (ppm)

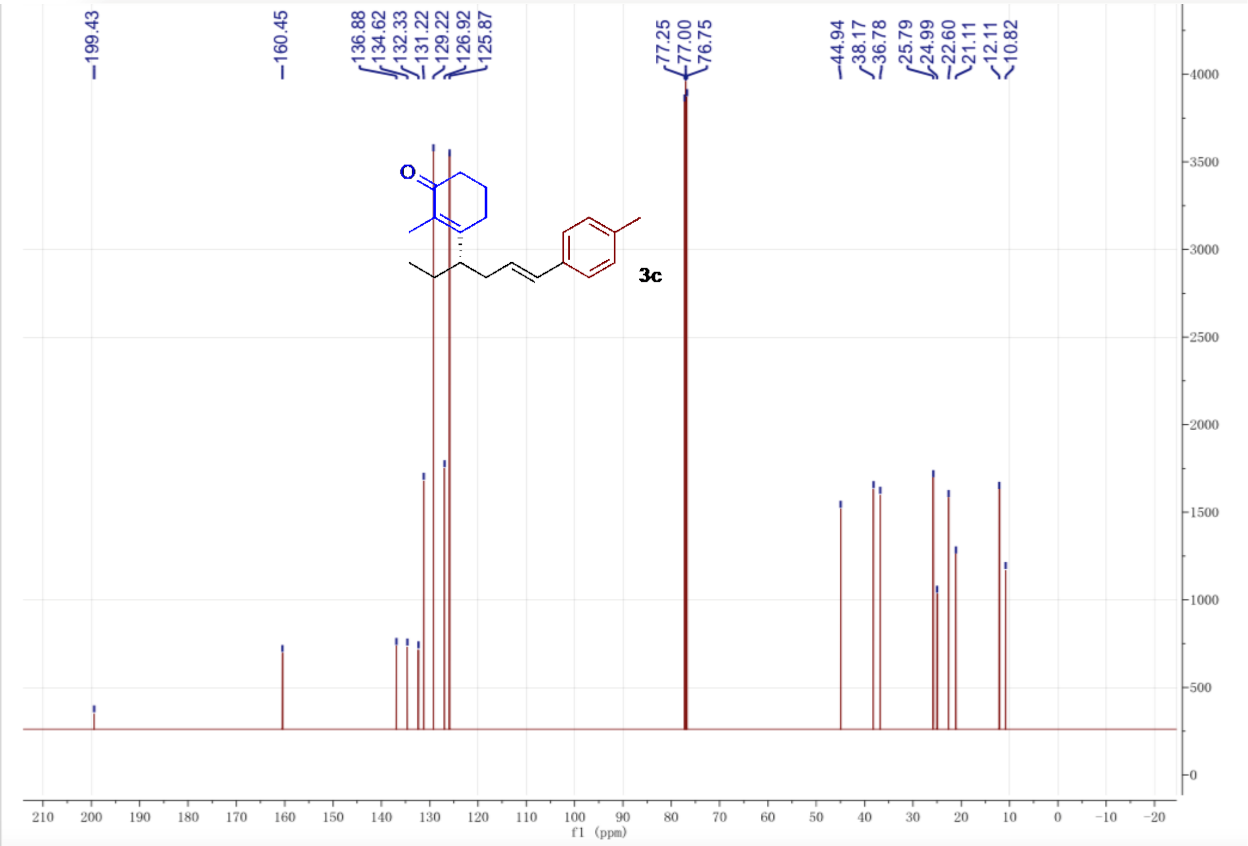
199.43
160.45
136.88
134.62
132.33
131.22
129.22
126.92
125.87
77.25
77.00
76.75
44.94
38.17
36.78
25.79
24.99
22.60
21.11
12.11
10.82
3c
f1 (ppm)

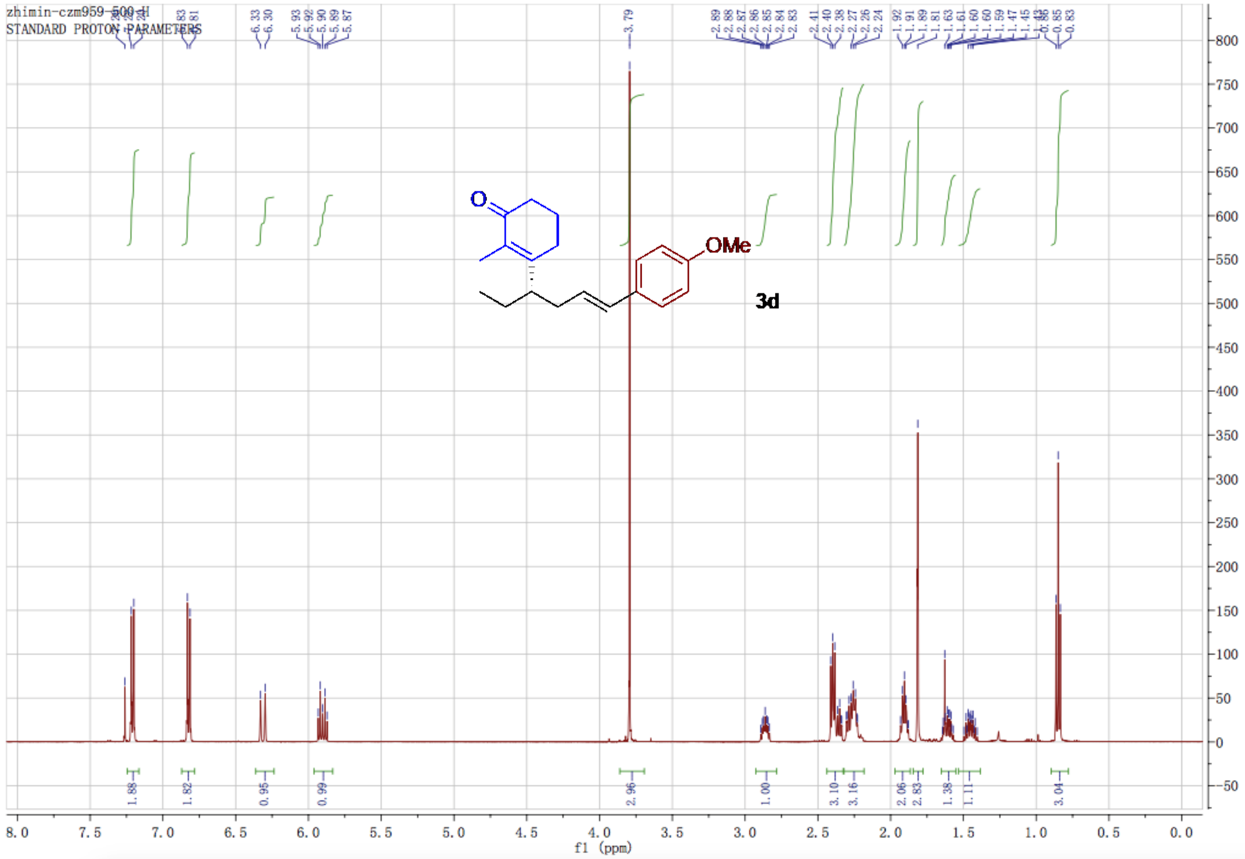

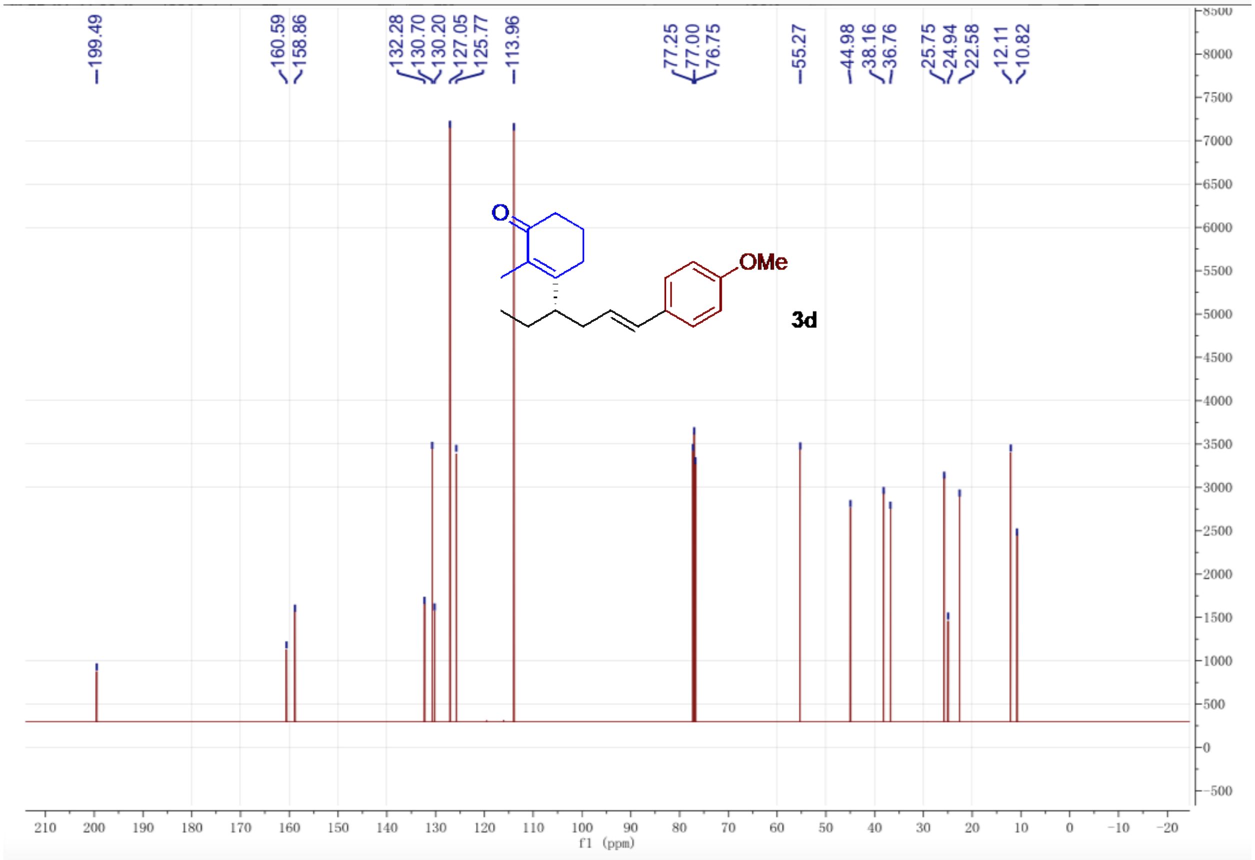

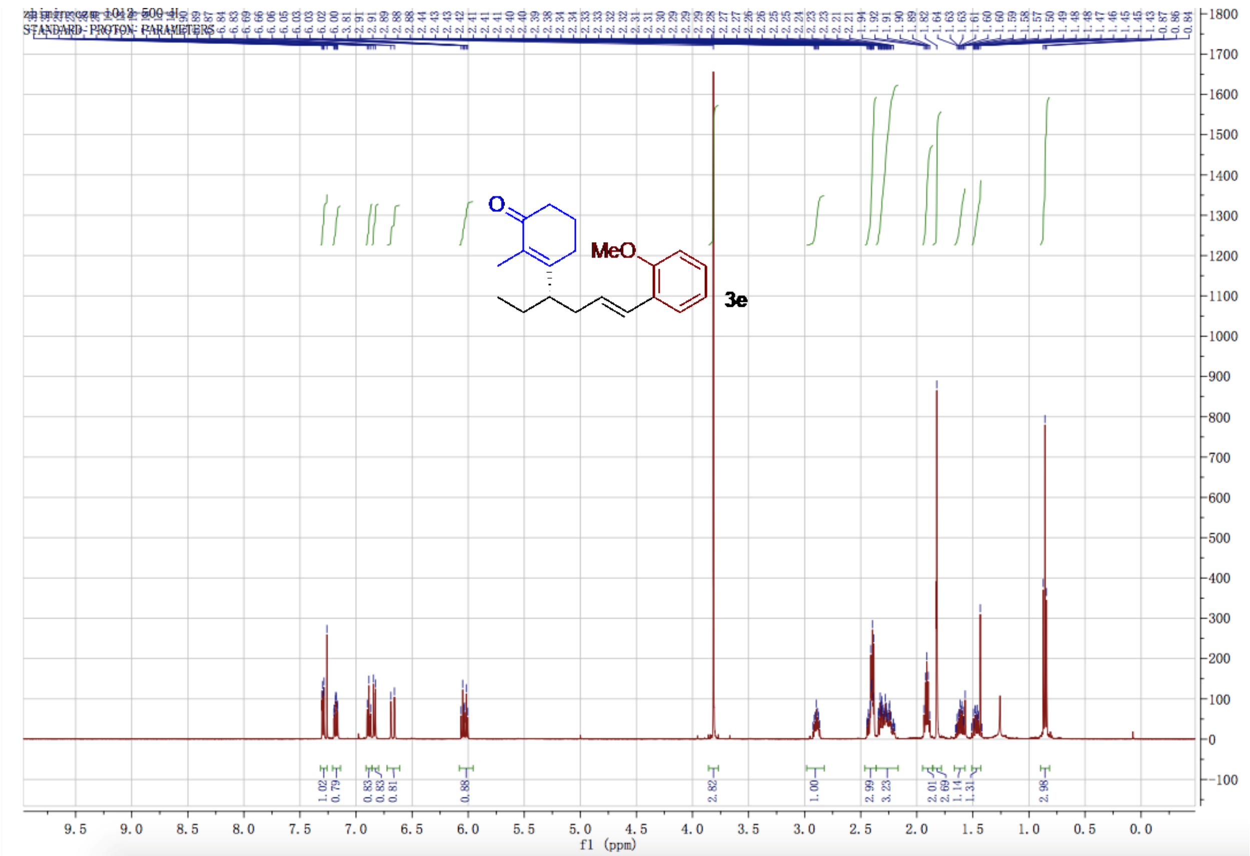

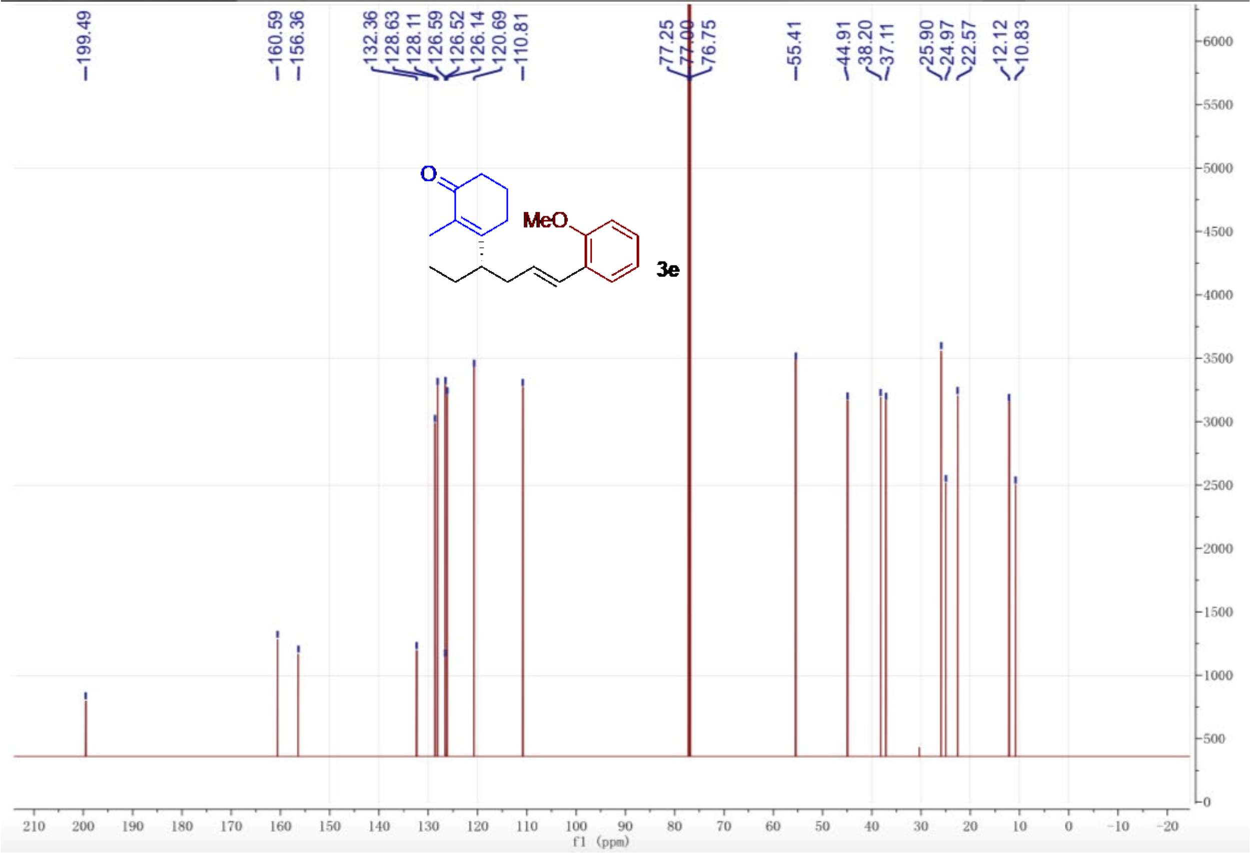

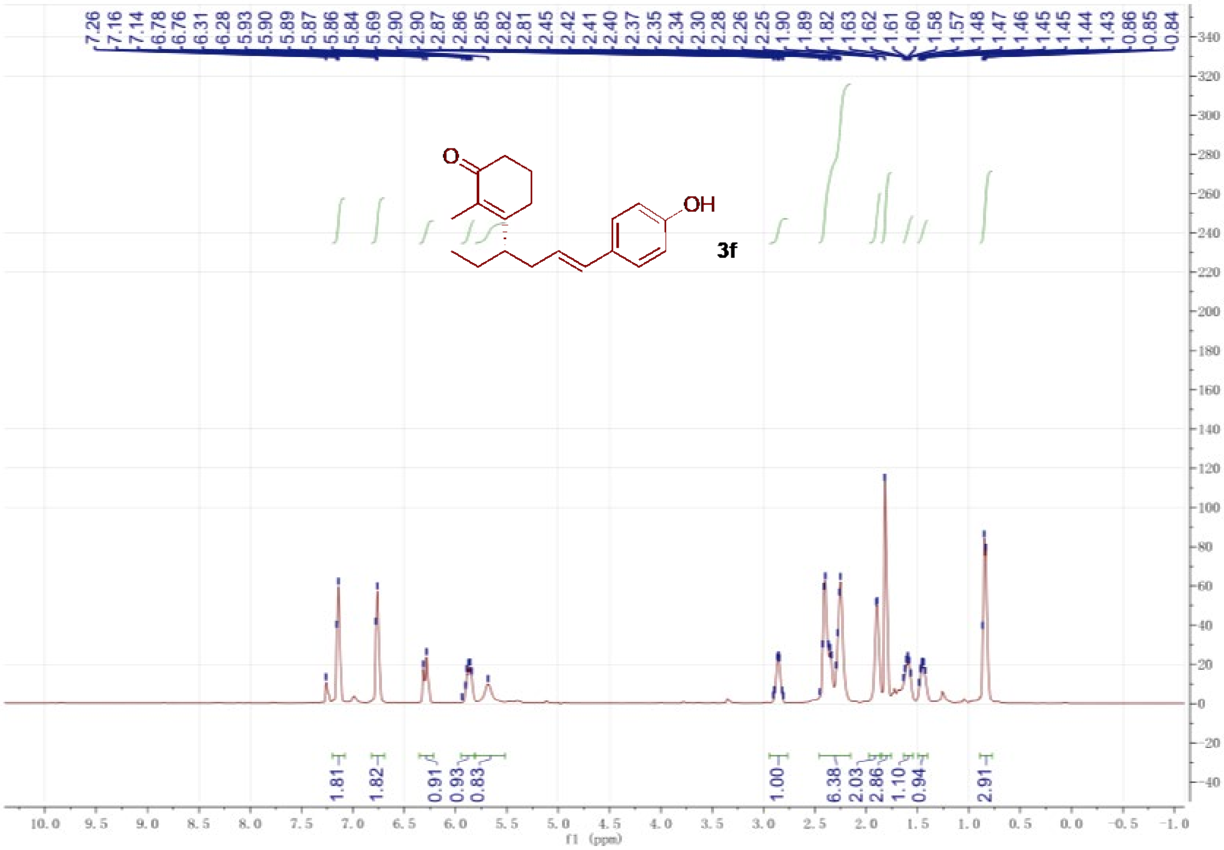
7.26
7.16
7.14
6.78
6.76
6.31
6.28
5.93
5.90
5.89
5.87
5.86
5.84
5.69
2.90
2.90
2.87
2.86
2.85
2.82
2.81
2.45
2.42
2.41
2.40
2.37
2.35
2.34
2.30
2.28
2.26
2.25
1.90
1.89
1.82
1.63
1.62
1.61
1.60
1.58
1.57
1.48
1.47
1.46
1.45
1.45
1.44
1.43
0.86
0.85
0.84
O
OH
3f
1.81
1.82
0.91
0.93
0.83
1.00
6.38
2.03
2.86
1.10
0.94
2.91
f1 (ppm)

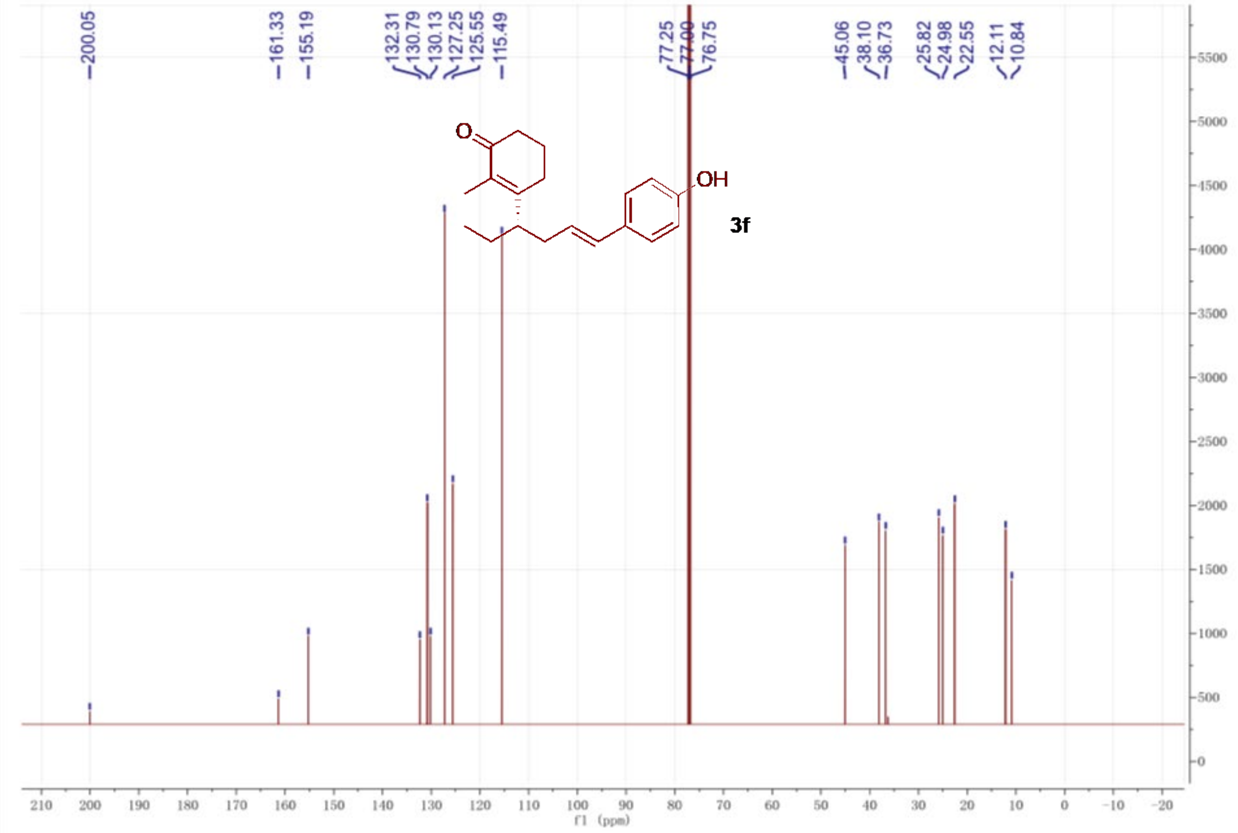
200.05
161.33
155.19
132.31
130.79
130.13
127.25
125.55
115.49
77.25
77.00
76.75
45.06
38.10
36.73
25.82
24.98
22.55
12.11
10.84
O
OH
3f
f1 (ppm)

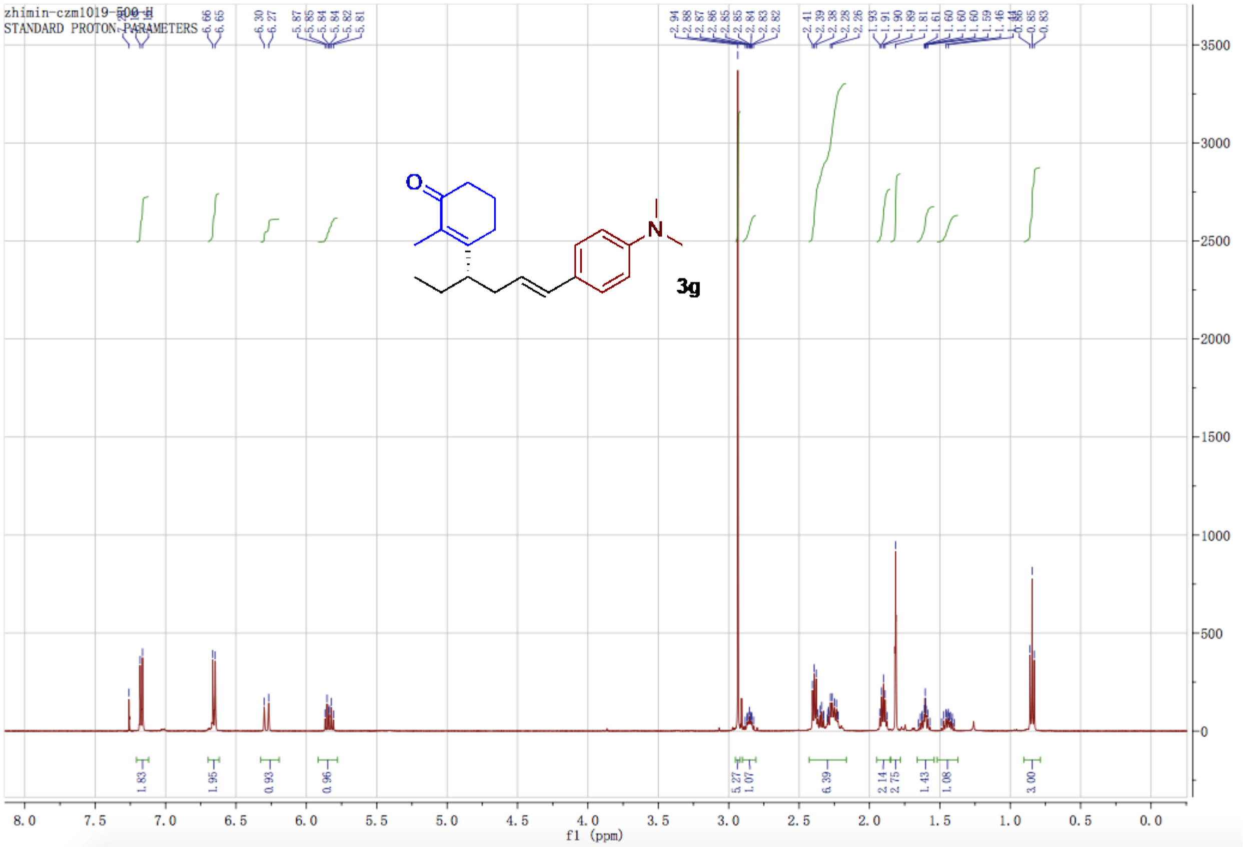
zhimin-czm1019-500-H
STANDARD PROTON PARAMETERS
3g
N
O
f1 (ppm)

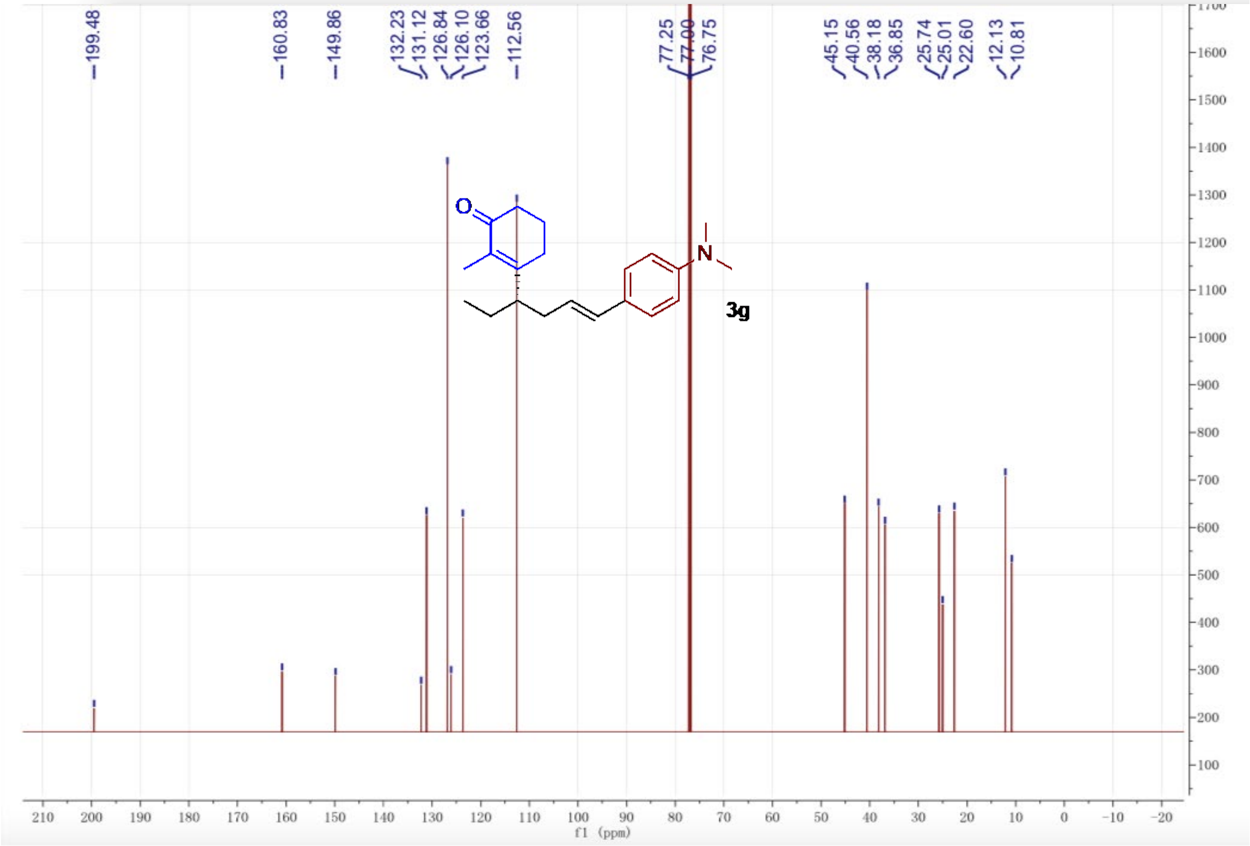
199.48
160.83
149.86
132.23
131.12
126.84
126.10
123.66
112.56
77.25
77.00
76.75
45.15
40.56
38.18
36.85
25.74
25.01
22.60
12.13
10.81
3g
N
O
f1 (ppm)

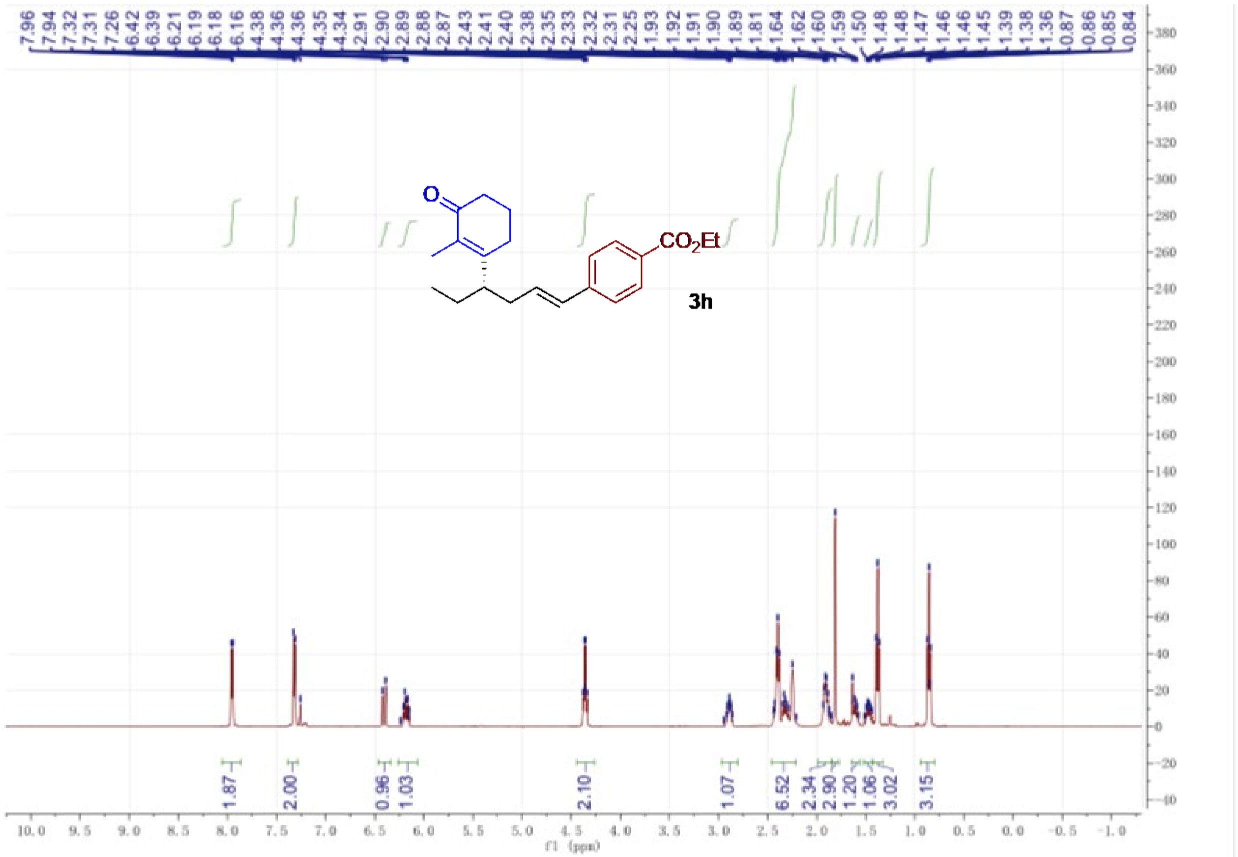

7.96
7.94
7.32
7.31
7.26
6.42
6.39
6.21
6.19
6.18
6.16
4.38
4.36
4.36
4.35
4.34
2.91
2.90
2.89
2.88
2.87
2.43
2.41
2.40
2.38
2.35
2.33
2.32
2.31
2.25
1.93
1.92
1.91
1.90
1.89
1.81
1.64
1.62
1.60
1.59
1.50
1.48
1.48
1.47
1.46
1.46
1.45
1.39
1.38
1.36
0.87
0.86
0.85
0.84
O
CO2Et
3h
1.87
2.00
0.96
1.03
2.10
1.07
6.52
2.34
2.90
1.20
1.06
3.02
3.15
f1 (ppm)

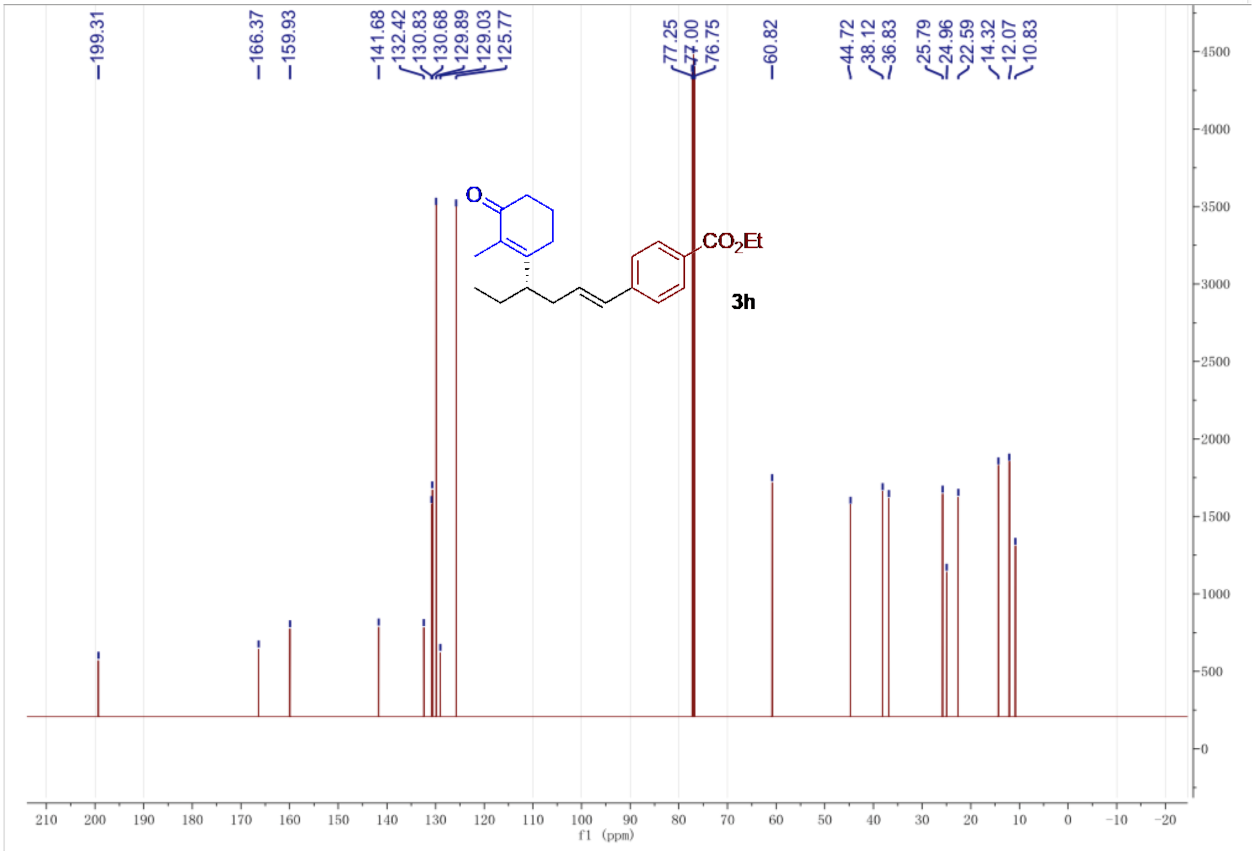

199.31
166.37
159.93
141.68
132.42
130.83
130.68
129.89
129.03
125.77
77.25
77.00
76.75
60.82
44.72
38.12
36.83
25.79
24.96
22.59
14.32
12.07
10.83
O
CO2Et
3h
f1 (ppm)

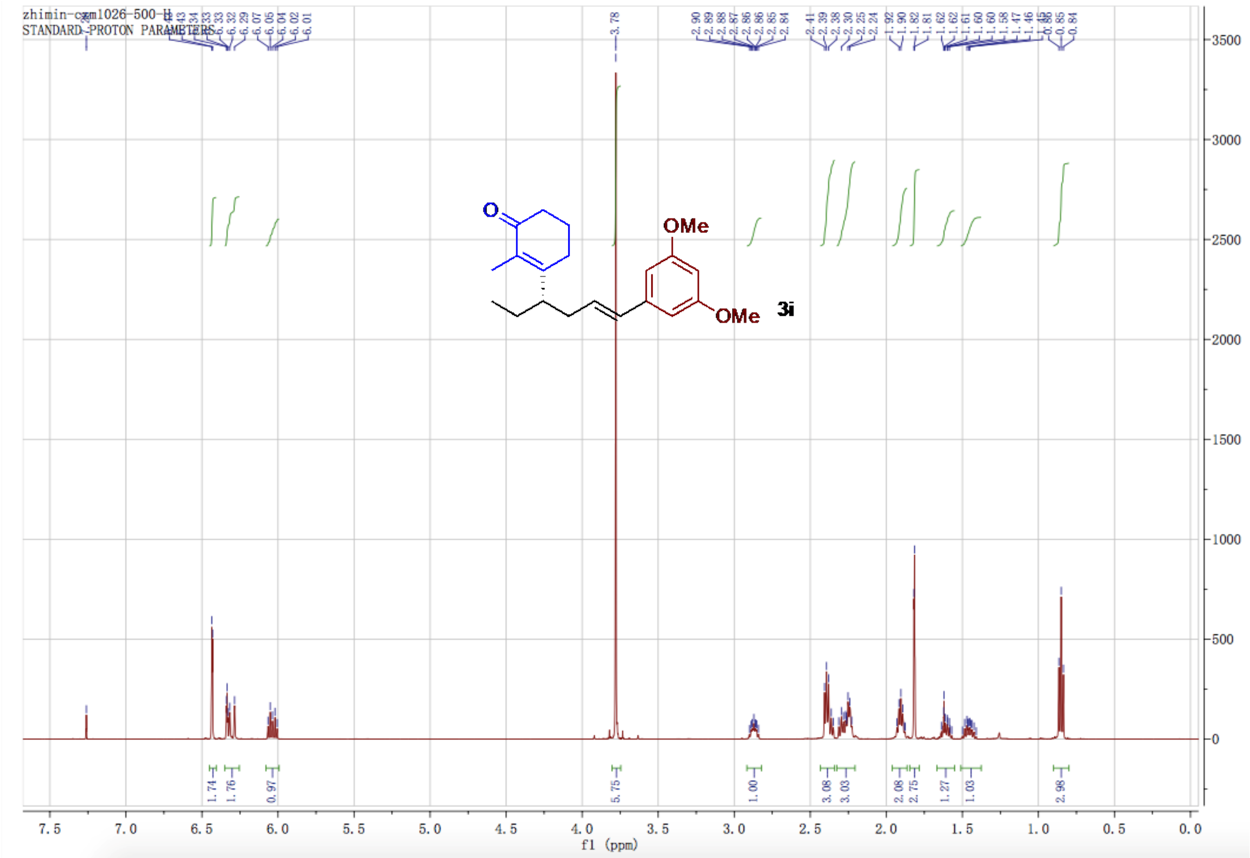

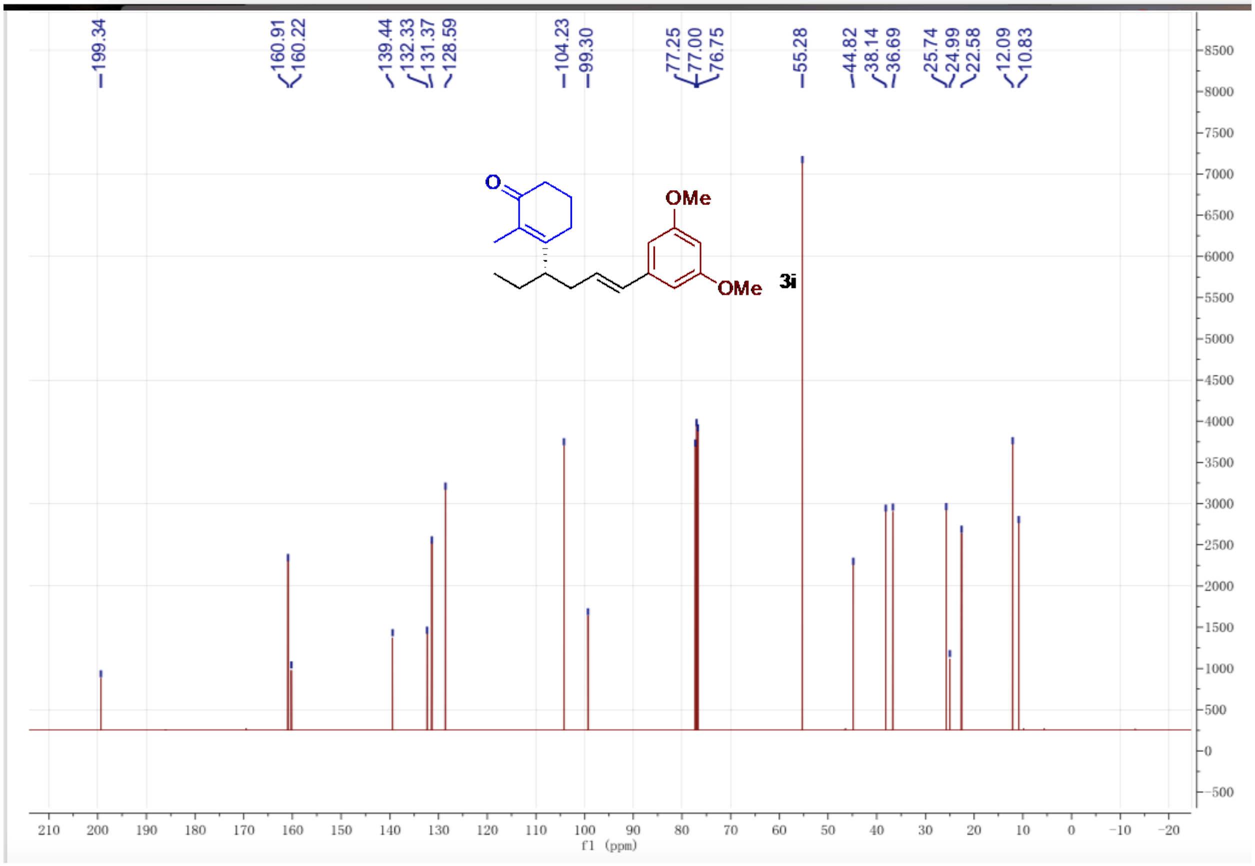

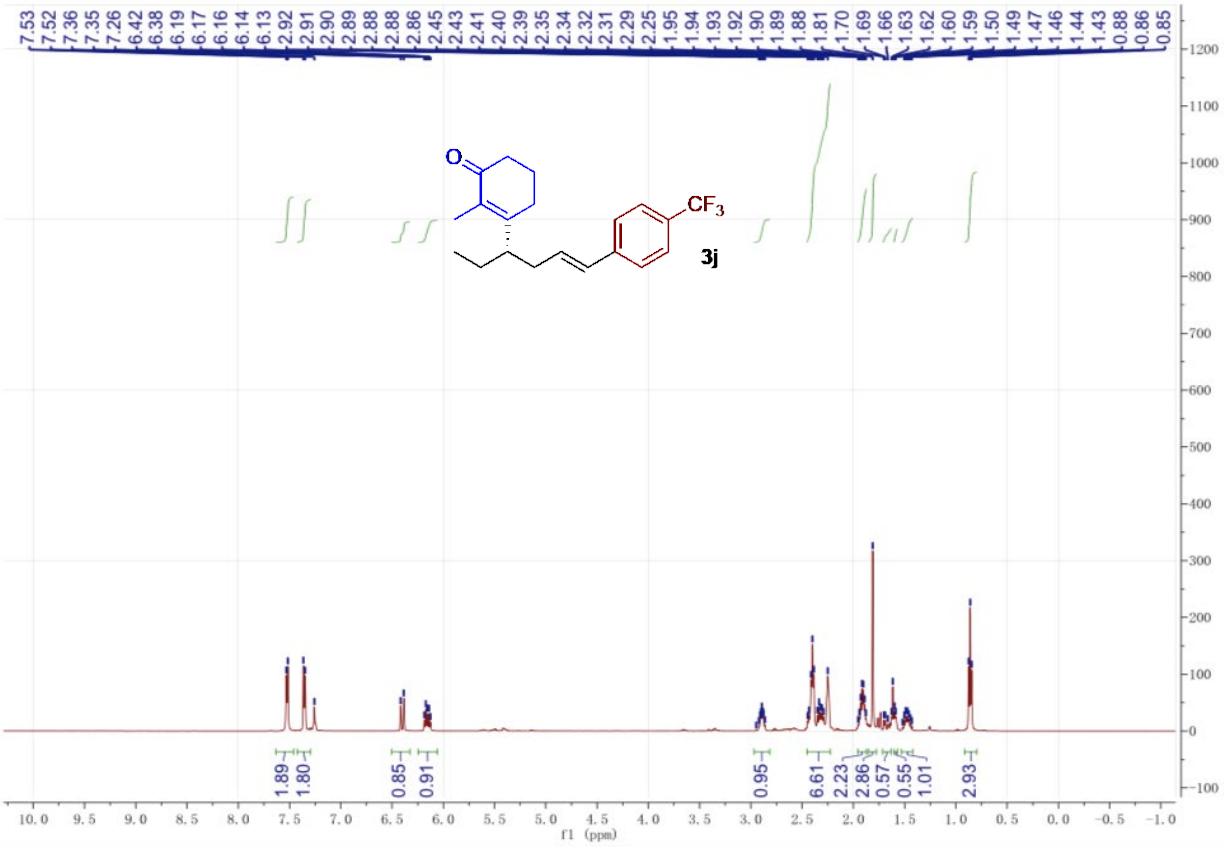
CF3
3j

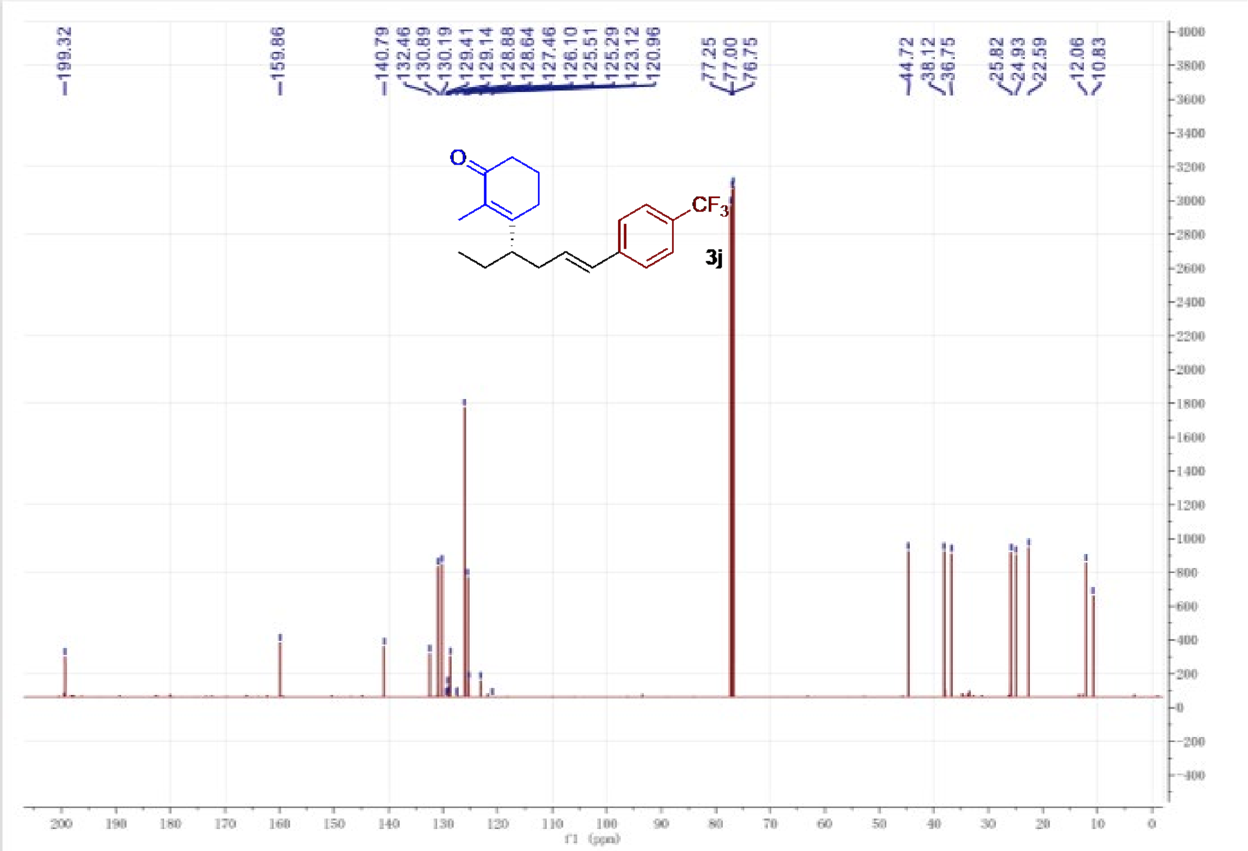
CF3
3j

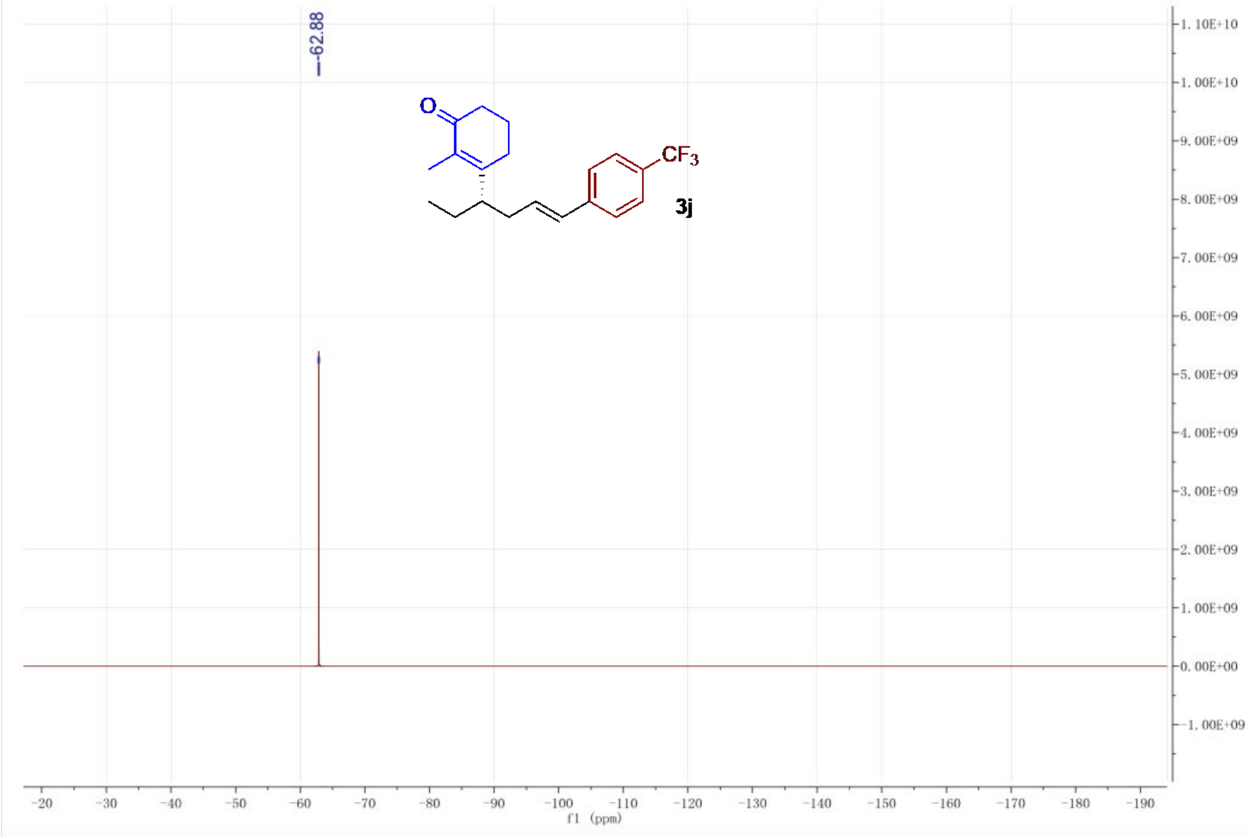

—62.88
O
CF3
3j
1.10E+10
1.00E+10
9.00E+09
8.00E+09
7.00E+09
6.00E+09
5.00E+09
4.00E+09
3.00E+09
2.00E+09
1.00E+09
0.00E+00
-1.00E+09
-20 -30 -40 -50 -60 -70 -80 -90 -100 -110 -120 -130 -140 -150 -160 -170 -180 -190
f1 (ppm)

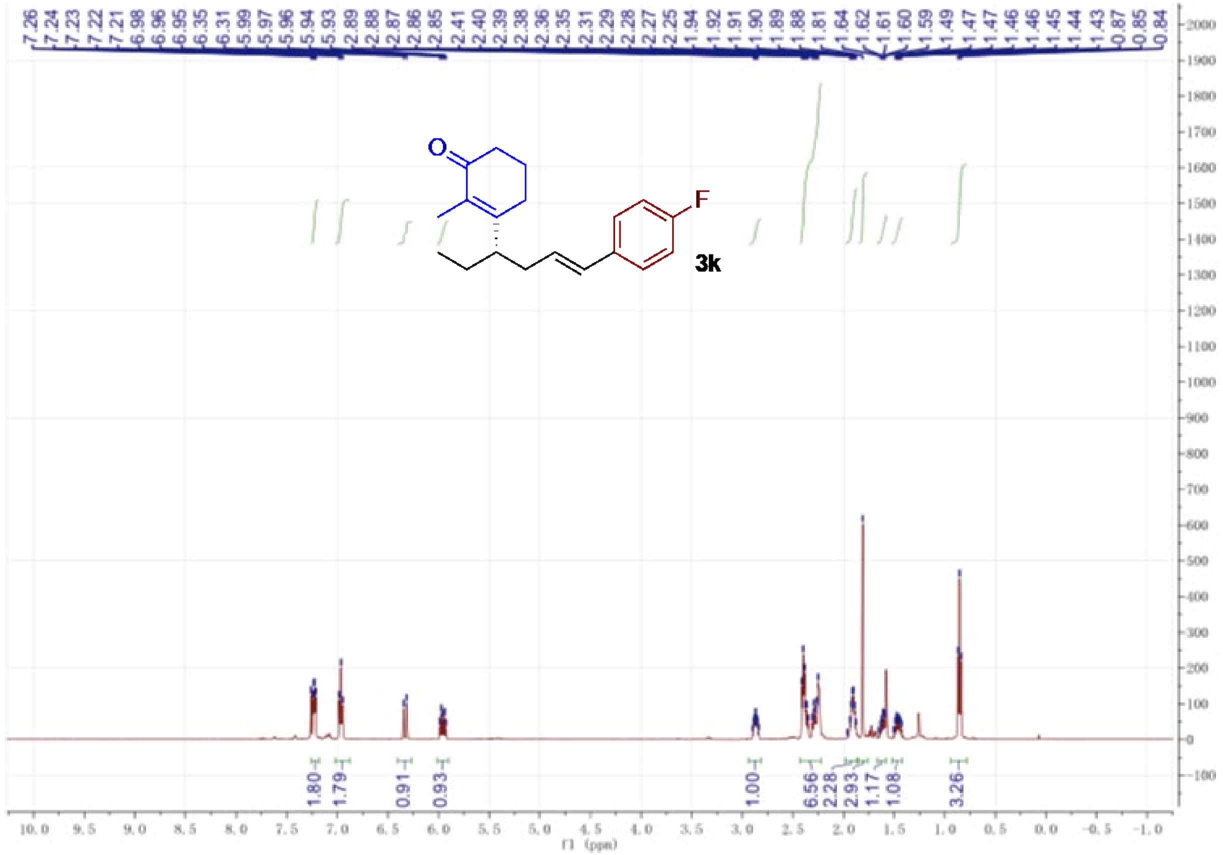

7.26 7.24 7.23 7.22 7.21 6.98 6.96 6.95 6.35 6.31 5.99 5.97 5.96 5.94 5.93 2.89 2.88 2.87 2.86 2.85 2.41 2.40 2.39 2.38 2.36 2.35 2.31 2.29 2.28 2.27 2.25 1.94 1.92 1.91 1.90 1.89 1.88 1.81 1.64 1.62 1.61 1.60 1.59 1.49 1.47 1.47 1.46 1.46 1.45 1.44 1.43 0.87 0.85 0.84
O
F
3k
1.80 1.79 0.91 0.93 1.00 6.56 2.28 2.93 1.17 1.08 3.26
10.0 9.5 9.0 8.5 8.0 7.5 7.0 6.5 6.0 5.5 5.0 4.5 4.0 3.5 3.0 2.5 2.0 1.5 1.0 0.5 0.0 -0.5 -1.0
f1 (ppm)

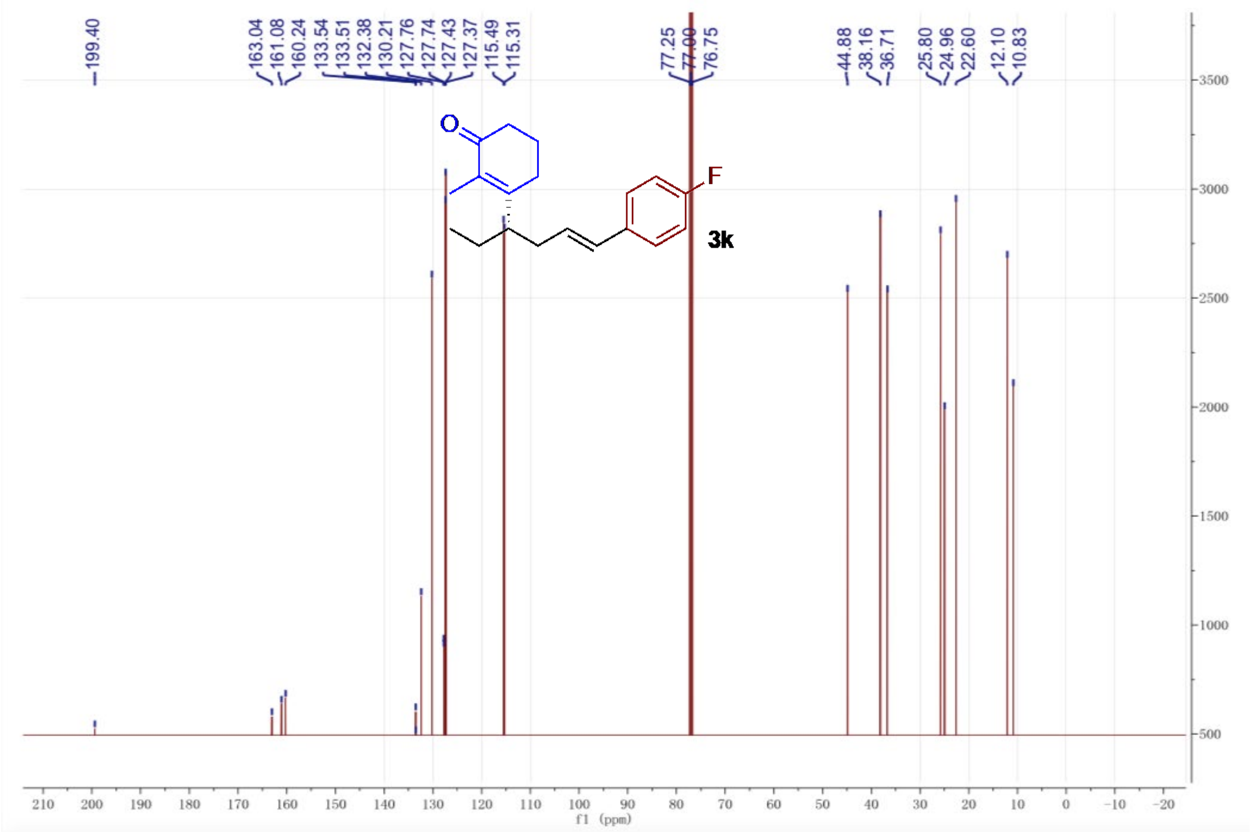
199.40
163.04
161.08
160.24
133.54
133.51
132.38
130.21
127.76
127.74
127.43
127.37
115.49
115.31
77.25
77.00
76.75
44.88
38.16
36.71
25.80
24.96
22.60
12.10
10.83
O
F
3k
f1 (ppm)

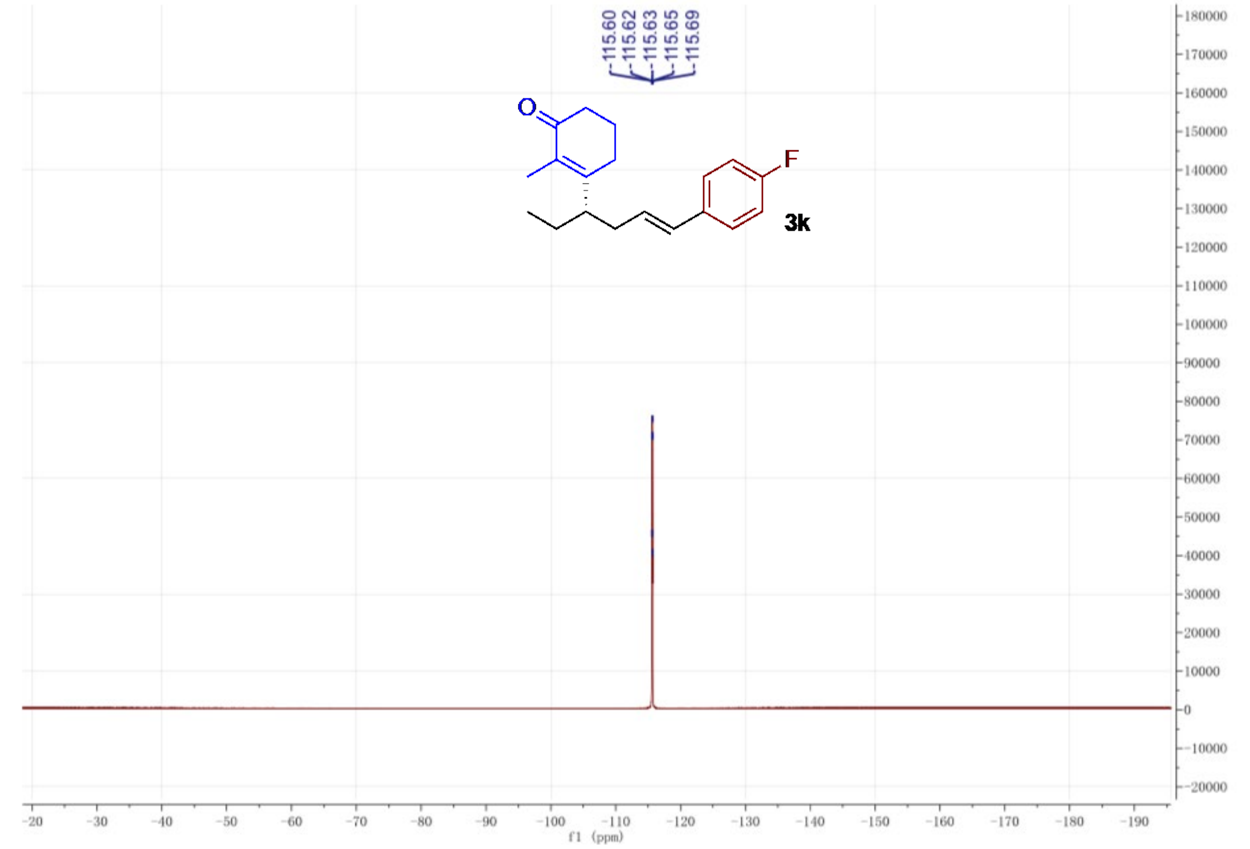
115.60
115.62
115.63
115.65
115.69
O
F
3k
f1 (ppm)

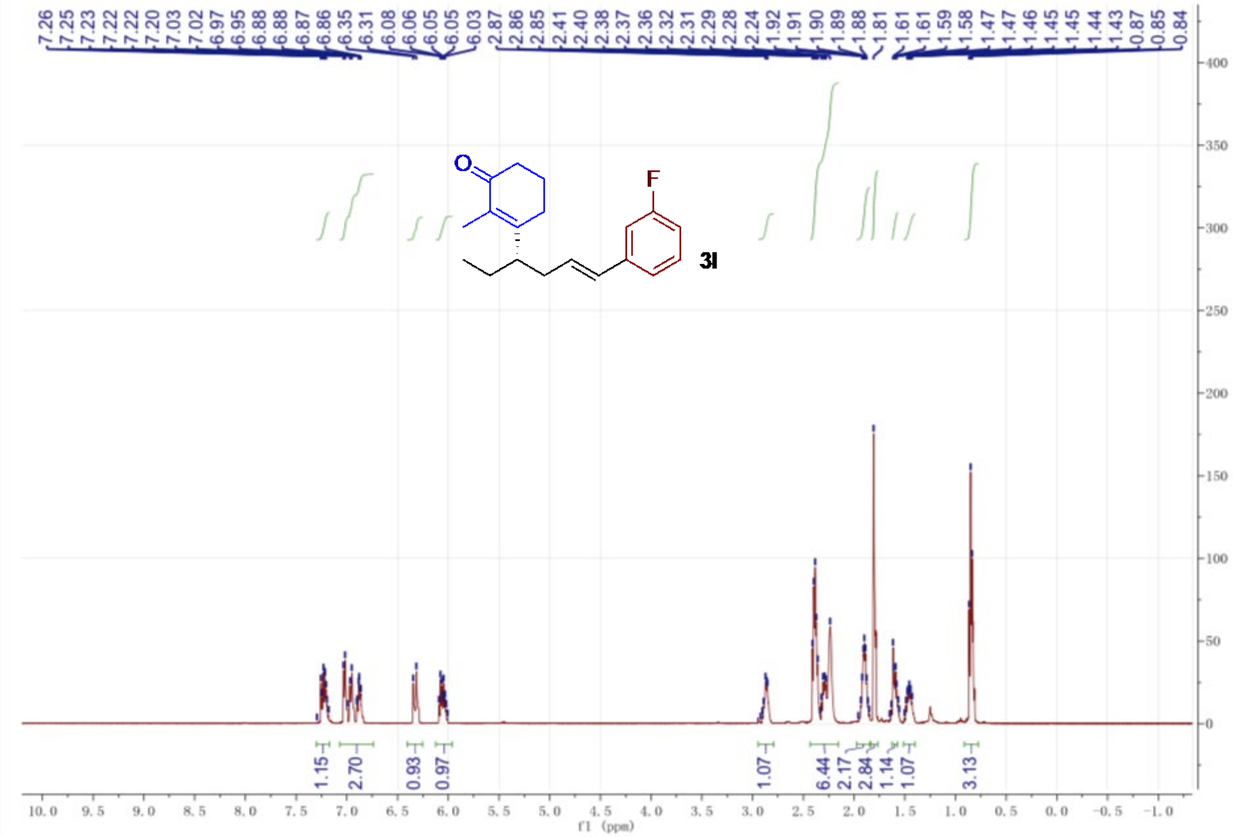
7.26
7.25
7.23
7.22
7.22
7.20
7.03
7.02
6.97
6.95
6.88
6.88
6.87
6.86
6.35
6.31
6.08
6.06
6.05
6.05
6.03
2.87
2.86
2.85
2.41
2.40
2.38
2.37
2.36
2.32
2.31
2.29
2.28
2.24
1.92
1.91
1.90
1.89
1.88
1.81
1.61
1.61
1.59
1.58
1.47
1.47
1.46
1.45
1.45
1.44
1.43
0.87
0.85
0.84
O
F
3l
1.15
2.70
0.93
0.97
1.07
6.44
2.17
2.84
1.14
1.07
3.13
f1 (ppm)

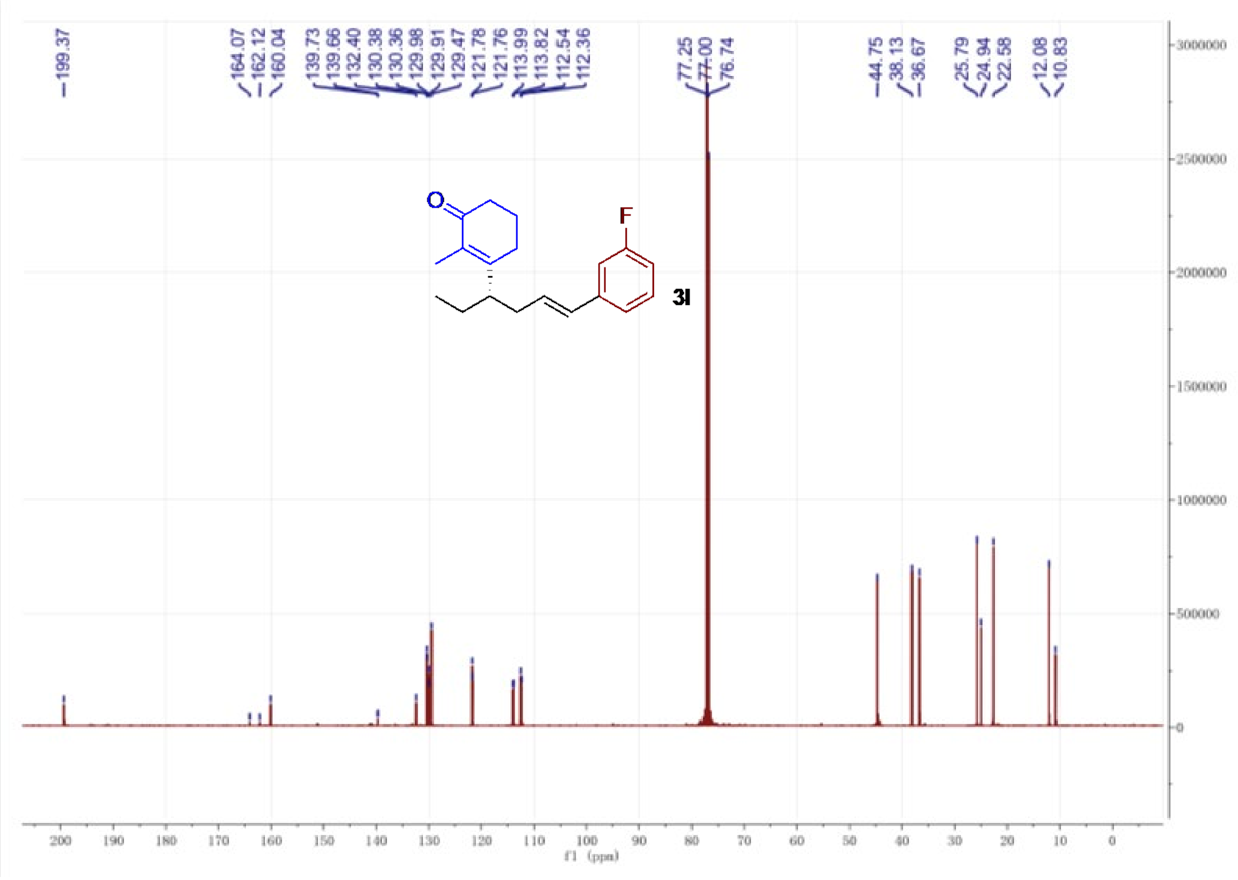
199.37
164.07
162.12
160.04
139.73
139.66
132.40
130.38
130.36
129.98
129.91
129.47
121.78
121.76
113.99
113.82
112.54
112.36
77.25
77.00
76.74
44.75
38.13
36.67
25.79
24.94
22.58
12.08
10.83
O
F
3l
f1 (ppm)

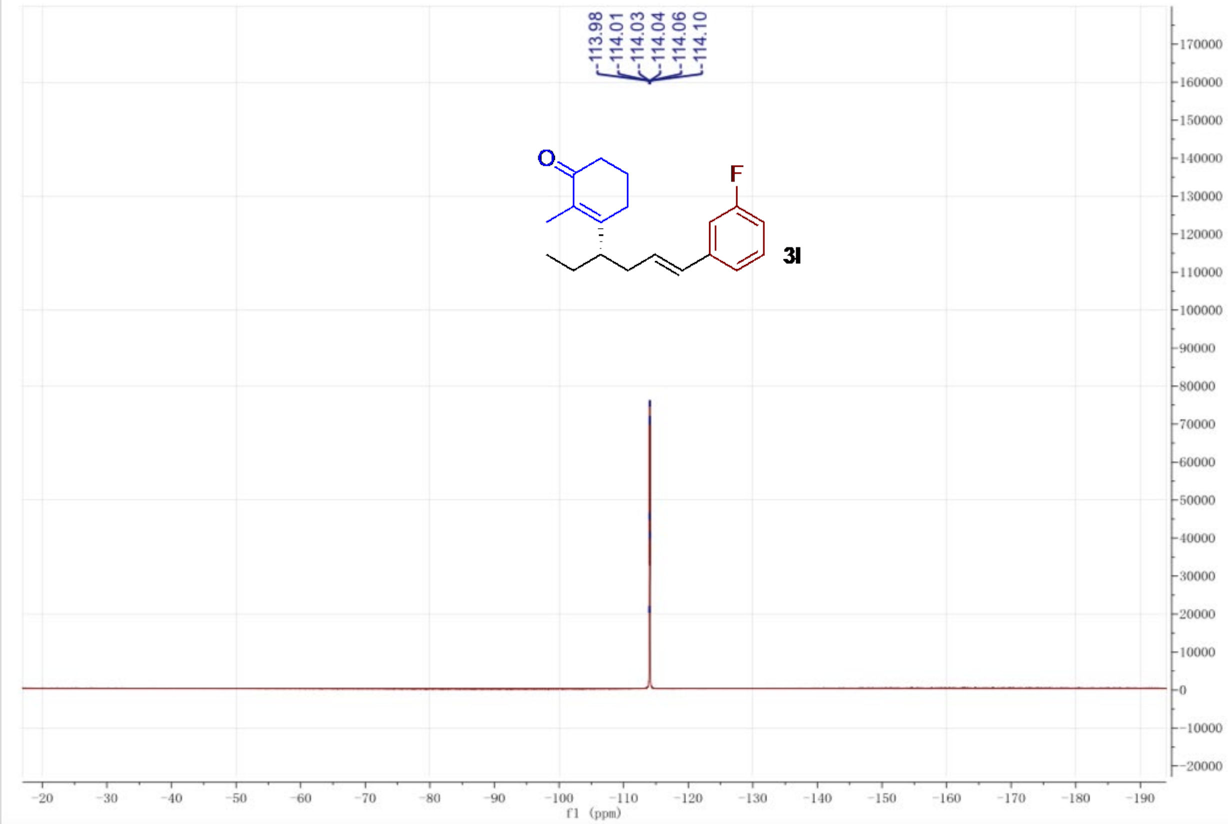
-113.98
-114.01
-114.03
-114.04
-114.06
-114.10
O
F
3l
f1 (ppm)

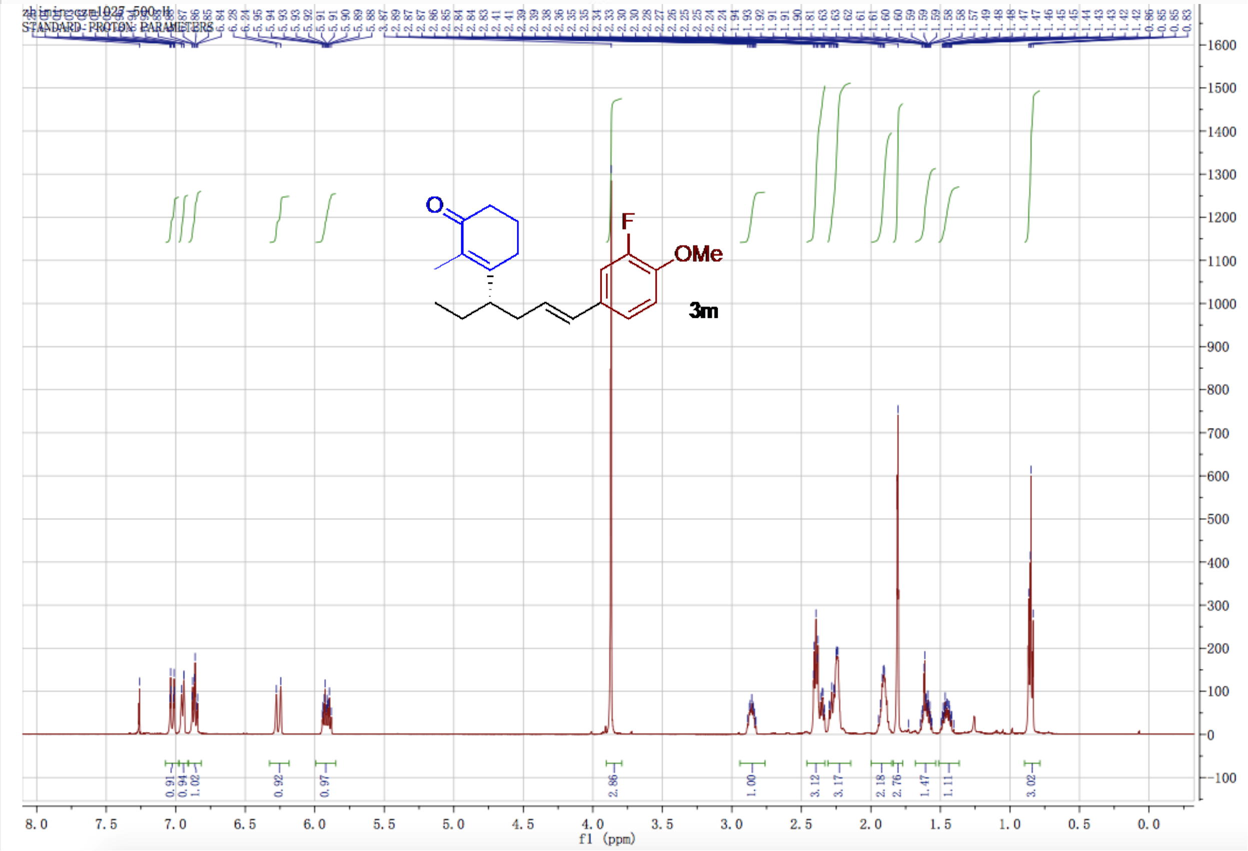
STANDARD PROTON PARAMETERS
O
F
OMe
3m
f1 (ppm)

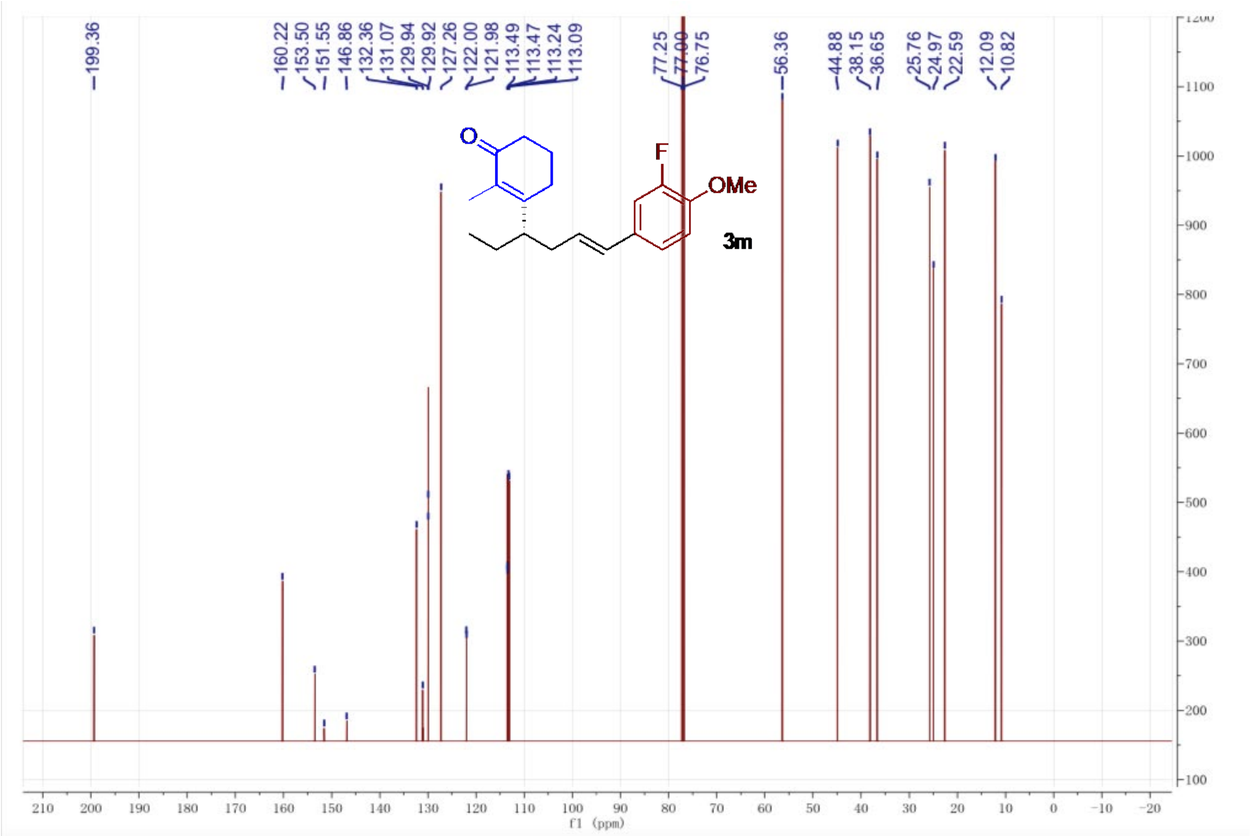
199.36
160.22
153.50
151.55
146.86
132.36
131.07
129.94
129.92
127.26
122.00
121.98
113.49
113.47
113.24
113.09
77.25
77.00
76.75
56.36
44.88
38.15
36.65
25.76
24.97
22.59
12.09
10.82
O
F
OMe
3m
f1 (ppm)

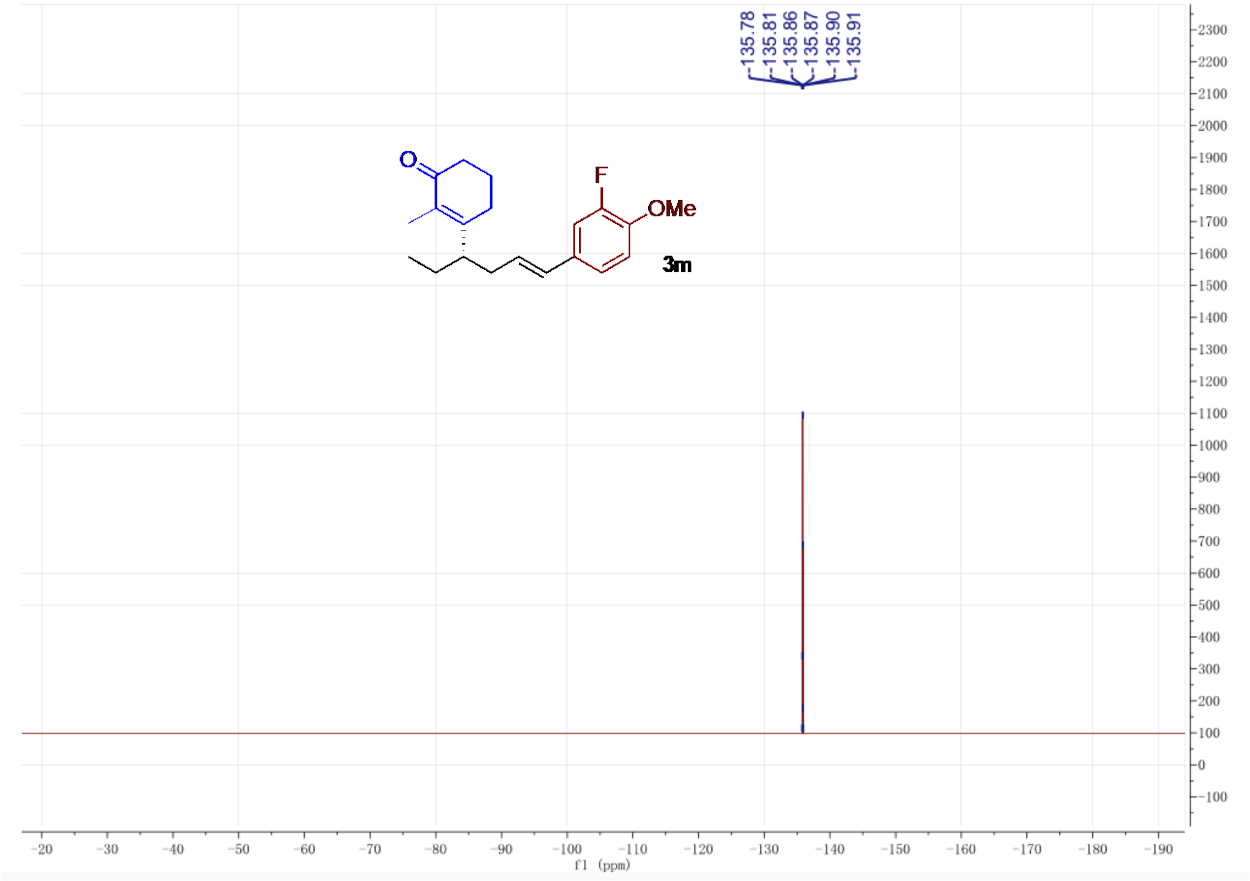
135.78
135.81
135.86
135.87
135.90
135.91
O
F
OMe
3m
f1 (ppm)

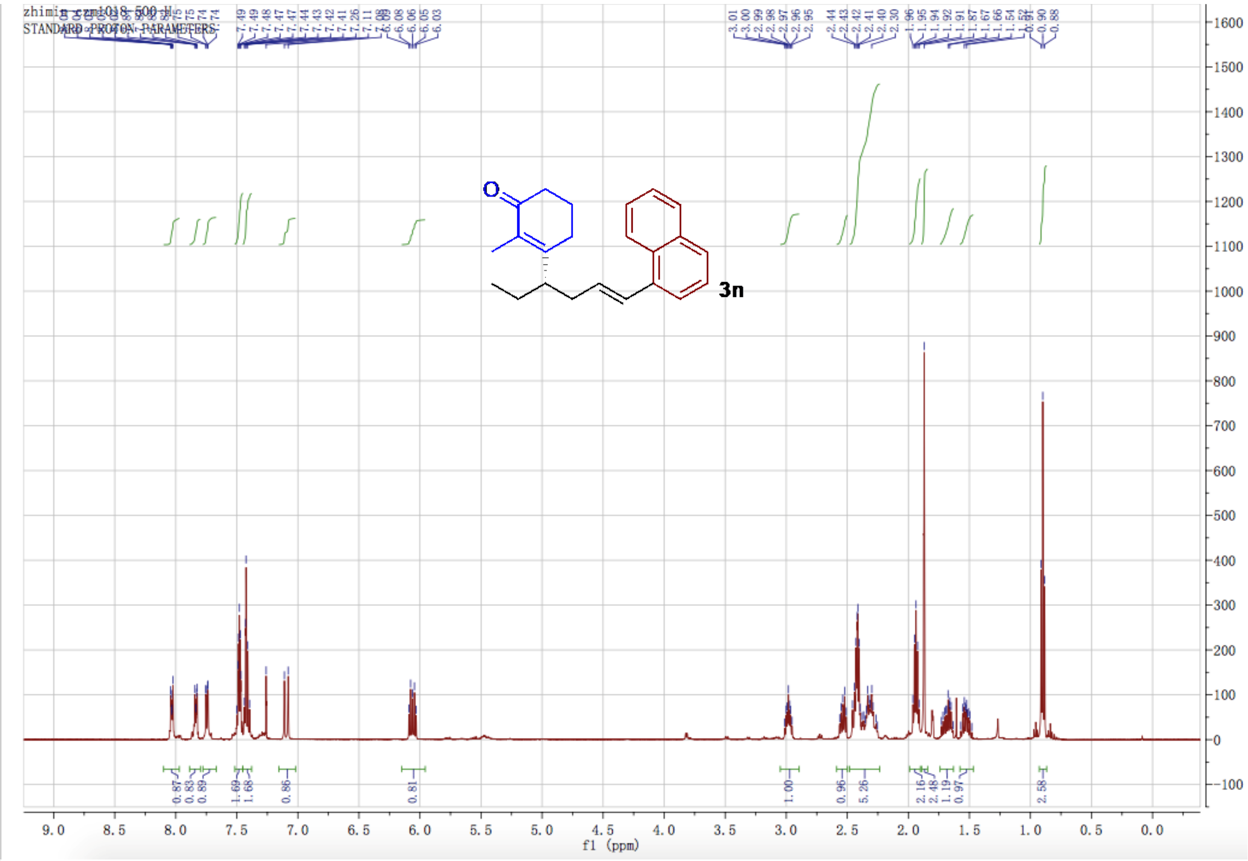
3n
0.87
0.83
0.89
1.69
1.68
0.86
0.81
1.00
0.96
5.26
2.16
2.48
1.19
0.97
2.58
f1 (ppm)

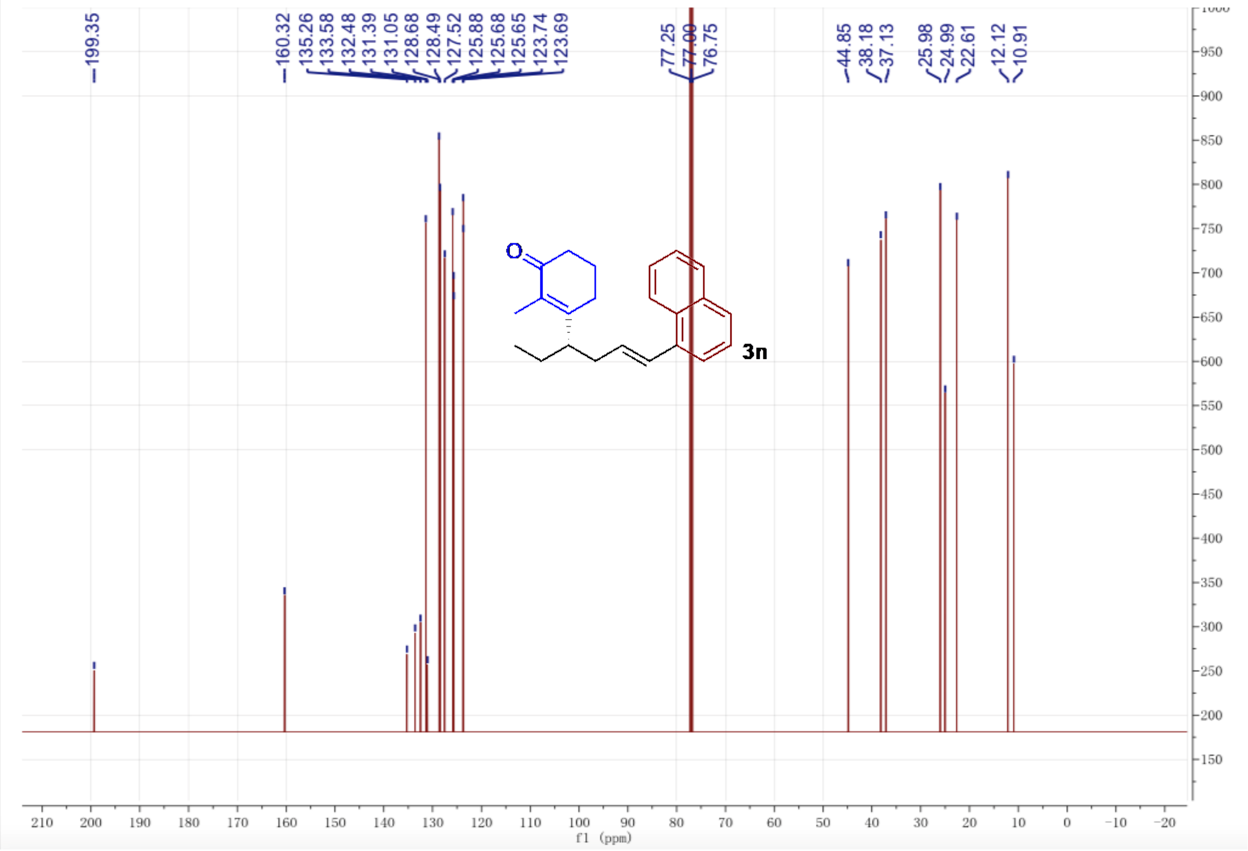
199.35
160.32
135.26
133.58
132.48
131.39
131.05
128.68
128.49
127.52
125.88
125.68
125.65
123.74
123.69
77.25
77.00
76.75
44.85
38.18
37.13
25.98
24.99
22.61
12.12
10.91
3n
f1 (ppm)

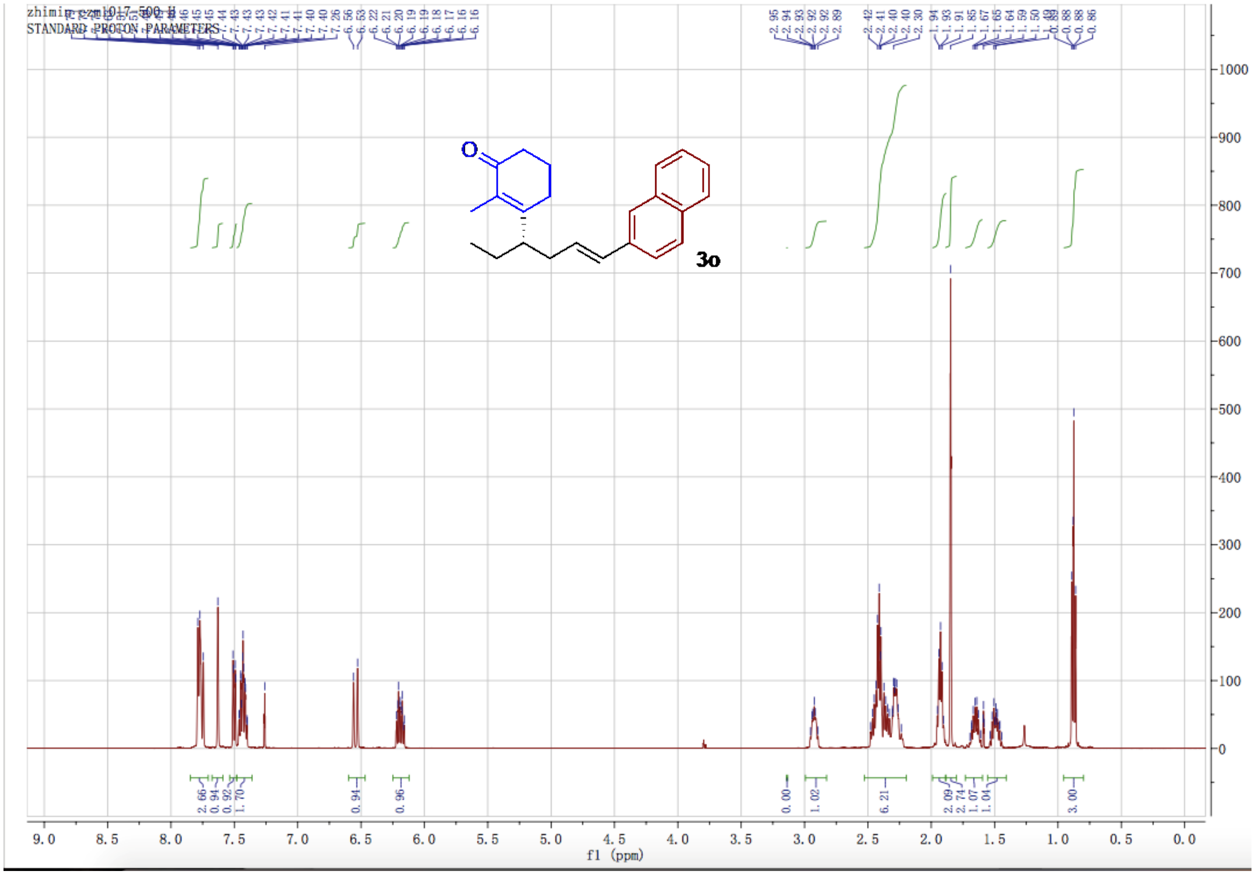

O
3o
f1 (ppm)

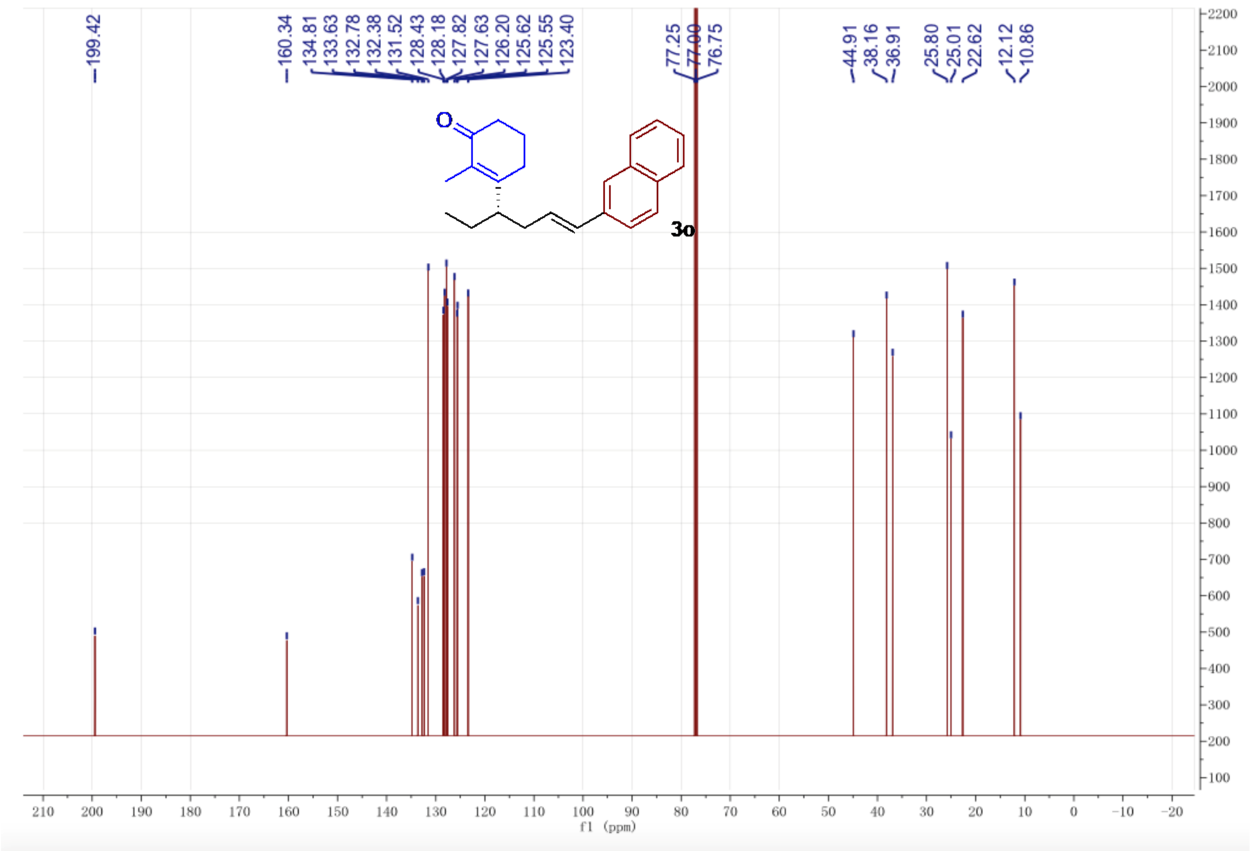

199.42
160.34
134.81
133.63
132.78
132.38
131.52
128.43
128.18
127.82
127.63
126.20
125.62
125.55
123.40
77.25
77.00
76.75
44.91
38.16
36.91
25.80
25.01
22.62
12.12
10.86
O
3o
f1 (ppm)

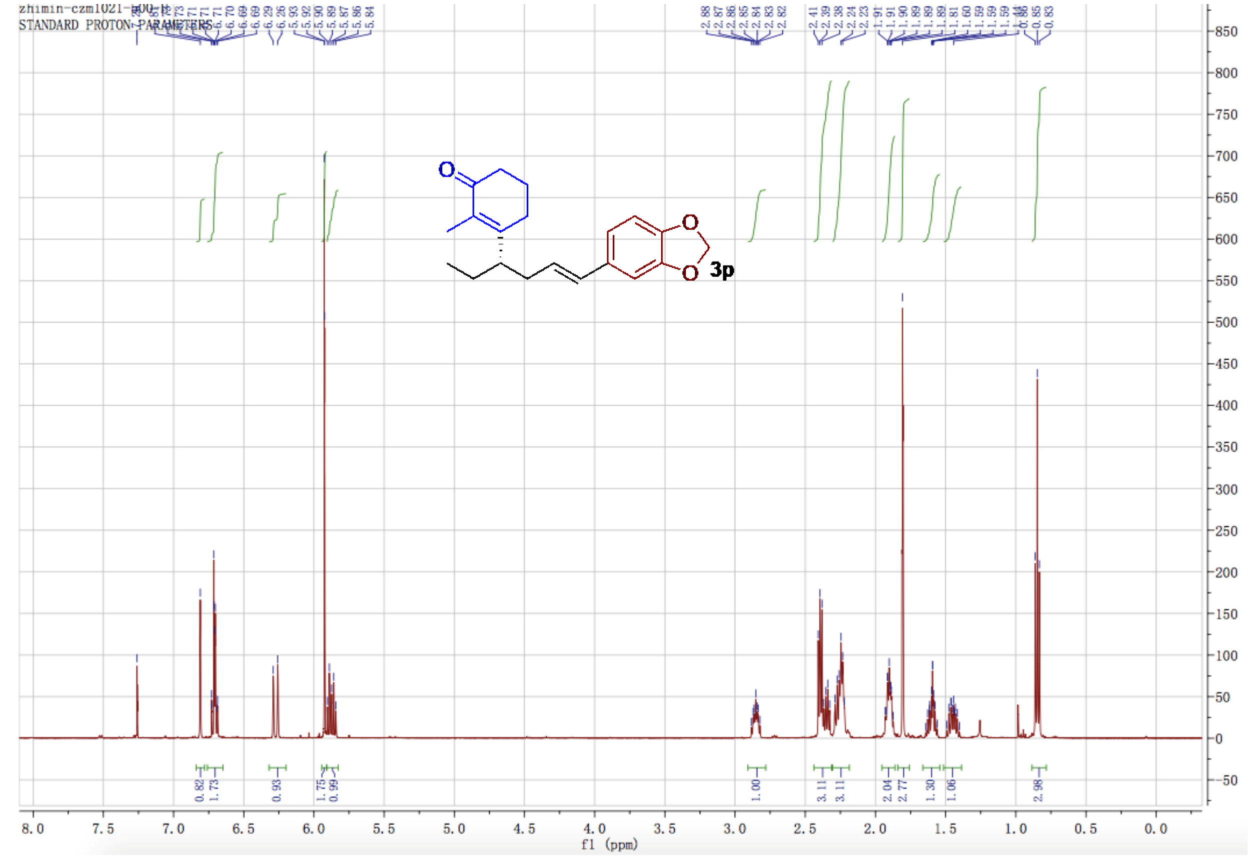
zhimin-czm1021-400-H
STANDARD PROTON PARAMETERS
3p
f1 (ppm)

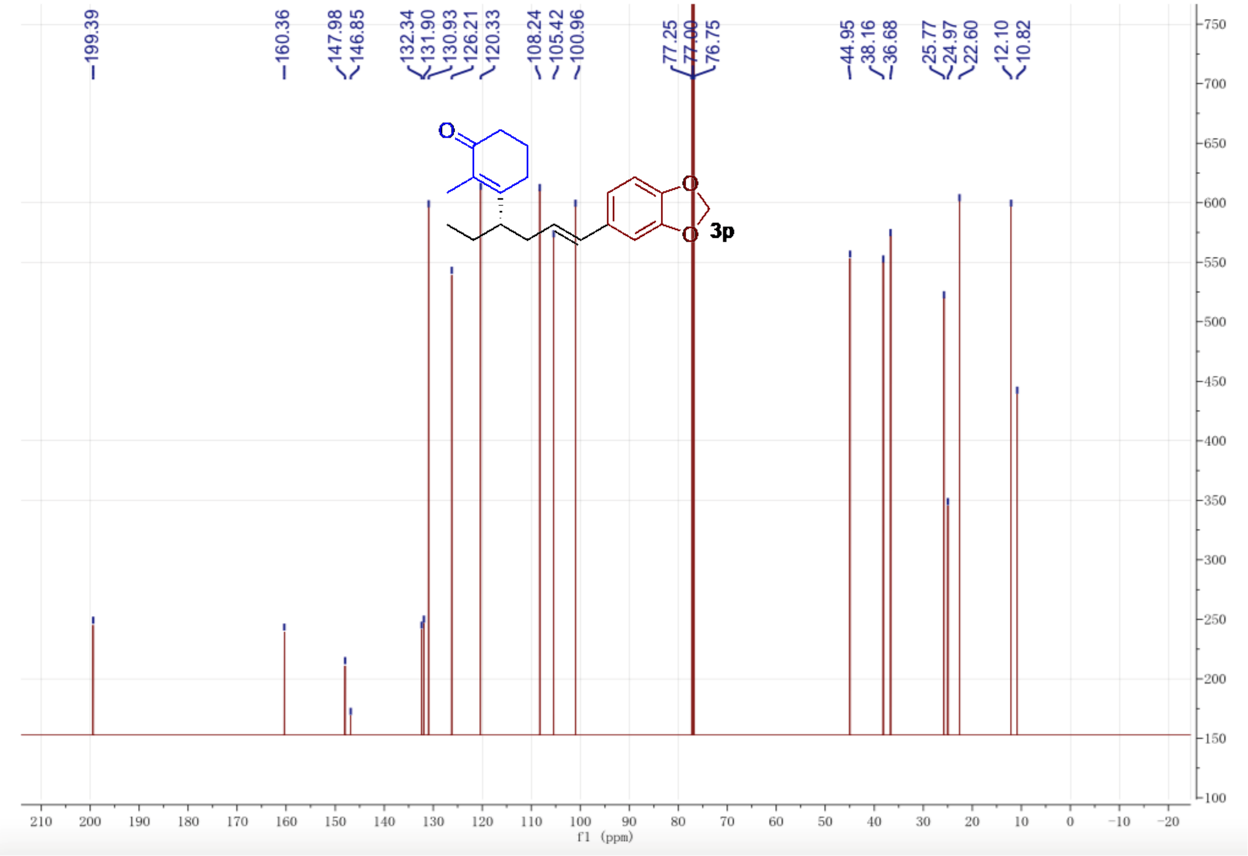
199.39
160.36
147.98
146.85
132.34
131.90
130.93
126.21
120.33
108.24
105.42
100.96
77.25
77.00
76.75
44.95
38.16
36.68
25.77
24.97
22.60
12.10
10.82
3p
f1 (ppm)

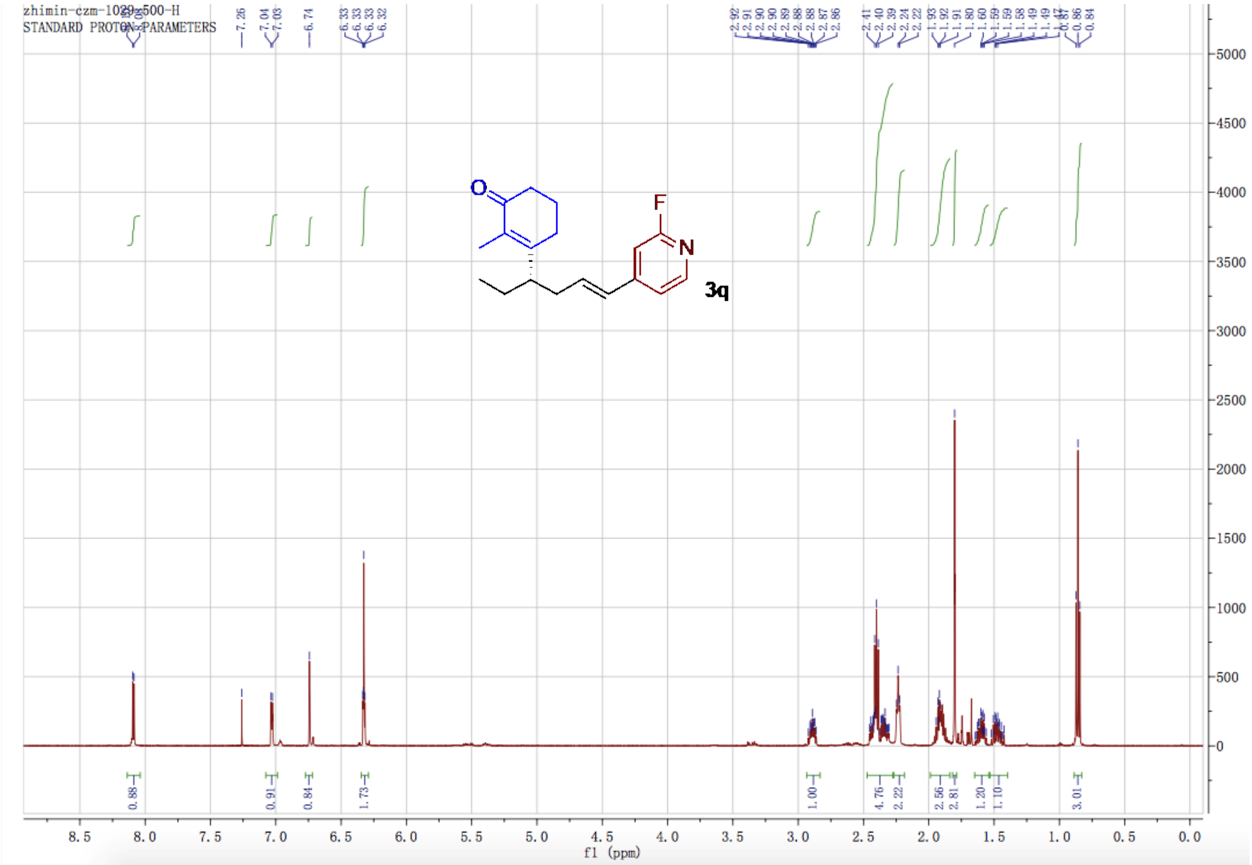

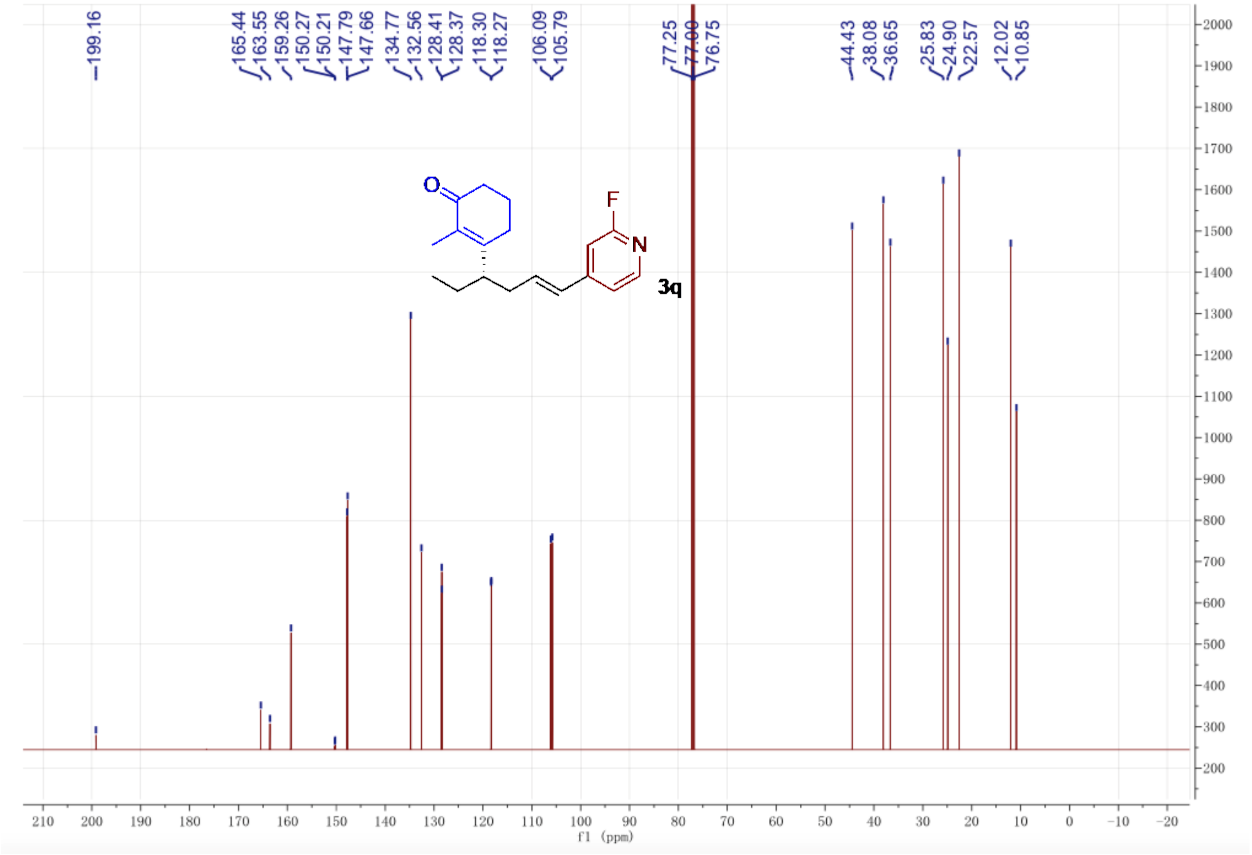

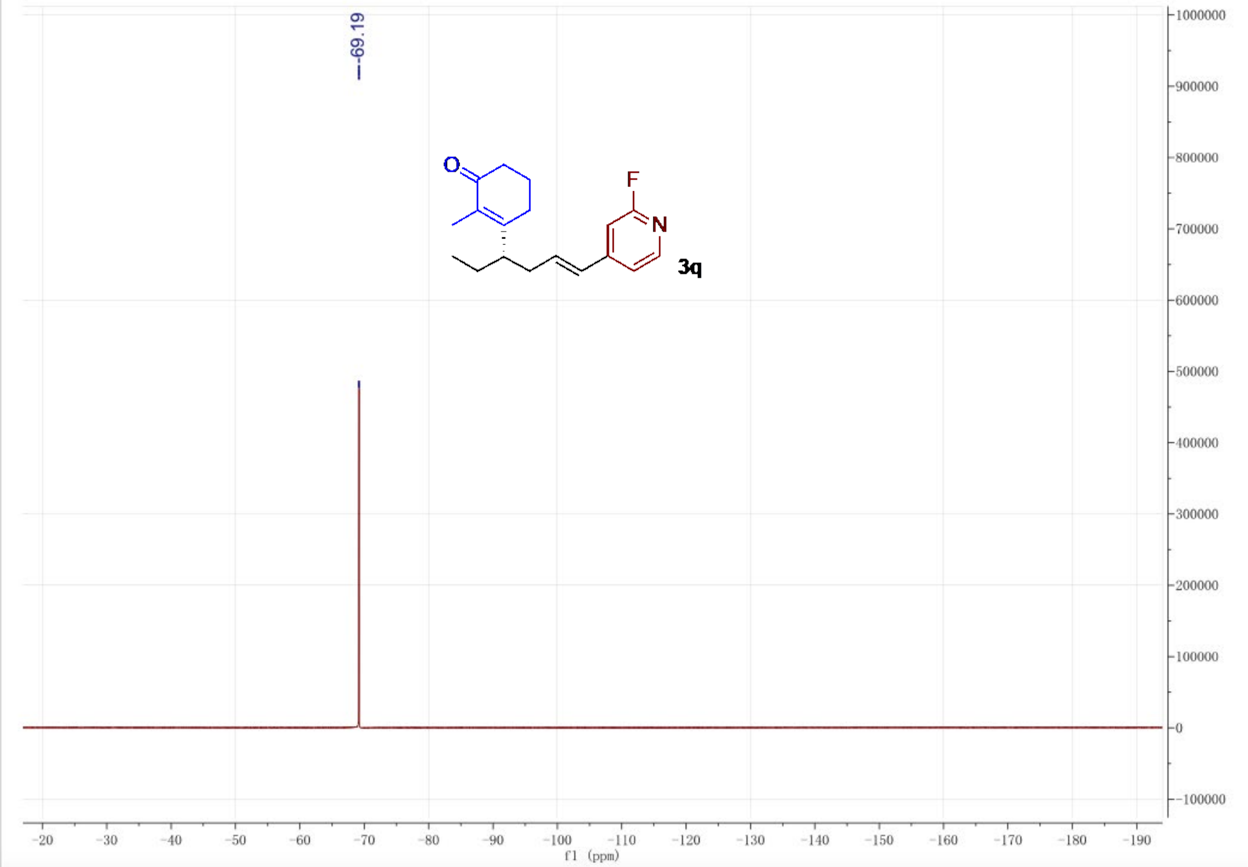

-69.19
O
F
N
3q
f1 (ppm)

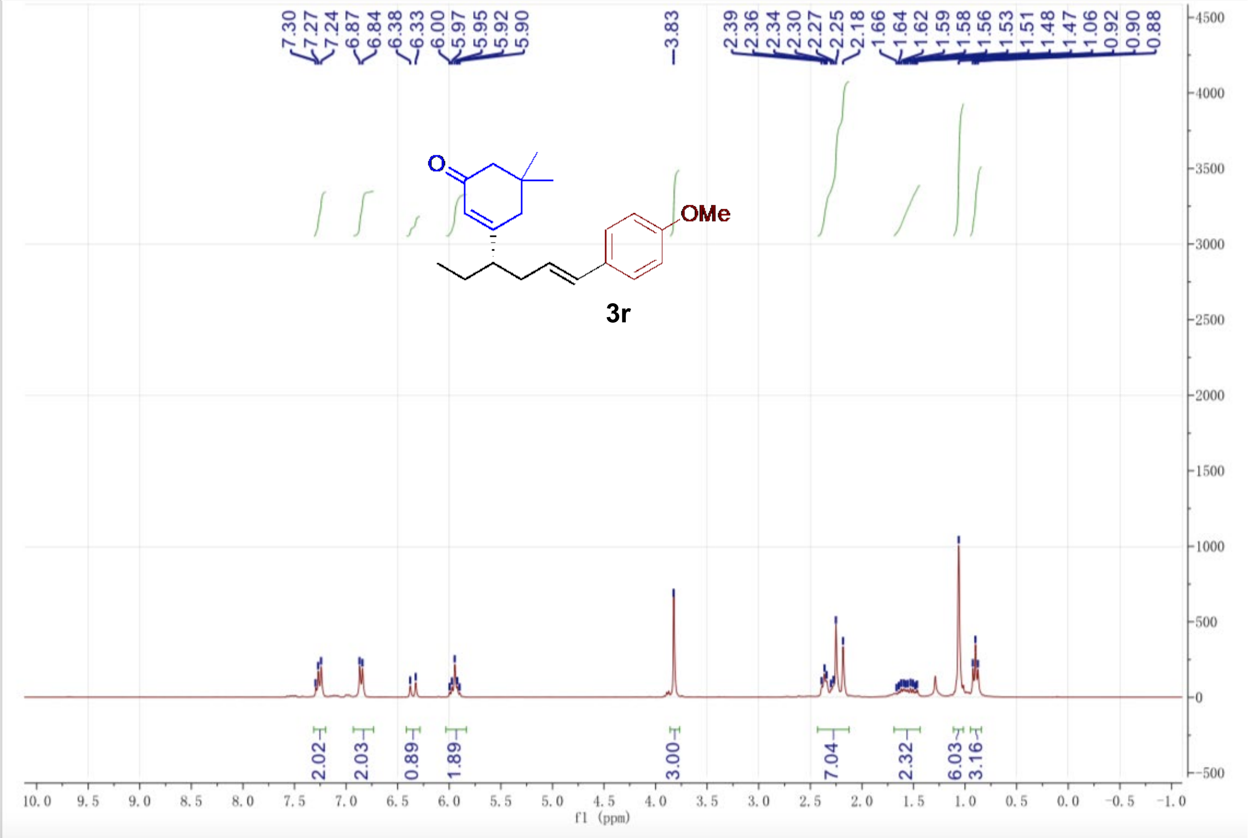

7.30
7.27
7.24
6.87
6.84
6.38
6.33
6.00
5.97
5.95
5.92
5.90
3.83
2.39
2.36
2.34
2.30
2.27
2.25
2.18
1.66
1.64
1.62
1.59
1.58
1.56
1.53
1.51
1.48
1.47
1.06
0.92
0.90
0.88
O
OMe
3r
2.02
2.03
0.89
1.89
3.00
7.04
2.32
6.03
3.16
f1 (ppm)

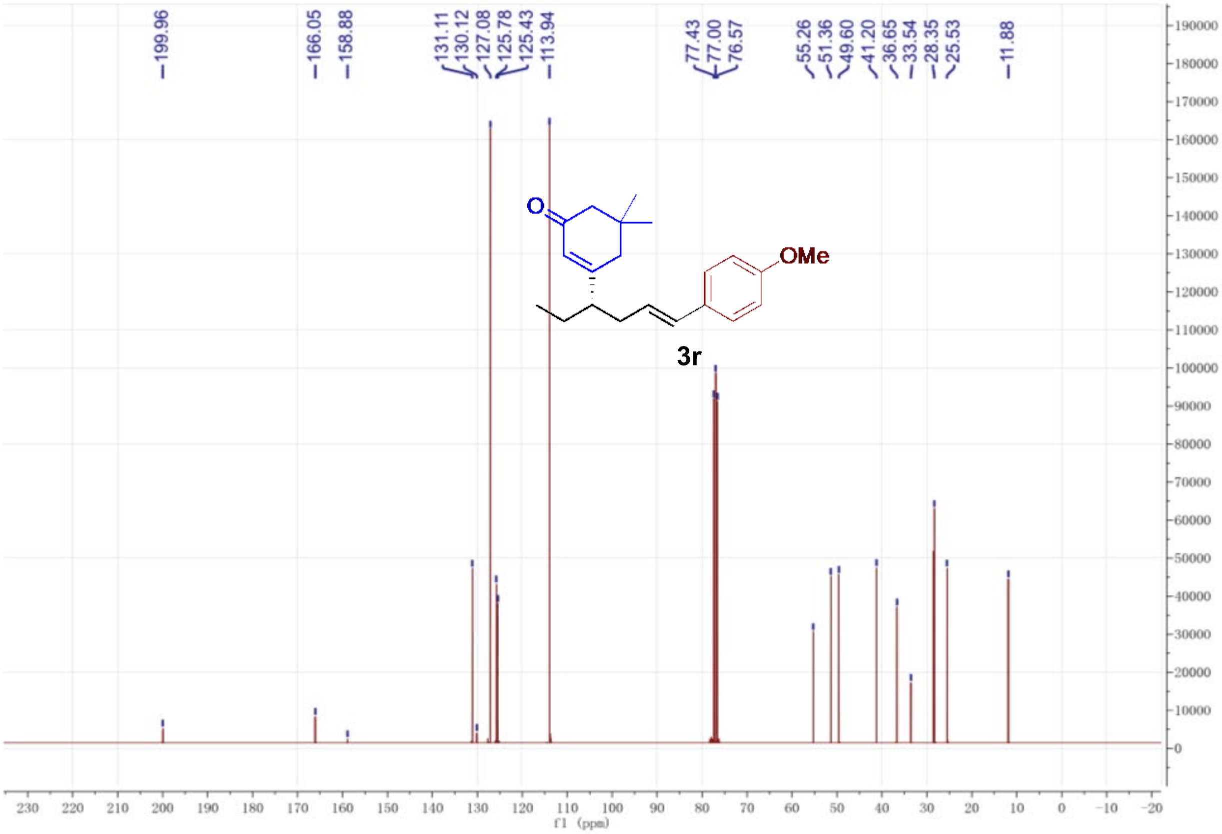
199.96
166.05
158.88
131.11
130.12
127.08
125.78
125.43
113.94
77.43
77.00
76.57
55.26
51.36
49.60
41.20
36.65
33.54
28.35
25.53
11.88
O
OMe
3r
f1 (ppm)

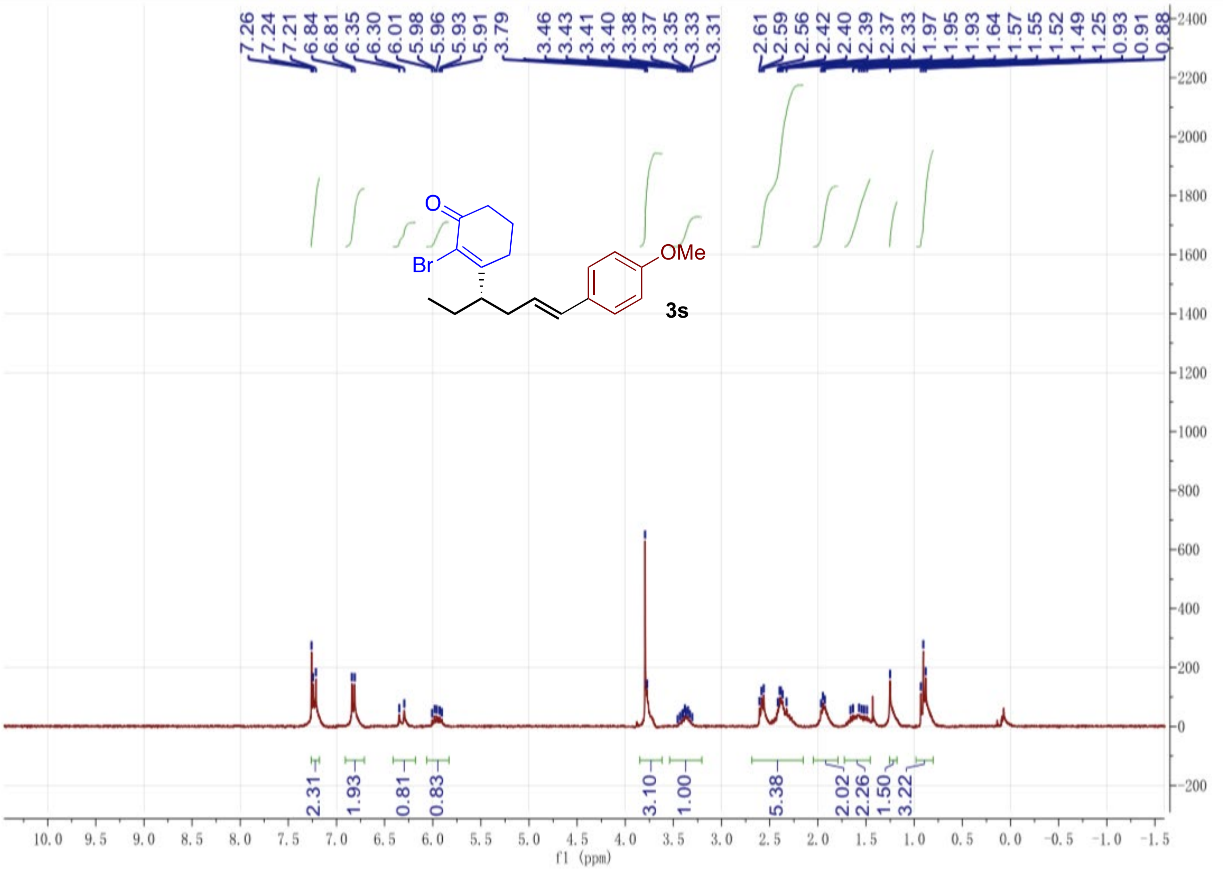
7.26
7.24
7.21
6.84
6.81
6.35
6.30
6.01
5.98
5.96
5.93
5.91
3.79
3.46
3.43
3.41
3.40
3.38
3.37
3.35
3.33
3.31
2.61
2.59
2.56
2.42
2.40
2.39
2.37
2.33
1.97
1.95
1.93
1.64
1.57
1.55
1.52
1.49
1.25
0.93
0.91
0.88
O
Br
OMe
3s
2.31
1.93
0.81
0.83
3.10
1.00
5.38
2.02
2.26
1.50
3.22
f1 (ppm)

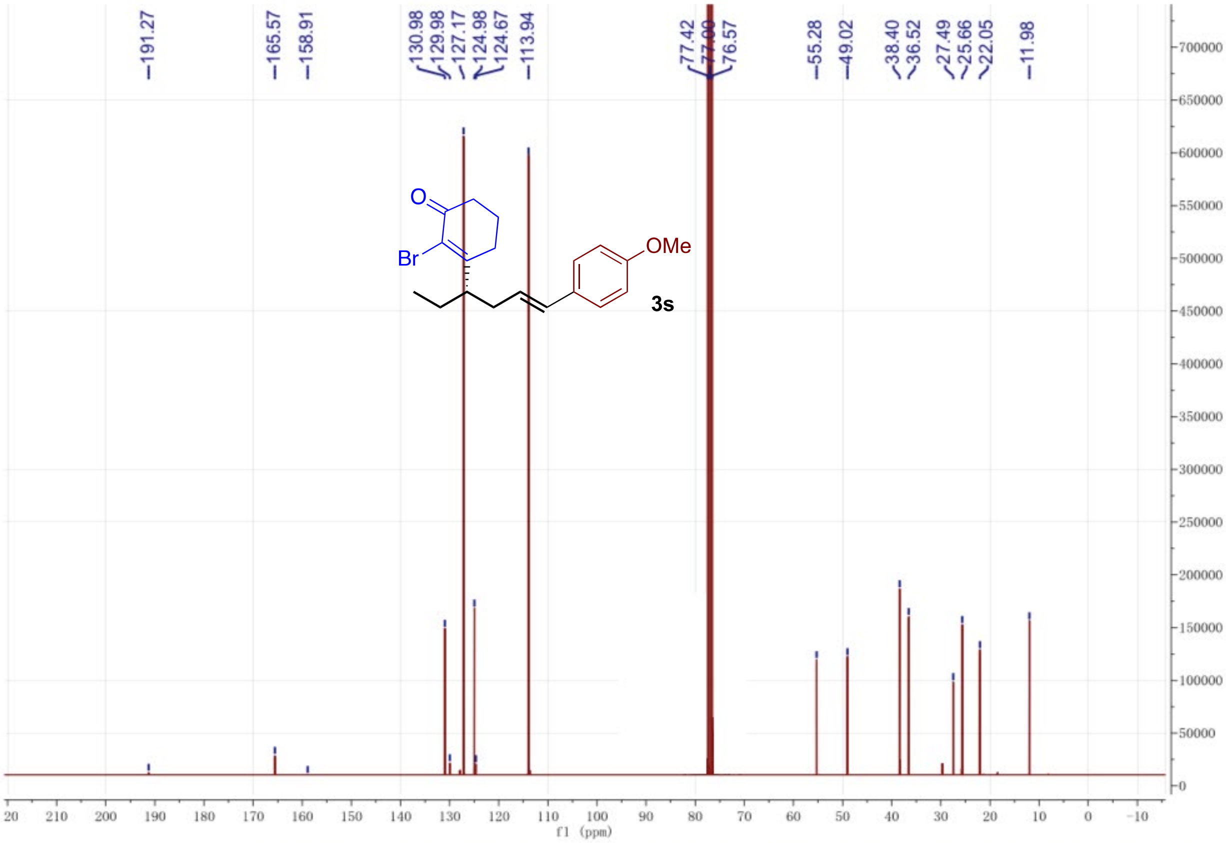

191.27
165.57
158.91
130.98
129.98
127.17
124.98
124.67
113.94
77.42
77.00
76.57
55.28
49.02
38.40
36.52
27.49
25.66
22.05
11.98
O
Br
OMe
3s
f1 (ppm)

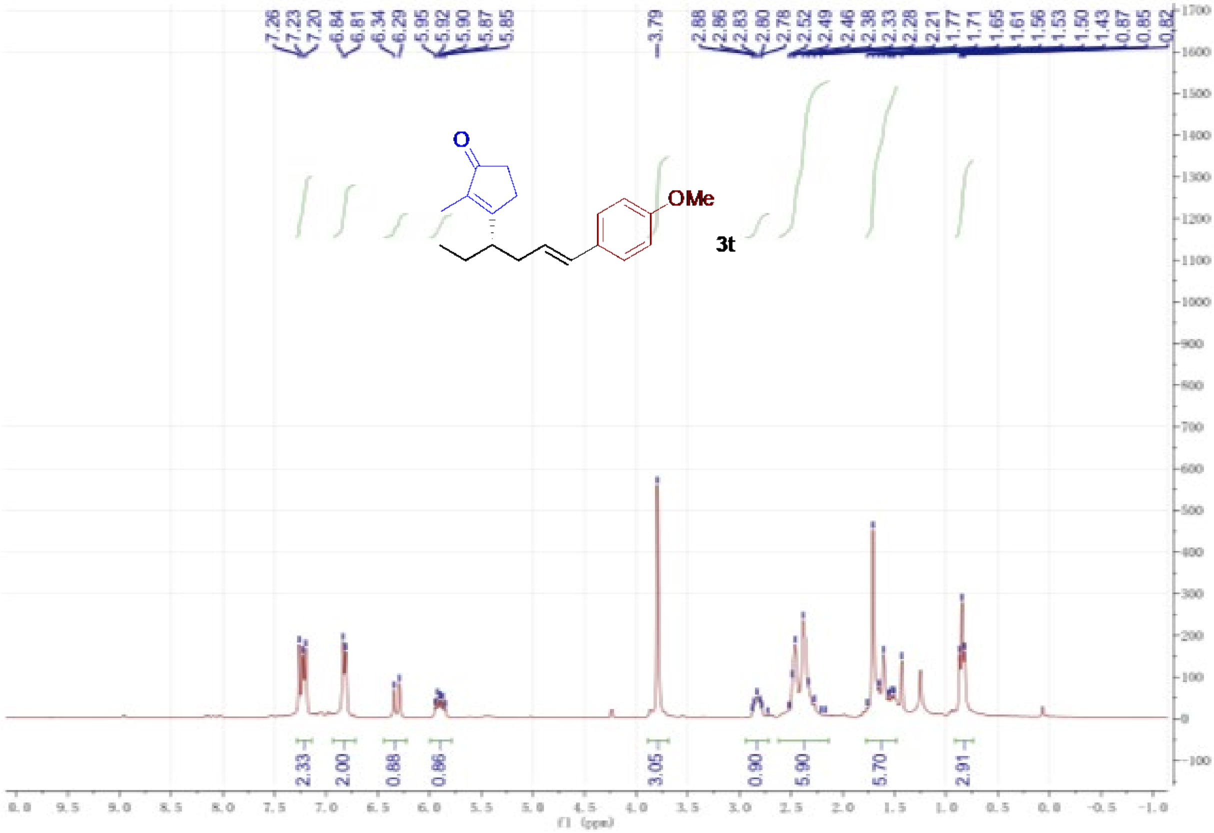

7.26
7.23
7.20
6.84
6.81
6.34
6.29
5.95
5.92
5.90
5.87
5.85
3.79
2.88
2.86
2.83
2.80
2.78
2.52
2.49
2.46
2.38
2.33
2.28
2.21
1.77
1.71
1.65
1.61
1.56
1.53
1.50
1.43
0.87
0.85
0.82
O
OMe
3t
2.33
2.00
0.88
0.86
3.05
0.90
5.90
5.70
2.91
f1 (ppm)

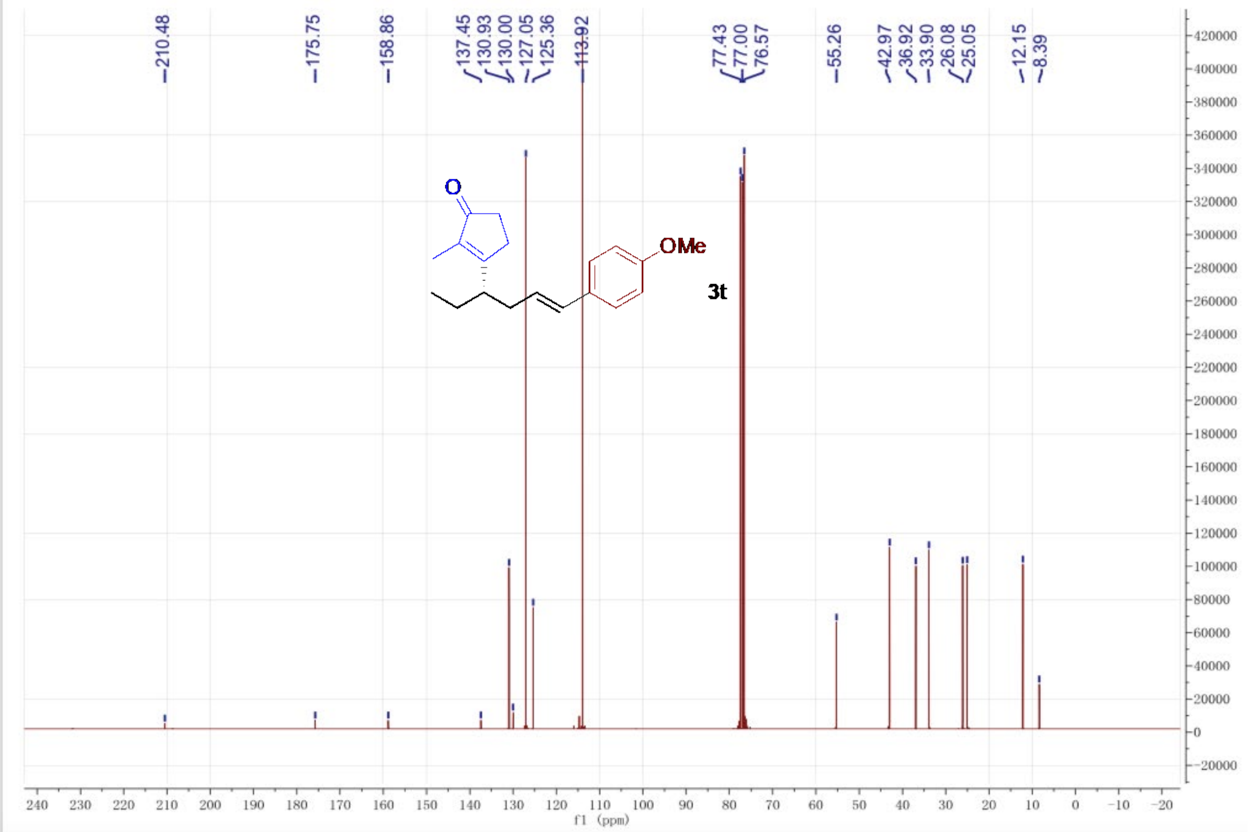
210.48
175.75
158.86
137.45
130.93
130.00
127.05
125.36
113.92
77.43
77.00
76.57
55.26
42.97
36.92
33.90
26.08
25.05
12.15
8.39
OMe
3t
f1 (ppm)

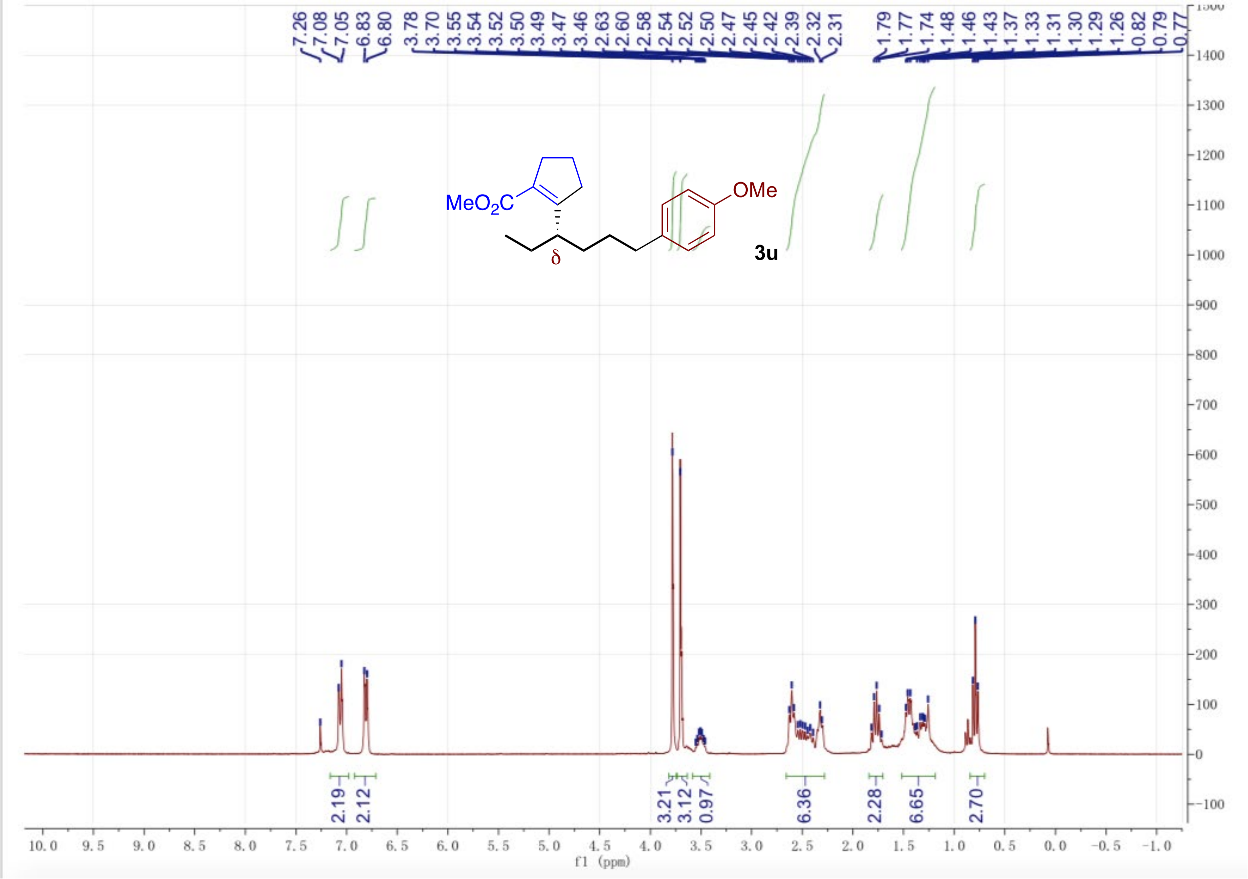
7.26
7.08
7.05
6.83
6.80
3.78
3.70
3.55
3.54
3.52
3.50
3.49
3.47
3.46
2.63
2.60
2.58
2.54
2.52
2.50
2.47
2.45
2.42
2.39
2.32
2.31
1.79
1.77
1.74
1.48
1.46
1.43
1.37
1.33
1.31
1.30
1.29
1.26
0.82
0.79
0.77
MeO_2C
OMe
δ
3u
2.19
2.12
3.21
3.12
0.97
6.36
2.28
6.65
2.70
f1 (ppm)

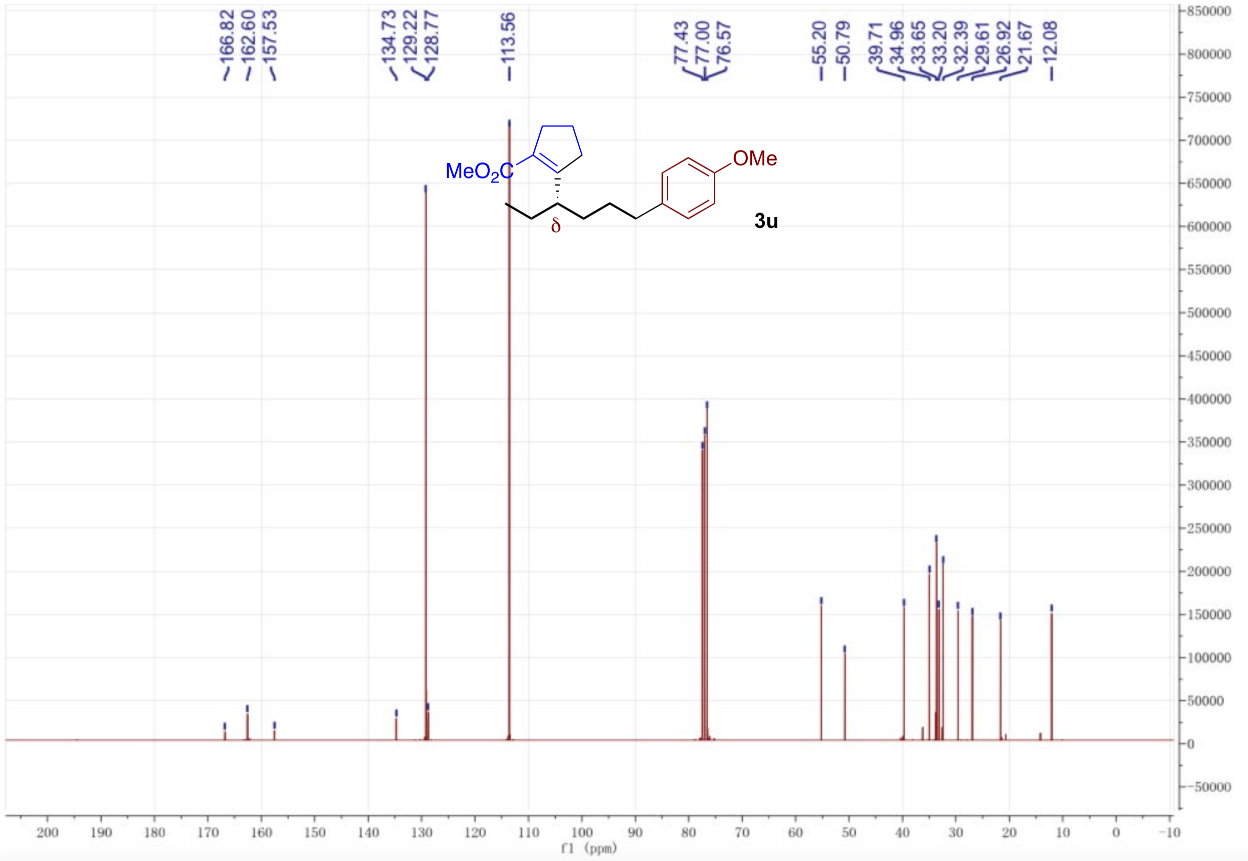
166.82
162.60
157.53
134.73
129.22
128.77
113.56
77.43
77.00
76.57
55.20
50.79
39.71
34.96
33.65
33.20
32.39
29.61
26.92
21.67
12.08
MeO2C
OMe
δ
3u
f1 (ppm)

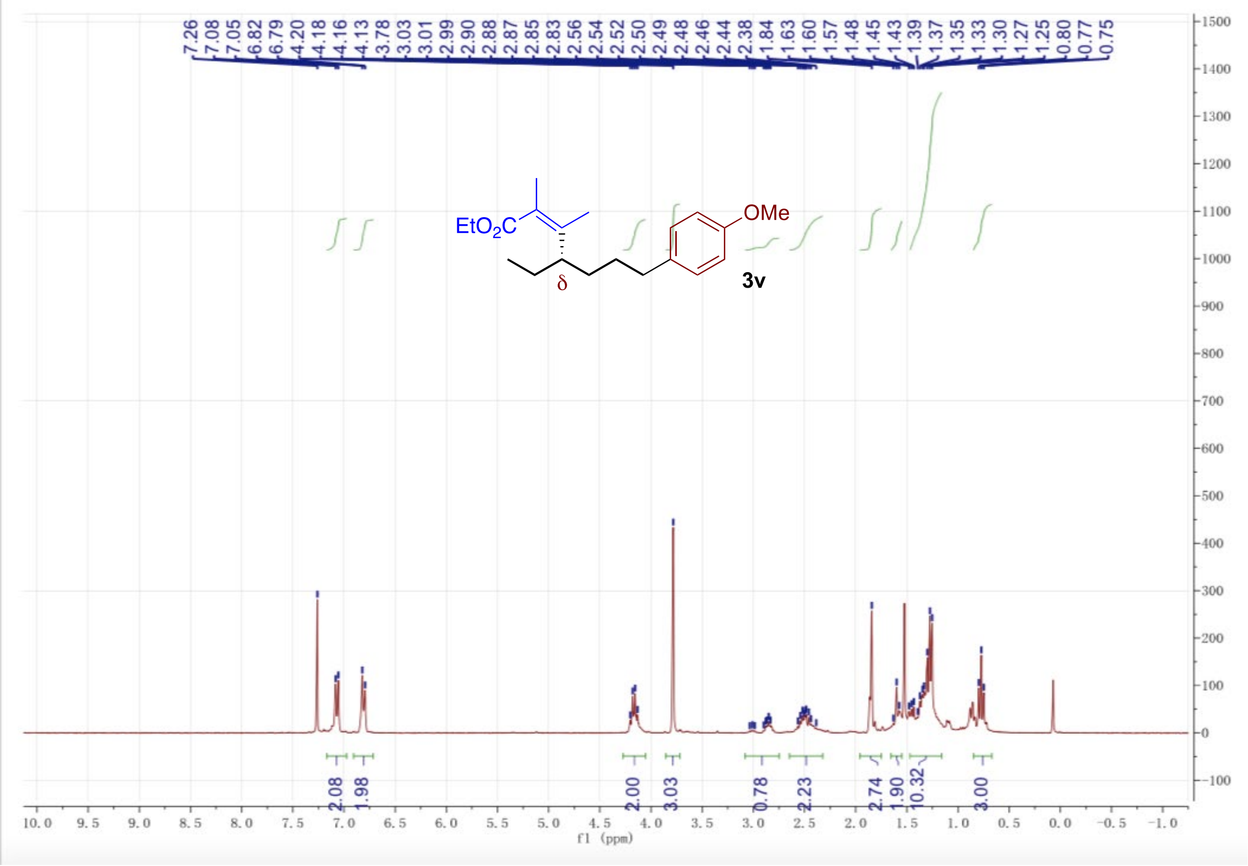
7.26
7.08
7.05
6.82
6.79
4.20
4.18
4.16
4.13
3.78
3.03
3.01
2.99
2.90
2.88
2.87
2.85
2.83
2.56
2.54
2.52
2.50
2.49
2.48
2.46
2.44
2.38
1.84
1.63
1.60
1.57
1.48
1.45
1.43
1.39
1.37
1.35
1.33
1.30
1.27
1.25
0.80
0.77
0.75
EtO2C
OMe
δ
3v
2.08
1.98
2.00
3.03
0.78
2.23
2.74
1.90
10.32
3.00
f1 (ppm)

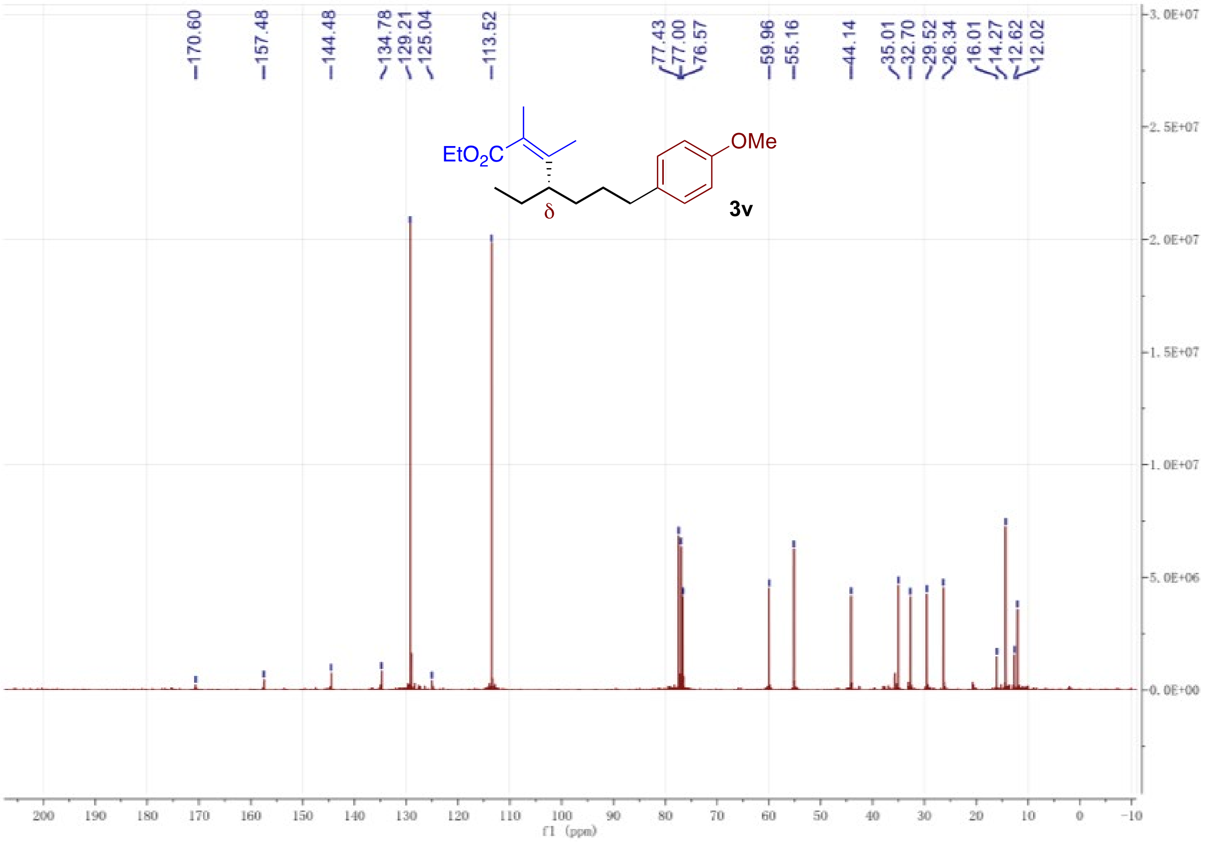

170.60
157.48
144.48
134.78
129.21
125.04
113.52
77.43
77.00
76.57
59.96
55.16
44.14
35.01
32.70
29.52
26.34
16.01
14.27
12.62
12.02
EtO2C
OMe
δ
3v
f1 (ppm)

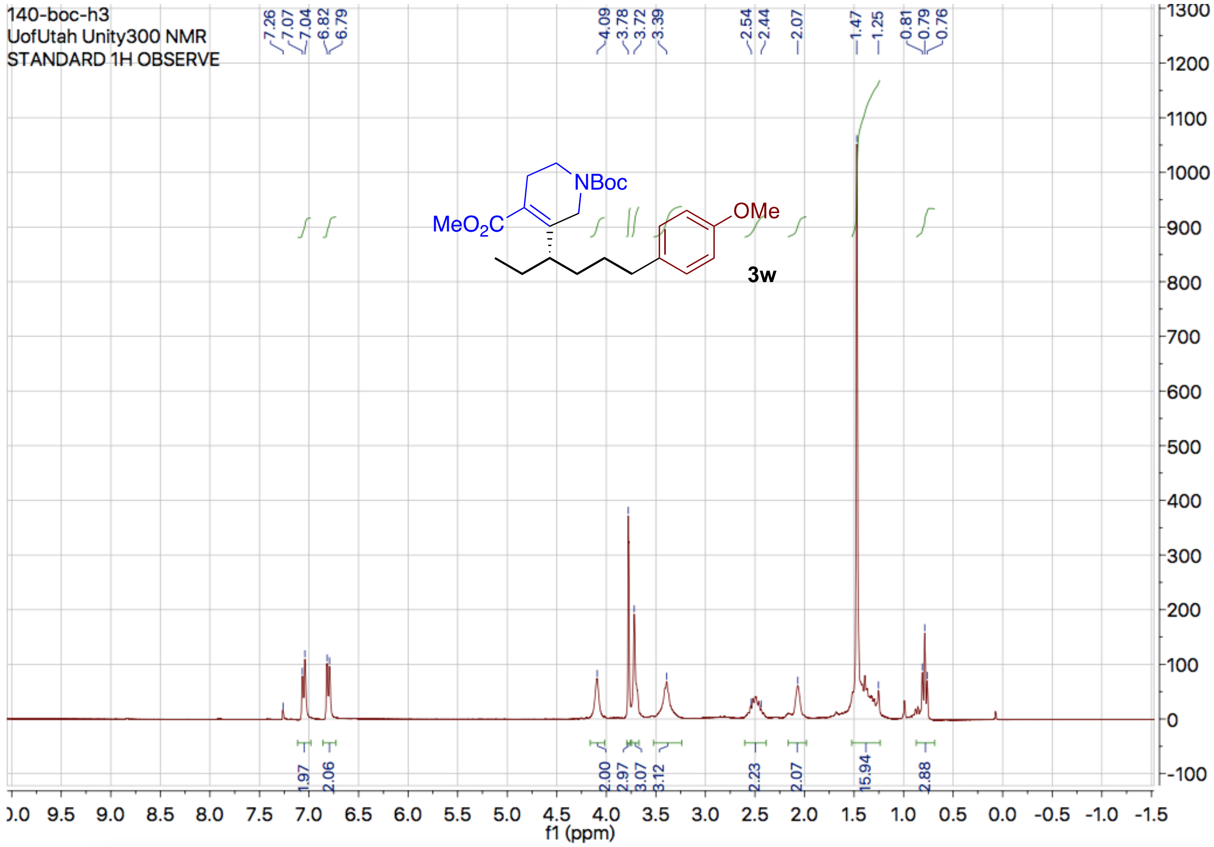

140-boc-h3
UofUtah Unity300 NMR
STANDARD 1H OBSERVE
7.26
7.07
7.04
6.82
6.79
4.09
3.78
3.72
3.39
2.54
2.44
2.07
1.47
1.25
0.81
0.79
0.76
NBoc
MeO2C
OMe
3w
1.97
2.06
2.00
2.97
3.07
3.12
2.23
2.07
15.94
2.88
f1 (ppm)

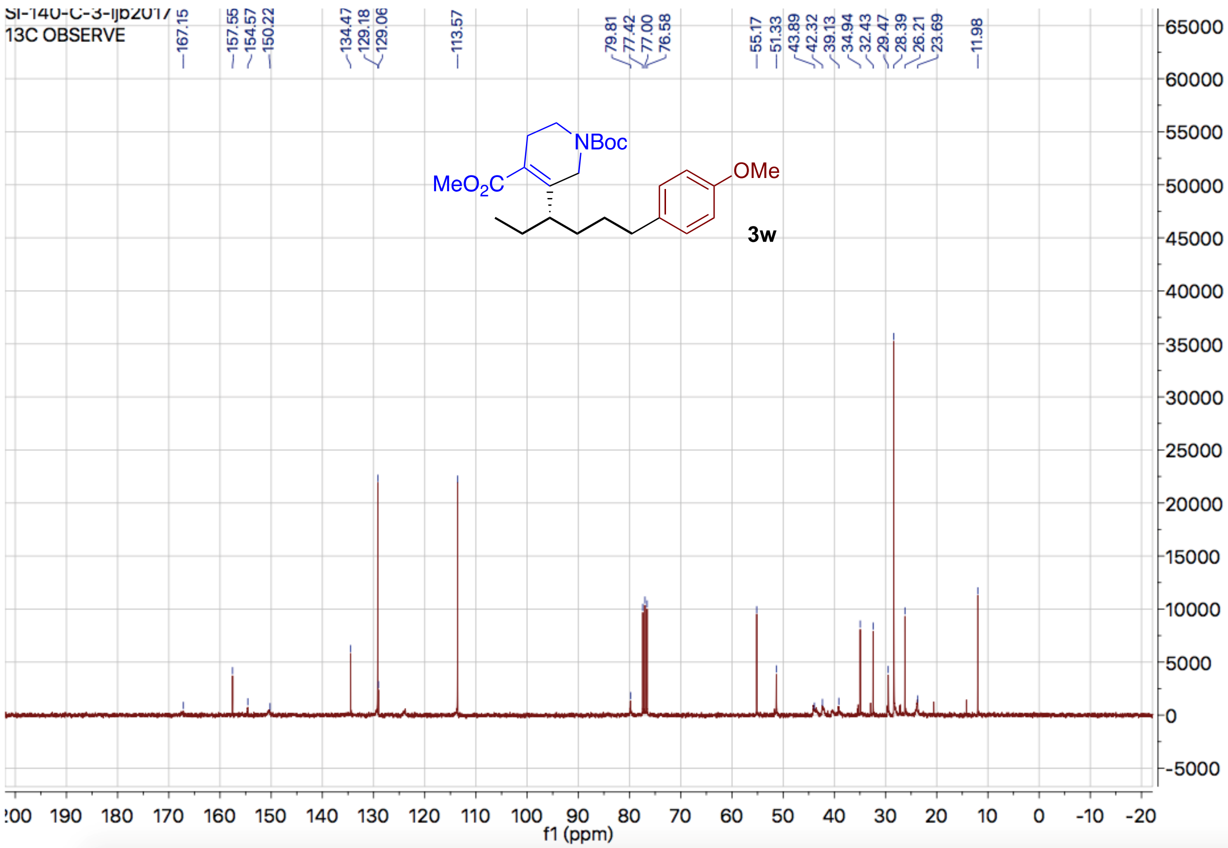
SI-140-C-3-ljb2017
13C OBSERVE
NBoc
MeO2C
OMe
3w
f1 (ppm)

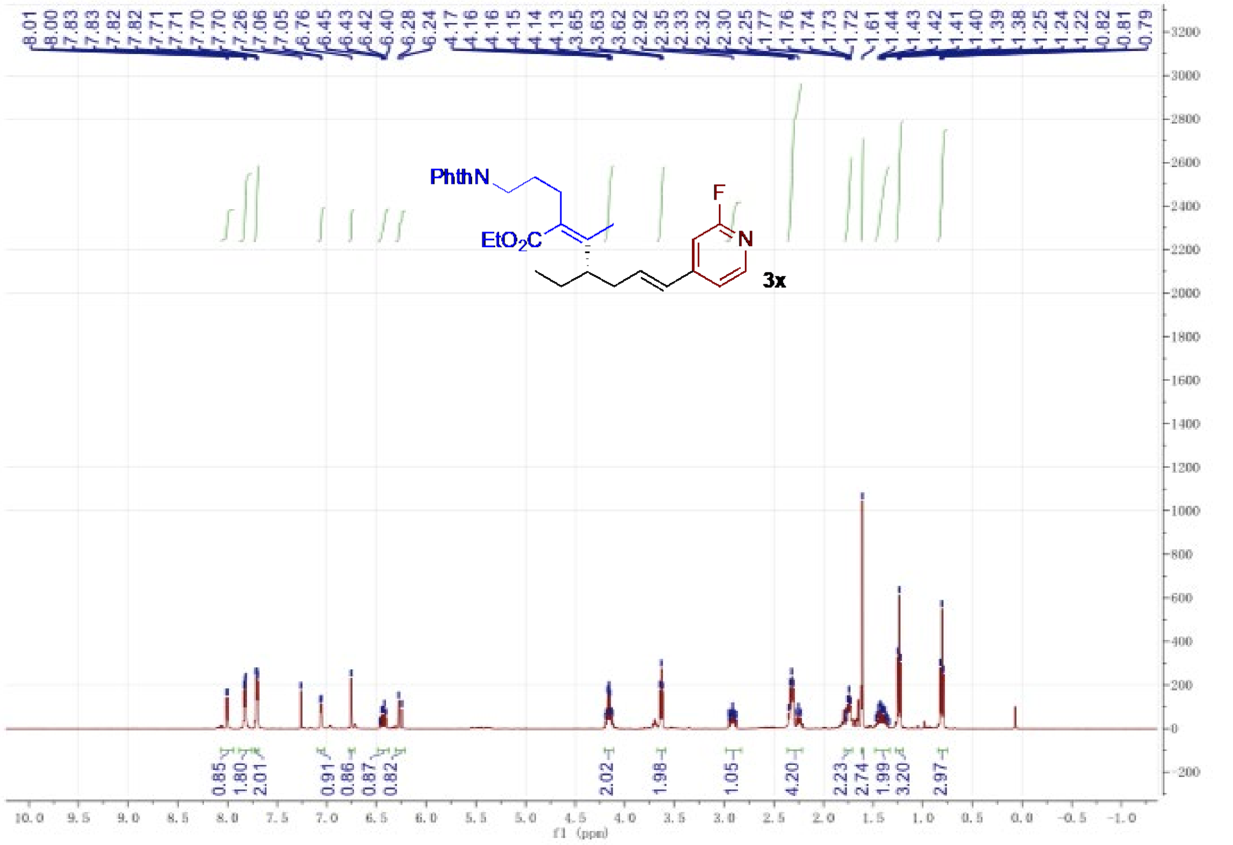
PhthN
EtO2C
F
N
3x
f1 (ppm)

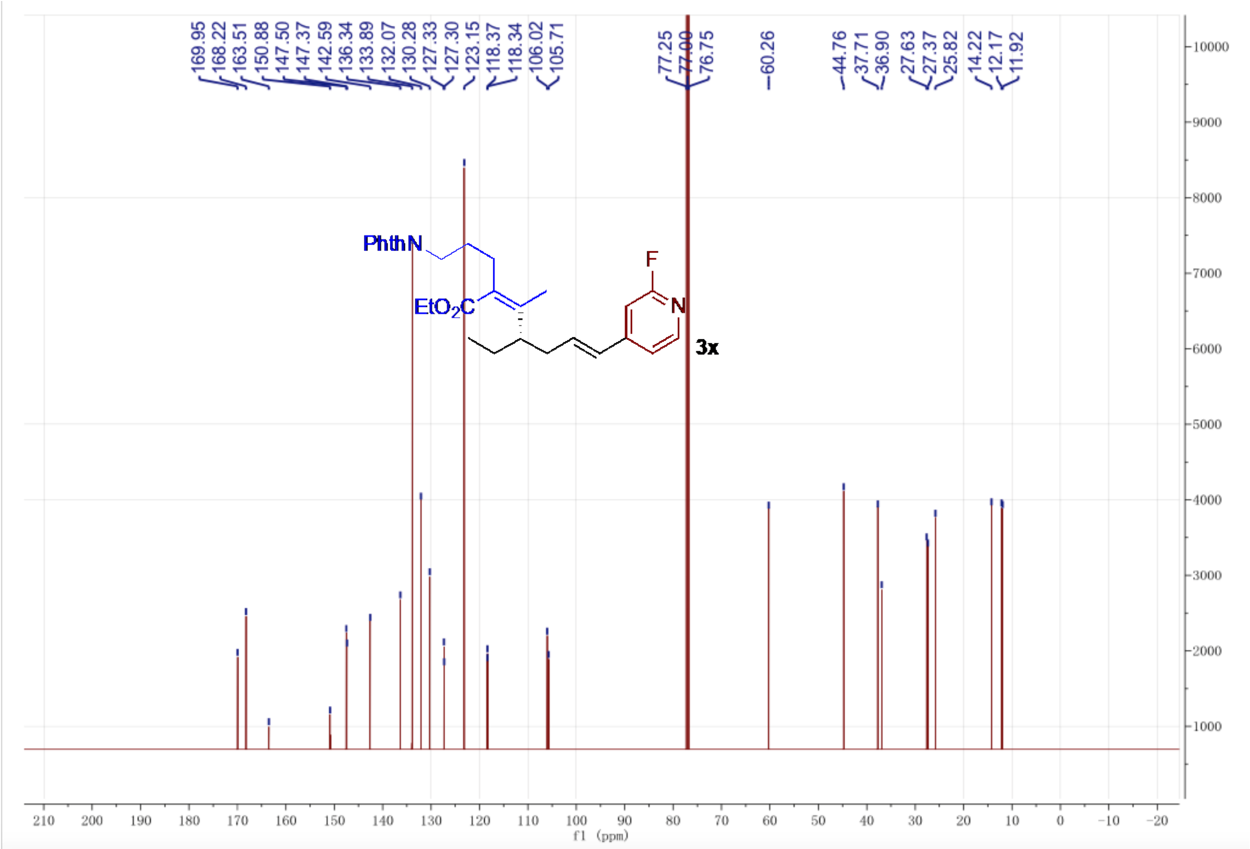
169.95
168.22
163.51
150.88
147.50
147.37
142.59
136.34
133.89
132.07
130.28
127.33
127.30
123.15
118.37
118.34
106.02
105.71
77.25
77.00
76.75
60.26
44.76
37.71
36.90
27.63
27.37
25.82
14.22
12.17
11.92
PhthN
EtO2C
F
N
3x
f1 (ppm)

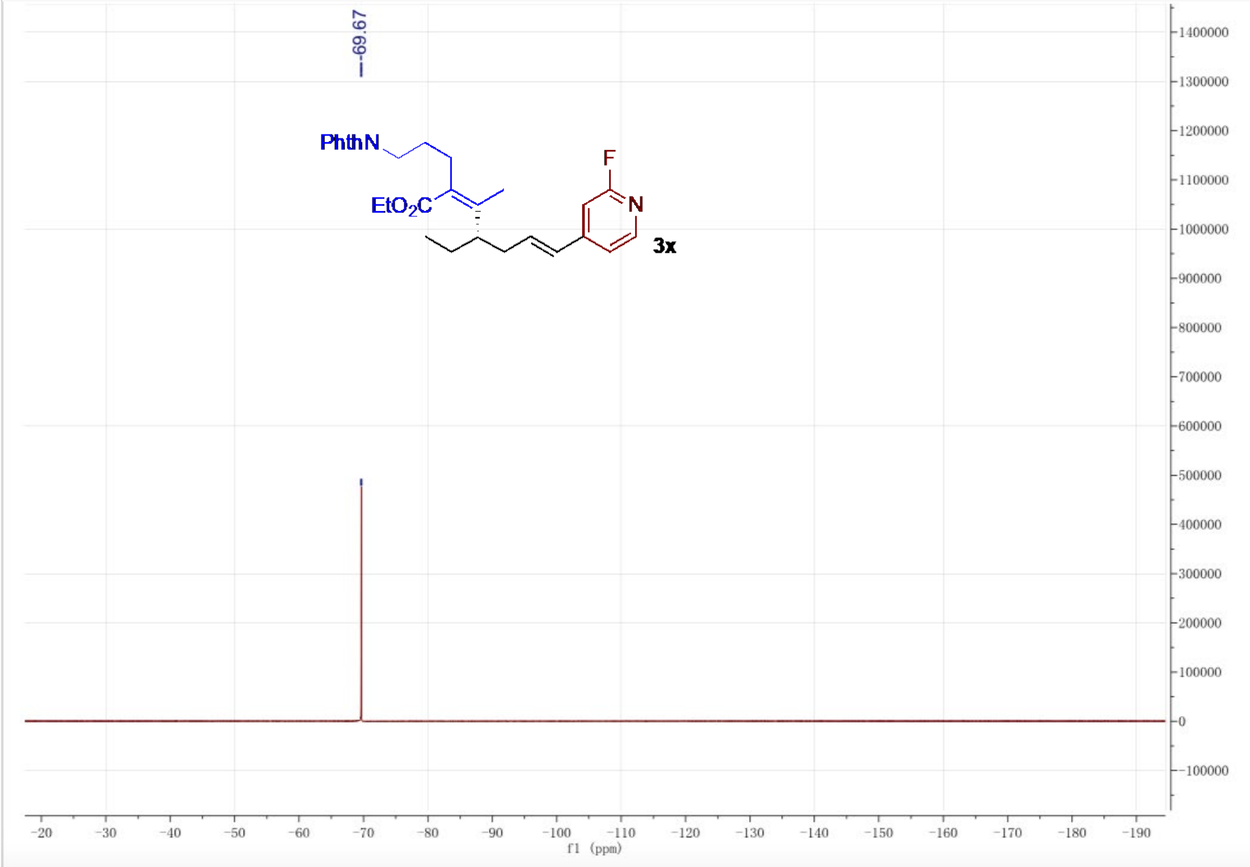
-69.67
PhthN
EtO2C
F
N
3x
f1 (ppm)

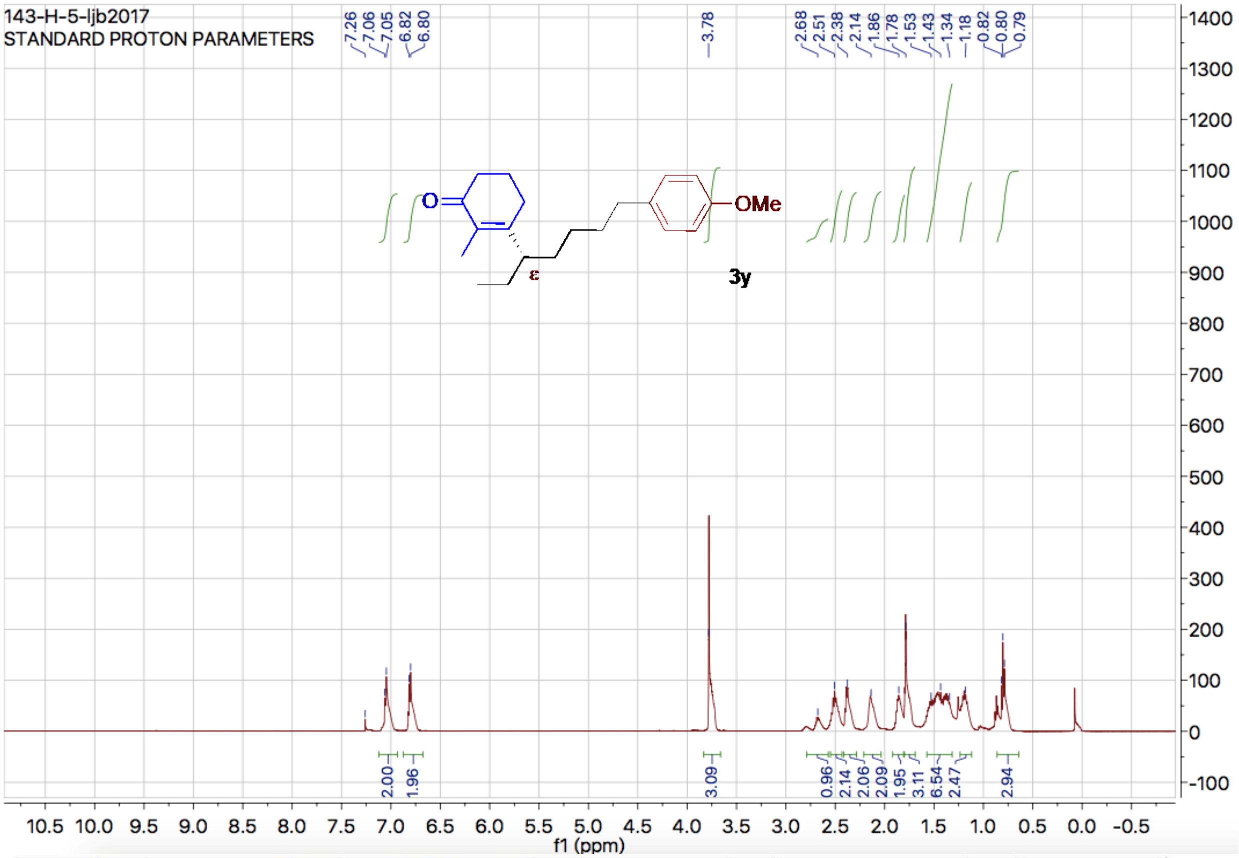
143-H-5-ljb2017
STANDARD PROTON PARAMETERS
7.26
7.06
7.05
6.82
6.80
3.78
2.68
2.51
2.38
2.14
1.86
1.78
1.53
1.43
1.34
1.18
0.82
0.80
0.79
O
OMe
ε
3y
2.00
1.96
3.09
0.96
2.14
2.06
2.09
1.95
3.11
6.54
2.47
2.94
f1 (ppm)

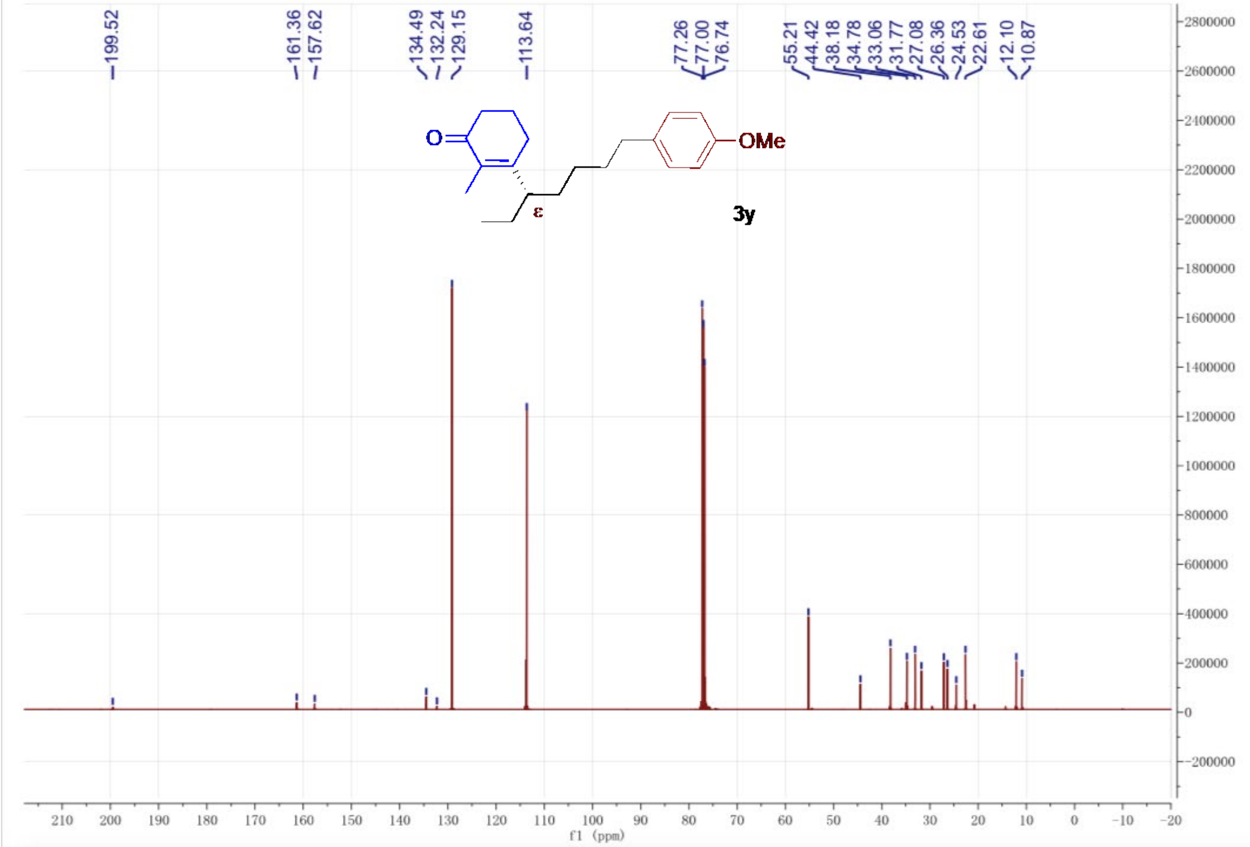
199.52
161.36
157.62
134.49
132.24
129.15
113.64
77.26
77.00
76.74
55.21
44.42
38.18
34.78
33.06
31.77
27.08
26.36
24.53
22.61
12.10
10.87
O
OMe
ε
3y
f1 (ppm)

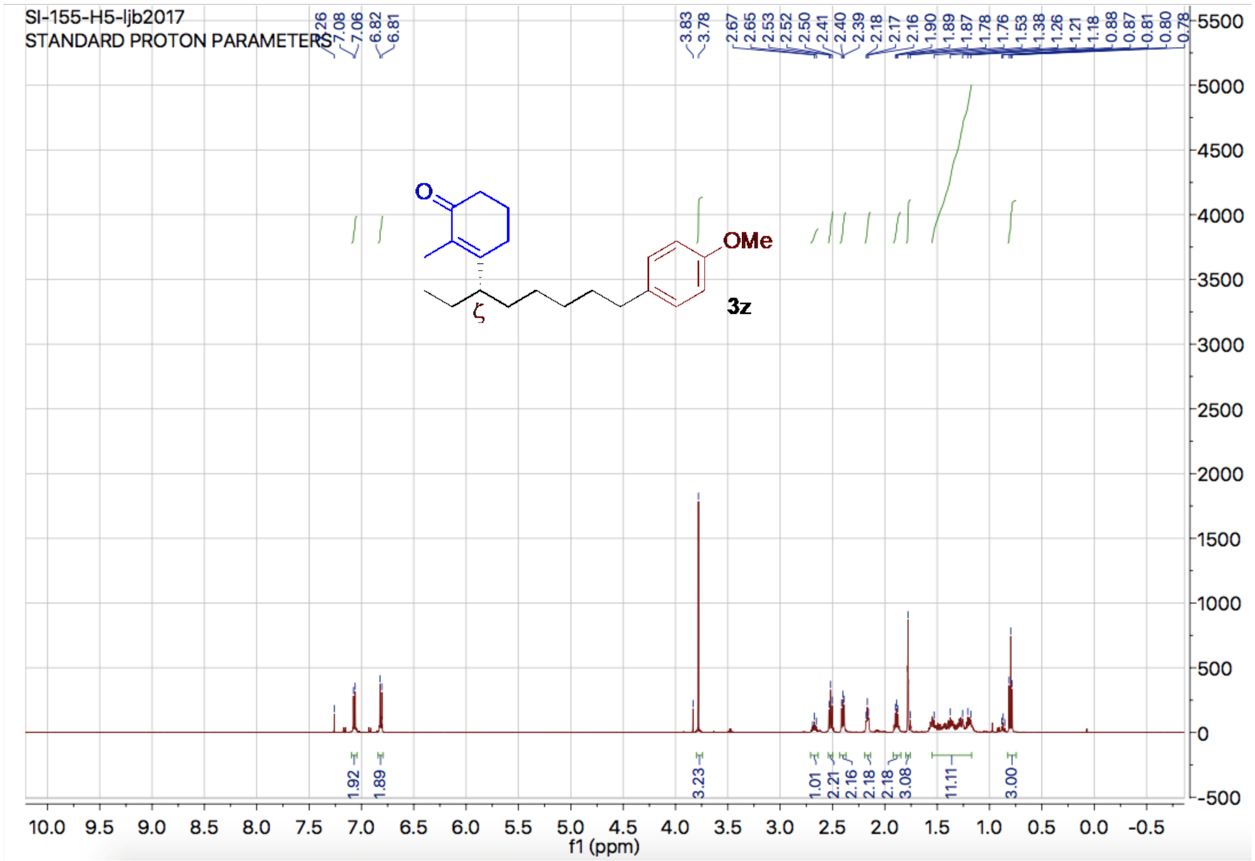
SI-155-H5-ljb2017
STANDARD PROTON PARAMETERS
7.26
7.08
7.06
6.82
6.81
3.83
3.78
2.67
2.65
2.53
2.52
2.50
2.41
2.40
2.39
2.18
2.17
2.16
1.90
1.89
1.87
1.87
1.78
1.76
1.53
1.38
1.26
1.21
1.18
0.88
0.87
0.81
0.80
0.78
O
OMe
ζ
3z
1.92
1.89
3.23
1.01
2.21
2.16
2.18
2.18
3.08
11.11
3.00
f1 (ppm)

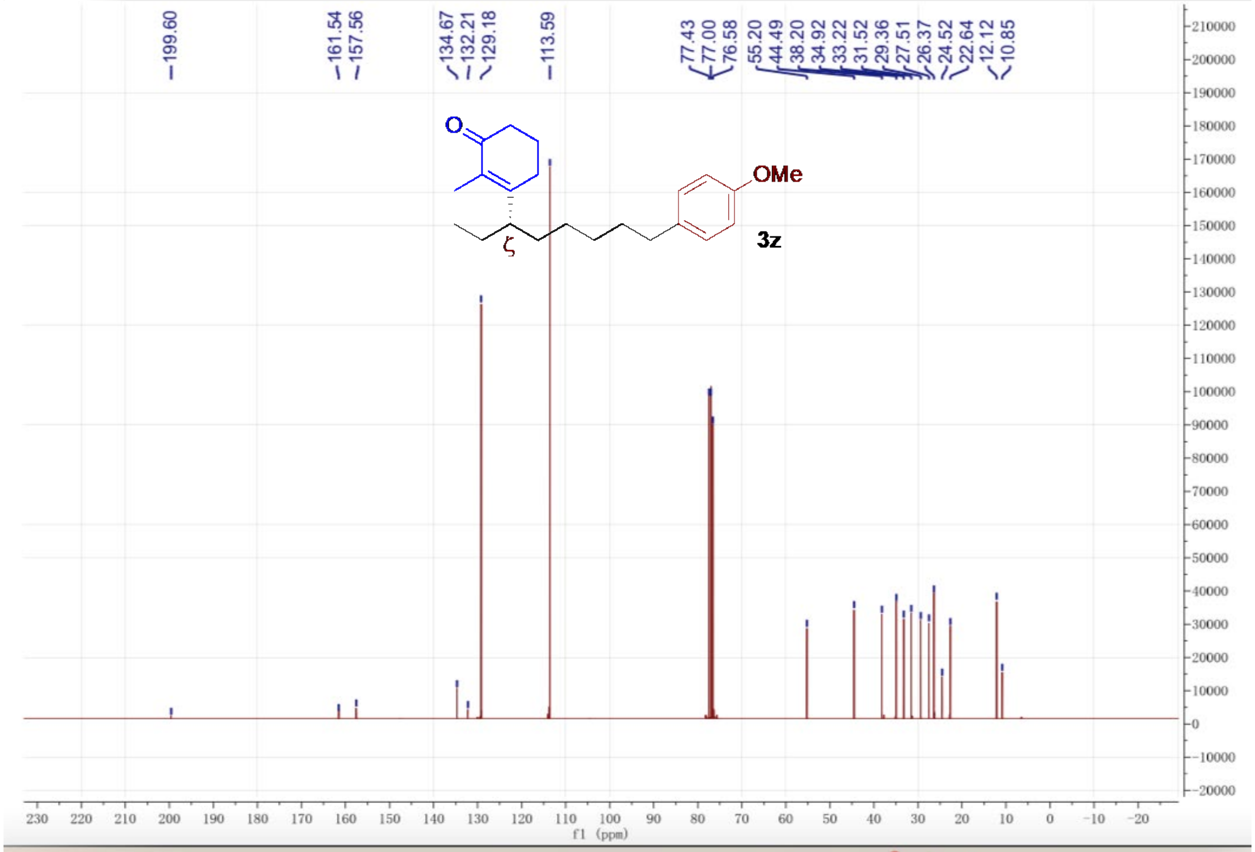
199.60
161.54
157.56
134.67
132.21
129.18
113.59
77.43
77.00
76.58
55.20
44.49
38.20
34.92
33.22
31.52
29.36
27.51
26.37
24.52
22.64
12.12
10.85
O
OMe
ζ
3z
f1 (ppm)

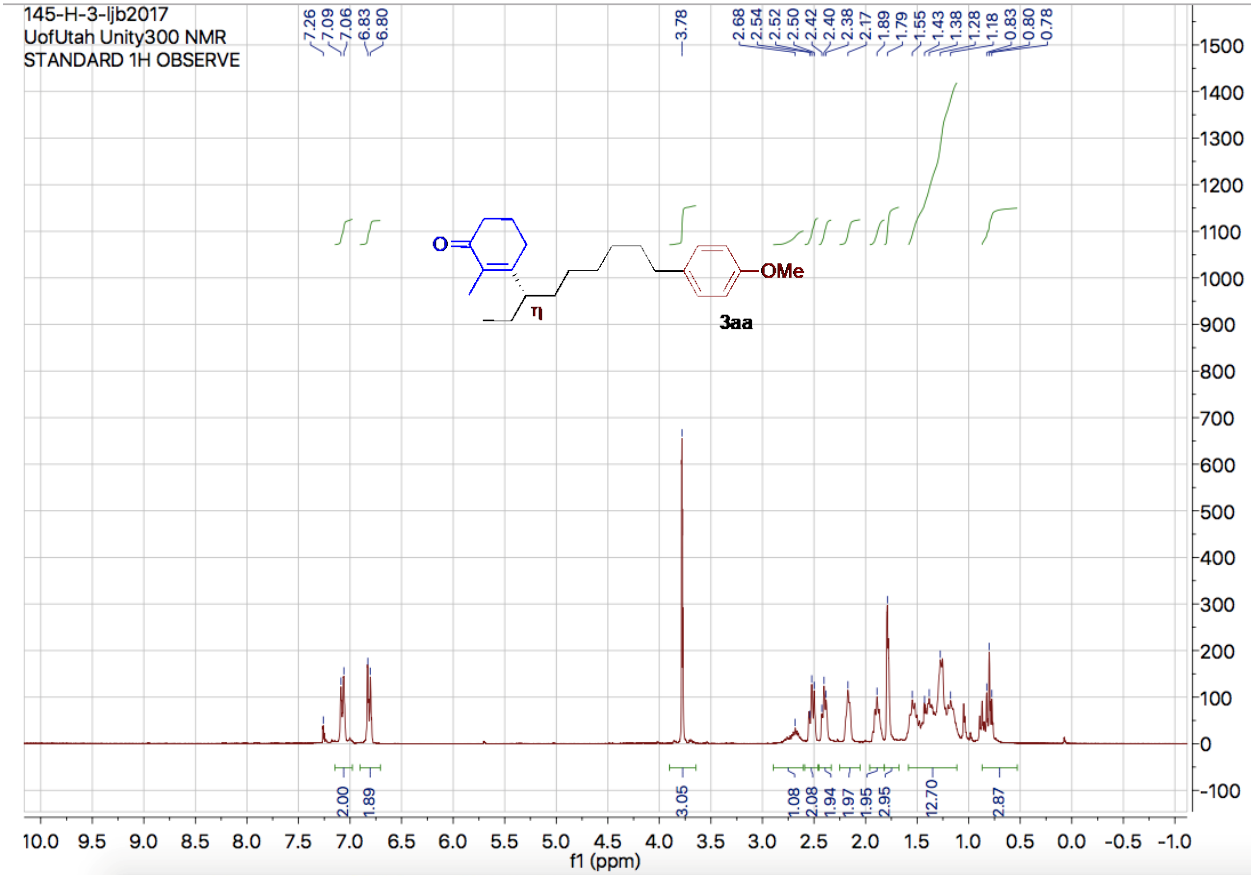
145-H-3-ljb2017
UofUtah Unity300 NMR
STANDARD 1H OBSERVE
7.26
7.09
7.06
6.83
6.80
3.78
2.68
2.54
2.52
2.50
2.42
2.40
2.38
2.17
1.89
1.79
1.55
1.43
1.38
1.28
1.18
0.83
0.80
0.78
OMe
3aa
2.00
1.89
3.05
1.08
2.08
1.94
1.97
1.95
2.95
12.70
2.87
f1 (ppm)

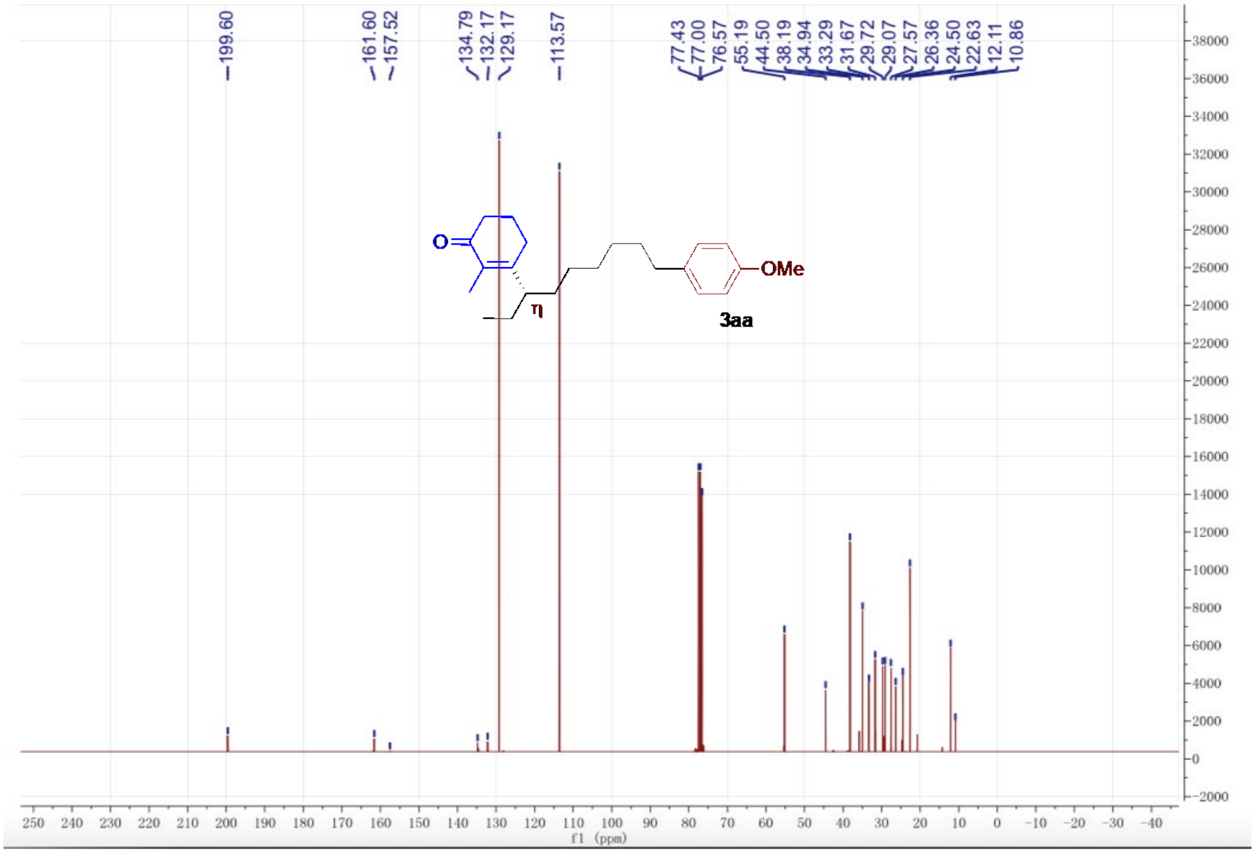
199.60
161.60
157.52
134.79
132.17
129.17
113.57
77.43
77.00
76.57
55.19
44.50
38.19
34.94
33.29
31.67
29.72
29.07
27.57
26.36
24.50
22.63
12.11
10.86
OMe
3aa
f1 (ppm)

As shown below, protons on the aryl ring of **3y** include Ha and Hb (1:1). Ha has a chemical shift of ~7.24 and Hb has a chemical shift of ~6.82. Similarly, protons on the aryl ring of side products **4y** have a chemical shift of ~7.06 and 6.82. From the 3y-^{1}H NMR, the total integration of **3y** is 1.48, **3y** (H-D) is 0.72 (0.36*2), and **3y** (D-H) is 0.38 (0.19*2), so the **3y** (D-D) is 0.38 (1.48-0.72-0.38). That is to say the ratio of **3y** (H-D), **3y** (D-H), and **3y** (D-D) is 72:38:38. As the overall yield of **3y** is 47%, we know the yields of every product (such as **3y** (H-D) is 47%*72/148=23%).

Product Distrubution

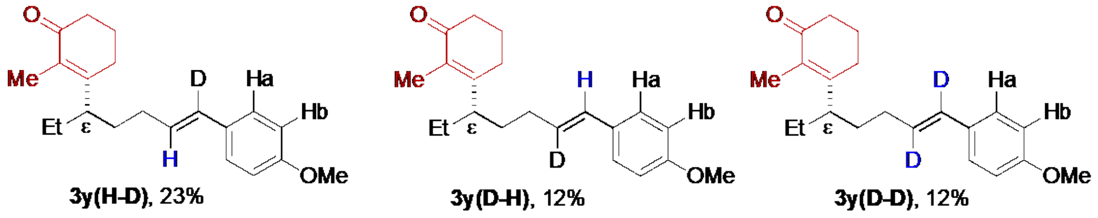

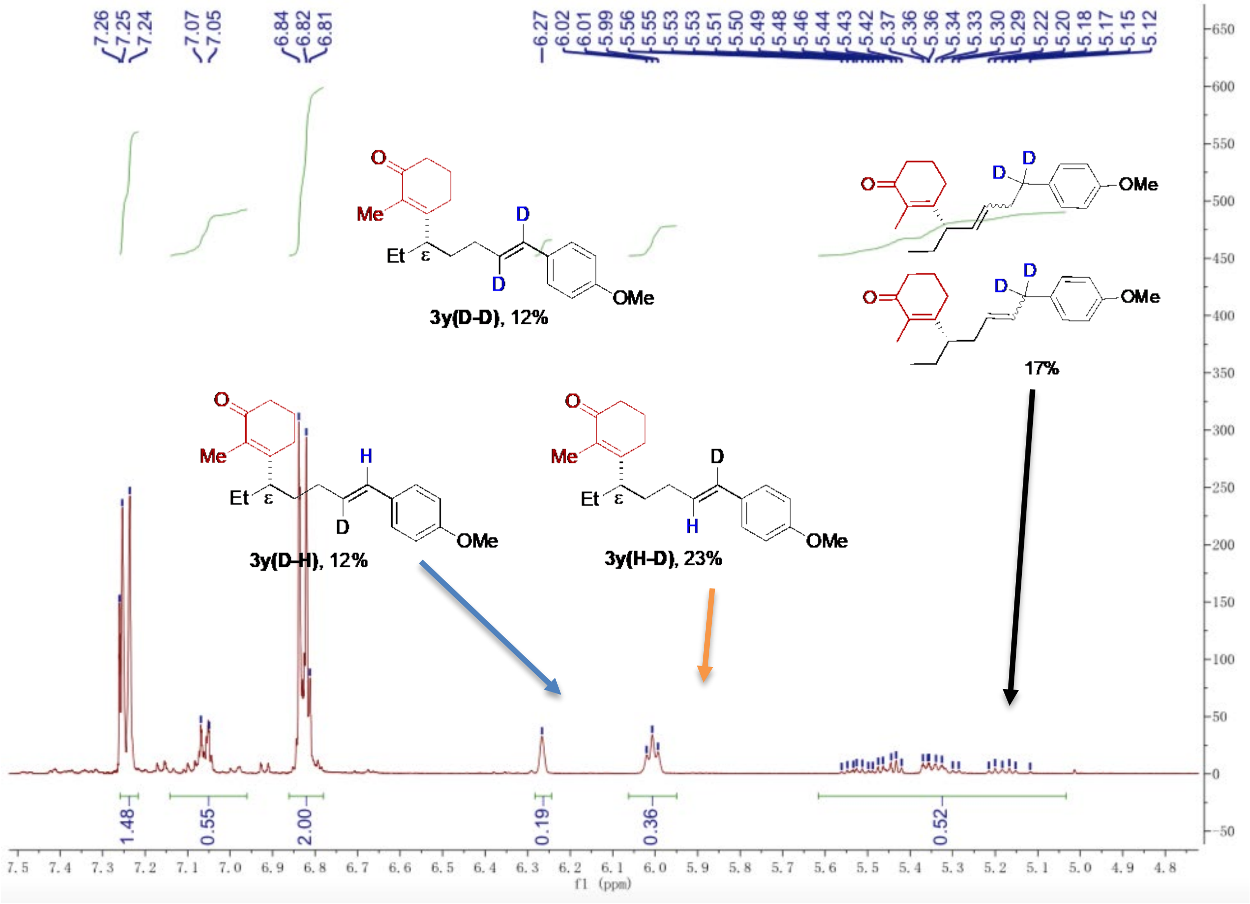

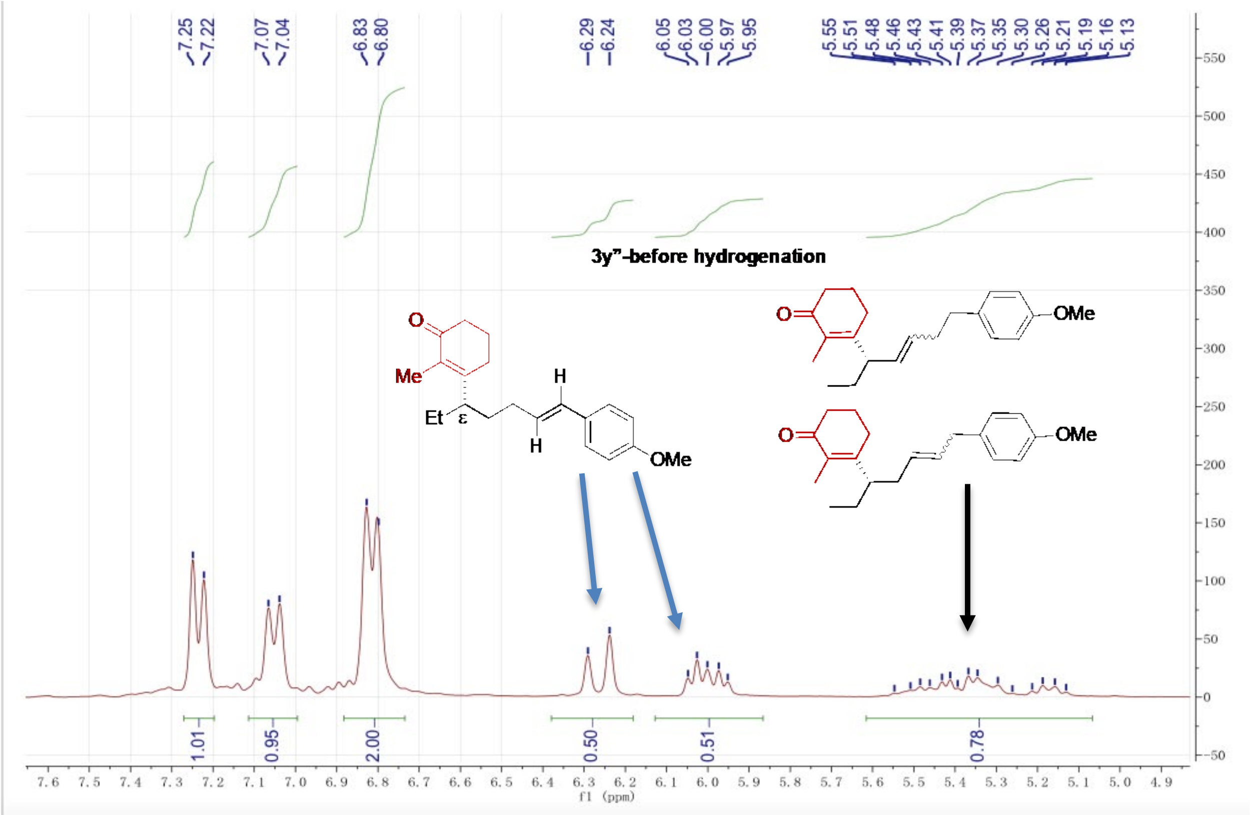

7.25
7.22
7.07
7.04
6.83
6.80
6.29
6.24
6.05
6.03
6.00
5.97
5.95
5.55
5.51
5.48
5.46
5.43
5.41
5.39
5.37
5.35
5.30
5.26
5.21
5.19
5.16
5.13
3y"-before hydrogenation
O
Me
Et
ε
H
H
OMe
O
OMe
O
OMe
1.01
0.95
2.00
0.50
0.51
0.78
f1 (ppm)

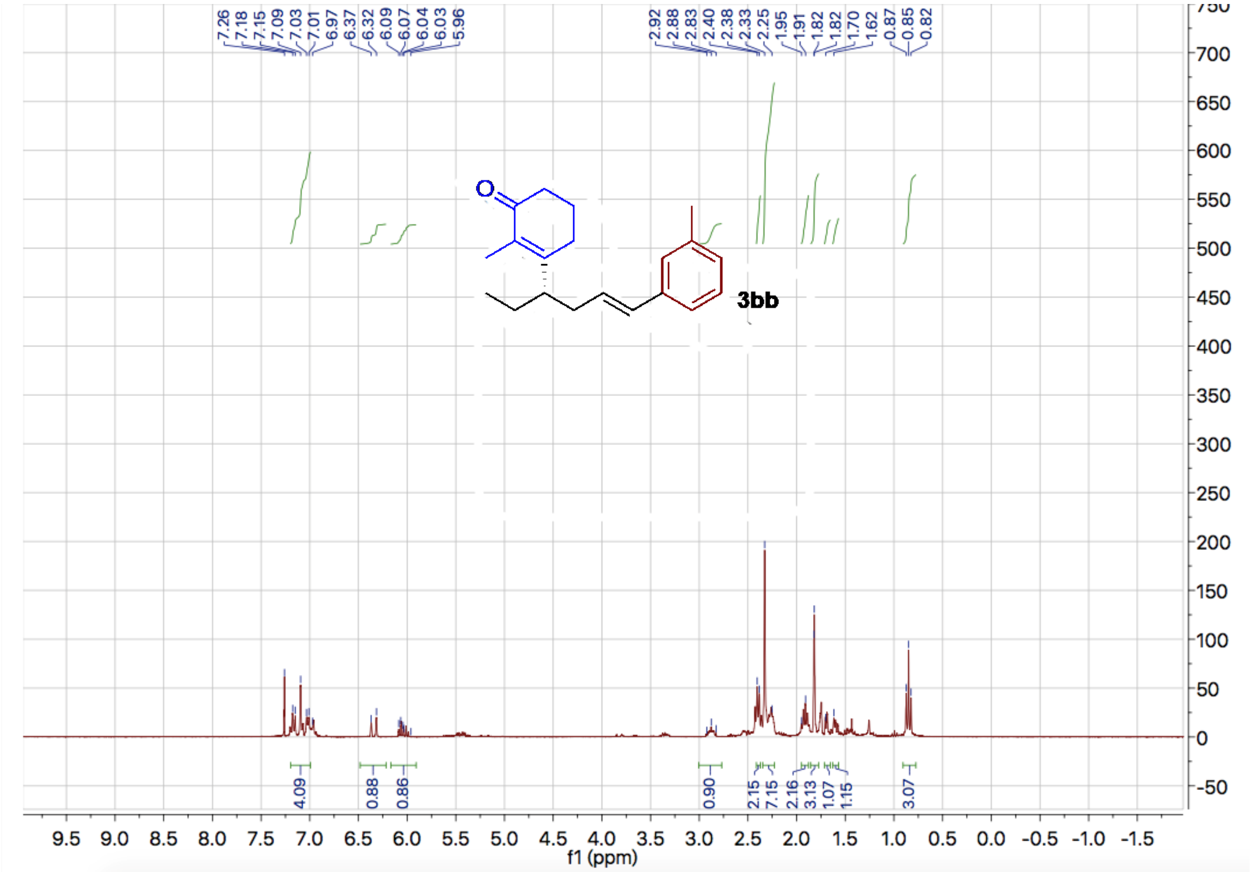
3bb
f1 (ppm)

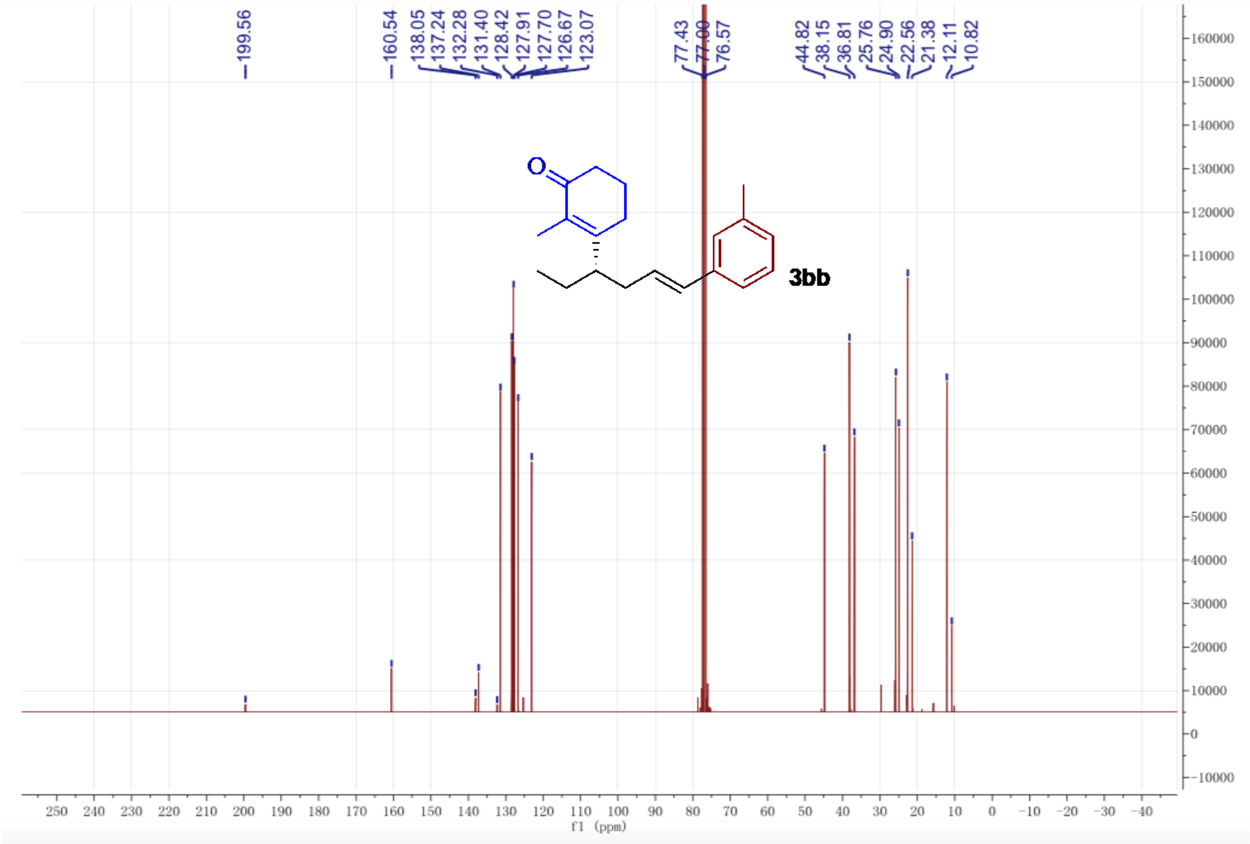
3bb
f1 (ppm)

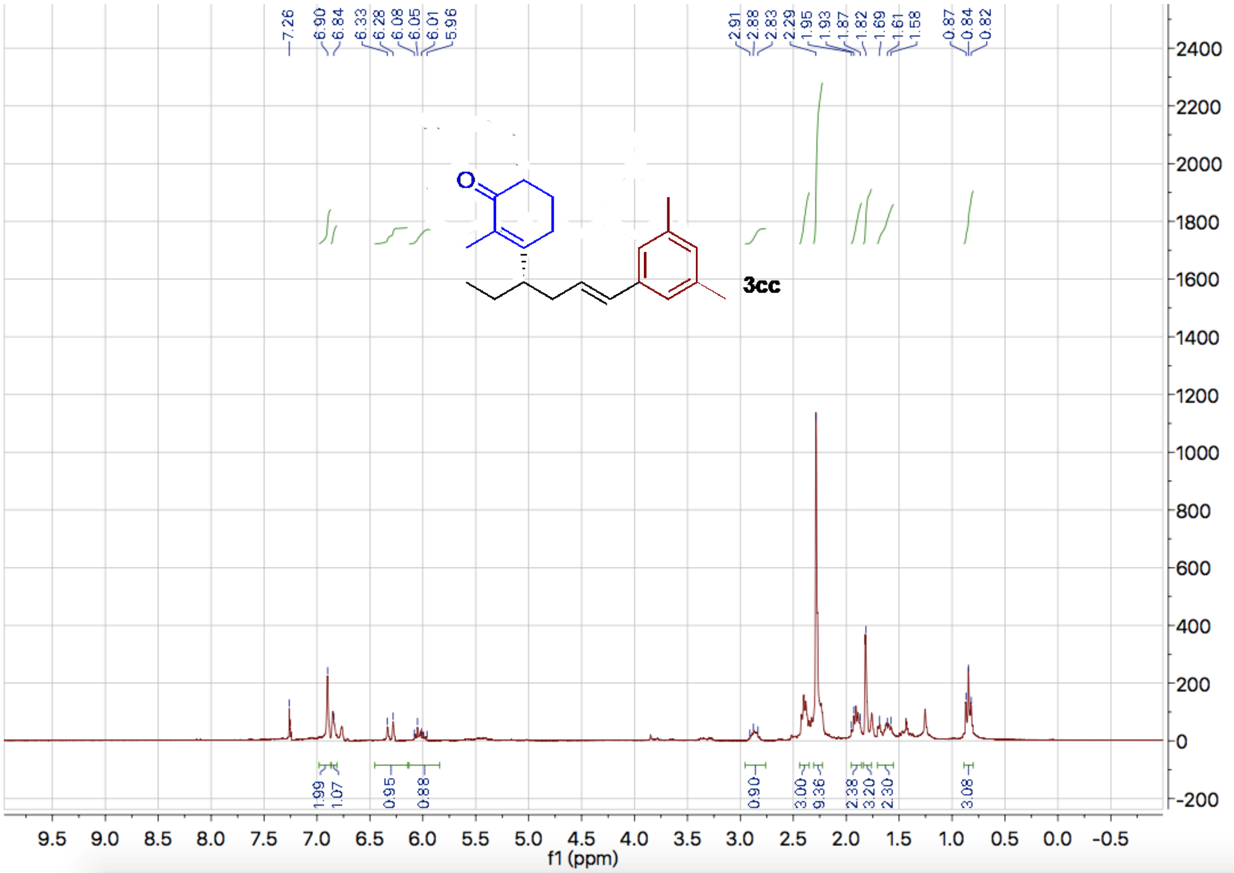
7.26
6.90
6.84
6.33
6.28
6.08
6.05
6.01
5.96
2.91
2.88
2.83
2.29
1.95
1.93
1.87
1.82
1.69
1.61
1.58
0.87
0.84
0.82
O
3cc
1.99
1.07
0.95
0.88
0.90
3.00
9.36
2.38
3.20
2.30
3.08
f1 (ppm)

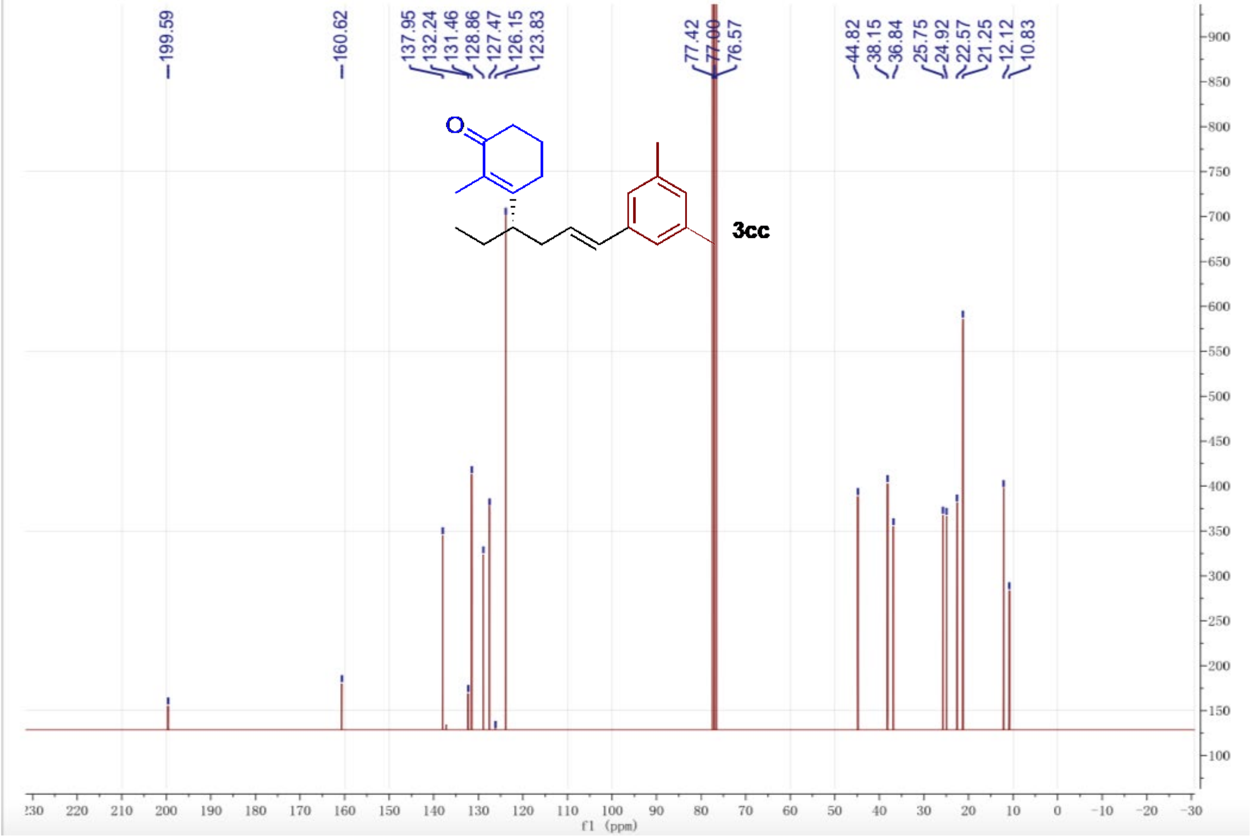
199.59
160.62
137.95
132.24
131.46
128.86
127.47
126.15
123.83
77.42
77.00
76.57
44.82
38.15
36.84
25.75
24.92
22.57
21.25
12.12
10.83
O
3cc
f1 (ppm)

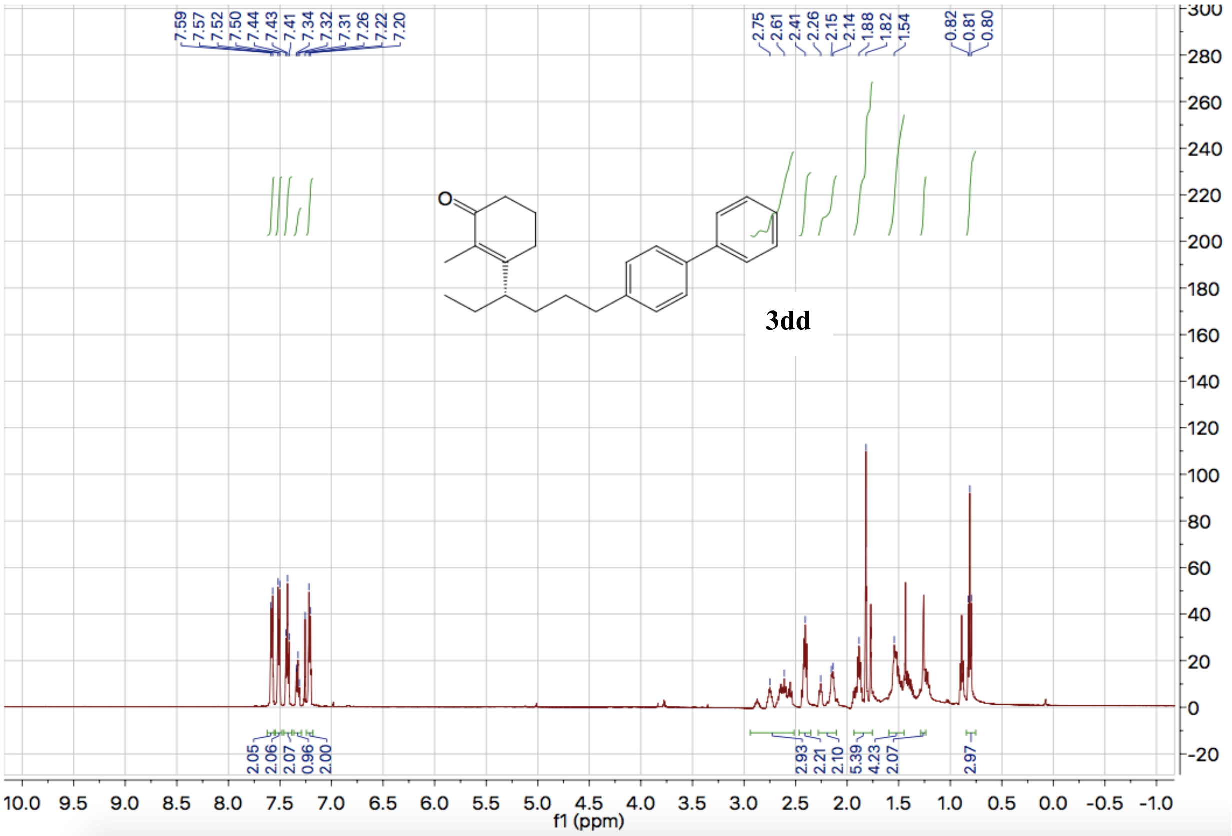

7.59
7.57
7.52
7.50
7.44
7.43
7.41
7.34
7.32
7.31
7.26
7.22
7.20
2.75
2.61
2.41
2.26
2.15
2.14
1.88
1.82
1.54
0.82
0.81
0.80
O
3dd
2.05
2.06
2.07
0.96
2.00
2.93
2.21
2.10
5.39
4.23
2.07
2.97
f1 (ppm)

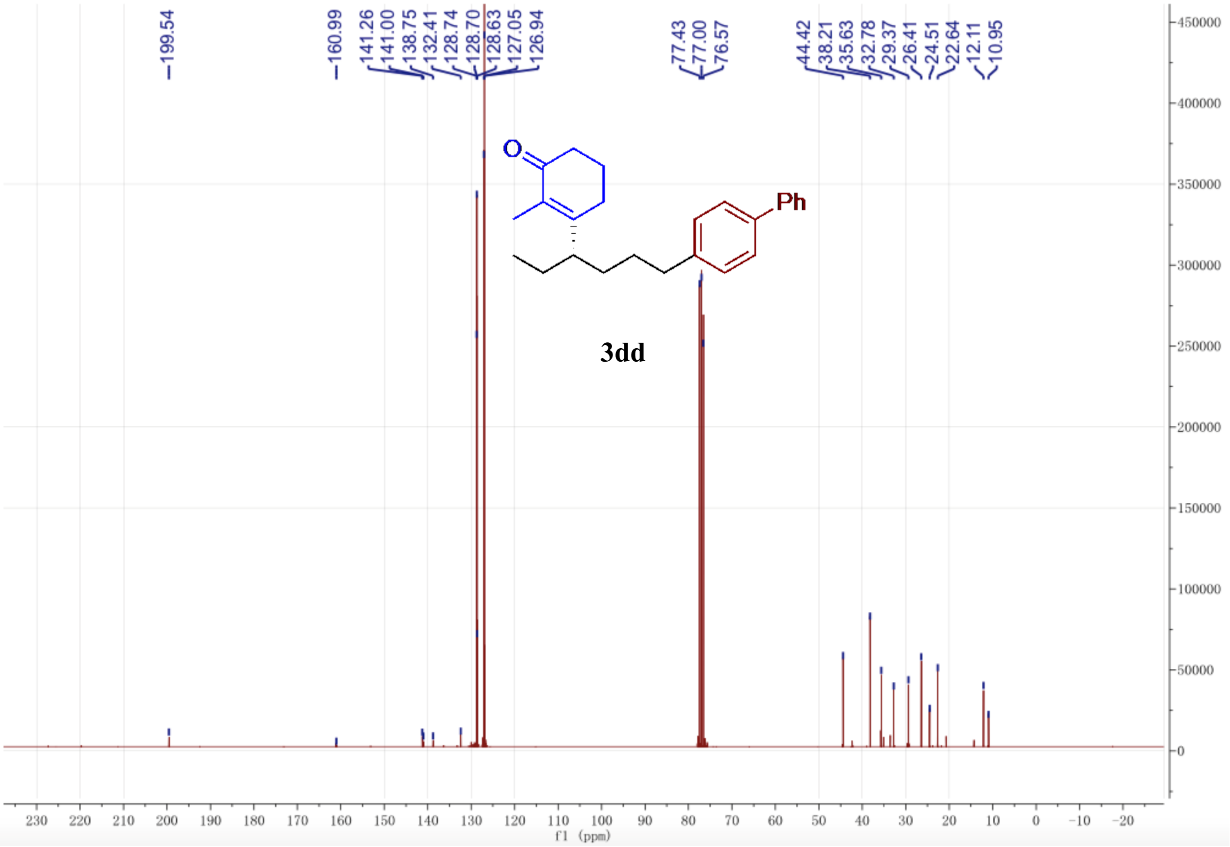

199.54
160.99
141.26
141.00
138.75
132.41
128.74
128.70
128.63
127.05
126.94
77.43
77.00
76.57
44.42
38.21
35.63
32.78
29.37
26.41
24.51
22.64
12.11
10.95
O
Ph
3dd
f1 (ppm)

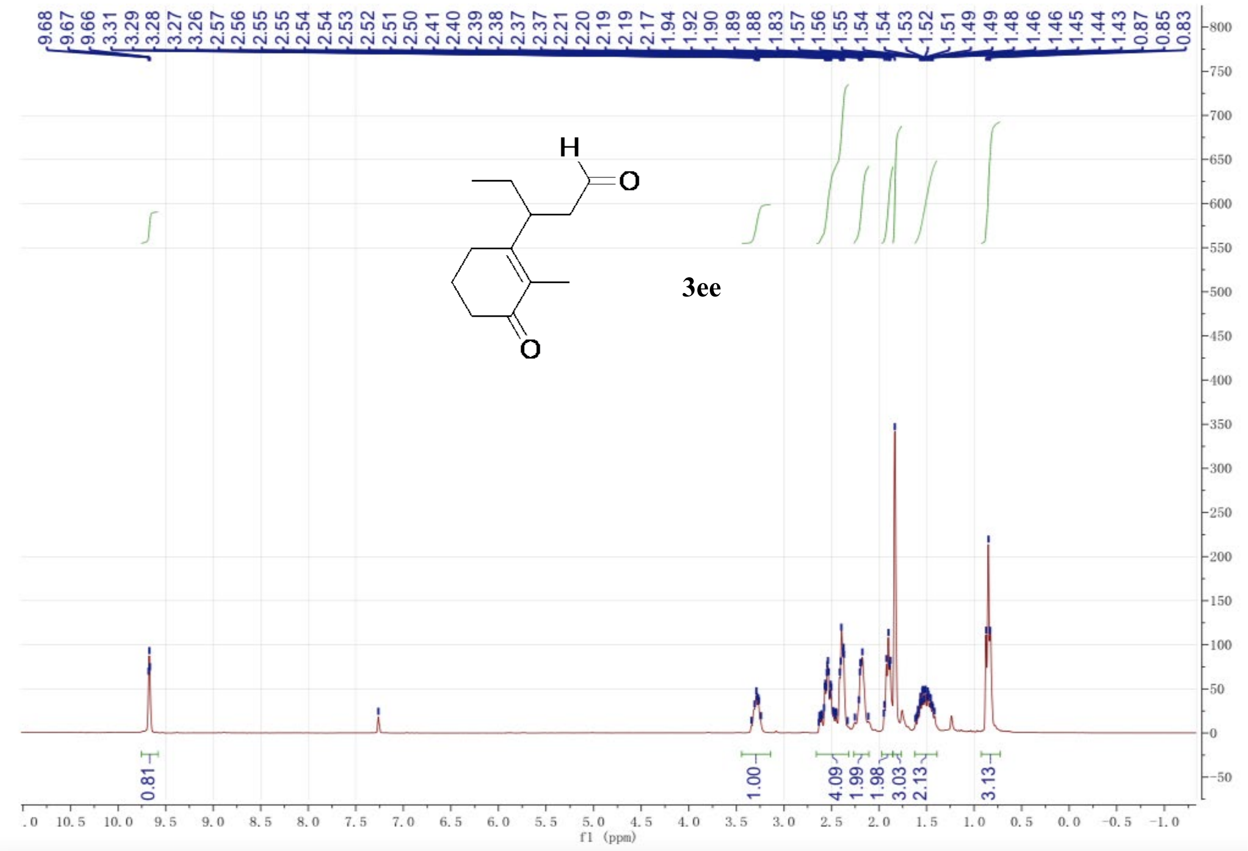
3ee
f1 (ppm)

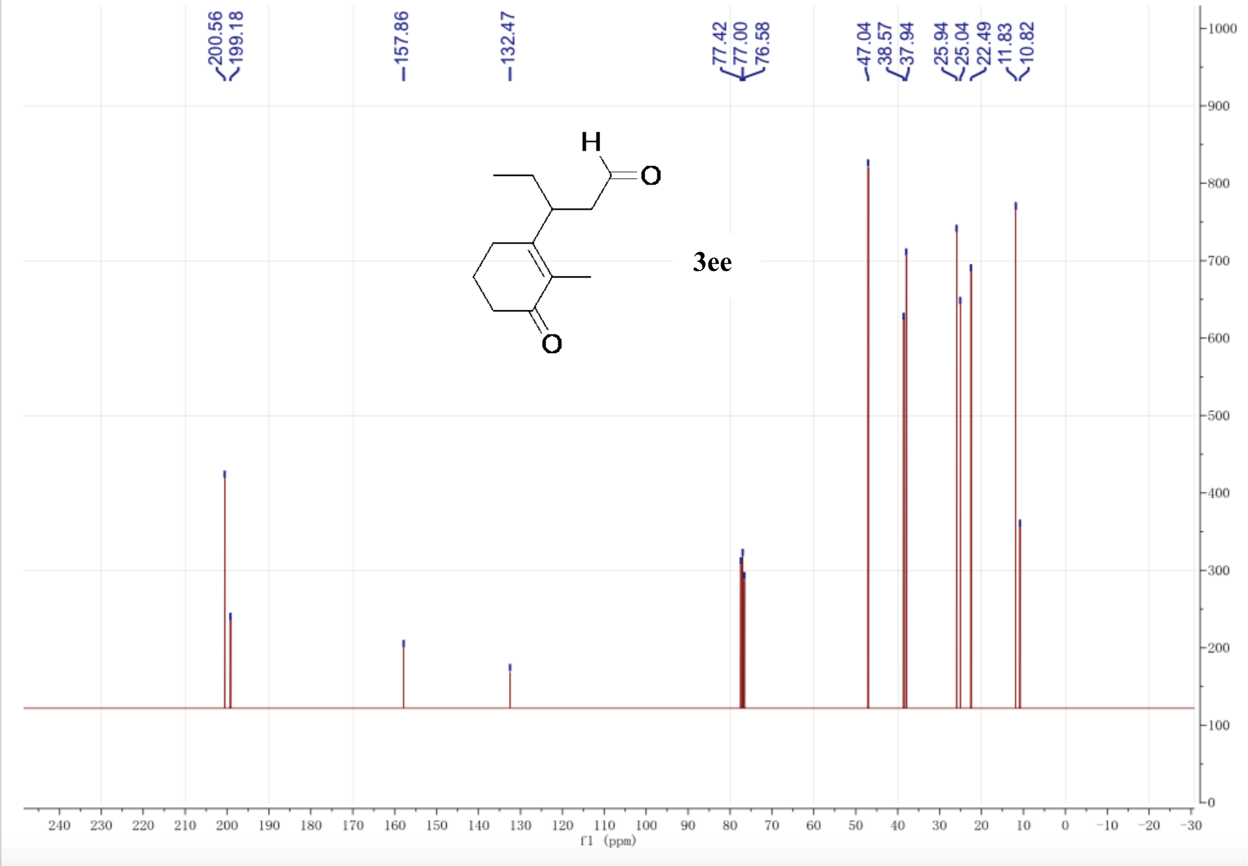
200.56
199.18
157.86
132.47
77.42
77.00
76.58
47.04
38.57
37.94
25.94
25.04
22.49
11.83
10.82
3ee
f1 (ppm)

The ^{1}H NMR of mixture for the correlation analysis describing the formation of alkene byproduct **4**

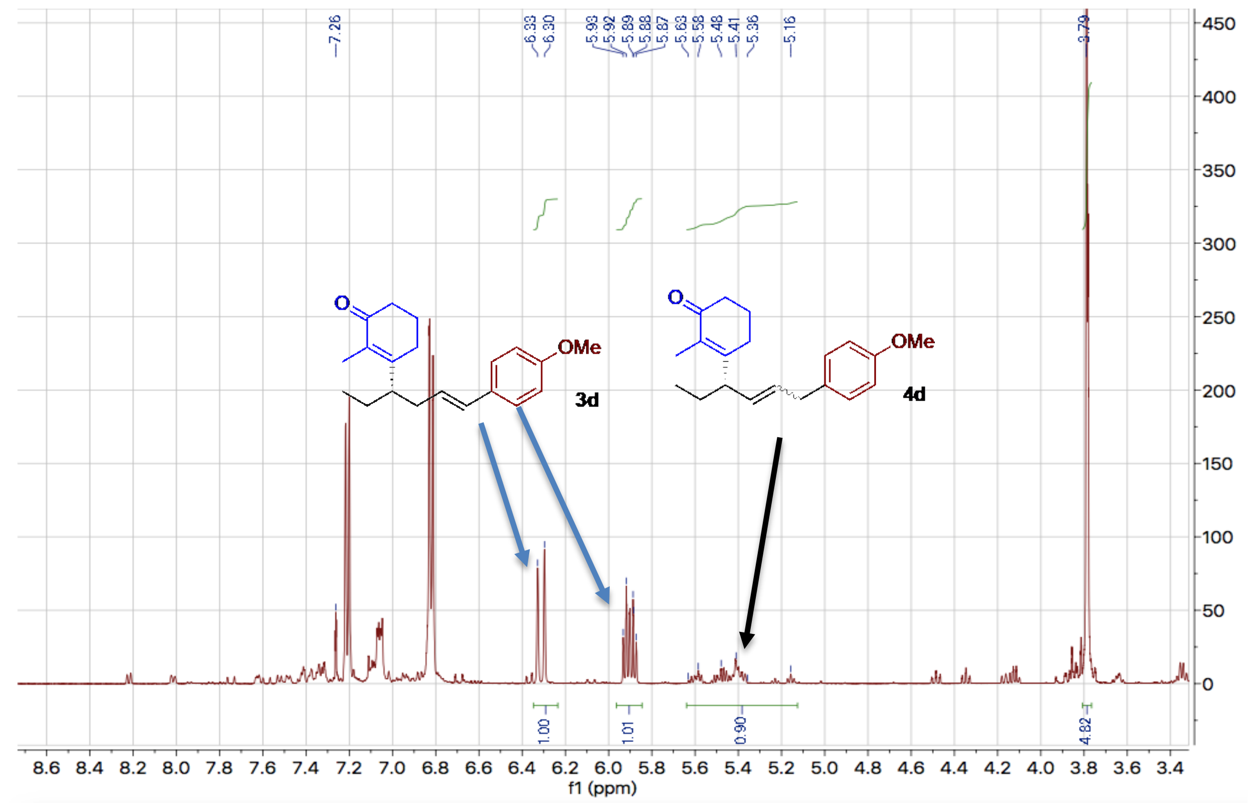

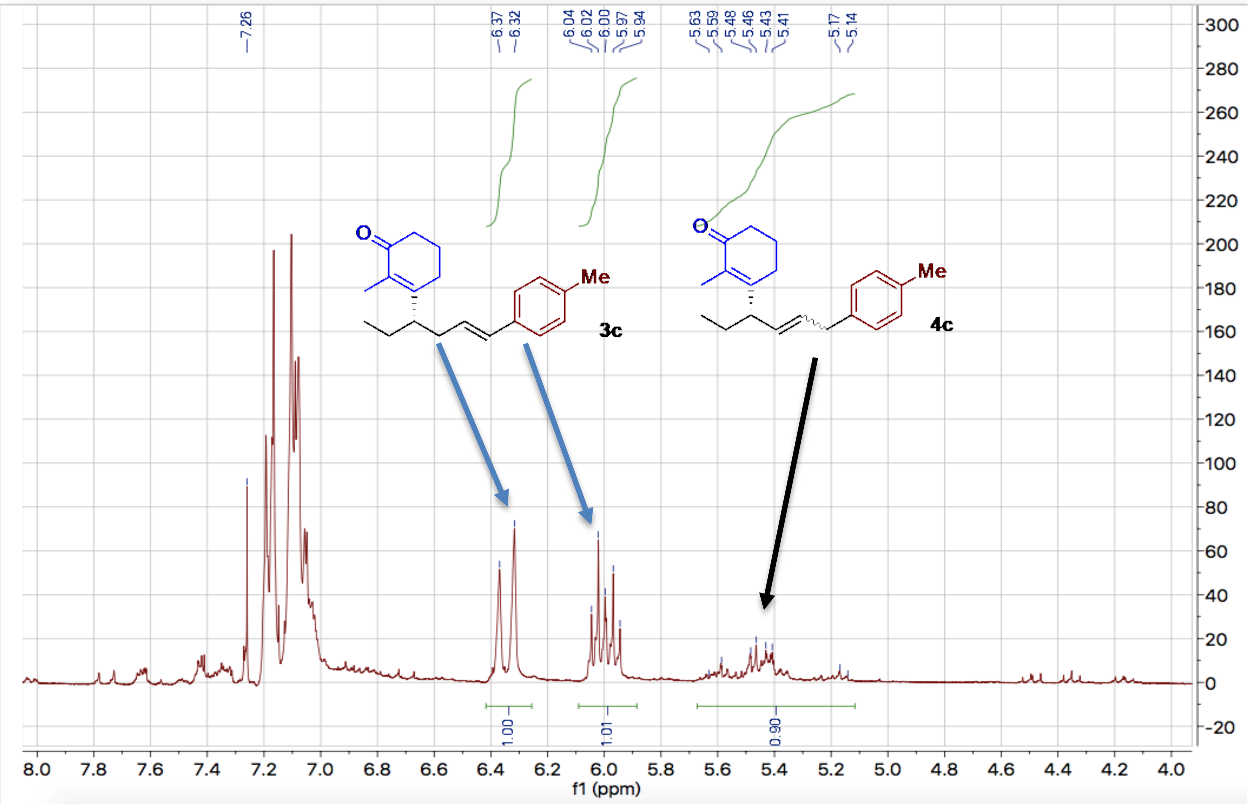

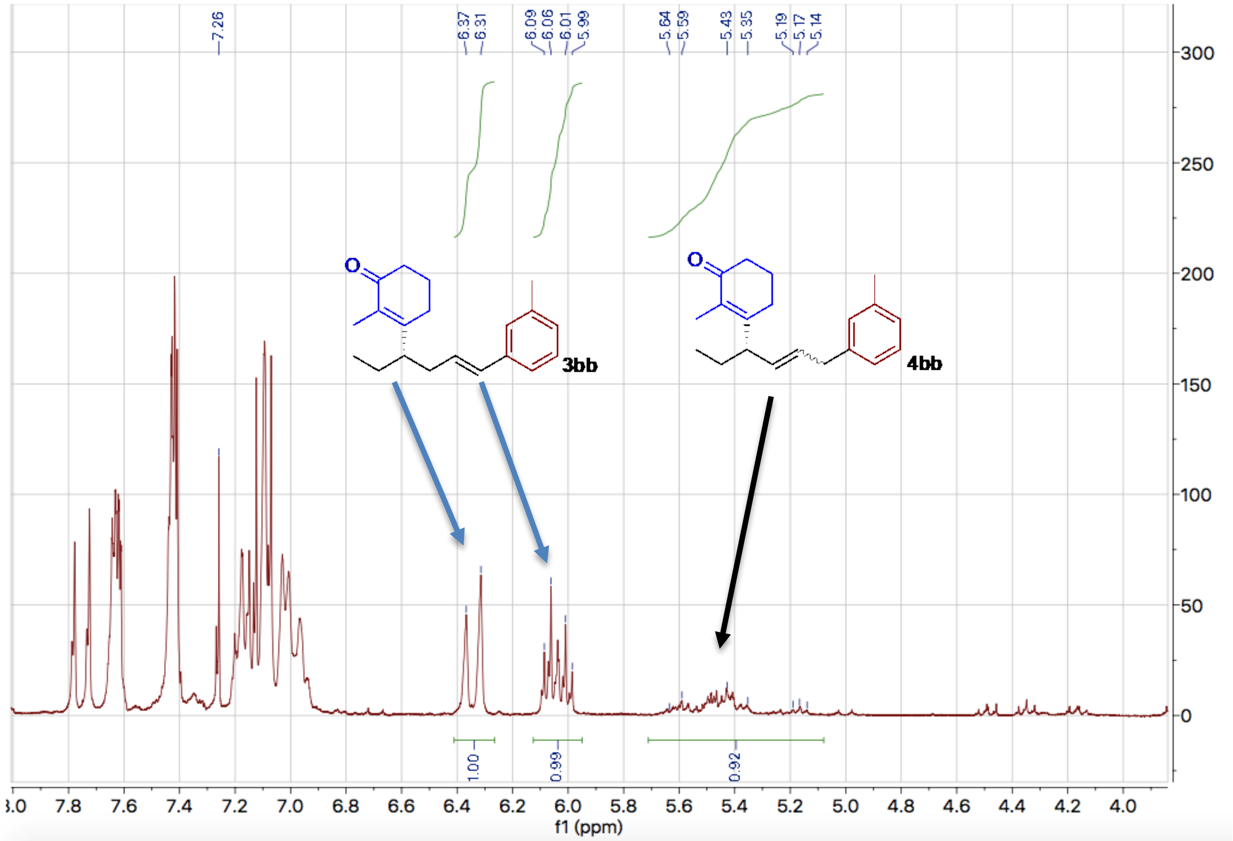
3bb
4bb
f1 (ppm)

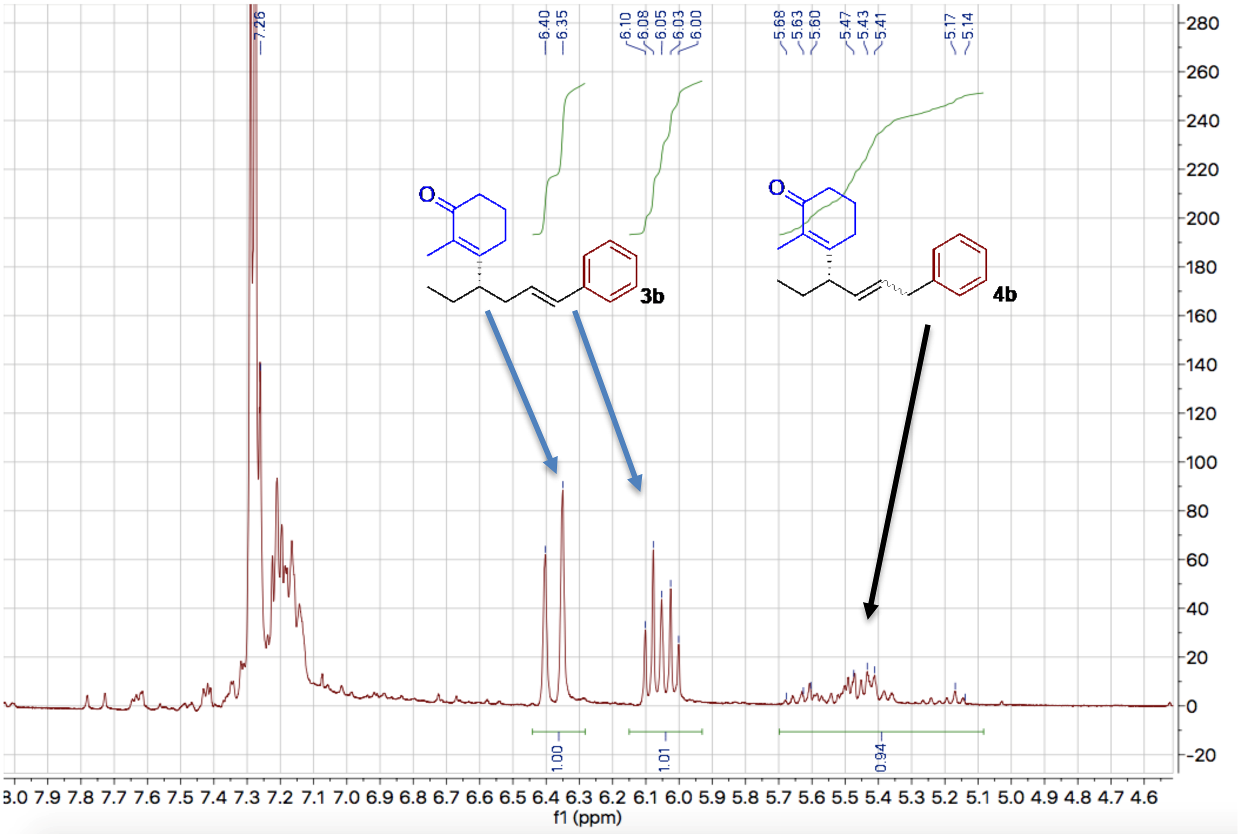
3b
4b
f1 (ppm)

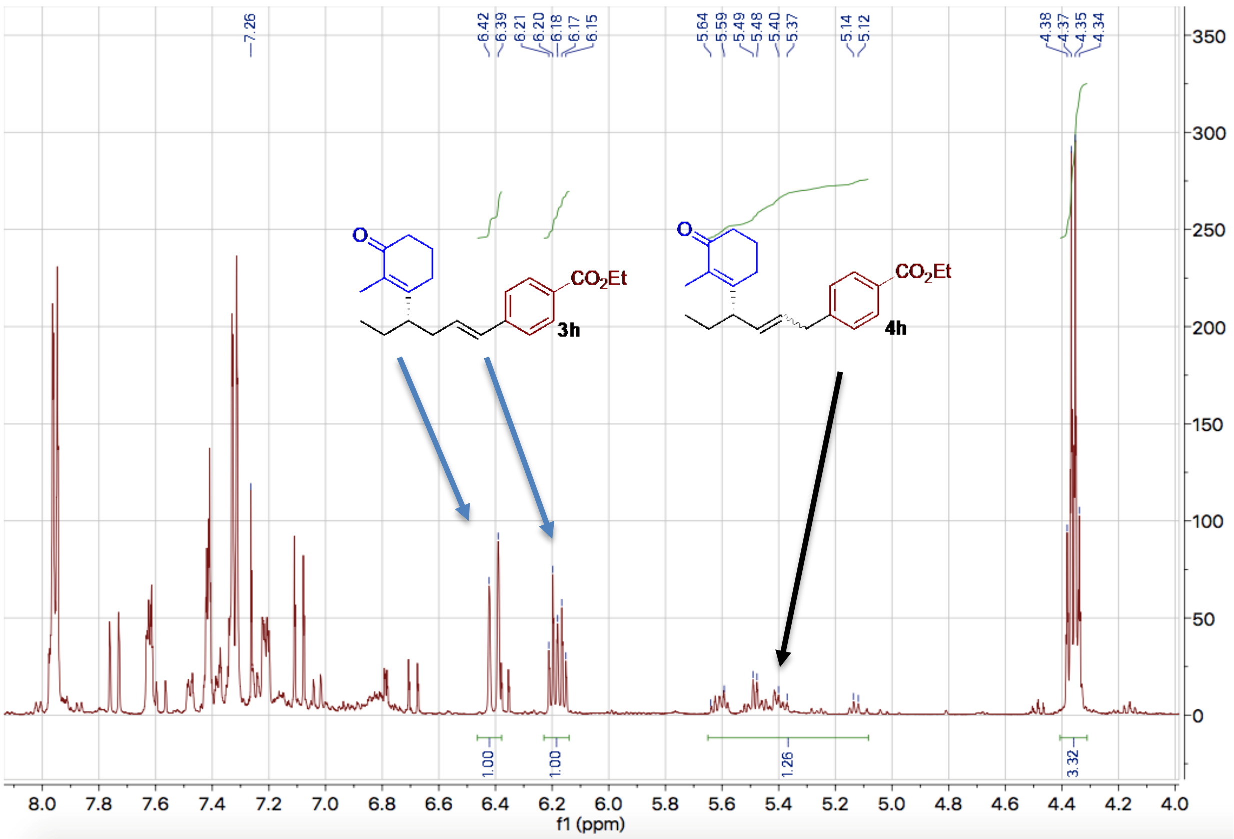
CO2Et
3h
4h
1.00
1.00
1.26
3.32
f1 (ppm)

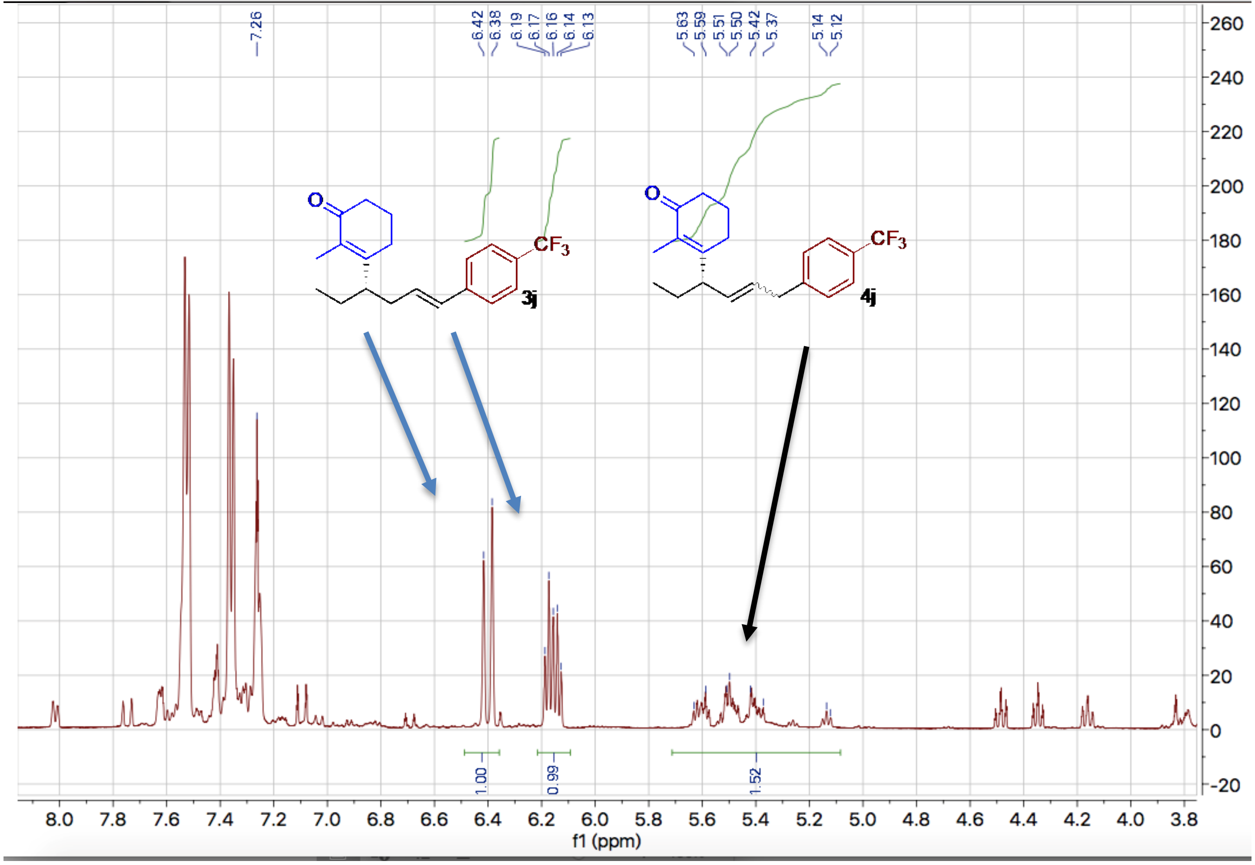
CF3
3j
4j
1.00
0.99
1.52
f1 (ppm)

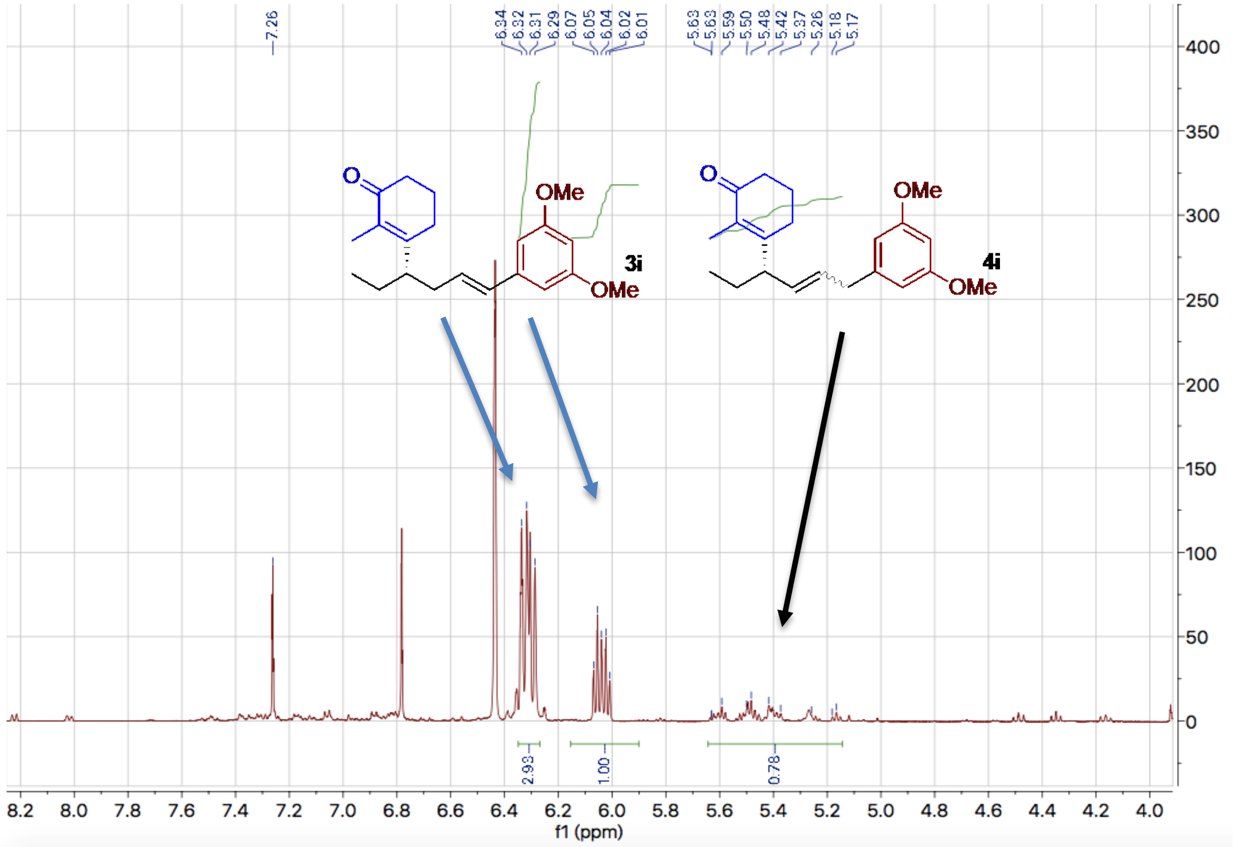

7.26
6.34
6.32
6.31
6.29
6.07
6.05
6.04
6.02
6.01
5.63
5.63
5.59
5.50
5.48
5.42
5.37
5.26
5.18
5.17
OMe
3i
OMe
OMe
4i
OMe
2.93
1.00
0.78
f1 (ppm)

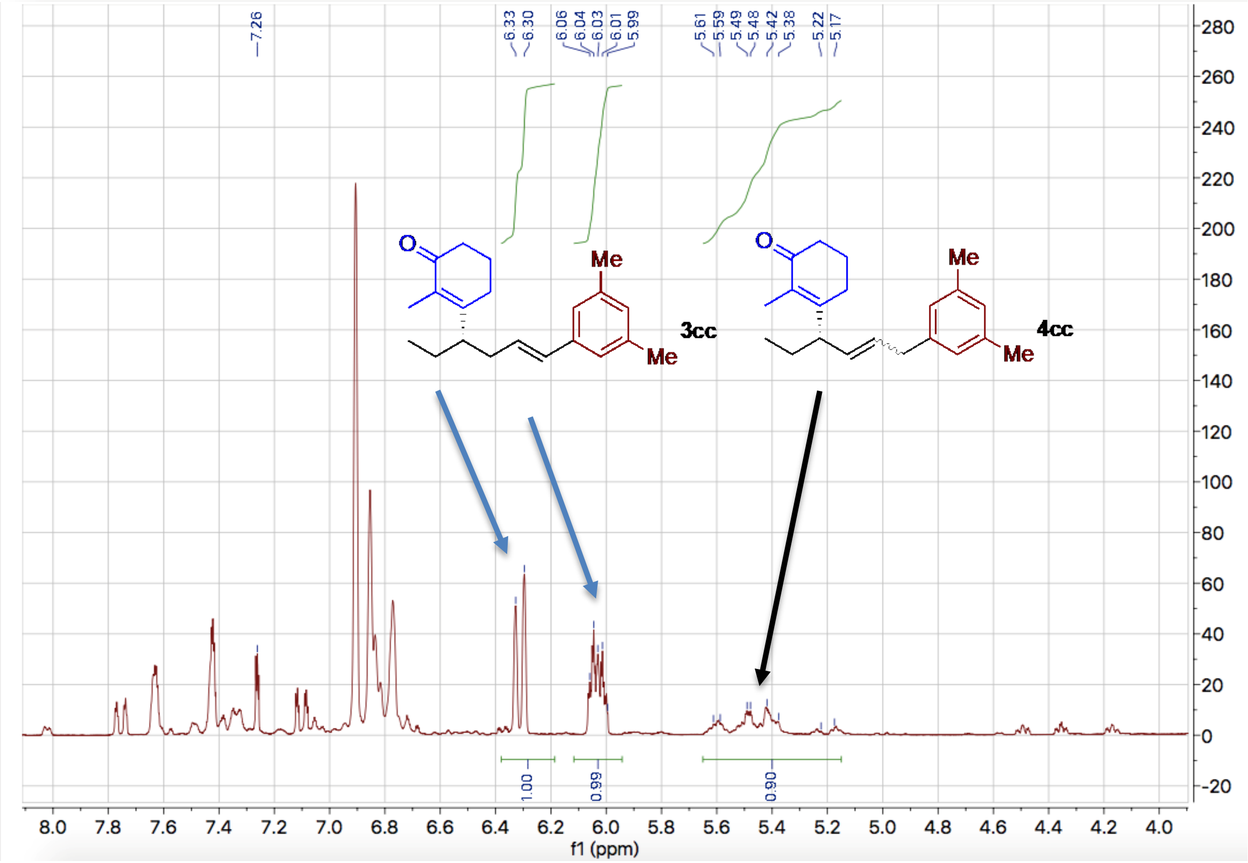

7.26
6.33
6.30
6.06
6.04
6.03
6.01
5.99
5.61
5.59
5.49
5.48
5.42
5.38
5.22
5.17
Me
3cc
Me
Me
4cc
Me
1.00
0.99
0.90
f1 (ppm)

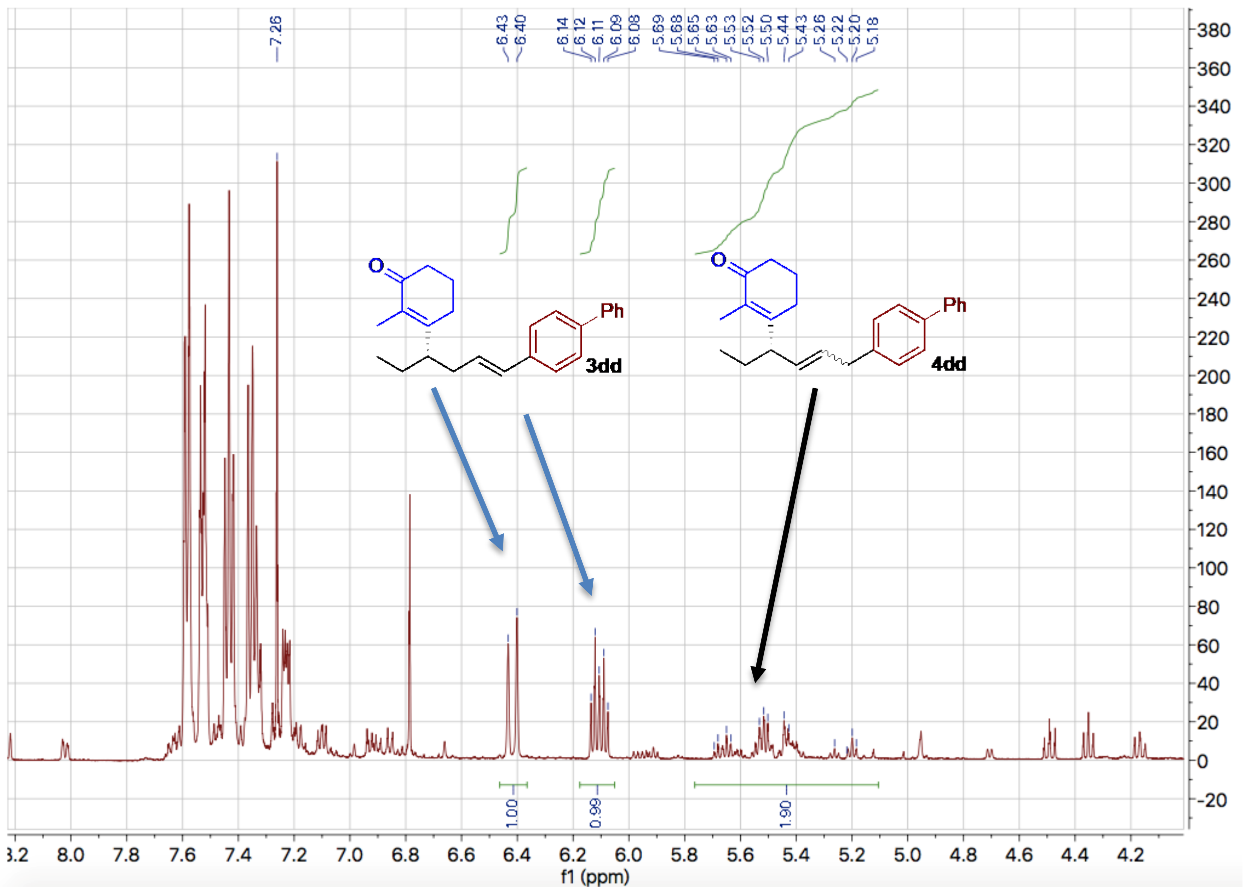

3dd
4dd
Ph
Ph
O
O
7.26
6.43
6.40
6.14
6.12
6.11
6.09
6.08
5.69
5.68
5.65
5.63
5.53
5.52
5.50
5.44
5.43
5.26
5.22
5.20
5.18
1.00
0.99
1.90
f1 (ppm)
